# Ni(0)-Catalyzed Efficient, Regioselective Synthesis of Dibenzo[*b*,*e*]oxepines and Dibenzo[*c*,*f*][1,2]oxathiepine 6,6-Dioxides: Mechanistic Study by DFT Calculation and Docking Interactions

**DOI:** 10.1021/acsomega.4c06569

**Published:** 2024-11-04

**Authors:** Uma Sankar Mandal, Sk Shamim Ahamed, Rabindranath Lo, Debashree Manna, Tapas Ghosh

**Affiliations:** † Department of Chemistry, Jadavpur University, Kolkata 700032, India; ‡ Institute of Organic Chemistry and Biochemistry, Czech Academy of Sciences, v.v.i., Flemingovo nám. 2, 16000 Prague 6, Czech Republic

## Abstract

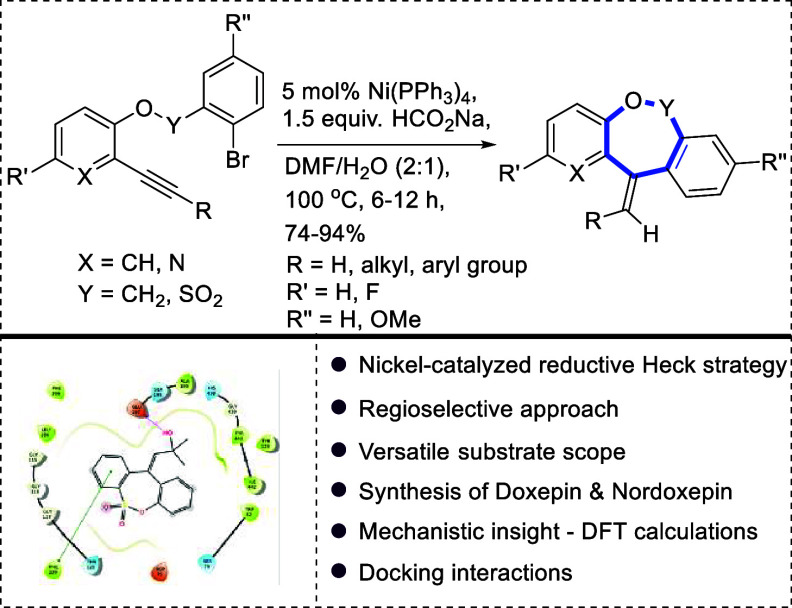

Herein, a nickel-catalyzed divergent reductive-Heck reaction of 1-bromo-2-((2-(aryl/alkyl ethynyl)phenoxy)methyl)benzene and 2-(aryl/alkyl ethynyl)phenyl 2-bromobenzenesulfonate derivatives has been demonstrated through the regulation of reducing agents and solvent systems. This scalable protocol offers regio- and stereoselective access to functionalized dibenzo[*b*,*e*]oxepine and dibenzo[*c*,*f*][1,2]oxathiepine 6,6-dioxide scaffolds in high to excellent yields under a mild set of reaction conditions. This methodology offers a predictable route for the synthesis of medium ring oxygen heterocycles and demonstrates wide substrate scope and outstanding tolerance to various functional groups like hydroxyl and, of course, practical instance via the synthesis of doxepin and nordoxepin molecules. We validate the experimentally proposed reaction mechanism using the density functional theory method. Further, molecular docking interactions were investigated accommodating some of our synthesized molecules.

## Introduction

Nickel-catalyzed reductive-Heck-type reactions for the construction of medium-ring heterocycles remain a major challenge to synthetic chemists, primarily owing to the energy barrier to the β-hydride elimination step being on the higher side for nickel compared to palladium systems. Earlier attempts were restricted to the usage of relatively reactive alkyl iodides and ended up with poor regioselectivity and reductive cyclization byproducts.^1^ Critical entropy and enthalpy factors along with transannular interaction make it demanding to form medium-sized ring heterocycles with ease.^2^ However, attempts were directed toward developing efficient protocols, including ring-closing metathesis^3^ and transition-metal-mediated cyclizations,^4^ etc., to access the oxacycles. Herein, we intend to develop a catalytic transformation that can proceed efficiently with outstanding olefin regioselectivity using nickel catalysts.

**Figure 1:**
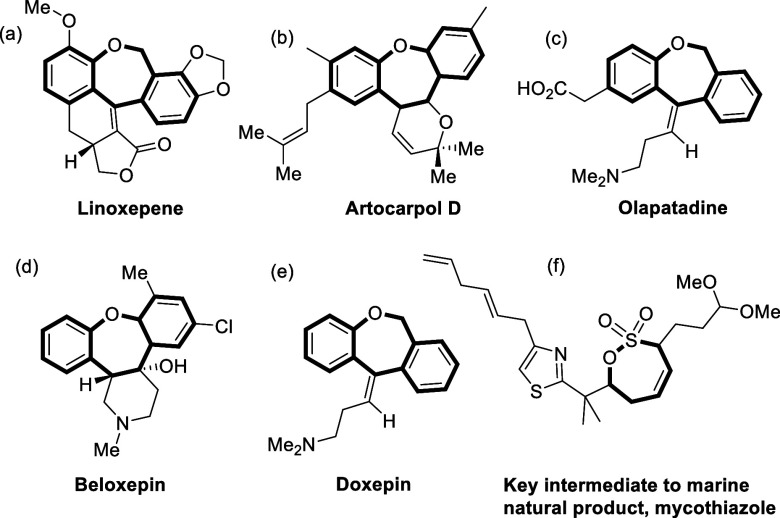
Natural products and APIs containing dibenzo[*b*,*e*]oxepine and sultone scaffolds.

Dibenzoxepine ring system constitutes the molecular skeleton of various biologically important natural products and active pharmaceutical ingredients.^5^ For instance, doxepin is widely used to treat depression, anxiety, and insomnia,^6^ and olopatadine, an antihistamine, anticholinergic drug, is used to treat allergic eye (Figure 1).^7^ Dibenzoxepines are also found in linoxepin, used as herbal remedies for pain and rheumatoid arthritis^8^; beloxepin acts as 5-HT2 receptor and norepinephrine transporter; artocarpol D is shown to have potent anti-inflammatory properties.^9^ Insubstantial approaches leading to the formation of the dibenzoxepine scaffold involved either the palladium-catalyzed cascade carbometalation followed by cross-coupling of alkynes or dehydration of carbinols and other methods.^10^ Therefore, the development of an efficient methodology for the synthesis of dibenzo[*b*,*e*]oxepines with convertible functional groups is of immense interest. Dibenzo[*c*,*f*][1,2]oxathiepine 6,6-dioxide (sultones) being recognized as the internal esters of hydroxyl sulfonic acids are utility molecules due to their possible modification in flexible fashion and scope to act as key intermediates in the total synthesis of eriolanin, pamamycin-607, eriolangin, and marine natural product mycothiazole.^11^ Major bioactivity of sultone scaffolds are engaged with toxicity, skin sensitization, and antiviral properties.^12^

Cross-coupling reactions involving transition metals are undoubtedly used vividly in organic transformations.^13^ In usual practice, aryl halides are employed frequently as the electrophilic terminals activated by metal catalysts.^14^ In this context, unquestionably, palladium has ruled synthetic methodologies for many decades when it comes to C–C bond construction, although it has been assigned as one of the premier elements facing scarcity. Nickel, generated in large scale every year and many-fold less expensive compared to palladium, iridium, and other metals, is used for similar purposes.^15,16^ Besides, nickel catalysts are well-known to assist Heck-type arylation and^17^ allylation^18^ of olefins and/or alkynes along with cascade, hydroarylation, etc.^19^ Since seminal report back in the late 1980s from Ronchi and co-workers on nickel-catalyzed reductive Heck-type reactions, tremendous advancements have been made paving avenues for organic transformations difficult erstwhile.^20^

Mondal et al. developed a palladium-catalyzed route to prepare medium-ring pyridine-fused benzoxepine derivatives and fused sultones.^21^ Their studies were limited to aromatic alkynes and tolerated by electron-donating groups in the aryl scaffold only. As a continuation of our ongoing interest in nickel-catalyzed hydroarylation, cascade reaction along with reductive electrophilic functionalization of olefins/alkynes and following the recent progress in hydrofunctionalization reaction,^22^ we envisage a nickel-catalyzed intramolecular reductive-Heck reaction of 1-bromo-2-((2-(alkyl/arylethynyl)phenoxy)methyl)benzene and 2-(alkyl/arylethynyl)phenyl 2-bromobenzenesulfonate with the assistance of a stoichiometric amount of a reducing agent to afford an array of benzene-fused tricyclic compounds bearing a trisubstituted exocyclic olefin. Extensive computational studies reveal the regioselectivity of the protocol and provide energy profiles that are in line with the experimental observation.

## Results and Discussion

**Scheme 1:**
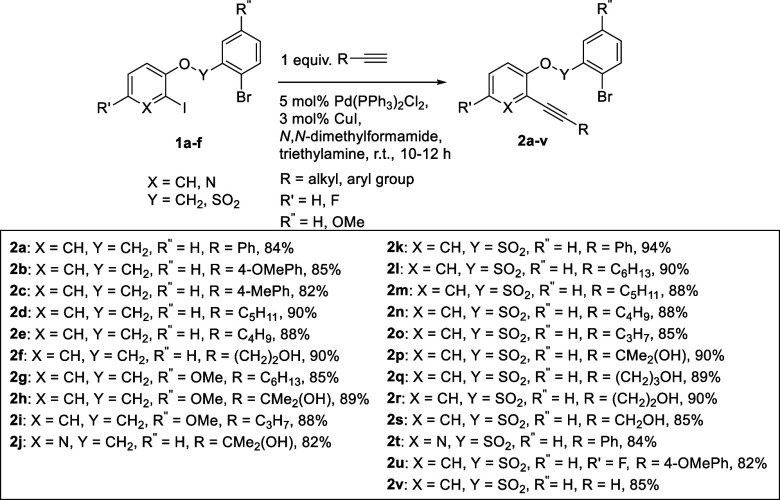
Synthesis of Dibenzo[*b*,*e*]oxepine and Sultone Precursors **2a**–**v**

Synthetic route to substrates of our interest **2a**–**v** is outlined in Scheme 1. Reaction of 2-iodophenol with 1-bromo-2-(bromomethyl)benzene (or 2-bromo-4-methoxybenzyl bromide) and 2-bromobenzenesulfonyl chloride using literature precedents furnished desired substrates **1a**–**f**. Further, the dibenzo[*b*,*e*]oxepine and sultone precursors **2a**–**v** were prepared by the chemoselective Sonogashira reaction of compounds **1a**–**f** with various alkyne counterparts in 82–94% yields (Scheme 1).^23^ At the outset of our studies, we investigated the reaction employing compound **2g** as the model substrate (Table 1) with a wide variation of nickel catalysts. The reductive-Heck reaction was initially attempted using **2g** along with various nickel catalysts, HCO_2_Na as the reducing agent, and *N*,*N*-dimethylformamide–water as a preferred solvent system (entries 1–6, Table 1). These reactions were unsuccessful, or only a trace amount of the desired product (**3g**) was obtained in these cases. Gratifyingly, the desired product was obtained in 72% yield on the treatment of the substrate with tetrakis(triphenylphosphine)nickel(0) and the reducing agent HCO_2_Na in *N*,*N*-dimethylformamide–water (2:1) solvent for 24 h at 100 °C temperature (entry 7, Table 1). To our delight, the yield was enhanced (85%) by reducing the reaction time to 12 h (entry 8, Table 1). Reducing the reaction time further led to decreased yield only (entry 9, Table 1).

**Table 1:**
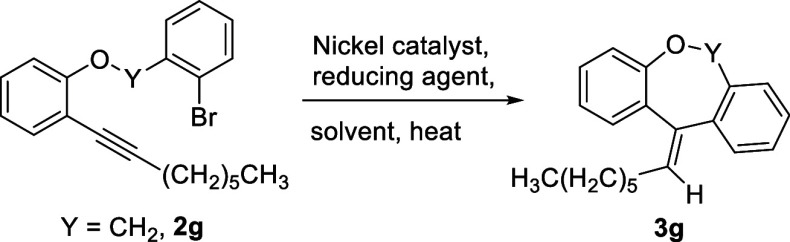
Screening for the Optimized Reaction Conditions

entry	catalyst (mol %)	reducing agent	solvent (v/v)	time (h)	temperature (°C)	yield^b^ (%)
1.	Ni(OAc)_2_(5)	HCO_2_Na	DMF-H_2_O	24	100	nr
2.	NiCl_2_(5)	HCO_2_Na	DMF-H_2_O	24	100	nr
3.	NiBr_2_(5)	HCO_2_Na	DMF-H_2_O	24	100	nr
4.	Ni(acac)_2_(5)	HCO_2_Na	DMF-H_2_O	24	100	18
5.	NiCl_2_(PPh_3_)_2_(5)	HCO_2_Na	DMF-H_2_O	24	100	20
6.	Ni(COD)_2_(5)	HCO_2_Na	DMF-H_2_O	24	100	nr
7.	Ni(PPh_3_)_4_(5)	HCO_2_Na	DMF-H_2_O	24	100	72
8.	Ni(PPh_3_)_4_(5)	HCO_2_Na	DMF-H_2_O	12	100	80
9.	Ni(PPh_3_)_4_(5)	HCO_2_Na	DMF-H_2_O	6	100	30
10.	Ni(PPh_3_)_4_(20)	HCO_2_Na	DMF-H_2_O	12	100	81
11.	Ni(PPh_3_)_4_(3)	HCO_2_Na	DMF-H_2_O	12	100	62
12.	Ni(PPh_3_)_4_(5)	HCO_2_Na	DMF	12	100	nr
13.	Ni(PPh_3_)_4_(5)		DMF	12	100	nr
14.	Ni(PPh_3_)_4_(5)	HCO_2_Na	DMF-^*i*^PrOH	12	100	74
15.	Ni(PPh_3_)_4_(5)	HCO_2_Na	*^i^*PrOH	12	100	nr
16.	Ni(PPh_3_)_4_(5)	HCO_2_Na	isobutanol	12	100	decomp.
17.	Ni(PPh_3_)_4_(5)	HCO_2_Na	DMF-D_2_O	12	100	no deuteriated pdt
18.	Ni(PPh_3_)_4_(5)	HCO_2_Na	DMF-H_2_O	12	120	55
19.	Ni(PPh_3_)_4_(5)	HCO_2_Na	DMF-H_2_O	12	80	46
20.	Ni(PPh_3_)_4_(5)	HCO_2_Na	DMF-H_2_O	12	100	20
21.	Ni(PPh_3_)_4_(5)	HCO_2_Na	toluene-H_2_O	12	100	nr
22.	Ni(PPh_3_)_4_(5)	HCO_2_Na	DMF-H_2_O	12	100	65
23.	Ni(PPh_3_)_4_(5)	HCO_2_Na	MeCN-H_2_O	12	100	42
24.	Ni(PPh_3_)_4_(5)	HCO_2_NH_4_	DMF-H_2_O	12	100	76
25.	Ni(PPh_3_)_4_(5)	HCO_2_NH_4_	DMF-H_2_O	12	120	49
26.	Ni(PPh_3_)_4_(5)	HCO_2_NH_4_	DMF-H_2_O	12	80	40

a Reducing agents were used in 1.5 equiv. in all cases.

b Isolated yield.

On enhancement of catalyst loading (20 mol %), the yield was not significantly improved (81%, entry 10, Table 1). A decrease in the catalyst loading (3%) led to a lowering of yield (entry 11, Table 1) of the desired product. The reaction did not proceed in the absence of the reducing agent or water (entries 12–13, Table 1). Replacing water with isopropyl alcohol furnished encouraging yield of the desired product (entry 14, Table 1). However, the reaction did not initiate or decomposition occurred with sole isopropanol or isobutanol solvent (entries 15–16, Table 1). To look in-depth for the origin of olefinic hydrogen in the exocyclic double bond of the tricyclic product, we run the reaction in the presence of nickel(0) catalyst and D_2_O with other conditions intact. In parity with earlier reports, we find no deuterated product.^21^ Sodium formate plays a pivotal role by acting as the source of exocyclic olefinic hydrogen, and water is involved in pair separation of sodium formate. Varying reaction temperatures did not produce any satisfactory result (entries 18–19, Table 1). The yield of product **3g** was not improved at all on changing the reaction medium and reducing agent (entries 20–26, Table 1). Thus, we found the optimized reaction conditions for this reaction using HCO_2_Na as a reducing agent in *N*,*N*-dimethylformamide–water (2:1) solvent at 100 °C to produce the desired dibenzo[*b*,*e*]oxepine derivative (**3g**) in 12 h with 85% yield (entry 8, Table 1).

**Scheme 2:**
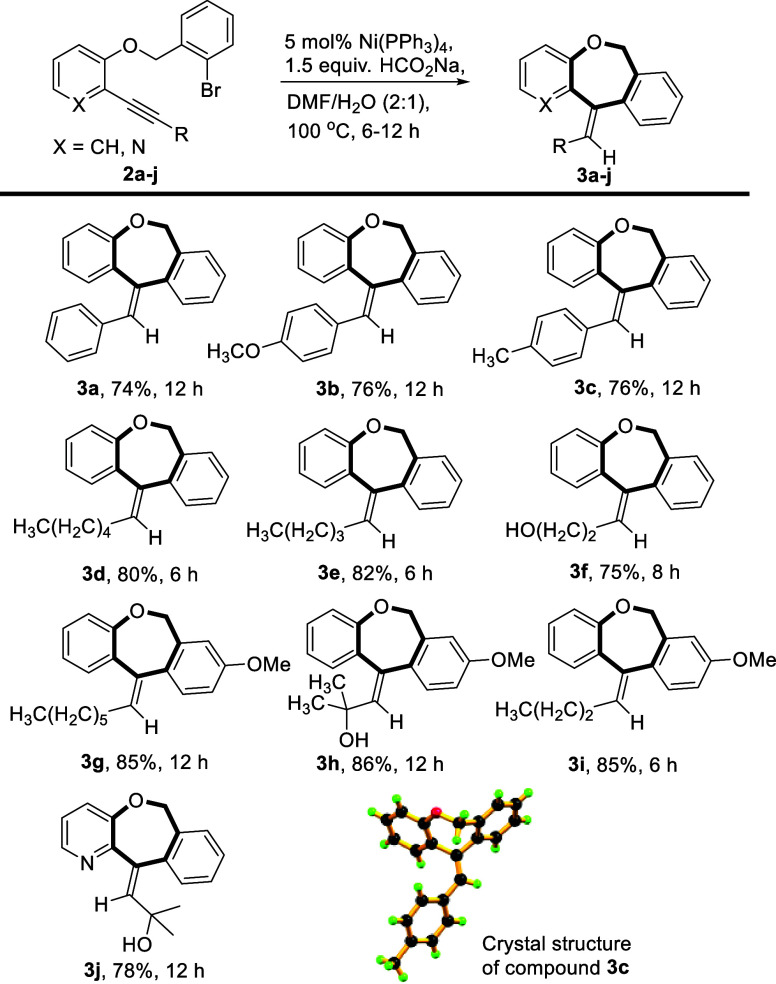
Substrate Scope to Access Dibenzo[*b*,*e*]oxepine Derivatives

The general applicability of the developed reaction conditions (entry 8, Table 1) using various substituted 1-bromo-2-((2-ethynylphenoxy)methyl)benzene (**2a−f**) and (**2h−j**) to obtain functionalized dibenzo[*b*,*e*]oxepine derivative moieties (3a−f) and (**3h−j**) were framed in Scheme 2. Aryl and alkyl substituents in the alkyne terminal of substrate **2** were well tolerated under these optimized reaction conditions.

Aliphatic alkynes furnished better yields (**3d**–**3f**, 75–82%) compared to aromatic alkynes (**3a**–**3c**, 74–76%). Even the hydroxyl functional group bearing alkyne **2f** smoothly produced a benzoxepine derivative in high yield (**3f**, 75%). The substrates with methoxy substitution at the phenyl ring of the benzyl bromide part (**2g**–**i**) also showed good reactivity and regioselectivity for the current transformations to furnish higher yields of cyclized products **3g**–**i** (85–86%). The aromatic ring with a phenolic–OH group was also replaced with a heteroarylpyridine scaffold and furnished the desired product **3j** in 78% yield under the same set of optimized reaction conditions. The NMR spectra are reported in Figures S1–S47. Moreover, we were successful in obtaining the single-crystal X-ray structure for product **3c** (CCDC no. 2366084, Scheme 2, Figure S48) to define the stereochemistry of the exocyclic olefin.

**Scheme 3:**
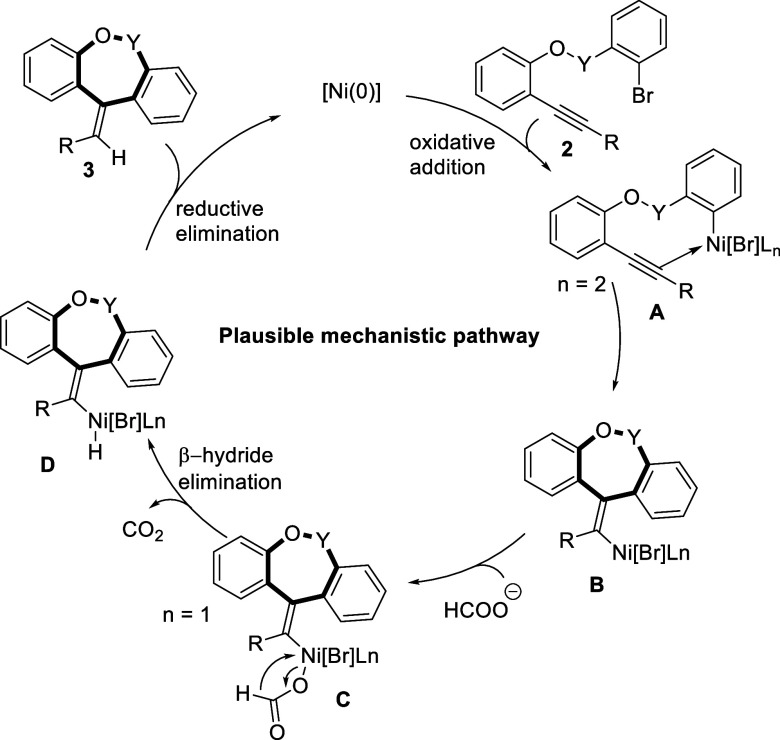
Plausible Mechanistic Approach for Tricyclic Scaffold

The regioselective synthesis of 7-*exo* cyclized products (**3a**–**v**) can be realized through a plausible mechanistic pathway depicted in Scheme 3, which further receives computational support for theoretical confirmation. This nickel (0)-catalyzed mechanism is similar to the one proposed in previous reports.^24^

Herein, at the initial stage, an aryl nickel π-complex **A** is generated from alkyne substrate **2** via oxidative addition and readily converted into a σ-vinyl nickel species **B**. Trapping of the newly generated species **B** by the formate group may then lead to complex **C**. The σ-vinyl complex **C** readily undergoes β-hydride elimination to form **D** and subsequent reductive elimination to regenerate the Ni(0) catalyst and delivers the Mizoroki-Heck-type carbocyclization product regioselectively. Syn-addition of nickel to the triple bond during the reductive-Heck reaction resulted in the regioselective formation of dibenzo[*b*,*e*]oxepine compounds **3a**–**i** possessing *Z*-configuration of the *exo*-cyclic double bond and, eventually, sultones **3k**–**s** and **3u** with *E*-configuration of the exocyclic double bond in exclusive fashion (Scheme 3). This mechanistic insight for the regio- and stereoselectivities has been confirmed by density functional theory (DFT) calculations, which are discussed further.

**Scheme 4:**
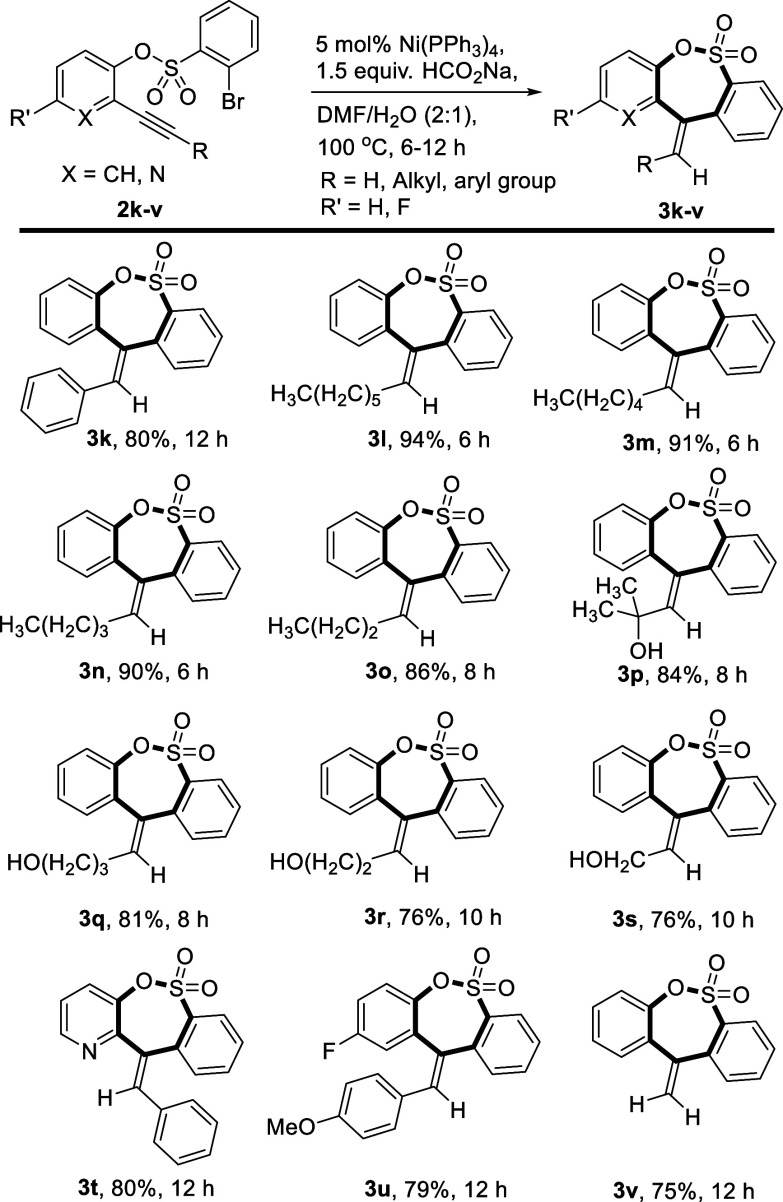
Substrate Scope to Access Sultone Derivatives

Next, we sought to assess the substrate scope toward 2-(alkyl/aryl ethynyl)phenyl 2-bromobenzenesulfonate substrates to access sultones in a regioselective fashion (Scheme 4). Satisfyingly, all substrates showed good reactivities and regioselectivities for the current transformations under the optimized set of reaction conditions.

Substrate **2k** with a phenyl substituent at the alkyne terminal tolerated the reaction conditions to furnish (*E*)-11-benzylidene-11*H*-dibenzo[*c*,*f*][1,2]oxathiepine 6,6-dioxide **3k** in 80% yield. Comparatively, substrates with linear aliphatic chains at the alkyne terminus exhibited excellent yields of the sultone product (**3l**–**o**, 86–94%). Alkynes with branched and linear hydroxyl functionals proved to be handy candidates for the protocol (**3p**–**s**, 76–84%). Meanwhile, the scope of heteroaryl alkynes was also investigated under this nickel catalysis, and it was found that they could also react smoothly to obtain corresponding sultone products regardless of their electronic character (**3t**, 80%). Substrate having an electron-withdrawing group (-F) placed at the phenol ring was also well tolerated under the optimized set of reaction conditions and furnished product **3u** in 79% yield. In addition, the substrate with terminal alkyne yielded product **3v** in 75% yield.^25^ This cemented the claim of the robustness of this nickel-catalyzed reductive-Heck strategy to access medium-ring oxacycles regioselectively in excellent yields.

**Figure 2:**
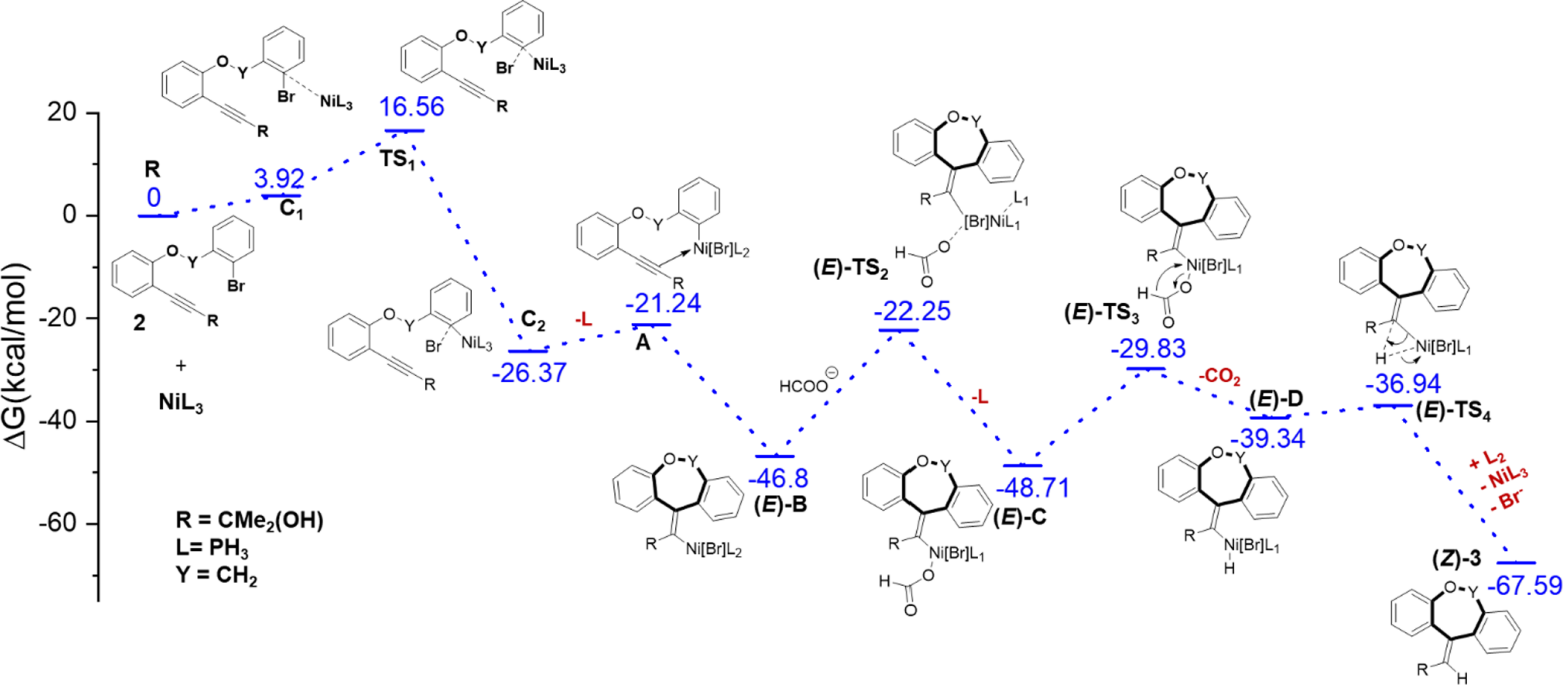
Free-energy diagram for forming the dibenzo[*b*,*e*]oxepine product.

We further validate the experimentally proposed mechanism by using the DFT method. The first step involves oxidative addition facilitated by the Ni(0) catalyst. In this mechanism, PPh_3_ ligands are substituted with PH_3_ for computational efficiency. The singlet Ni(0) has a lower free energy of 32.7 kcal/mol than the triplet Ni(0). In the suggested mechanism, activating the C–Br bond of alkyne substrate **2** involves the coordination of the singlet Ni(0) complex to the carbon atom (Figure 2). The calculated free-energy barrier of oxidative addition is 16.56 kcal/mol for the separated reactants. Ni(0) attacks the *ipso*-carbon as a nucleophile through an S_N_2-like transition state. A stable reaction complex (**C**_**2**_) forms, where Ni coordinates with the carbon atom of **2**. A square planar Ni(II) π-complex (**A**) is formed during the reaction pathway by dissociating a phosphine ligand and capturing the bromide. The formation of **A** results in a reduction in free energy of −21.24 kcal/mol compared with the isolated reactants. The optimized geometries are detailed in Figure S49, ESI. During the reaction, the regioselective formation of the 7-*exo*-cyclized product occurs through the reductive Heck reaction. Due to the *syn*-addition to the triple bond, the σ-vinyl nickel species **B** possesses the *E*-conformation of the exocyclic double bond. Endocyclization is quite unlikely due to the high strain imposed by the trans geometry around the double bond in the 8-membered ring.^24a^ The *endo*-cyclized 8-memebered ring structure is energetically less stable by 12.90 kcal/mol compared to exocyclized nickel species **B** (Figure S50, ESI). The free-energy profile indicates that the cyclization process occurs at a favorable rate, with stabilization of −25.56 kcal/mol compared to that of **A**. Then, the formate ion coordinates through a transition state (**TS2**) to generate the formate complex, **C**. Subsequently, a decarboxylation reaction occurs, producing the hydride complex, **D**. In this β-hydride elimination step, the C–H bond is broken and a Ni–H bond is formed with a reaction free energy of +9.37 kcal/mol. This suggests that the β-hydride elimination step is more challenging to occur. However, the subsequent product formation step is straightforward, with a free energy barrier of 2.4 kcal/mol. Ultimately, the dibenzo[*b*,*e*]oxepine product with a *Z*-configuration is formed through this reductive elimination process. The Ni(0) catalyst is regenerated within the reaction mixture. The oxidative coupling step controls the overall reaction, during which the oxidation state of the nickel metal changes from 0 to +2 through transition state **TS1**. The electron-donating group present in the alkyne facilitates its attack on the metal center.

**Figure 3:**
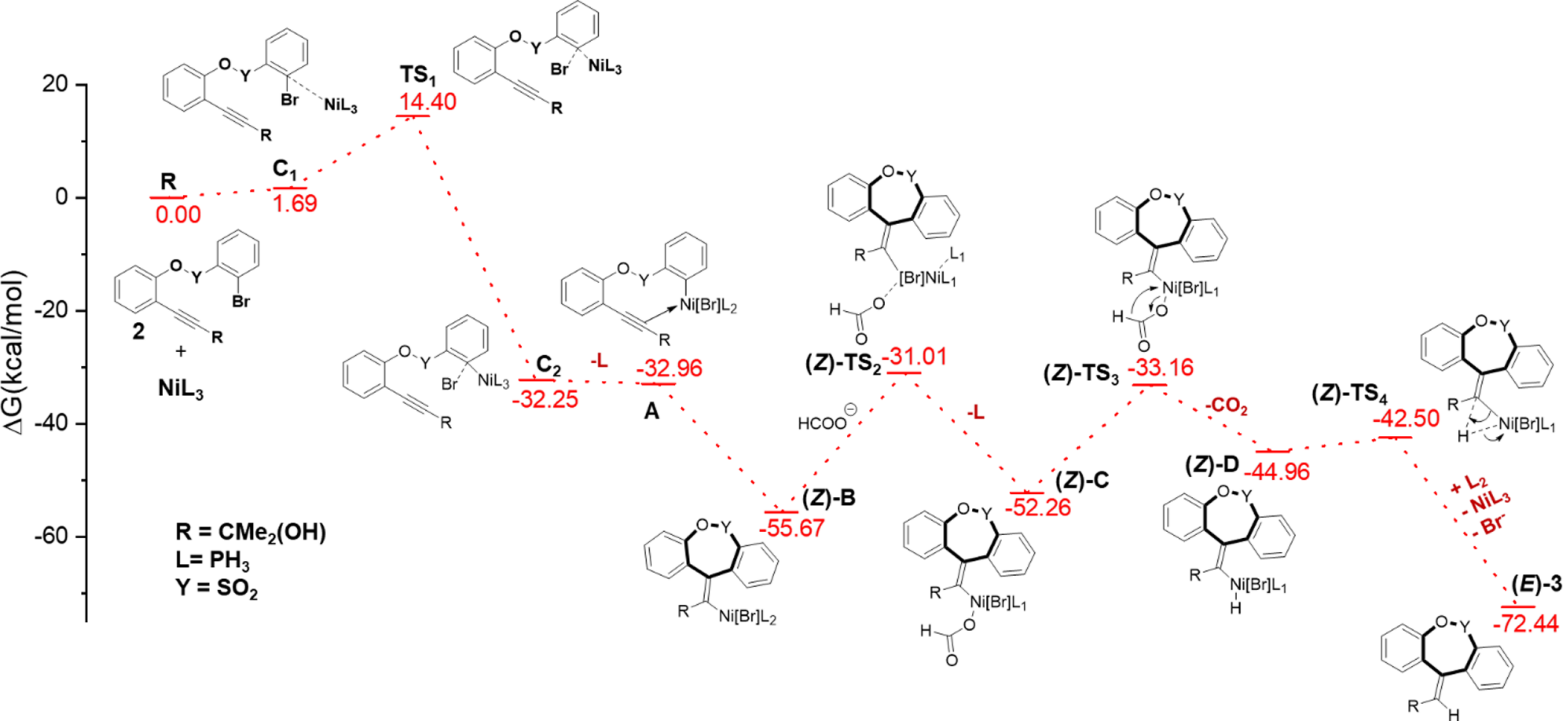
Free-energy diagram for forming the sultone product.

The formation of sultone with the *E*-configuration of the *exo*-cyclic double bond also follows the same mechanistic pathway (Figure 3). In this process, during the *syn*-addition to the triple bond, the σ-vinyl nickel species **B** adopts the *Z*-conformation. The cyclization process proceeds favorably with an exergonic change of −22.71 kcal/mol relative to species **A**. The formate complex **C** is generated with an activation barrier of 24.66 kcal/mol with respect to **B**. Then, the β-hydride elimination followed by the reductive elimination regenerates the Ni(0) catalyst and produces the sultone product. The optimized geometries of stationary points are described in Figures S51 and S52, ESI. For the benzopyrido-fused oxepine or sultone, the stereochemistry of the exocyclic double bond is inverted relative to the dibenzo-fused ring.^21^ The pyridine-fused benzoxepine derivative (**3j**) adopts the *E*-configuration, while the pyridine-fused sultone derivative (**3t**) exhibits the *Z*-configuration (Schemes 2 and 4). The stereochemical product in the reaction of the pyridine derivative is controlled by the N–Ni interaction (Figure S53, ESI). The natural bond order analysis, combined with the Wiberg bond index (WBI),^26^ provides a comprehensive overview of the bonding arrangement within a molecule. WBI quantitatively measures the level of electron sharing between two atoms in a molecule. The WBI calculations support the formation and stability of the N–Ni bond. The calculated WBI for the N–Ni bond is 0.268 for oxepine and 0.259 for the sultone. The N–Ni bond is formed when the lone pair on the pyridinic nitrogen is donated to the antibonding orbital of the nickel center.

**Scheme 5:**
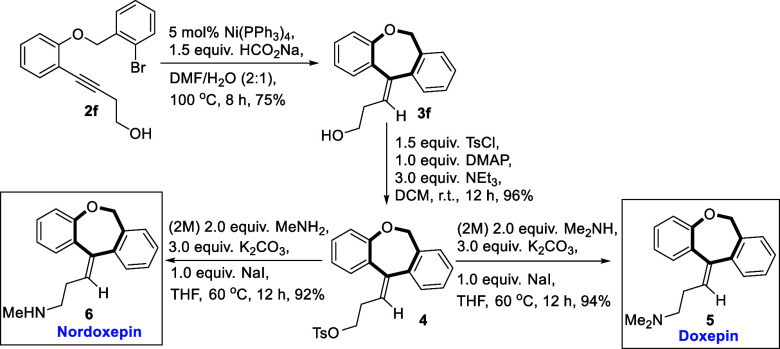
Employing Our Protocol to Access Doxepin And Nordoxepin

To demonstrate the synthetic utility of the dibenzoxepine products, concise syntheses of the active pharmaceutical ingredients doxepin and nordoxepin were chosen as prime targets. The approach began with our substrate **2f** subjected to the nickel-catalyzed reductive-Heck protocol to furnish the desired reductive-Heck product **3f** (Scheme 5). Compound **3f** was tosylated to transform the hydroxyl functionality into a leaving group that is better (**4**). Tosyl derivative **4** was finally put to react with 2(M) dimethylamine and K_2_CO_3_ in the presence of sodium iodide in THF solvent at 60 °C for 12 h to produce doxepin in 94% yield. With the same tosyl derivative **4**, we have also accessed nordoxepin which is more potent and selective as a norepinephrine reuptake inhibitor relative to doxepin.^27^

**Figure 4:**
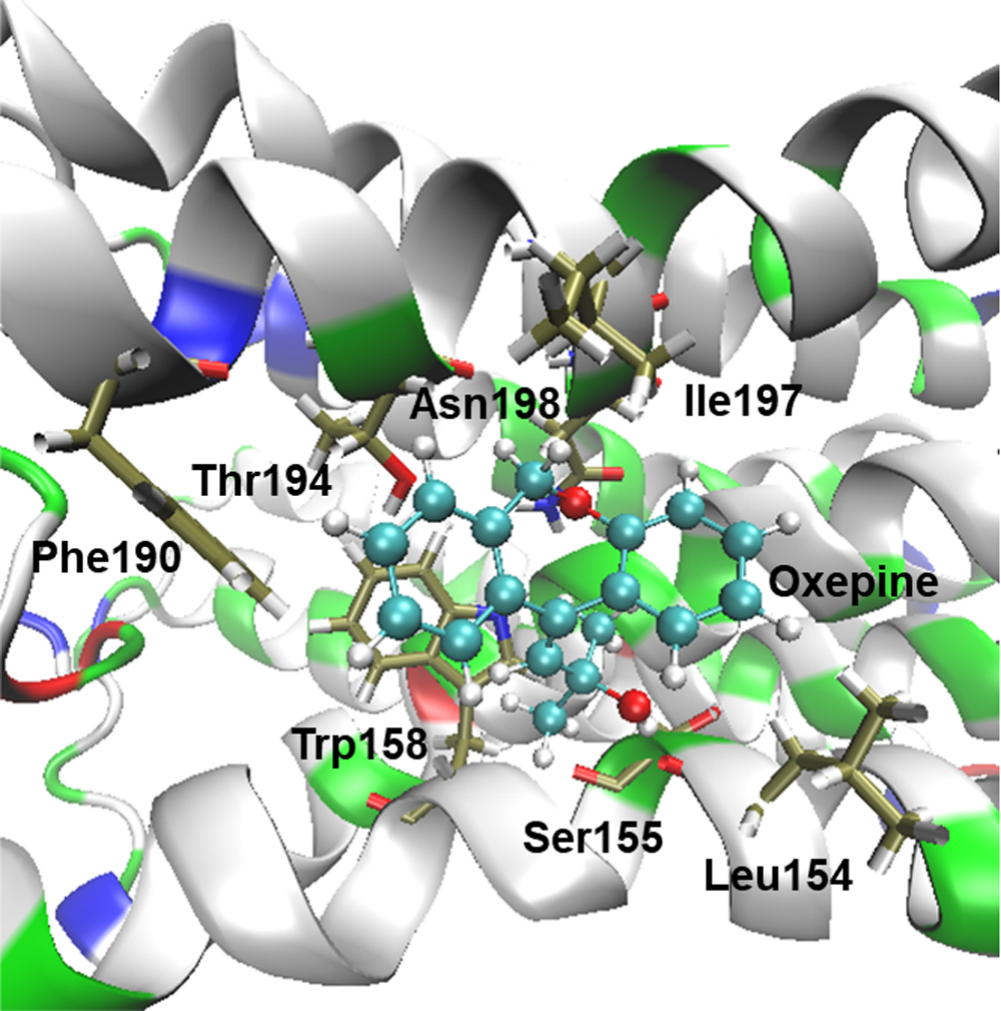
Molecular docking interactions of the receptor (PDB ID: 8 × 5Y) with dibenzo[*b*,*e*]oxepine. The binding pose of oxepine in the active site of the receptor is shown.

As mentioned, doxepin, a first-generation histamine H_1_ receptor (H_1_R) antagonist, alleviates allergic reaction symptoms.^28^ Numerous studies have been conducted to develop H_1_R antagonists. However, a significant issue with these compounds is their tendency to cause considerable side effects, including sedation, dry mouth, and arrhythmia.^28^ These side effects occur due to their ability to penetrate the blood-brain barrier and their low receptor selectivity. In this section, we explore the binding activity of oxepine at the binding pocket of the H_1_R protein site. The docking study was conducted using the H_1_R protein (8 × 5Y)^29^ which has a resolution of 3.00 Å. The docking study reveals that the tricyclic dibenzo[*b*,*e*]oxepine ring is encircled by residues Ile197, Asn198, Thr194, Phe190, and Trp158 through hydrophobic interactions (Figures 4 and S54, ESI). Additionally, Leu154 forms an O···H–O hydrogen bond with the oxepine hydroxyl group, stabilizing the ligand within the cavity. The calculated binding energy of this oxepine is–5.44 kcal/mol.

**Figure 5:**
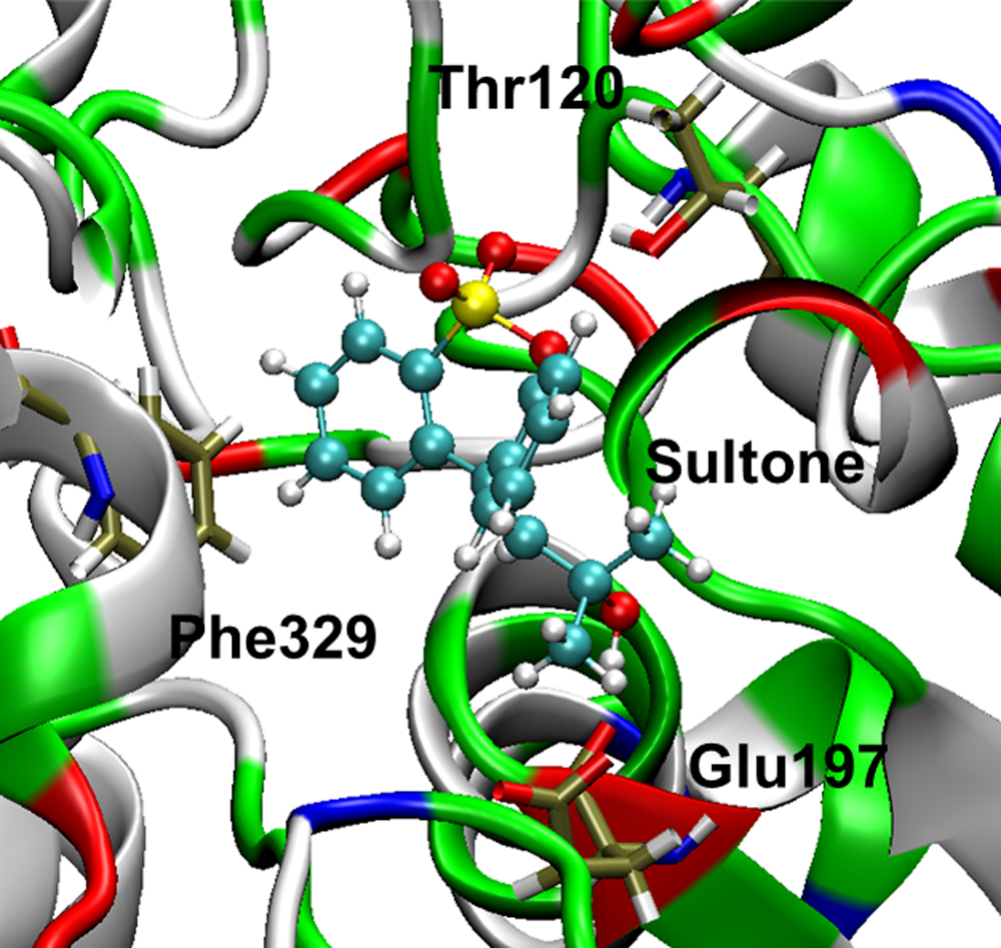
Molecular docking interactions of the receptor (PDB ID: 5LKR) with sultone. The binding pose of sultone in the active site of the receptor is shown.

We investigated the binding affinity of sultone as an inhibitor of butyrylcholinesterase (BuChE). Recently, a novel class of δ-sultone-fused pyrazole scaffolds have emerged as highly selective submicromolar BuChE inhibitors.^30^ These compounds are effective and selective BuChE inhibitors, exhibiting nontoxic, lipophilic properties and significant neuroprotective activity. Additionally, this particular scaffold displayed reversible, mixed-type BuChE inhibitory activity, which has the potential to help alleviate disease symptoms in progressive AD. In this study, we have docked the sultone in the active site of BuChE (5LKR).^31^ The docking model shows that the benzene ring of sultone forms π–π interactions with Phe329 (Figures 5 and S55, ESI). In addition, SO_2_ of the sultone ring interacts with Thr120. Furthermore, Glu197 creates a hydrogen bond (O···H–O) with the hydroxyl group, contributing to the stabilization of the ligand within the cavity. The sultone has a substantial calculated binding energy of −6.46 kcal/mol.

## Conclusions

Nickel-catalyzed regioselective access to dibenzo[*b*,*e*]oxepine and the dibenzo[*c*,*f*][1,2]oxathiepine 6,6-dioxide scaffold is described. This reductive-Heck approach involved the use of water as the solvent and furnished the desired products in high to excellent yields (74–94%). This protocol tolerated aryl and aliphatic alkynes with even hydroxyl functional. While synthesizing both the scaffolds, we found that water plays a pivotal role and can be supplemented with isopropanol if needed. In-depth mechanistic exploitation through computational studies boosted experimental observations. The practical utility of this methodology has been demonstrated via the formation of doxepin and nordoxepin molecules, frequently recommended to psychoneurotic patients with depression or anxiety.

## Experimental Section

All reagents were purchased from commercial suppliers and used without further purification unless otherwise specified. Commercially supplied ethyl acetate and petroleum ether were distilled before use. All solvents were dried using the usual methods. The petroleum ether used in our experiments has a boiling range of 60–80 °C. Analytical thin-layer chromatography was performed on 0.25 mm extra-hard silica gel plates with a UV254 fluorescent indicator. The reported melting points are uncorrected. ^1^H NMR and ^13^C NMR spectra were recorded at ambient temperature using both 300 and 400 MHz spectrometers (300 and 400 MHz for ^1^H and 75 and 100 MHz for ^13^C). Chemical shifts are reported in ppm with respect to the tetramethylsilane internal reference, and coupling constants are reported in Hz. Proton multiplicities are represented as s (singlet), d (doublet), dd (double doublet), t (triplet), q (quartet), and m (multiplet). Infrared spectra were recorded on an FT-IR spectrometer in thin films. HR-MS data were recorded using an LCQ-ORBITRAP-XL instrument.

## Data Availability

The data underlying this study are available in the published article and its Supporting Information.

## Supporting Information Available

ESI-MS spectra; spectroscopic and XRD data; NMR spectra; computational details; and additional figures (PDF)
