# Supplementary material for: Ni(0)-Catalyzed Efficient, Regioselective Synthesis of Dibenzo[b,e]oxepines and Dibenzo[c,f][1,2]oxathiepine 6,6-Dioxides: Mechanistic Study by DFT Calculation and Docking Interactions
Source: ACS Omega. 2024 Nov 4;9(46):46148–56. doi: 10.1021/acsomega.4c06569 (PMC11579748; doi:10.1021/acsomega.4c06569)
Supplement: Supplementary file 1 — ao4c06569_si_001.pdf [file ao4c06569_si_001.pdf]

## Supporting Information

# Ni(0)-Catalyzed Efficient, Regioselective Synthesis of Dibenzo[*b*,*e*]oxepines & Dibenzo[*c*,*f*][1,2]oxathiepine 6,6-dioxides: Mechanistic Study by DFT Calculation and Docking Interactions

Uma Sankar Mandal,[a‖] Sk Shamim Ahamed,[a‖] Rabindranath Lo,[b*] Debashree Manna,[b*] Tapas Ghosh[a*]

[a]Uma Sankar Mandal, Sk Shamim Ahamed, Dr. Tapas Ghosh, Department of Chemistry, Jadavpur University, Kolkata 700032, India. e-mail: tapasg.chemistry@jadavpuruniversity.in

[b]Dr. Rabindranath Lo, Dr. Debashree Manna, Institute of Organic Chemistry and Biochemistry, Czech Academy of Sciences, v.v.i., Flemingovo nám. 2, 16610 Prague 6, Czech Republic, e-mail: rabindranath.lo@uochb.cas.cz, debashree.manna@uochb.cas.cz

‖Equal contribution

## Table of contents:

1) General Information – Materials and Equipment……………………………………......S2

2) General procedure for synthesis and characterization data of starting materials **1a-f**.....S2-3

3) General procedure for synthesis and characterization data of alkyne substrates **2a-v**..S3-15

4) General procedure for synthesis and characterization data of dibenzo[*b*,*e*]oxepins **3a-j** & dibenzo[*c*,*f*][1,2]oxathiepine 6,6-dioxides **3k-v**.........................................………...........S15-26

5) General procedure for synthesis and characterization data compounds **4-6** ...............S26-27

6) $^{1}$H and $^{13}$C NMR spectra of compounds **2a-v**………………….................................S28-49

7) $^{1}$H and $^{13}$C NMR spectra of compounds **3a-v**……………………………..................S50-71

8) $^{1}$H and $^{13}$C NMR spectra of compounds **4-6**……………………….......................S72-74

9) X-ray crystal data for compound **3c**..................................................................S75-76

10) Computational analysis of reaction pathway and study of bioactivity......................S77-81

**Experimental section:**

## 1) General Information – Materials and Equipments:

Melting points were determined in open capillaries and are uncorrected. IR spectra were run for KBr discs (and neat for liquid samples) on AIM-8800 infrared microscope connected to a Shimadzu IR Affinity FT-IR spectrometer ($\upsilon_{max}$ in $cm^{-1}$) and NMR spectra were recorded on a Bruker-Daltonics Avance-300 & 400 spectrometer operating at 300 MHz, 400 MHz ($^1$H) or 75 MHz, 100 MHz ($^{13}$C), with the residual protic solvent used as the internal standard. Mass spectra were recorded using LCQ-ORBITRAP-XL instrument. Silica gel (60 - 120 mesh) and (230 - 400 mesh) were used for chromatographic separation. Petroleum-ether refers to the fraction between 60 $^o$C and 80 $^o$C.

## 2) General procedure for the preparation of compound 1a-f:

Sodium hydride (230 mg, 9.6 mmol) was washed free of mineral oil (3 hexane washings) and treated with DMF (15 mL) followed by a solution of 2-iodophenol (1.5 g, 6.4 mmol) in DMF (10 mL). After $H_2$ evolution had ceased, solid 2-bromobenzyl bromide (1.6 g, 6.4 mmol) or 1-bromo-2-(bromomethyl)-4-methoxybenzene (1.79 g, 6.4 mmol) was added and the reaction mixture was stirred at room temperature for 16 h followed by stirring at 70 $^o$C for 2 additional hours. The reaction mixture was cooled to room temperature, poured into 2N HCl (50 mL), and extracted with ether (3 x 30 mL). The combined organic parts were washed with brine (50 mL), dried over $MgSO_4$, and concentrated under reduced pressure, and purified using flash column chromatography on silica gel (elution with 11% diethyl ether in hexanes) to give benzylic ether **1a-1b** as a white crystalline solid. 2-iodophenyl 2-bromobenzenesulfonate, **1c** was also prepared using the literature procedure and obtained as white crystalline solid.[1] Substrates **1d-f** were also prepared following literature procedure.[1]

**1-bromo-2-((2-iodophenoxy)methyl)benzene (1a):**

White solid; mp: 72 - 74 $^o$C; yield = 94% (1.9 g); IR (KBr): 3073, 2931, 1444, 1175 $cm^{-1}$**;** $^1$H NMR (300 MHz, $CDCl_3$) δ 7.82 (dd, *J* = 7.8, 1.8 Hz, 1H), 7.76 - 7.80 (m, 1H), 7.58 (dd, *J* = 7.8, 1.2 Hz, 1H), 7.38 (td, *J* = 7.6, 1.2 Hz, 1H), 7.28 - 7.34 (m, 1H), 7.17 - 7.23 (m, 1H), 6.89 (dd, *J* = 8.1, 1.2 Hz, 1H), 6.76 (td, *J* = 7.6, 1.4 Hz, 1H), 5.19 (s, 2H); $^{13}$C NMR (75 MHz, $CDCl_3$) δ 156.94, 139.74, 135.93, 132.53, 129.68, 129.25, 128.82, 127.83, 123.17, 121.60, 112.72, 86.66, 70.26.

**1-bromo-2-((2-iodophenoxy)methyl)-4-methoxybenzene (1b):**

White solid; mp: 68 - 70 °C; yield = 93% (2.0 g); IR (KBr): 1582, 1566 $cm^{-1}$; $^{1}H$ NMR (500 MHz, $CDCl_3$) δ 7.84 (d, *J* = 7.8 Hz, 1H), 7.43 - 7.49 (m, 2H), 7.35 (t, *J* = 7.8 Hz, 1H), 6.93 (d, *J* = 8.0 Hz, 1H), 6.76 - 6.81 (m, 2H), 5.16 (s, 2H), 3.86 (s, 3H); $^{13}C$ NMR (100 MHz, $CDCl_3$) δ 159.44, 156.82, 139.69, 136.93, 133.07, 129.76, 123.22, 115.63, 113.79, 112.67, 111.46, 86.64, 70.01, 65.79.

**2-iodophenyl 2-bromobenzenesulfonate (1c):**

White solid; mp: 120 - 123 °C; yield = 94% (2.1 g); IR (KBr): 3077, 2934, 1444, 1180 $cm^{-1}$; $^{1}H$ NMR (300 MHz, $CDCl_3$) δ 8.05 (dd, *J* = 7.5 Hz, 2.1 Hz, 1H), 7.81 - 7.87 (m , 2H), 7.46 - 7.56 (m, 2H), 7.28 - 7.31 (m, 1H), 6.96 - 7.07 (m, 2H); $^{13}C$ NMR (75 MHz, $CDCl_3$) δ 150.28, 140.36, 136.77, 136.00, 135.16, 132.31, 129.49, 128.57, 127.71, 122.93, 121.64, 90.11.

**3-((2-bromobenzyl)oxy)-2-iodopyridine (1d):**

White solid; m.p. 87 - 89 °C; yield = 92% (1.9 g); IR (KBr): 3077, 2934, 1444, 1180 $cm^{-1}$; $^{1}H$ NMR (300 MHz, $CDCl_3$) δ 8.04 (dd, *J* = 4.6, 1.5 Hz, 1H), 7.71 - 7.74 (m, 1H), 7.59 (dd, *J* = 8.0, 1.2 Hz, 1H), 7.39 (td, *J* = 7.6, 1.2 Hz, 1H), 7.17 - 7.24 (m, 2H), 7.04 (dd, *J* = 8.2, 1.5 Hz, 1H), 5.20 (s, 2H); $^{13}C$ NMR (75 MHz, $CDCl_3$) δ 154.13, 143.29, 134.93, 132.69, 129.64, 128.82, 127.98, 123.62, 121.72, 118.62, 112.23, 70.37.

**2-iodopyridin-3-yl 2-bromobenzenesulfonate (1e):**

White solid; m.p. 141 - 143 °C; yield = 93% (2.1 g); IR (KBr): 3099, 2981, 1386, 1180 $cm^{-1}$; $^{1}H$ NMR (300 MHz, $CDCl_3$) δ 8.29 (dd, *J* = 4.6, 1.7 Hz, 1H), 8.06 - 8.10 (m, 1H), 7.84 - 7.88 (m, 1H), 7.47 - 7.58 (m, 3H), 7.26 (dd, *J* = 8.1, 4.8 Hz, 1H); $^{13}C$ NMR (75 MHz, $CDCl_3$) δ 148.82, 148.27, 136.36, 136.32, 135.73, 132.49, 130.16, 128.05, 123.65, 121.93, 114.40.

**4-fluoro-2-iodophenyl 2-bromobenzenesulfonate (1f):**

White solid; m.p. 92 - 94 °C; yield = 83% (1.6 g); IR (KBr): 3072, 2931, 1438, 1175 $cm^{-1}$; $^{1}H$ NMR (400 MHz, $CDCl_3$) δ 8.05 (dd, *J* = 7.6, 2.0 Hz, 1H), 7.86 (dd, *J* = 7.7, 1.6 Hz, 1H), 7.47 -7.56 (m, 3H), 6.98 - 7.07 (m, 2H); $^{13}C$ NMR (75 MHz, $CDCl_3$) δ 162.11, 158.77, 146.79, 146.75, 136.57, 136.19, 135.44, 132.51, 127.91, 127.28, 126.94, 123.87, 123.75, 121.81, 116.61, 116.30, 90.36, 90.25.

### 3) General procedure for the preparation of staring materials 2a-v:

A mixture of compound **1a** (500 mg, 1.28 mmol), phenylacetylene (131 mg, 1.28 mmol), $Pd(PPh_3)_2Cl_2$ (45 mg, 5 mol%), CuI (7 mg, 3 mol%) and dry $Et_3N$ (2 ml) in dry DMF (5 ml) was stirred at room temperature for 10 h. After completion of the reaction as monitored by TLC, the reaction mixture was cooled and water (5 mL) was added and then extracted with

EtOAc (3 x 15 mL). The organic extract was washed with water (2 x 10 mL) followed by brine (10 mL) and subsequently the organic layer was dried over $MgSO_4$. Further concentration furnished a crude mass which was purified by column chromatography over silica-gel. Elution of the column with petroleum ether-ethyl acetate (19:1) mixture afforded the product **2a**. Similarly other alkynes were treated with compounds **1a-f** to produce the corresponding substrates **2b-v**.

**1-bromo-2-((2-(phenylethynyl)benzyl)oxy)methyl)benzene (2a):**

Yellow solid; m.p. 66 - 68 °C; yield = 84% (392 mg); IR (KBr): 2986, 2930, 2228, 1598 $cm^{-1}$; $^1H$ NMR (400 MHz, $CDCl_3$) δ 7.78 - 7.86 (m, 1H), 7.53 - 7.59 (m, 4H), 7.29 - 7.39 (m, 5H), 7.19 (td, *J* = 7.7, 1.7 Hz, 1H), 6.97 - 7.01 (m, 2H), 5.25 (s, 2H); $^{13}C$ NMR (100 MHz, $CDCl_3$) δ 159.00, 136.52, 133.47, 132.48, 131.69, 129.88, 129.11, 128.57, 128.47, 128.28, 127.67, 123.81, 121.63, 121.30, 113.47, 112.79, 93.95, 85.97, 69.80; LC-MS ($[M+H]^+$): for $C_{21}H_{15}BrO$ calcd. 363.0; found 363.1.

Compounds **2b-v** were obtained using the general procedure used for the synthesis of substrate **2a**.

A mixture of compound **1a** (500 mg, 1.28 mmol), 1-ethynyl-4-methoxybenzene (169 mg, 1.28 mmol), $Pd(PPh_3)_2Cl_2$ (45 mg, 5 mol%), CuI (7 mg, 3 mol%) and dry $Et_3N$ (2 ml) in dry DMF (5 ml) was stirred at room temperature for 12 h. After completion of the reaction as monitored by TLC, the reaction mixture was cooled and water (5 mL) was added and then extracted with EtOAc (3 x 15 mL). The organic extract was washed with water (2 x 10 mL) followed by brine (10 mL) and subsequently the organic layer was dried over $MgSO_4$. Further concentration furnished a crude mass which was purified by column chromatography over silica-gel. Elution of the column with petroleum ether-ethyl acetate (19:1) mixture afforded the product **2b**.

**1-bromo-2-((2-((4-methoxyphenyl)ethynyl)phenoxy)methyl)benzene (2b):**

White solid; m.p. 84 - 86 °C; yield = 85% (430 mg); IR (KBr): 2987, 2930, 2227, 1597 $cm^{-1}$; $^1H$ NMR (400 MHz, $CDCl_3$) δ 7.86 (dd, *J* = 7.6 , 0.8 Hz, 1H), 7.58 (dd, *J* = 8.0 Hz, 1.2 Hz, 1H), 7.49 - 7.54 (m, 3H), 7.34 (td, *J* = 7.6 Hz, 1.2 Hz, 1H), 7.29 (td, *J* = 8.0 Hz, 1.7 Hz, 1H), 7.19 (td, *J* = 7.7 Hz, 1.7 Hz, 1H), 6.98 (t, *J* = 8.0 Hz, 2H), 6.90 (d, *J* = 8.8 Hz, 2H), 5.24 (s, 2H), 3.84 (s, 3H); $^{13}C$ NMR (100 MHz, $CDCl_3$) δ 159.70, 158.85, 136.59, 133.26, 133.11, 132.46, 129.49, 129.07, 128.59, 127.64, 121.62, 121.29, 115.96, 114.13, 113.83, 112.82, 93.96, 84.60, 69.81, 55.43; LC-MS ($[M+H]^+$): for $C_{22}H_{17}BrO_2$ calcd. 393.0; found 393.2.

A mixture of compound **1a** (500 mg, 1.28 mmol), 1-ethynyl-4-methylbenzene (148 mg, 1.28 mmol), $Pd(PPh_3)_2Cl_2$ (45 mg, 5 mol%), CuI (7 mg, 3 mol%) and dry $Et_3N$ (2 ml) in dry DMF (5 ml) was stirred at room temperature for 12 h. After completion of the reaction as monitored by TLC, the reaction mixture was cooled and water (5 mL) was added and then extracted with EtOAc (3 x 15 mL). The organic extract was washed with water (2 x 10 mL) followed by brine (10 mL) and subsequently the organic layer was dried over $MgSO_4$. Further concentration furnished a crude mass which was purified by column chromatography over silica-gel. Elution of the column with petroleum ether-ethyl acetate (19:1) mixture afforded the product **2c**.

**1-bromo-2-((2-(*p*-tolylethynyl)phenoxy)methyl)benzene (2c):**

Yellow solid; m.p. 74 - 76 $^oC$; yield = 82% (398 mg); IR (KBr): 2980, 2926, 2225, 1592 $cm^{-1}$; $^1H$ NMR (400 MHz, $CDCl_3$) δ 7.86 (dd, *J* = 7.8 Hz, 1.7 Hz, 1H), 7.58 (dd, *J* = 8.0 Hz, 1.3 Hz, 1H), 7.53 (dd, *J* = 7.8 Hz, 1.7 Hz, 1H), 7.45 (d, *J* = 8.0 Hz, 2H), 7.34 (td, *J* = 7.6 Hz, 1.2 Hz, 1H), 7.27 - 7.32 (m, 1H), 7.14 - 7.21 (m, 3H), 6.94 - 7.02 (m, 2H), 5.24 (s, 2H), 2.38 (s, 3H); $^{13}C$ NMR (100 MHz, $CDCl_3$) δ 158.95, 138.40, 136.59, 133.39, 132.47, 131.59, 129.67, 129.24, 129.09, 128.60, 127.68, 121.62, 121.30, 120.75, 113.72, 112.83, 94.16, 85.27, 69.82, 21.66; LC-MS ($[M+H]^+$): for $C_{22}H_{17}BrO$ calcd. 377.0; found 377.2.

A mixture of compound **1a** (500 mg, 1.28 mmol), hept-1-yne (123 mg, 1.28 mmol), $Pd(PPh_3)_2Cl_2$ (45 mg, 5 mol%), CuI (7 mg, 3 mol%) and dry $Et_3N$ (2 ml) in dry DMF (5 ml) was stirred at room temperature for 11 h. After completion of the reaction as monitored by TLC, the reaction mixture was cooled and water (5 mL) was added and then extracted with EtOAc (3 x 15 mL). The organic extract was washed with water (2 x 10 mL) followed by brine (10 mL) and subsequently the organic layer was dried over $MgSO_4$. Further concentration furnished a crude mass which was purified by column chromatography over silica-gel. Elution of the column with petroleum ether-ethyl acetate (19:1) mixture afforded the product **2d**.

**1-bromo-2-((2-(hept-1-yn-1-yl)phenoxy)methyl)benzene (2d):**

Brown gummy; yield = 90% (413 mg); IR (KBr): 2988, 2933, 2230, 1599 $cm^{-1}$; $^1H$ NMR (400 MHz, $CDCl_3$) δ 7.75 (dd, *J* = 7.7 Hz, 1.9 Hz, 1H), 7.37 (dd, *J* = 7.9, 1.2 Hz, 1H), 7.41 (dd, *J* = 7.7 Hz, 1.7 Hz, 1H), 7.34 (td, *J* = 7.6 Hz, 1.2 Hz, 2H), 7.22 (td, *J* = 8.1 Hz, 1.9 Hz, 1H), 7.18 (td, *J* = 7.7 Hz, 1.6 Hz, 2H), 6.92 (t, *J* = 7.7 Hz, 3H), 5.20 (s, 2H), 2.48 (t, *J* = 7.1 Hz, 2H), 1.65 (p, *J* = 7.1 Hz, 2H), 1.43 - 1.50 (m, 2H), 1.31 - 1.40 (m, 3H), 0.91 (t, *J* = 7.2 Hz, 4H); $^{13}C$ NMR (100 MHz, $CDCl_3$) δ 158.93, 136.64, 133.62, 132.46, 129.04, 128.94,

128.59, 127.59, 121.67, 121.21, 114.26, 112.86, 95.13, 69.84, 31.29, 28.74, 22.42, 19.89, 14.14; LC-MS ($[M+Na]^+$): for $C_{21}H_{23}BrO$ calcd. 393.1; found 393.2.

A mixture of compound **1a** (500 mg, 1.28 mmol), hex-1-yne (105 mg, 1.28 mmol), $Pd(PPh_3)_2Cl_2$ (45 mg, 5 mol%), CuI (7 mg, 3 mol%) and dry $Et_3N$ (2 ml) in dry DMF (5 ml) was stirred at room temperature for 12 h. After completion of the reaction as monitored by TLC, the reaction mixture was cooled and water (5 mL) was added and then extracted with EtOAc (3 x 15 mL). The organic extract was washed with water (2 x 10 mL) followed by brine (10 mL) and subsequently the organic layer was dried over $MgSO_4$. Further concentration furnished a crude mass which was purified by column chromatography over silica-gel. Elution of the column with petroleum ether-ethyl acetate (19:1) mixture afforded the product **2e**.

**1-bromo-2-((2-(hex-1-yn-1-yl)phenoxy)methyl)benzene (2e):**

Brown gummy; yield = 88% (388 mg); IR (KBr): 2978, 2921, 2220, 1578 $cm^{-1}$; $^1H$ NMR (300 MHz, $CDCl_3$) δ 7.75 (dd, *J* = 7.8 Hz, 1.9 Hz, 1H), 7.57 (dd, *J* = 7.9 Hz, 1.2 Hz, 1H), 7.41 (dd, *J* = 7.8 Hz, 1.6 Hz, 1H), 7.34 (td, *J* = 7.6, 1.2 Hz, 1H), 7.15 - 7.23 (m, 2H), 6.89 - 6.94 (m, 2H), 5.19 (s, 2H), 2.49 (t, *J* = 6.9 Hz, 2H), 1.68 - 1.46 (m, 4H), 0.94 (t, *J* = 7.2 Hz, 3H); $^1H$ NMR (300 MHz, $CDCl_3$) δ 7.85 - 7.88 (m, 1H), 7.52 - 7.55 (m, 1H), 7.39 - 7.47 (m, 4H), 7.27 - 7.32 (m, 1H), 7.23 (t, *J* = 1.3 Hz, 1H), 6.14 (t, *J* = 7.6 Hz, 1H), 2.13 - 2.22 (m, 4H), 1.46 (p, *J* = 7.5 Hz, 2H), 1.24 - 1.29 (m, 4H), 0.84 - 0.90 (m, 3H); $^{13}C$ NMR (75 MHz, $CDCl_3$) δ 146.94, 139.24, 138.86, 136.01, 134.92, 133.24, 132.16, 130.35, 129.75, 128.53, 127.72, 127.16, 122.87, 31.31, 29.66, 29.07, 22.44, 13.99; LC-MS ($[M+H]^+$): for $C_{20}H_{21}BrO$ calcd. 357.1; found 357.2.

A mixture of compound **1a** (500 mg, 1.28 mmol), but-3-yn-1-ol (90 mg, 1.28 mmol), $Pd(PPh_3)_2Cl_2$ (45 mg, 5 mol%), CuI (7 mg, 3 mol%) and dry $Et_3N$ (2 ml) in dry DMF (5 ml) was stirred at room temperature for 10 h. After completion of the reaction as monitored by TLC, the reaction mixture was cooled and water (5 mL) was added and then extracted with EtOAc (3 x 15 mL). The organic extract was washed with water (2 x 10 mL) followed by brine (10 mL) and subsequently the organic layer was dried over $MgSO_4$. Further concentration furnished a crude mass which was purified by column chromatography over silica-gel. Elution of the column with petroleum ether-ethyl acetate (9:1) mixture afforded the product **2f**.

**4-(2-((2-bromobenzyl)oxy)phenyl)but-3-yn-1-ol (2f):**

Brown gummy; yield = 90% (383 mg); IR (KBr): 2230, 1585 $cm^{-1}$; $^{1}H$ NMR (300 MHz, $CDCl_3$) δ 7.67 (dd, *J* = 7.7 Hz, 1.7 Hz, 1H), 7.58 (dd, *J* = 8.0 Hz, 1.3 Hz, 1H), 7.41 (dd, *J* = 7.5 Hz, 1.8 Hz, 1H), 7.35 (td, *J* = 7.6 Hz, 1.3 Hz, 1H), 7.25 (td, *J* = 8.0 Hz, 1.8 Hz, 1H), 7.19 (td, *J* = 7.8 Hz, 1.8 Hz, 1H), 6.92 (td, *J* = 8.3 Hz, 1.4 Hz, 2H), 5.20 (s, 2H), 3.81 (q, *J* = 5.9 Hz, 2H), 2.75 (t, *J* = 6.1 Hz, 2H), 2.02 (t, *J* = 6.6 Hz, 1H); $^{13}C$ NMR (75 MHz, $CDCl_3$) δ 159.09, 136.31, 133.47, 132.65, 129.47, 129.32, 128.71, 127.73, 121.95, 121.22, 113.38, 112.66, 91.03, 79.03, 69.95, 61.28, 24.36; LC-MS ($[M+H]^+$): for $C_{19}H_{19}BrO$ calcd. 343.1; found 343.1.

A mixture of compound **1b** (500 mg, 1.19 mmol), oct-1-yne (131 mg, 1.19 mmol), $Pd(PPh_3)_2Cl_2$ (42 mg, 5 mol%), CuI (7 mg, 3 mol%) and dry $Et_3N$ (2 ml) in dry DMF (5 ml) was stirred at room temperature for 12 h. After completion of the reaction as monitored by TLC, the reaction mixture was cooled and water (5 mL) was added and then extracted with EtOAc (3 x 15 mL). The organic extract was washed with water (2 x 10 mL) followed by brine (10 mL) and subsequently the organic layer was dried over $MgSO_4$. Further concentration furnished a crude mass which was purified by column chromatography over silica-gel. Elution of the column with petroleum ether-ethyl acetate (9:1) mixture afforded the product **2g**.

**1-bromo-4-methoxy-2-((2-(oct-1 -yn-1-yl)phenoxy)methyl)benzene (2g):**

Brown gummy; yield = 85% (407 mg); IR (KBr): 2982, 2920, 2224, 1575 $cm^{-1}$; $^{1}H$ NMR (300 MHz, $CDCl_3$), 7.39 - 7.45 (m, 2H), 7.34 (d, *J* = 3.1 Hz, 1H), 7.19 - 7.25 (m, 1H), 6.89 - 6.94 (m, 2H), 6.73 (dd, *J* = 8.7 Hz, 3.1 Hz, 1H), 5.15 (s, 2H), 3.79 (s, 3H), 2.46 (t, *J* = 7.1 Hz, 2H), 1.61 - 1.69 (m, 2H), 1.41 - 1.50 (m, 2H), 1.27 - 1.33 (m, 4H), 0.86 - 0.91 (m, 3H); $^{13}C$ NMR (75 MHz, $CDCl_3$) δ 159.39, 158.81, 137.62, 133.70, 133.00, 128.98, 121.25, 114.81, 114.24, 114.16, 112.89, 111.71, 95.10, 69.74, 55.54, 31.53, 29.02, 28.83, 22.70, 19.93, 14.22; LC-MS ($[M+H]^+$): for $C_{22}H_{25}BrO_2$ calcd. 401.1; found 401.3.

A mixture of compound **1b** (500 mg, 1.19 mmol), 2-methylbut-3-yn-2-ol (100 mg, 1.19 mmol), $Pd(PPh_3)_2Cl_2$ (42 mg, 5 mol%), CuI (7 mg, 3 mol%) and dry $Et_3N$ (2 ml) in dry DMF (5 ml) was stirred at room temperature for 12 h. After completion of the reaction as monitored by TLC, the reaction mixture was cooled and water (5 mL) was added and then extracted with EtOAc (3 x 15 mL). The organic extract was washed with water (2 x 10 mL) followed by brine (10 mL) and subsequently the organic layer was dried over $MgSO_4$. Further concentration furnished a crude mass which was purified by column chromatography

over silica-gel. Elution of the column with petroleum ether-ethyl acetate (9:1) mixture afforded the product **2h**.

**4-(2-((2-bromo-5-methoxybenzyl)oxy)phenyl)-2-methylbut-3-yn-2-ol (2h):**

Brown gummy; yield = 89% (398 mg); IR (KBr): 2230, 1585 $cm^{-1}$; $^{1}H$ NMR (300 MHz, $CDCl_3$) δ 7.40 - 7.48 (m, 3H), 7.25 - 7.31 (m, 1H), 6.91 - 6.96 (m, 2H), 6.73 (dd, *J* = 8.7 Hz, 3.1 Hz, 1H), 5.12 (s, 2H), 3.81 (s, 3H), 1.86 (s, 1H), 1.65 (s, 6H); $^{13}C$ NMR (75 MHz, $CDCl_3$) δ 159.30, 158.99, 137.63, 133.51, 133.00, 129.83, 121.26, 115.40, 113.67, 113.02, 112.86, 111.70, 98.58, 78.50, 69.67, 65.85, 55.74, 31.63; LC-MS ($[M+Na]^+$): for $C_{19}H_{19}BrO_3$ calcd. 397.0; found 397.2.

A mixture of compound **1b** (500 mg, 1.19 mmol), pent-1-yne (81 mg, 1.19 mmol), $Pd(PPh_3)_2Cl_2$ (42 mg, 5 mol%), CuI (7 mg, 3 mol%) and dry $Et_3N$ (2 ml) in dry DMF (5 ml) was stirred at room temperature for 11 h. After completion of the reaction as monitored by TLC, the reaction mixture was cooled and water (5 mL) was added and then extracted with EtOAc (3 x 15 mL). The organic extract was washed with water (2 x 10 mL) followed by brine (10 mL) and subsequently the organic layer was dried over $MgSO_4$. Further concentration furnished a crude mass which was purified by column chromatography over silica-gel. Elution of the column with petroleum ether-ethyl acetate (9:1) mixture afforded the product **2i**.

**1-bromo-4-methoxy-2-((2-(pent-1-yn-1-yl)phenoxy)methyl)benzene (2i):**

Brown gummy; yield = 88% (377 mg); IR (KBr): 2987, 2929, 2226, 1595 $cm^{-1}$; $^{1}H$ NMR (400 MHz, $CDCl_3$) δ 7.40 - 7.44 (m, 2H), 7.35 (d, *J* = 3.2 Hz, 1H), 7.21 - 7.25 (m,1H), 6.90 - 6.94 (m, 2H), 6.73 (dd, *J* = 8.4 Hz, 3.2 Hz, 1H), 5.15 (s, 2H), 3.79 (s, 3H), 2.46 (t, *J* = 7.0 Hz, 2H), 1.67 (h, *J* = 7.2 Hz, 2H), 1.05 (t, *J* = 7.2 Hz, 3H); $^{13}C$ NMR (100 MHz, $CDCl_3$) δ 159.37, 158.79, 137.58, 133.69, 132.99, 128.99, 121.21, 114.87, 114.81, 114.16, 112.77, 111.72, 94.86, 69.68, 55.57, 55.53, 22.47, 21.88, 13.70; LC-MS ($[M+H]^+$): for $C_{19}H_{19}BrO_2$ calcd. 359.1; found 359.2.

A mixture of compound **1d** (500 mg, 1.28 mmol), 2-methylbut-3-yn-2-ol (108 mg, 1.28 mmol), $Pd(PPh_3)_2Cl_2$ (45 mg, 5 mol%), CuI (7 mg, 3 mol%) and dry $Et_3N$ (2 ml) in dry DMF (5 ml) was stirred at room temperature for 12 h. After completion of the reaction as monitored by TLC, the reaction mixture was cooled and water (5 mL) was added and then extracted with EtOAc (3 x 15 mL). The organic extract was washed with water (2 x 10 mL) followed by brine (10 mL) and subsequently the organic layer was dried over $MgSO_4$. Further concentration furnished a crude mass which was purified by column chromatography

over silica-gel. Elution of the column with petroleum ether-ethyl acetate (9:1) mixture afforded the product **2j**.

**4-(3-((2-bromobenzyl)oxy)pyridin-2-yl)-2-methylbut-3-yn-2-ol (2j):**

Brown gummy; yield = 82% (364 mg); IR (KBr): 2230, 1585 $cm^{-1}$; $^{1}H$ NMR (300 MHz, $CDCl_3$) δ 8.19 - 8.21 (m, 1H), 7.72 (dd, *J* = 7.5, 1.6 Hz, 1H), 7.57 (d, *J* = 7.8 Hz, 1H), 7.32 - 7.38 (m, 1H), 7.17 - 7.23 (m, 3H), 5.17 (s, 2H), 2.82 (s, 1H), 1.95 (s, 1H), 1.66 (s, 6H); $^{13}C$ NMR (75 MHz, $CDCl_3$) δ 155.79, 142.36, 135.51, 133.87, 132.62, 129.51, 128.70, 128.55, 127.74, 123.81, 121.69, 119.80, 99.16, 69.83, 65.60, 31.36; LC-MS ($[M+H]^+$): for $C_{17}H_{16}BrNO_2$ calcd. 346.0; found 346.1.

A mixture of compound **1c** (500 mg, 1.14 mmol), phenylacetylene (116 mg, 1.14 mmol), $Pd(PPh_3)_2Cl_2$ (40 mg, 5 mol%), CuI (6 mg, 3 mol%) and dry $Et_3N$ (2 ml) in dry DMF (5 ml) was stirred at room temperature for 10 h. After completion of the reaction as monitored by TLC, the reaction mixture was cooled and water (5 mL) was added and then extracted with EtOAc (3 x 15 mL). The organic extract was washed with water (2 x 10 mL) followed by brine (10 mL) and subsequently the organic layer was dried over $MgSO_4$. Further concentration furnished a crude mass which was purified by column chromatography over silica-gel. Elution of the column with petroleum ether-ethyl acetate (9:1) mixture afforded the product **2k**.

**2-(phenylethynyl)phenyl 2-bromobenzenesulfonate (2k):**

Yellow gummy; yield = 94% (442 mg); IR (KBr): 3069, 2212, 1374, 1181 $cm^{-1}$; $^{1}H$ NMR (300 MHz, $CDCl_3$) δ 8.00 - 8.06 (m, 1H), 7.65 - 7.71 (m, 1H), 7.52 - 7.55 (m, 1H), 7.42 - 7.45 (m, 2H), 7.31 - 7.38 (m, 5H), 7.25 - 7.29 (m, 2H), 7.08 - 7.14 (m, 1H); $^{13}C$ NMR (75 MHz, $CDCl_3$) δ 149.84, 136.79, 135.95, 134.85, 133.81, 132.25, 131.93, 129.46, 128.78, 128.31, 127.64, 127.20, 122.92, 122.85, 121.52, 118.57, 94.79, 83.90; LC-MS ($[M+H]^+$): for $C_{20}H_{13}BrO_3S$ calcd. 412.9; found 413.3.

A mixture of compound **1c** (500 mg, 1.14 mmol), oct-1-yne (116 mg, 1.14 mmol), $Pd(PPh_3)_2Cl_2$ (40 mg, 5 mol%), CuI (6 mg, 3 mol%) and dry $Et_3N$ (2 ml) in dry DMF (5 ml) was stirred at room temperature for 12 h. After completion of the reaction as monitored by TLC, the reaction mixture was cooled and water (5 mL) was added and then extracted with EtOAc (3 x 15 mL). The organic extract was washed with water (2 x 10 mL) followed by brine (10 mL) and subsequently the organic layer was dried over $MgSO_4$. Further concentration furnished a crude mass which was purified by column chromatography over

silica-gel. Elution of the column with petroleum ether-ethyl acetate (9:1) mixture afforded the product **2l**.

**2-(oct-1-yn-1-yl)phenyl 2-bromobenzenesulfonate (2l):**

Brown gummy; yield = 90% (432 mg); IR (KBr): 2927, 1469, 1367, 1184 $cm^{-1}$; $^{1}H$ NMR (300 MHz, $CDCl_3$) δ 7.99 (dd, *J* = 7.8 Hz, 2.1 Hz, 1H), 7.83 (dd, *J* = 7.8 Hz, 1.5 Hz, 1H), 7.37 - 7.51 (m, 3H), 7.16 -7.20 (m, 2H), 7.01 - 7.04 (m, 1H), 2.27 (t, *J* = 7.2 Hz, 2H), 1.47 - 1.54 (m, 2H), 1.24 - 1.42 (m, 6H), 0.90 (t, *J* = 7.2 Hz, 3H); $^{13}C$ NMR (75 MHz, $CDCl_3$) δ 149.89, 136.95, 135.84, 134.85, 134.02, 132.40, 128.59, 127.60, 127.05, 122.74, 121.71, 119.24, 96.58, 74.99, 31.51, 28.76, 28.59, 22.68, 19.84, 14.22; LC-MS ($[M+H]^+$): for $C_{20}H_{21}BrO_3S$ calcd. 421.0; found 421.2.

A mixture of compound **1c** (500 mg, 1.14 mmol), hept-1-yne (110 mg, 1.14 mmol), $Pd(PPh_3)_2Cl_2$ (40 mg, 5 mol%), CuI (6 mg, 3 mol%) and dry $Et_3N$ (2 ml) in dry DMF (5 ml) was stirred at room temperature for 11 h. After completion of the reaction as monitored by TLC, the reaction mixture was cooled and water (5 mL) was added and then extracted with EtOAc (3 x 15 mL). The organic extract was washed with water (2 x 10 mL) followed by brine (10 mL) and subsequently the organic layer was dried over $MgSO_4$. Further concentration furnished a crude mass which was purified by column chromatography over silica-gel. Elution of the column with petroleum ether-ethyl acetate (9:1) mixture afforded the product **2m**.

**2-(hept-1-yn-1-yl)phenyl 2-bromobenzenesulfonate (2m):**

Brown gummy; yield = 88% (408 mg); IR (KBr): 2922, 1463, 1361, 1183 $cm^{-1}$; $^{1}H$ NMR (400 MHz, $CDCl_3$) δ 8.00 (dd, *J* = 7.8 Hz, 2 Hz, 1H), 7.83 (dd, *J* = 7.8 Hz, 1.6 Hz, 1H), 7.37 - 7.51 (m, 2H), 7.15 - 7.21 (m, 2H), 7.00 - 7.05 (m, 1H), 2.27 (t, *J* = 7.2 Hz, 2H), 1.03 (p, *J* = 7.6 Hz, 7.2 Hz, 2H), 1.21 - 1.41 (m, 4H), 0.91 (t, *J* = 7.2 Hz, 3H); $^{13}C$ NMR (100 MHz, $CDCl_3$) δ 149.89, 136.95, 135.84, 134.85, 134.02, 132.39, 128.59, 127.60, 127.05, 122.72, 121.70, 119.24, 96.57, 77.48, 77.16, 76.84, 74.99, 31.24, 28.31, 22.36, 19.80, 14.12; LC-MS ($[M+H]^+$): for $C_{19}H_{19}BrO_3S$ calcd. 407.0; found 407.2.

A mixture of compound **1c** (500 mg, 1.14 mmol), hex-1-yne (94 mg, 1.14 mmol), $Pd(PPh_3)_2Cl_2$ (40 mg, 5 mol%), CuI (6 mg, 3 mol%) and dry $Et_3N$ (2 ml) in dry DMF (5 ml) was stirred at room temperature for 11 h. After completion of the reaction as monitored by TLC, the reaction mixture was cooled and water (5 mL) was added and then extracted with EtOAc (3 x 15 mL). The organic extract was washed with water (2 x 10 mL) followed by brine (10 mL) and subsequently the organic layer was dried over $MgSO_4$. Further

concentration furnished a crude mass which was purified by column chromatography over silica-gel. Elution of the column with petroleum ether-ethyl acetate (9:1) mixture afforded the product **2n**.

**2-(hex-1-yn-1-yl)phenyl 2-bromobenzenesulfonate (2n):**

Light brown gummy; yield = 88% (394 mg); IR (KBr): 2917, 1461, 1359, 1178 $cm^{-1}$; $^{1}H$ NMR (400 MHz, $CDCl_3$) δ 8.00 (dd, *J* = 7.6 Hz, 2.0 Hz, 1H), 7.83 (dd, *J* = 7.8 Hz, 1.6 Hz, 1H), 7.38 - 7.50 (m, 3H), 7.15 - 7.22 (m, 2H), 7.02 - 7.04 (m, 1H), 2.29 (t, *J* = 7.0 Hz, 2H), 1.39 - 1.53 (m, 4H), 0.92 (t, *J* = 7.2 Hz, 3H); $^{13}C$ NMR (100 MHz, $CDCl_3$) δ 149.90, 136.95, 135.85, 134.85, 134.02, 132.40, 128.59, 127.61, 127.05, 122.71, 121.70, 119.24, 96.51, 75.00, 30.66, 22.15, 19.52, 13.77; LC-MS ($[M+H]^+$): for $C_{18}H_{17}BrO_3S$ calcd. 393.0; found 393.2.

A mixture of compound **1c** (500 mg, 1.14 mmol), pent-1-yne (78 mg, 1.14 mmol), $Pd(PPh_3)_2Cl_2$ (40 mg, 5 mol%), CuI (6 mg, 3 mol%) and dry $Et_3N$ (2 ml) in dry DMF (5 ml) was stirred at room temperature for 10 h. After completion of the reaction as monitored by TLC, the reaction mixture was cooled and water (5 mL) was added and then extracted with EtOAc (3 x 15 mL). The organic extract was washed with water (2 x 10 mL) followed by brine (10 mL) and subsequently the organic layer was dried over $MgSO_4$. Further concentration furnished a crude mass which was purified by column chromatography over silica-gel. Elution of the column with petroleum ether-ethyl acetate (9:1) mixture afforded the product **2o**.

**2-(pent-1-yn-1-yl)phenyl 2-bromobenzenesulfonate (2o):**

Brown gummy; yield = 85% (367 mg); IR (KBr): 2915, 1459, 1361, 1179 $cm^{-1}$; $^{1}H$ NMR (400 MHz, $CDCl_3$) δ 8.00 (dd, *J* = 7.6 Hz, 2.0 Hz, 1H), 7.83 (dd, *J* = 7.6 Hz, 1.6 Hz, 1H), 7.38 - 7.50 (m, 3H), 7.17 - 7.19 (m, 2H), 7.01 - 7.03 (m, 1H), 2.27 (t, *J* = 7.2 Hz, 2H), 1.52 - 1.58 (m, 2H), 0.99 (t, *J* = 7.6 Hz, 3H); $^{13}C$ NMR (100 MHz, $CDCl_3$) δ 149.92, 135.85, 134.85, 134.02, 132.39, 128.60, 127.61, 127.04, 122.68, 121.69, 119.24, 96.37, 77.48, 77.16, 76.84, 75.17, 22.07, 21.79, 13.69; LC-MS ($[M+H]^+$): for $C_{17}H_{15}BrO_3S$ calcd. 379.0; found 379.2.

A mixture of compound **1c** (500 mg, 1.14 mmol), 2-methylbut-3-yn-2-ol (96 mg, 1.14 mmol), $Pd(PPh_3)_2Cl_2$ (40 mg, 5 mol%), CuI (6 mg, 3 mol%) and dry $Et_3N$ (2 ml) in dry DMF (5 ml) was stirred at room temperature for 12 h. After completion of the reaction as monitored by TLC, the reaction mixture was cooled and water (5 mL) was added and then

extracted with EtOAc (3 x 15 mL). The organic extract was washed with water (2 x 10 mL) followed by brine (10 mL) and subsequently the organic layer was dried over $MgSO_4$. Further concentration furnished a crude mass which was purified by column chromatography over silica-gel. Elution of the column with petroleum ether-ethyl acetate (9:1) mixture afforded the product **2p**.

**2-(3-hydroxy-3-methylbut-1-yn-1-yl)phenyl 2-bromobenzenesulfonate (2p):**

Brown gummy; yield = 90% (405 mg); IR (KBr): 2230, 1585 $cm^{-1}$; $^1H$ NMR (300 MHz, $CDCl_3$) δ 8.04 (dd, $J$ = 7.35 Hz, 2.4 Hz, 1H), 7.86 (dd, $J$ = 7.65 Hz, 1.5 Hz, 1H), 7.44 - 7.55 (m, 3H), 7.18 - 7.24 (m, 2H), 6.88 - 6.94 (m, 1H), 1.93 (s, 1H), 1.58 (s, 6H); $^{13}C$ NMR (75 MHz, $CDCl_3$) δ 150.02, 136.71, 136.05, 135.09, 133.73, 132.33, 129.43, 127.82, 127.14, 122.09, 121.36, 118.50, 99.92, 84.13, 65.72, 31.27; LC-MS ($[M+H]^+$): for $C_{17}H_{15}BrO_4S$ calcd. 394.9; found 395.2.

A mixture of compound **1c** (500 mg, 1.14 mmol), pent-4-yn-1-ol (96 mg, 1.14 mmol), $Pd(PPh_3)_2Cl_2$ (40 mg, 5 mol%), CuI (6 mg, 3 mol%) and dry $Et_3N$ (2 ml) in dry DMF (5 ml) was stirred at room temperature for 10 h. After completion of the reaction as monitored by TLC, the reaction mixture was cooled and water (5 mL) was added and then extracted with EtOAc (3 x 15 mL). The organic extract was washed with water (2 x 10 mL) followed by brine (10 mL) and subsequently the organic layer was dried over $MgSO_4$. Further concentration furnished a crude mass which was purified by column chromatography over silica-gel. Elution of the column with petroleum ether-ethyl acetate (9:1) mixture afforded the product **2q**.

**2-(5-hydroxypent-1-yn-1-yl)phenyl 2-bromobenzenesulfonate (2q):**

Yellow gummy; yield = 89% (401 mg); IR (KBr): 2230, 1585 $cm^{-1}$; $^1H$ NMR (400 MHz, $CDCl_3$) δ 7.99 (dd, $J$ = 8.0 Hz, 2.0 Hz, 1H), 7.85 (dd, $J$ = 7.6 Hz, 1.2 Hz, 1H), 7.39 - 7.52 (m, 3H), 7.14 - 7.21 (m, 2H), 6.87 - 6.92 (m, 1H), 3.81 (t, $J$ = 6.4 Hz, 2H), 2.47 (t, $J$ = 6.8 Hz, 2H), 1.80 (p, $J$ = 6.4 Hz, 2H), 1.76 (s, 1H); $^{13}C$ NMR (100 MHz, $CDCl_3$) δ 150.02, 136.71, 135.93, 135.03, 133.86, 132.39, 128.79, 127.71, 127.16, 122.34, 121.54, 119.24, 95.70, 75.66, 61.48, 31.04, 16.27; LC-MS ($[M+H]^+$): for $C_{17}H_{15}$BrO4S calcd 394.9; found 395.1.

A mixture of compound **1c** (500 mg, 1.14 mmol), but-3-yn-1-ol (80 mg, 1.14 mmol), $Pd(PPh_3)_2Cl_2$ (40 mg, 5 mol%), CuI (6 mg, 3 mol%) and dry $Et_3N$ (2 ml) in dry DMF (5 ml) was stirred at room temperature for 12 h. After completion of the reaction as monitored by TLC, the reaction mixture was cooled and water (5 mL) was added and then extracted with

EtOAc (3 x 15 mL). The organic extract was washed with water (2 x 10 mL) followed by brine (10 mL) and subsequently the organic layer was dried over $MgSO_4$. Further concentration furnished a crude mass which was purified by column chromatography over silica-gel. Elution of the column with petroleum ether-ethyl acetate (9:1) mixture afforded the product **2r**.

**2-(4-hydroxybut-1-yn-1-yl)phenyl 2-bromobenzenesulfonate (2r):**

Brown gummy; yield = 90% (391 mg); IR (KBr): 2230, 1585 $cm^{-1}$; $^1H$ NMR (300 MHz, $CDCl_3$) δ 8.00 (dd, $J$ = 7.65 Hz, 2.1 Hz, 1H), 7.86 (dd, $J$ = 7.5 Hz, 1.5 Hz, 1H), 7.42 - 7.55 (m, 3H), 7.16 - 7.23 (m, 2H), 6.86 - 6.93 (m, 1H), 3.81 (q, $J$ = 6.0 Hz, 2H), 2.64 (t, $J$ = 6.00 Hz, 2H), 2.47 (t, $J$ = 6.9 Hz, 1H); $^{13}C$ NMR (75 MHz, $CDCl_3$) δ 150.01, 136.45, 135.97, 135.16, 133.83, 132.38, 129.09, 127.79, 127.29, 122.41, 121.49, 119.14, 93.32, 77.58, 77.16, 77.09, 76.74, 61.00, 24.39; LC-MS ($[M+H]^+$): for $C_{16}H_{13}BrO_4S$ calcd. 380.9; found 381.1.

A mixture of compound **1c** (500 mg, 1.14 mmol), prop-2-yn-1-ol (64 mg, 1.14 mmol), $Pd(PPh_3)_2Cl_2$ (40 mg, 5 mol%), CuI (6 mg, 3 mol%) and dry $Et_3N$ (2 ml) in dry DMF (5 ml) was stirred at room temperature for 10 h. After completion of the reaction as monitored by TLC, the reaction mixture was cooled and water (5 mL) was added and then extracted with EtOAc (3 x 15 mL). The organic extract was washed with water (2 x 10 mL) followed by brine (10 mL) and subsequently the organic layer was dried over $MgSO_4$. Further concentration furnished a crude mass which was purified by column chromatography over silica-gel. Elution of the column with petroleum ether-ethyl acetate (9:1) mixture afforded the product **2s**.

**2-(3-hydroxyprop-1-yn-1-yl)phenyl 2-bromobenzenesulfonate (2s):**

Brown gummy; yield = 85% (355 mg); IR (KBr): 2230, 1585 $cm^{-1}$; $^1H$ NMR (300 MHz, $CDCl_3$) δ 8.00 (dd, $J$ = 7.7 Hz, 1.9 Hz, 1H), 7.85 (dd, $J$ = 7.6 Hz, 1.5 Hz, 1H), 7.42 - 7.52 (m, 3H), 7.19 - 7.30 (m, 2H), 7.07 (dd, $J$ = 8.2 Hz, 1.3 Hz, 1H), 4.37 (s, 2H), 1.87 (s, 1H); $^{13}C$ NMR (75 MHz, $CDCl_3$) δ 150.00, 136.62, 135.96, 135.12, 133.91, 132.41, 129.79, 127.77, 127.26, 122.96, 121.62, 117.87, 92.94, 80.14, 51.71.; LC-MS ($[M+Na]^+$): for $C_{15}H_{11}BrO_4S$ calcd. 388.9; found 389.1.

A mixture of compound **1d** (500 mg, 1.13 mmol), ethynylbenzene (116 mg, 1.13 mmol), $Pd(PPh_3)_2Cl_2$ (40 mg, 5 mol%), CuI (6 mg, 3 mol%) and dry $Et_3N$ (2 ml) in dry DMF (5 ml) was stirred at room temperature for 10 h. After completion of the reaction as monitored by TLC, the reaction mixture was cooled and water (5 mL) was added and then extracted with

EtOAc (3 x 15 mL). The organic extract was washed with water (2 x 10 mL) followed by brine (10 mL) and subsequently the organic layer was dried over $MgSO_4$. Further concentration furnished a crude mass which was purified by column chromatography over silica-gel. Elution of the column with petroleum ether-ethyl acetate (9:1) mixture afforded the product **2t**.

**2-(phenylethynyl)pyridin-3-yl 2-bromobenzenesulfonate (2t):**

White solid; m.p. 111 - 113 °C; yield = 85% (395 mg); IR (KBr): 3070, 2220, 1437, 1189 $cm^{-1}$; $^1H$ NMR (400 MHz, $CDCl_3$) δ 8.50 (dd, *J* = 4.8 Hz, 1.2 Hz, 1H), 7.98 - 8.03 (m, 1H), 7.62 - 7.66 (m, 1H), 7.55 (dd, *J* = 8.4 Hz, 1.6 Hz, 1H), 7.44 - 7.47 (m, 2H), 7.29 - 7.38 (m, 5H), 7.25 (dd, *J* = 8.4 Hz, 4.7 Hz, 1H); $^{13}C$ NMR (100 MHz, $CDCl_3$) δ 148.36, 147.40, 137.93, 136.21, 136.07, 135.22, 132.26, 132.21, 130.59, 129.44, 128.30, 127.79, 123.56, 121.82, 121.48, 94.98, 83.93; LC-MS ($[M]^+$): for $C_{19}H_{12}BrNO_3S$ calcd. 412.9; found 413.2.

A mixture of compound **1f** (500 mg, 1.09 mmol), 1-ethynyl-4-methoxybenzene (152 mg, 1.15 mmol), $Pd(PPh_3)_2Cl_2$ (38 mg, 5 mol%), CuI (6 mg, 3 mol%) and dry $Et_3N$ (2 ml) in dry DMF (5 ml) was stirred at room temperature for 10 h. After completion of the reaction as monitored by TLC, the reaction mixture was cooled and water (5 mL) was added and then extracted with EtOAc (3 x 15 mL). The organic extract was washed with water (2 x 10 mL) followed by brine (10 mL) and subsequently the organic layer was dried over $MgSO_4$. Further concentration furnished a crude mass which was purified by column chromatography over silica-gel. Elution of the column with petroleum ether-ethyl acetate (9:1) mixture afforded the product **2u**.

**4-fluoro-2-((4-methoxyphenyl)ethynyl)phenyl 2-bromobenzenesulfonate (2u):**

Brown gummy; yield = 82% (414 mg); IR (KBr): 2987, 2930, 2227, 1597 $cm^{-1}$; $^1H$ NMR (300 MHz, $CDCl_3$) δ 7.98 - 8.04 (m, 1H), 7.67 - 7.71 (m, 1H), 7.35 - 7.41 (m, 4H), 7.19 (dd, *J* = 8.6, 3.0 Hz, 1H), 7.05 (dd, *J* = 9.0, 4.8 Hz, 1H), 6.91 - 7.07 (m, 1H), 6.83 - 6.88 (m, 2H), 3.84 (s, 3H); $^{13}C$ NMR (100 MHz, $CDCl_3$) δ 161.79, 160.31, 159.33, 145.75, 145.72, 136.51, 136.01, 134.96, 133.58, 132.34, 127.70, 124.43, 124.34, 121.55, 120.73, 120.63, 119.98, 119.74, 116.18, 115.94, 114.46, 114.03, 96.05, 81.83, 81.81, 55.48; LC-MS ($[M]^+$): for $C_{21}H_{14}BrFO_4S$ calcd. 459.9; found 459.9.

Compound 2-((trimethylsilyl)ethynyl)phenyl 2-bromobenzenesulfonate (500 mg, 1.22 mmol) was dissolved in 5 ml of ethanol, add potassium carbonate (1013 mg, 7.34 mmol) and refluxed for 12 h. After completion of the reaction as monitored by TLC, the reaction mixture

was cooled and water (5 mL) was added and then extracted with EtOAc (3 x 15 mL). The organic extract was washed with water (2 x 10 mL) followed by brine (10 mL) and subsequently the organic layer was dried over $MgSO_4$. Further concentration furnished a crude mass which was purified by column chromatography over silica-gel. Elution of the column with petroleum ether-ethyl acetate (19:1) mixture afforded the product **2v**.

**2-ethynylphenyl 2-bromobenzenesulfonate (2v):**

Brown gummy; yield = 85% (350 mg); IR (KBr): 2230, 1610 $cm^{-1}$; $^1H$ NMR (300 MHz, $CDCl_3$) δ 8.01 (dd, *J* = 7.6, 1.8 Hz, 1H), 7.83 (dd, *J* = 7.7, 1.4 Hz, 1H), 7.41 - 7.53 (m, 3H), 7.20 - 7.33 (m, 2H), 7.09 (dd, *J* = 8.1, 1.5 Hz, 1H), 3.06 (s, 1H); $^{13}C$ NMR (100 MHz, $CDCl_3$) δ 150.44, 136.58, 135.99, 135.11, 134.48, 132.52, 130.13, 127.70, 127.20, 123.04, 121.88, 117.33, 82.69, 77.66; LC-MS ($[M+H]^+$): for $C_{14}H_9BrO_3S$ calcd. 336.9; found 337.1.

**4) General procedure for the reductive Heck cyclization of substrates 2a-v to form dibenzo[*b*,*e*]oxepines 3a-j & dibenzo[*c*,*f*][1,2]oxathiepine 6,6-dioxides 3k-v:**

1-bromo-2-((2-(phenylethynyl)phenoxy)methyl)benzene, **2a** (200 mg, 0.55 mmol) was dissolved in DMF (2 mL) followed by addition of $Ni(PPh_3)_4$ (32 mg, 0.027 mmol), HCOONa (112 mg, 1.65 mmol) and distilled water (1 mL) and heated under continuous stirring at 100 °C for 12 h. Upon completion of the reaction, as monitored by TLC, the reaction mixture was cooled. The reaction mixture was diluted with EtOAc (40 mL), washed with water (2 x 40 mL), dried over $MgSO_4$ and concentrated under reduced pressure. The crude product was subjected to column chromatography on silica gel with petrolium ether - EtOAc (19:1) as eluent to give pure product **3a**. Similarly other compounds **2b-v** were subjected to optimized reaction conditions to produce the corresponding products **3b-v**.

**(*Z*)-11-benzylidene-6,11-dihydrodibenzo[*b*,*e*]oxepine (3a):**

Brown solid; m.p. 64 - 66 °C; yield = 74% (116 mg); IR (KBr): 2927, 1601 $cm^{-1}$; $^1H$ NMR (400 MHz, $CDCl_3$) δ 7.48 (d, *J* = 7.4 Hz, 1H), 7.36 - 7.42 (m, 3H), 7.32 - 7.33 (m, 2H), 7.23 - 7.27 (m, 2H), 7.10 - 7.21 (m, 3H), 6.89 (dd, *J* = 8.1 Hz, 1.3 Hz, 1H), 6.62 - 6.71 (m, 2H), 5.35 (s, 2H); $^{13}C$ NMR (100 MHz, $CDCl_3$) δ 155.79, 146.02, 140.44, 136.90, 134.06, 132.34, 130.70, 129.75, 129.68, 129.42, 128.32, 127.91, 127.22, 125.73, 124.00, 120.85, 119.90, 70.38; HRMS (ESI $[M+H]^+$) for $C_{21}H_{16}O$ calcd. 285.1279; found 285.1273.

1-bromo-2-((2-((4-methoxyphenyl)ethynyl)phenoxy)methyl)benzene, **2b** (200 mg, 0.51 mmol) was dissolved in DMF (2 mL) followed by addition of $Ni(PPh_3)_4$ (30 mg, 0.025

mmol), HCOONa (104 mg, 1.525 mmol) and distilled water (1 mL) and heated under continuous stirring at 100 °C for 12 h. Upon completion of the reaction, as monitored by TLC, the reaction mixture was cooled. The reaction mixture was diluted with EtOAc (40 mL), washed with water (2 x 40 mL), dried over $MgSO_4$ and concentrated under reduced pressure. The crude product was subjected to column chromatography on silica gel with petrolium ether - EtOAc (19:1) as eluent to give pure **3b**.

**(*Z*)-11-(4-methoxybenzylidene)-6,11-dihydrodibenzo[*b*,*e*]oxepine (3b):**

Brown gummy; yield = 76% (121 mg); IR (KBr): 2926, 1599 $cm^{-1}$; $^1H$ NMR (300 MHz, $CDCl_3$) δ. 7.46 - 7.49 (m, 1H), 7.36 - 7.40 (m, 1H), 7.29 - 7.36 (m, 4H), 7.09 - 7.22 (m, 2H), 6.89 (dd, *J* = 8.3, 1.3 Hz, 1H), 6.76 - 6.81 (m, 2H), 6.67 - 6.74 (m, 1H), 6.59 (s, 1H), 5.34 (s, 2H), 3.80 (s, 3H); $^{13}C$ NMR (75 MHz, $CDCl_3$) δ 158.78, 155.69, 146.27, 138.31, 134.08, 132.23, 130.91, 130.64, 130.22, 129.55, 129.37, 129.28, 127.87, 127.71, 125.66, 124.18, 120.84, 119.88, 113.73, 113.60, 70.35, 55.30; HRMS (ESI $[M+H]^+$) for $C_{22}H_{18}O_2$ calcd. 315.1385; found 315.1390.

1-bromo-2-((2-(p-tolylethynyl)phenoxy)methyl)benzene, **2c** (200 mg, 0.53 mmol) was dissolved in DMF (2 mL) followed by addition of $Ni(PPh_3)_4$ (31 mg, 0.026 mmol), HCOONa (108 mg, 1.59 mmol) and distilled water (1 mL) and heated under continuous stirring at 100 °C for 12 h. Upon completion of the reaction, as monitored by TLC, the reaction mixture was cooled. The reaction mixture was diluted with EtOAc (40 mL), washed with water (2 x 40 mL), dried over $MgSO_4$ and concentrated under reduced pressure. The crude product was subjected to column chromatography on silica gel with petrolium ether - EtOAc (19:1) as eluent to give pure **3c**.

**(*Z*)-11-(4-methylbenzylidene)-6,11-dihydrodibenzo[*b*,*e*]oxepine (3c):**

White solid; m.p. 67 - 69 °C; yield = 76% (120 mg); IR (KBr): 2930, 1603 $cm^{-1}$; $^1H$ NMR (300 MHz, $CDCl_3$) δ 7.47 (d, *J* = 7.4 Hz, 1H), 7.35 - 7.42 (m, 1H), 7.31 (dd, *J* = 3.9 Hz, 1.0 Hz, 2H), 7.26 (d, *J* = 8.0 Hz, 2H), 7.11 (td, *J* = 7.5 Hz, 1.5 Hz, 2H), 7.05 (d, *J* = 7.9 Hz, 2H), 6.85 - 6.90 (m, 1H), 6.67 (td, *J* = 7.5 Hz, 1.3 Hz, 1H), 6.61 (s, 1H), 5.34 (s, 2H), 2.32 (s, 3H); $^{13}C$ NMR (75 MHz, $CDCl_3$) δ 155.71, 146.21, 139.47, 137.08, 134.08, 133.93, 132.31, 130.68, 129.64, 129.59, 129.40, 129.04, 127.89, 127.81, 125.71, 124.14, 120.83, 119.86, 70.38, 21.38; HRMS (ESI $[M+K]^+$) for $C_{22}H_{18}O$ calcd. 337.0995 ; found 337.0871.

1-bromo-2-((2-(hept-1-yn-1-yl)phenoxy)methyl)benzene, **2d** (200 mg, 0.56 mmol) was dissolved in DMF (2 mL) followed by addition of $Ni(PPh_3)_4$ (33 mg, 0.028 mmol), HCOONa

(114 mg, 1.68 mmol) and distilled water (1 mL) and heated under continuous stirring at 100 $^{o}$C for 6 h. Upon completion of the reaction, as monitored by TLC, the reaction mixture was cooled. The reaction mixture was diluted with EtOAc (40 mL), washed with water (2 x 40 mL), dried over $MgSO_4$ and concentrated under reduced pressure. The crude product was subjected to column chromatography on silica gel with petrolium ether - EtOAc (19:1) as eluent to give pure **3d**.

**(*Z*)-11-hexylidene-6,11-dihydrodibenzo[*b*,*e*]oxepine (3d):**

White gummy; yield = 80% (125 mg); IR (KBr): 2929, 1602 cm$^{-1}$; $^{1}$H NMR (300 MHz, $CDCl_3$) δ 7.26 - 7.36 (m, 4H), 7.12 - 7.18 (m, 2H), 6.85 - 6.92 (m, 2H), 5.70 (t, *J* = 7.4 Hz, 1H), 5.23 (s, 2H), 2.42 (q, *J* = 7.4 Hz, 2H), 1.47 - 1.55 (m, 2H), 1.28 - 1.36 (m, 4H), 0.87 - 0.92 (m, 3H); $^{13}$C NMR (75 MHz, $CDCl_3$) δ 155.59, 146.19, 138.91, 133.93, 133.68, 131.69, 129.19, 129.13, 127.56, 127.45, 126.39, 124.34, 120.42, 119.62, 70.53, 31.69, 29.83, 29.72, 22.67, 14.18; HRMS (ESI [M+K]) for $C_{20}H_{22}O$ calcd. 317.1308; found 317.1873.

1-bromo-2-((2-(hex-1-yn-1-yl)phenoxy)methyl)benzene, **2e** (200 mg, 0.58 mmol) was dissolved in DMF (2 mL) followed by addition of $Ni(PPh_3)_4$ (34 mg, 0.029 mmol), HCOONa (119 mg, 1.75 mmol) and distilled water (1 mL) and heated under continuous stirring at 100 $^{o}$C for 6 h. Upon completion of the reaction, as monitored by TLC, the reaction mixture was cooled. The reaction mixture was diluted with EtOAc (40 mL), washed with water (2 x 40 mL), dried over $MgSO_4$ and concentrated under reduced pressure. The crude product was subjected to column chromatography on silica gel with petrolium ether - EtOAc (19:1) as eluent to give pure **3e**.

**(*Z*)-11-pentylidene-6,11-dihydrodibenzo[*b*,*e*]oxepine (3e):**

White gummy; yield = 82% (126 mg); IR (KBr): 2928, 1603 cm$^{-1}$; $^{1}$H NMR (300 MHz, $CDCl_3$) δ 7.52 - 7.49 (m, 2H), 7.42 - 7.29 (m, 4H), 7.23 - 7.18 (m, 1H), 6.93 - 6.87 (m, 1H), 5.16 (s, 2H), 2.48 (t, *J* = 6.9 Hz, 2H), 1.67 - 1.55 (m, 2H), 1.54 - 1.43 (m, 2H), 0.93 (t, *J* = 7.2 Hz, 2H); $^{13}$C NMR (75 MHz, $CDCl_3$) δ 159.29, 137.28, 133.57, 128.82, 128.53, 127.80, 127.12, 120.95, 114.30, 112.94, 94.93, 70.55, 31.04, 22.10, 19.56, 13.80. HRMS (ESI $[M+H]^+$): for $C_{19}H_{20}O$ calcd. 265.1592; found 265.1608 .

4-(2-((2-bromobenzyl)oxy)phenyl)but-3-yn-1-ol, **2f** (200 mg, 0.60 mmol) was dissolved in DMF (2 mL) followed by addition of $Ni(PPh_3)_4$ (35 mg, 0.03 mmol), HCOONa (123 mg, 1.81 mmol) and distilled water (1 mL) and heated under continuous stirring at 100 $^{o}$C for 8 h. Upon completion of the reaction, as monitored by TLC, the reaction mixture was cooled. The

reaction mixture was diluted with EtOAc (40 mL), washed with water (2 x 40 mL), dried over $MgSO_4$ and concentrated under reduced pressure. The crude product was subjected to column chromatography on silica gel with petrolium ether - EtOAc (9:1) as eluent to give pure **3f**.

**(*Z*)-3-(dibenzo[*b*,*e*]oxepin-11(6*H*)-ylidene)propan-1-ol (3f):**

Yellow solid; m.p. 82 - 84 °C; yield = 75% (114 mg); IR (KBr): 2925, 1599 $cm^{-1}$; $^1H$ NMR (400 MHz, $CDCl_3$) δ 7.29 - 7.33 (m, 2H), 7.23 - 7.28 (m, 2H), 7.12 - 7.21 (m, 2H), 6.90 (ddd, *J* = 14.4 Hz, 7.8 Hz, 1.3 Hz, 2H), 5.75 (t, *J* = 7.4 Hz, 1H), 5.23 (s, 2H), 3.78 (t, *J* = 6.5 Hz, 2H), 2.70 (q, *J* = 6.7 Hz, 2H); $^{13}C$ NMR (100 MHz, $CDCl_3$) δ 155.66, 145.48, 141.73, 133.82, 131.43, 129.44, 129.15, 128.76, 127.68, 127.52, 126.36, 124.40, 120.73, 119.82, 70.66, 62.73, 33.24; HRMS (ESI $[M+H]^+$): for $C_{17}H_{16}O_2$ calcd. 253.1229; found 253.1216.

1-bromo-4-methoxy-2-((2-(oct-1-yn-1-yl)phenoxy)methyl)benzene, **2g** (200 mg, 0.50 mmol) was dissolved in DMF (2 mL) followed by addition of $Ni(PPh_3)_4$ (29 mg, 0.025 mmol), HCOONa (102 mg, 1.49 mmol) and distilled water (1 mL) and heated under continuous stirring at 100 °C for 12 h . Upon completion of the reaction, as monitored by TLC, the reaction mixture was cooled. The reaction mixture was diluted with EtOAc (40 mL), washed with water (2 x 40 mL), dried over $MgSO_4$ and concentrated under reduced pressure. The crude product was subjected to column chromatography on silica gel with petrolium ether - EtOAc (19:1) as eluent to give pure **3g.**

**(*Z*)-11-heptylidene-8-methoxy-6,11-dihydrodibenzo[*b*,*e*]oxepine (3g):**

Yellow solid; m.p. 123 - 125 °C; yield = 75% (137 mg); IR (KBr): 2923, 1596 $cm^{-1}$; $^1H$ NMR (400 MHz, $CDCl_3$) δ 7.13 - 7.24 (m, 3H), 6.82 - 6.92 (m, 4H), 5.67 (t, *J* = 7.4 Hz, 1H), 5.19 (s, 2H), 3.81 (s, 3H), 2.40 (q, *J* = 7.2 Hz, 2H), 1.50 (p, *J* = 7.2 Hz, 2H), 1.28 - 1.39 (m, 6H), 0.89 (t, *J* = 6.4 Hz, 3H); $^{13}C$ NMR (100 MHz, $CDCl_3$) δ 159.04, 155.57, 138.55, 138.21, 135.03, 133.25, 131.49, 128.98, 127.65, 125.23, 120.57, 119.66, 114.11, 113.07, 70.80, 55.52, 31.84, 30.15, 29.77, 29.16, 22.76, 14.22; HRMS (ESI $[M+H]^+$): for $C_{22}H_{26}O_2$ calcd. 323.2011; found 323.0704.

4-(2-((2-bromo-5-methoxybenzyl)oxy)phenyl)-2-methylbut-3-yn-2-ol, **2h** (200 mg, 0.53 mmol) was dissolved in DMF (2 mL) followed by addition of $Ni(PPh_3)_4$ (31 mg, 0.026 mmol), HCOONa ( 108 mg, 1.60 mmol) and distilled water (1 mL) and heated under continuous stirring at 100 °C for 12 h. Upon completion of the reaction, as monitored by TLC, the reaction mixture was cooled. The reaction mixture was diluted with EtOAc (40

mL), washed with water (2 x 40 mL), dried over $MgSO_4$ and concentrated under reduced pressure. The crude product was subjected to column chromatography on silica gel with petrolium ether - EtOAc (9:1) as eluent to give pure **3h**.

**(*Z*)-1-(8-methoxydibenzo[b,e]oxepin-11(6*H*)-ylidene)-2-methylpropan-2-ol (3h):**

Brown gummy; yield = 86% (136 mg); IR (KBr): 2921, 1594 $cm^{-1}$; $^{1}H$ NMR (300 MHz, $CDCl_3$) δ 7.40 (dd, $J$ = 7.7, 1.8 Hz, 1H), 7.22 - 7.32 (m, 2H), 7.02 - 7.13 (m, 2H), 6.89 - 6.94 (m, 2H), 6.85 (dd, $J$ = 8.1, 2.1 Hz, 1H), 5.12 (s, 2H), 3.82 (s, 3H), 1.93 (s, 1H), 1.63 (s, 6H); $^{13}C$ NMR (75 MHz, $CDCl_3$) δ 159.91, 159.39, 138.80, 133.48, 129.74, 129.60, 121.04, 119.18, 119.15, 113.04, 112.99, 112.97, 112.95, 98.40, 78.58, 70.39, 65.84, 55.42, 31.61; HRMS (ESI $[M+Na]^+$): for $C_{19}H_{20}O_3$ calcd. 319.1310; found 319.1322.

1-bromo-4-methoxy-2-((2-(pent-1-yn-1-yl)phenoxy)methyl)benzene, **2i** (200 mg, 0.55 mmol) was dissolved in DMF (2 mL) followed by addition of $Ni(PPh_3)_4$ (32 mg, 0.028 mmol), HCOONa (113 mg, 1.67 mmol) and distilled water (1 mL) and heated at under continuous stirring at 100 °C for 6 h. Upon completion of the reaction, as monitored by TLC, the reaction mixture was cooled. The reaction mixture was diluted with EtOAc (40 mL), washed with water (2 x 40 mL), dried over $MgSO_4$ and concentrated under reduced pressure. The crude product was subjected to column chromatography on silica gel with petrolium ether - EtOAc (19:1) as eluent to give pure **3i.**

**(*Z*)-11-butylidene-8-methoxy-6,11-dihydrodibenzo[*b*,*e*]oxepine (3i):**

Brown gummy; yield = 85% (133 mg); IR (KBr): 2927, 1603 $cm^{-1}$; $^{1}H$ NMR (400 MHz, $CDCl_3$) δ 7.22 (d, $J$ = 8.0 Hz, 1H), 7.13 - 7.17 (m, 2H), 6.81 - 6.92 (m, 4H), 5.67 (t, $J$ = 7.2 Hz, 1H), 5.19 (s, 2H), 2.38 (q, $J$ = 7.4 Hz, 2H), 2.38 (q, $J$ = 7.4 Hz, 2H), 0.96 (t, J = 7.2 Hz, 3H); $^{13}C$ NMR (75 MHz, $CDCl_3$) δ 159.05, 155.58, 138.53, 138.40, 135.03, 133.04, 131.51, 128.99, 127.66, 125.27, 120.59, 119.67, 114.12, 113.07, 55.54, 31.84, 23.39, 14.04; HRMS (ESI $[M+H]^+$): for $C_{19}H_{20}O_2$ calcd. 281.1542; found 281.1559.

4-(3-((2-bromobenzyl)oxy)pyridin-2-yl)-2-methylbut-3-yn-2-ol, **2j** (200 mg, 0.58 mmol) was dissolved in DMF (2 mL) followed by addition of $Ni(PPh_3)_4$ (34 mg, 0.029 mmol), HCOONa (118 mg, 1.73 mmol) and distilled water (1 mL) and heated under continuous stirring at 100 °C for 12 h. Upon completion of the reaction, as monitored by TLC, the reaction mixture was cooled. The reaction mixture was diluted with EtOAc (40 mL), washed with water (2 x 40 mL), dried over $MgSO_4$ and concentrated under reduced pressure. The crude product was

subjected to column chromatography on silica gel with petrolium ether - EtOAc (9:1) as eluent to give pure **3j**.

**(*E*)-1-(benzo[5,6]oxepino[3,2-*b*]pyridin-11(6*H*)-ylidene)-2-methylpropan-2-ol (3j):**

Brown gummy; yield = 78% (120 mg); IR (KBr): 2898, 1572 $cm^{-1}$; $^{1}H$ NMR (400 MHz, $CDCl_3$) δ 8.16 - 8.18 (m, 1H), 7.27 - 7.43 (m, 4H), 7.20 - 7.22 (m, 1H), 7.12 - 7.15 (m, 1H), 6.13 (s, 1H), 5.21 (s, 2H), 1.72 (s, 1H), 1.41 (s, 6H); $^{13}C$ NMR (100 MHz, $CDCl_3$) δ 153.59, 146.05, 144.66, 141.26, 140.54, 139.38, 133.62, 129.95, 128.77, 128.27, 127.89, 127.61, 124.43, 77.36, 70.53, 68.79, 30.68; HRMS (ESI [M+H]): for $C_{17}H_{17}NO_2$ calcd. 268.1338; found 268.1331.

2-(phenylethynyl)phenyl 2-bromobenzenesulfonate, **2k** (200 mg, 0.48 mmol) was dissolved in DMF (2 mL) followed by addition of $Ni(PPh_3)_4$ (28 mg, 0.024 mmol), HCOONa (99 mg, 1.45 mmol) and distilled water (1 mL) and heated under continuous stirring at 100 $^{o}C$ for 12 h. Upon completion of the reaction, as monitored by TLC, the reaction mixture was cooled. The reaction mixture was diluted with EtOAc (40 mL), washed with water (2 x 40 mL), dried over $MgSO_4$ and concentrated under reduced pressure. The crude product was subjected to column chromatography on silica gel with petrolium ether - EtOAc (19:1) as eluent to give pure **3k**.

**(*E*)-11-benzylidene-11*H*-dibenzo[*c*,*f*][1,2]oxathiepine 6,6-dioxide (3k):**

Yellow solid; m.p. 152 - 154 $^{o}C$; yield = 80% (129 mg); IR (KBr): 3062, 1484, 1353, 1176 $cm^{-1}$; $^{1}H$ NMR (300 MHz, $CDCl_3$) δ 7.92 - 7.96 (m, 1H), 7.61 - 7.69 (m, 2H), 7.48 - 7.54 (m, 1H), 7.34 - 7.45 (m, 2H), 7.18 - 7.21 (m, 3H), 7.06 - 7.14 (m, 4H), 7.03 (s, 1H); $^{13}C$ NMR (75 MHz, $CDCl_3$) δ 147.65, 139.62, 136.19, 135.65, 135.57, 133.74, 131.38, 130.99, 130.22, 129.63, 129.21, 128.96, 128.41, 128.12, 128.03, 127.36, 122.83; HRMS (ESI [M]): for $C_{20}H_{14}O_3S$ calcd. 334.0664; found 334.0688.

2-(oct-1-yn-1-yl)phenyl 2-bromobenzenesulfonate, **2l** (200 mg, 0.47 mmol) was dissolved in DMF (2 mL) followed by addition of $Ni(PPh_3)_4$ (28 mg, 0.024 mmol), HCOONa (97 mg, 1.42 mmol) and distilled water (1 mL) and heated under continuous stirring at 100 $^{o}C$ for 6 h. Upon completion of the reaction, as monitored by TLC, the reaction mixture was cooled. The reaction mixture was diluted with EtOAc (40 mL), washed with water (2 x 40 mL), dried over $MgSO_4$ and concentrated under reduced pressure. The crude product was subjected to column chromatography on silica gel with petrolium ether - EtOAc (9:1) as eluent to give pure **3l**.

**(*E*)-11-heptylidene-11*H*-dibenzo[*c,f*][1,2]oxathiepine 6,6-dioxide (3l):**

Brown gummy; yield = 94% (153 mg); IR (KBr): 3064, 1487, 1355, 1179 $cm^{-1}$; $^{1}H$ NMR (300 MHz, $CDCl_3$) δ 7.85 - 7.88 (m, 1H), 7.53 (dd, *J* = 7.2 Hz, 1.5 Hz, 1H), 7.39 - 7.47 (m, 4H), 7.24 - 7.32 (m, 3H), 6.14 (t, *J* = 7.6 Hz, 1H), 2.17 (q, *J* = 7.5 Hz, 2H), 1.46 (p, *J* = 7.1 Hz, 2H), 1.18 - 1.34 (m, 6H), 0.86 (t, *J* = 6.9 Hz, 3H); $^{13}C$ NMR (75 MHz, CDCl3) δ 147.04, 139.35, 138.92, 136.13, 135.03, 133.27, 132.29, 130.41, 129.83, 129.80, 128.61, 127.86, 127.22, 123.00, 31.69, 29.78, 29.45, 28.91, 22.67, 14.18; HRMS (ESI $[M+Na]^+$): for $C_{20}H_{22}O_3S$ calcd. 365.1187; found 365.1184.

2-(hept-1-yn-1-yl)phenyl 2-bromobenzenesulfonate, **2m** (200 mg, 0.49 mmol) was dissolved in DMF (2 mL) followed by addition of $Ni(PPh_3)_4$ (29 mg, 0.024 mmol), HCOONa (100 mg, 1.47 mmol) and distilled water (1 mL) and heated under continuous stirring at 100 °C for 6 h. Upon completion of the reaction, as monitored by TLC, the reaction mixture was cooled. The reaction mixture was diluted with EtOAc (40 mL), washed with water (2 x 40 mL), dried over $MgSO_4$ and concentrated under reduced pressure. The crude product was subjected to column chromatography on silica gel with petrolium ether - EtOAc (9:1) as eluent to give pure **3m**.

**(*E*)-11-hexylidene-11*H*-dibenzo[*c,f*][1,2]oxathiepine 6,6-dioxide (3m):**

White gummy; yield = 91% (147 mg); IR (KBr): 3068, 1491, 1357, 1183 $cm^{-1}$; $^{1}H$ NMR (300 MHz, $CDCl_3$) δ 7.85 - 7.88 (m, 1H), 7.52 - 7.55 (m, 1H), 7.39 - 7.47 (m, 4H), 7.27 - 7.32 (m, 1H), 7.23 (t, *J* = 1.3 Hz, 1H), 6.14 (t, *J* = 7.6 Hz, 1H), 2.13 - 2.22 (m, 4H), 1.46 (p, *J* = 7.5 Hz, 2H), 1.24 - 1.29 (m, 4H), 0.84 - 0.90 (m, 3H); $^{13}C$ NMR (75 MHz, $CDCl_3$) δ 146.94, 139.24, 138.86, 136.01, 134.92, 133.24, 132.16, 130.35, 129.75, 128.53, 127.72, 127.16, 122.87, 31.31, 29.66, 29.07, 22.44, 13.99; HRMS (ESI $[M+H]^+$): for $C_{19}H_{20}O_3S$ calcd. 329.1211; found 329.1222.

2-(hex-1-yn-1-yl)phenyl 2-bromobenzenesulfonate, **2n** (200 mg, 0.51 mmol) was dissolved in DMF (2 mL) followed by addition of $Ni(PPh_3)_4$ (30 mg, 0.025 mmol), HCOONa (104 mg, 1.52 mmol) and distilled water (1 mL) and heated at under continuous stirring at 100 °C for 6 h. Upon completion of the reaction, as monitored by TLC, the reaction mixture was cooled. The reaction mixture was diluted with EtOAc (40 mL), washed with water (2 x 40 mL), dried over $MgSO_4$ and concentrated under reduced pressure. The crude product was subjected to

column chromatography on silica gel with petrolium ether - EtOAc (19:1) as eluent to give pure **3n**.

**(*E*)-11-pentylidene-11*H*-dibenzo[*c*,*f*][1,2]oxathiepine 6,6-dioxide (3n):**

White gummy; yield = 90% (144 mg); IR (KBr): 3060, 1481, 1349, 1173 $cm^{-1}$; $^1H$ NMR (300 MHz, $CDCl_3$) δ 7.85 - 7.88 (m, 1H), 7.52 - 7.57 (m, 1H), 7.38 - 7.47 (m, 4H), 7.24 - 7.32 (m, 2H), 6.14 (t, $J$ = 7.8 Hz, 1H), 2.18 (q, $J$ = 7.5 Hz, 2H), 1.44 (p, $J$ = 7.2 Hz, 2H), 1.25 - 1.35 (m, 2H), 0.85 (t, $J$ = 7.2 Hz, 3H); $^{13}C$ NMR (75 MHz, $CDCl_3$) δ 146.91, 139.22, 138.79, 135.98, 134.91, 133.17, 132.14, 130.30, 129.73, 129.69, 128.51, 127.74, 127.12, 122.88, 31.53, 29.41, 22.23, 13.87; HRMS (ESI $[M+H]^+$): for $C_{18}H_{18}O_3S$ calcd. 315.1055; found 315.1064.

2-(hex-1-yn-1-yl)phenyl 2-bromobenzenesulfonate, **2o** (200 mg, 0.53 mmol) was dissolved in DMF (2 mL) followed by addition of $Ni(PPh_3)_4$ (31 mg, 0.026 mmol), HCOONa (107 mg, 1.58 mmol) and distilled water (1 mL) and heated under continuous stirring at 100 °C for 8 h. Upon completion of the reaction, as monitored by TLC, the reaction mixture was cooled. The reaction mixture was diluted with EtOAc (40 mL), washed with water (2 x 40 mL), dried over $MgSO_4$ and concentrated under reduced pressure. The crude product was subjected to column chromatography on silica gel with petrolium ether - EtOAc (19:1) as eluent to give pure **3o.**

**(*E*)-11-butylidene-11*H*-dibenzo[*c*,*f*][1,2]oxathiepine 6,6-dioxide (3o):**

Yellow gummy; yield = 86% (136 mg); IR (KBr): 3052, 1478, 1341, 1169 $cm^{-1}$; $^1H$ NMR (400 MHz, $CDCl_3$) δ 7.86 (dd, $J$ = 8.2, 1.6 Hz, 1H), 7.52 - 7.56 (m, 1H), 7.43 - 7.47 (m, 2H), 7.39 - 7.40 (m, 2H), 7.29 - 7.32 (m, 2H), 6.14 (t, $J$ = 7.6 Hz, 1H), 2.16 (q, $J$ = 7.2 Hz, 2H), 1.45 - 1.53 (m, 2H), 0.92 (t, $J$ = 7.6 Hz, 3H); $^{13}C$ NMR (100 MHz, $CDCl_3$) δ 147.06, 139.39, 138.68, 136.17, 135.28, 133.28, 132.27, 130.43, 129.84, 129.79, 128.63, 127.88, 127.22, 123.01, 31.83, 22.76, 13.86; HRMS (ESI $[M+Na]^+$): for $C_{17}H_{16}O_3S$ calcd. 323.0718; found 323.0704.

2-(3-hydroxy-3-methylbut-1-yn-1-yl)phenyl 2-bromobenzenesulfonate, **2p** (200 mg, 0.50 mmol) was dissolved in DMF (2 mL) followed by addition of $Ni(PPh_3)_4$ (30 mg, 0.025 mmol), HCOONa (103 mg, 1.52 mmol) and distilled water (1 mL) and heated under continuous stirring at 100 °C for 8 h. Upon completion of the reaction, as monitored by TLC, the reaction mixture was cooled. The reaction mixture was diluted with EtOAc (40 mL), washed with water (2 x 40 mL), dried over $MgSO_4$ and concentrated under reduced pressure.

The crude product was subjected to column chromatography on silica gel with petrolium ether - EtOAc (4:1) as eluent to give pure **3p**.

**(*E*)-11-(2-hydroxy-2-methylpropylidene)-11*H*-dibenzo[*c,f*][1,2]oxathiepine 6,6-dioxide (3p):**

Brown gummy; yield = 84% (134 mg); IR (KBr): 2954, 1581, 1369, 1184 $cm^{-1}$; $^{1}H$ NMR (300 MHz, $CDCl_3$) δ 7.84 (dd, *J* = 7.8 Hz, 1.5 Hz, 1H), 7.54 (td, *J* = 7.5 Hz, 1.5 Hz, 1H), 7.36 - 7.48 (m, 5H), 7.24 - 7.30 (m, 1H), 6.21 (s, 1H), 1.38 (d, *J* = 8.1 Hz, 6H); $^{13}C$ NMR (75 MHz, $CDCl_3$) δ 146.05, 144.17, 140.22, 135.94, 133.53, 133.47, 133.33, 130.42, 130.16, 129.23, 129.06, 128.21, 127.24, 122.98, 71.84, 31.19, 31.00; HRMS (ESI $[M+Na]^+$): for $C_{17}H_{16}O_4S$ calcd. 339.0667; found 339.0658.

2-(5-hydroxypent-1-yn-1-yl)phenyl 2-bromobenzenesulfonate, **2q** (200 mg, 0.50 mmol) was dissolved in DMF (2 mL) followed by addition of $Ni(PPh_3)_4$ (30 mg, 0.025 mmol), HCOONa (103 mg, 1.52 mmol) and distilled water (1 mL) and heated under continuous stirring at 100 $^oC$ for 8 h. Upon completion of the reaction, as monitored by TLC, the reaction mixture was cooled. The reaction mixture was diluted with EtOAc (40 mL), washed with water (2 x 40 mL), dried over $MgSO_4$ and concentrated under reduced pressure. The crude product was subjected to column chromatography on silica gel with petrolium ether - EtOAc (4:1) as eluent to give pure **3q.**

**(*E*)-11-(4-hydroxybutylidene)-11*H*-dibenzo[*c,f*][1,2]oxathiepine 6,6-dioxide (3q):**

Brown gummy; yield = 81% (130 mg); IR (KBr): 2952, 1576, 1367, 1180 $cm^{-1}$; $^{1}H$ NMR (300 MHz, $CDCl_3$) δ 7.86 - 7.90 (m, 1H), 7.54 - 7.59 (m, 1H), 7.43 - 7.49 (m, 2H), 7.35 - 7.40 (m, 2H), 7.28 - 7.30 (m, 2H), 6.21 (t, *J* = 7.5 Hz, 1H), 3.63 (t, *J* = 6.3 Hz, 2H), 2.33 (q, *J* = 7.5 Hz, 2H), 1.68 - 1.77 (m, 2H); $^{13}C$ NMR (75 MHz, $CDCl_3$) δ 147.19, 139.34, 137.92, 135.90, 135.83, 133.57, 131.06, 130.64, 130.00, 129.69, 128.68, 127.78, 127.11, 122.77, 77.58, 77.16, 76.74, 62.04, 32.18, 26.33; HRMS (ESI $[M+H]^+$): for $C_{17}H_{16}O_4S$ calcd. 317.0846; found 317.0844.

2-(4-hydroxybut-1-yn-1-yl)phenyl 2-bromobenzenesulfonate, **2r** (200 mg, 0.52 mmol) was dissolved in DMF (2 mL) followed by addition of $Ni(PPh_3)_4$ (31 mg, 0.026 mmol), HCOONa (107 mg, 1.57 mmol) and distilled water (1 mL) and heated under continuous stirring at 100 $^oC$ for 10 h. Upon completion of the reaction, as monitored by TLC, the reaction mixture was cooled. The reaction mixture was diluted with EtOAc (40 mL), washed with water (2 x 40 mL), dried over $MgSO_4$ and concentrated under reduced pressure. The crude product was

subjected to column chromatography on silica gel with petrolium ether - EtOAc (4:1) as eluent to give pure **3r**.

**(*E*)-11-(3-hydroxypropylidene)-11*H*-dibenzo[*c,f*][1,2]oxathiepine 6,6-dioxide (3r):**

Brown gummy; yield = 76% (120 mg); IR (KBr): 2951, 1578, 1361, 1179 $cm^{-1}$; $^1H$ NMR (300 MHz, $CDCl_3$) δ 7.87 - 7.90 (m, 1H), 7.54 - 7.60 (m, 1H), 7.54 - 7.60 (m, 1H), 7.43 - 7.48 (m, 2H), 7.35 - 7.41 (m, 2H), 7.28 - 7.33 (m, 2H), 6.14 (t, *J* = 7.8 Hz, 1H), 3.72 (t, *J* = 6.3 Hz, 2H), 2.49 (q, *J* = 6.3 Hz, 2H); $^{13}C$ NMR (75 MHz, $CDCl_3$) δ 13C NMR (75 MHz, CDCl3) δ 147.28, 139.29, 137.89, 135.70, 134.69, 133.72, 130.77, 130.39, 130.09, 129.60, 128.76, 127.67, 127.05, 122.63, 77.58, 77.16, 76.74, 61.92, 33.13; HRMS (ESI $[M+Na]^+$): for $C_{16}H_{14}O_4S$ calcd. 325.0510; found 325.0552.

2-(3-hydroxyprop-1-yn-1-yl)phenyl 2-bromobenzenesulfonate, **2s** (200 mg, 0.69 mmol) was dissolved in DMF (2 mL) followed by addition of $Ni(PPh_3)_4$ (41 mg, 0.035 mmol), HCOONa (141 mg, 2.08 mmol) and distilled water (1 mL) and heated under continuous stirring at 100 $^oC$ for 10 h. Upon completion of the reaction, as monitored by TLC, the reaction mixture was cooled. The reaction mixture was diluted with EtOAc (40 mL), washed with water (2 x 40 mL), dried over $MgSO_4$ and concentrated under reduced pressure. The crude product was subjected to column chromatography on silica gel with petrolium ether - EtOAc (4:1) as eluent to give pure **3s**.

**(*E*)-11-(2-hydroxyethylidene)-11*H*-dibenzo[*c,f*][1,2]oxathiepine 6,6-dioxide (3s):**

Yellow gummy; yield = 76% (119 mg); IR (KBr): 2947, 1570, 1358, 1178 $cm^{-1}$; $^1H$ NMR (300 MHz, $CDCl_3$) δ 7.89 - 7.92 (m, 1H), 7.57 - 7.62 (m, 1H), 7.47 - 7.52 (m, 2H), 7.35 - 7.45 (m, 2H), 7.22 - 7.32 (m, 2H), 6.28 (t, *J* = 6.8 Hz, 1H), 4.27 (d, *J* = 6.9 Hz, 2H), 2.16 (s, 1H); $^{13}C$ NMR (75 MHz, $CDCl_3$) δ 146.97, 138.42, 137.59, 136.08, 136.00, 133.74, 130.52, 130.51, 130.09, 129.65, 129.14, 127.84, 127.14, 122.79, 60.27; HRMS (ESI $[M+Na]^+$): for $C_{15}H_{12}O_4S$ calcd. 311.0354; found 311.0279.

2-(phenylethynyl)pyridin-3-yl 2-bromobenzenesulfonate, **2t** (200 mg, 0.48 mmol) was dissolved in DMF (2 mL) followed by addition of $Ni(PPh_3)_4$ (28 mg, 0.024 mmol), HCOONa ( 98 mg, 1.45 mmol) and distilled water (1 mL) and heated under continuous stirring at 100 $^oC$ for 12 h. Upon completion of the reaction, as monitored by TLC, the reaction mixture was cooled. The reaction mixture was diluted with EtOAc (40 mL), washed with water (2 x 40 mL), dried over $MgSO_4$ and concentrated under reduced pressure. The crude product was

subjected to column chromatography on silica gel with petrolium ether - EtOAc (9:1) as eluent to give pure **3t**.

**(*Z*)-11-benzylidene-11*H*-benzo[3,4][1,2]oxathiepino[6,7-*b*]pyridine 6,6-dioxide (3t):**
White solid; m.p. 161- 163 °C; yield = 80% (129 mg); IR (KBr): 3062, 1579, 1369, 1184 $cm^{-1}$; $^1H$ NMR (300 MHz, $CDCl_3$) δ 8.38 (dd, *J* = 4.7 Hz, 1.4 Hz, 1H), 7.96 (dd, *J* = 7.8 Hz, 1.2 Hz, 1H), 7.67 - 7.78 (m, 3H), 7.51 - 7.57 (m, 1H), 7.35 (dd, *J* = 8.3 Hz, 4.7 Hz, 1H), 7.19 - 7.21 (m, 3H), 7.12 (s, 1H); $^{13}C$ NMR (75 MHz, $CDCl_3$) δ 148.83, 148.01, 145.14, 138.60, 138.53, 135.60, 135.45, 135.36, 134.39, 130.49, 129.87, 129.64, 129.08, 128.36, 128.34, 127.79, 124.96; HRMS (ESI $[M+H]^+$): for $C_{19}H_{13}NO_3S$ calcd. 336.0694; found 336.0687.

4-fluoro-2-((4-methoxyphenyl)ethynyl)phenyl 2-bromobenzenesulfonate, **2u** (200 mg, 0.43 mmol) was dissolved in DMF (2 mL) followed by addition of $Ni(PPh_3)_4$ (24 mg, 0.022 mmol), HCOONa (88 mg, 1.30 mmol) and distilled water (1 mL) and heated under continuous stirring at 100 °C for 10 h. Upon completion of the reaction, as monitored by TLC, the reaction mixture was cooled. The reaction mixture was diluted with EtOAc (40 mL), washed with water (2 x 40 mL), dried over $MgSO_4$ and concentrated under reduced pressure. The crude product was subjected to column chromatography on silica gel with petrolium ether - EtOAc (19:1) as eluent to give pure **3u**.

**(*E*)-2-fluoro-11-(4-methoxybenzylidene)-11*H*-dibenzo[*c*,*f*][1,2]oxathiepine 6,6-dioxide (3u):**
Brown gummy; yield = 79% (132 mg); IR (KBr): 2922, 1597 $cm^{-1}$; $^1H$ NMR (400 MHz, $CDCl_3$) δ 7.91 (d, *J* = 8.0 Hz, 1H), 7.60 - 7.65 (m, 2H), 7.48 - 7.52 (m, 1H), 7.41 - 7.45 (m, 1H), 7.02 - 7.09 (m, 3H), 6.99 (s, 1H), 6.83 - 6.87 (m, 1H), 6.74 - 6.77 (m, 2H), 3.78 (s, 3H); $^{13}C$ NMR (75 MHz, $CDCl_3$) δ 162.49, 159.74, 159.20, 143.54, 143.50, 139.21, 136.09, 135.91, 134.30, 134.18, 133.68, 132.02, 132.00, 131.04, 129.29, 129.00, 128.14, 127.42, 124.78, 124.66, 117.40, 117.11, 117.08, 116.79, 114.06, 55.36; HRMS (ESI [M]): for $C_{21}H_{15}FO_4S$ calcd. 382.0675; found 382.0677.

2-ethynylphenyl 2-bromobenzenesulfonate, **2v** (200 mg, 0.59 mmol) was dissolved in DMF (2 mL) followed by addition of $Ni(PPh_3)_4$ (33 mg, 0.029 mmol), HCOONa (121 mg, 1.78 mmol) and distilled water (1 mL) and heated under continuous stirring at 100 °C for 12 h. Upon completion of the reaction, as monitored by TLC, the reaction mixture was cooled. The reaction mixture was diluted with EtOAc (40 mL), washed with water (2 x 40 mL), dried over $MgSO_4$ and concentrated under reduced pressure. The crude product was subjected to

column chromatography on silica gel with petrolium ether - EtOAc (19:1) as eluent to give pure **3v**.

**11-methylene-11*H*-dibenzo[*c*,*f*][1,2]oxathiepine 6,6-dioxide (3v):**

Brown gummy; yield = 75% (115 mg); IR (KBr): 2987, 2930, 2227, 1597 $cm^{-1}$; $^1H$ NMR (400 MHz, $CDCl_3$) δ 8.04 (d, *J* = 8.0 Hz, 1H), 7.48 - 7.53 (m, 2H), 7.29 - 7.38 (m, 4H), 7.15 - 7.23 (m, 2H), 7.03 (d, *J* = 12.0 Hz, 1H), 6.87 (d, *J* = 12.0 Hz, 1H); $^{13}C$ NMR (75 MHz, $CDCl_3$) δ 147.47, 135.02, 134.47, 133.15, 131.56, 131.15, 130.58, 129.88, 129.67, 129.18, 128.69, 128.13, 127.39, 122.69; HRMS (ESI $[M+Na]^+$) for $C_{14}H_{10}O_3S$ calcd. 281.0248; found 281.0490.

**5) Synthetic procedure for the access doxepin (5) and nordoxepin (6):**

(*Z*)-3-(dibenzo[*b*,*e*]oxepin-11(6*H*)-ylidene)propyl-4-methylbenzenesulfonate, **4** (200 mg, 0.49 mmol) was dissolved in THF (5 ml ) followed by addition of $Me_2NH$ (44 mg, 0.98 mmol), $K_2CO_3$ (204 mg, 1.47 mmol), NaI (74 mg, 0.49 mmol) and heated under continuous stirring at 60 °C for 12 h. Upon completion of the reaction, as monitored by TLC, the reaction mixture was cooled. The reaction mixture was diluted with EtOAc (40 mL), washed with water (2 x 40 mL), dried over $MgSO_4$ and concentrated under reduced pressure. The crude product was subjected to column chromatography on silica gel with petrolium ether - EtOAc (19:1) as eluent to give pure **5**.

**(*Z*)-3-(dibenzo[*b*,*e*]oxepin-11(6*H*)-ylidene)-*N*,*N*-dimethylpropan-1-amine (5):**

White solid; m.p. 155 - 157 °C; yield = 94% (137 mg); IR (KBr): 3016, 2965, 1359, 1172 $cm^{-1}$; $^1H$ NMR (300 MHz, $CDCl_3$) δ 7.23 - 7.34 (m, 4H), 7.14 (td, *J* = 7.1 Hz, 1.8 Hz, 2H), 6.84 - 6.91 (m, 2H), 5.70 (t, *J* = 7.1 Hz, 1H), 5.21 (s, 1H), 2.58 - 2.65 (m, 2H), 2.47 - 2.52 (m, 2H), 2.26 (s, 6H); $^{13}C$ NMR (75 MHz, $CDCl_3$) δ 155.60, 145.72, 140.38, 133.85, 131.38, 130.21, 129.36, 129.16, 127.60, 127.55, 126.40, 124.21, 120.60, 119.73, 70.57, 59.44, 45.32, 27.99; HRMS (ESI $[M+H]^+$): for $C_{19}H_{21}NO$ calcd. 280.1701; found 280.1702.

(*Z*)-3-(dibenzo[*b*,*e*]oxepin-11(6*H*)-ylidene)propyl-4-methylbenzenesulfonate, **4** (200 mg, 0.49 mmol) was dissolved in THF (5 ml) followed by addition of $MeNH_2$ (30 mg, 0.98 mmol), $K_2CO_3$ (204 mg, 1.47 mmol), NaI (74 mg, 0.49 mmol) and heated under continuous stirring at 60 °C for 12 h. Upon completion of the reaction, as monitored by TLC, the reaction mixture was cooled. The reaction mixture was diluted with EtOAc (40 mL), washed with water (2 x 40 mL), dried over $MgSO_4$ and concentrated under reduced pressure. The crude

product was subjected to column chromatography on silica gel with petrolium ether - EtOAc (19:1) as eluent to give pure **6**.

**(*Z*)-3-(dibenzo[*b*,*e*]oxepin-11(6*H*)-ylidene)-*N*-methylpropan-1-amine (6):**

White solid; m.p. 61 - 63 °C; yield = 92% (133 mg); IR (KBr): 3025, 2969, 1364, 1178 $cm^{-1}$; $^{1}H$ NMR (300 MHz, $CDCl_3$) δ 7.24 - 7.31 (m, 4H), 7.14 (td, *J* = 7.1 Hz, 1.8 Hz, 2H), 5.21 (s, 2H), 2.72 - 2.80 (m, 2H), 2.64 (q, *J* = 7.2 Hz, 2H), 2.43 (s, 3H), 2.23 (s, 1H); $^{13}C$ NMR (75 MHz, $CDCl_3$) δ 155.64, 145.53, 141.05, 133.85, 131.41, 129.94, 129.42, 129.16, 127.66, 127.54, 126.39, 124.30, 120.71, 119.79, 70.65, 51.69, 36.05, 29.80; HRMS (ESI $[M+H]^+$): for $C_{18}H_{19}NO$ calcd. 266.1545; found 266.1552.

## 6) $^{1}$H and $^{13}$C-NMR spectra of compounds 2a-v:

**Figure S1. $^{1}$H NMR (400 MHz) and $^{13}$C NMR (100 MHz) spectra of compound 1-bromo-2-((2-(phenylethynyl)phenoxy)methyl)benzene in (2a) in $CDCl_3$.**

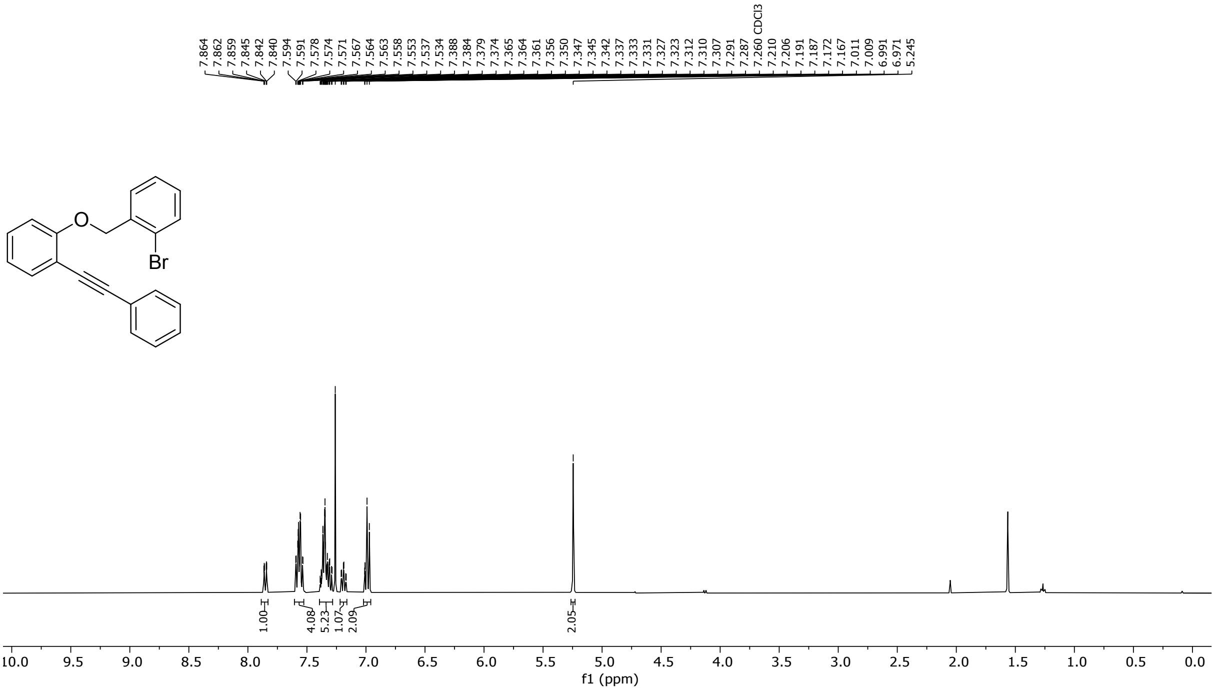


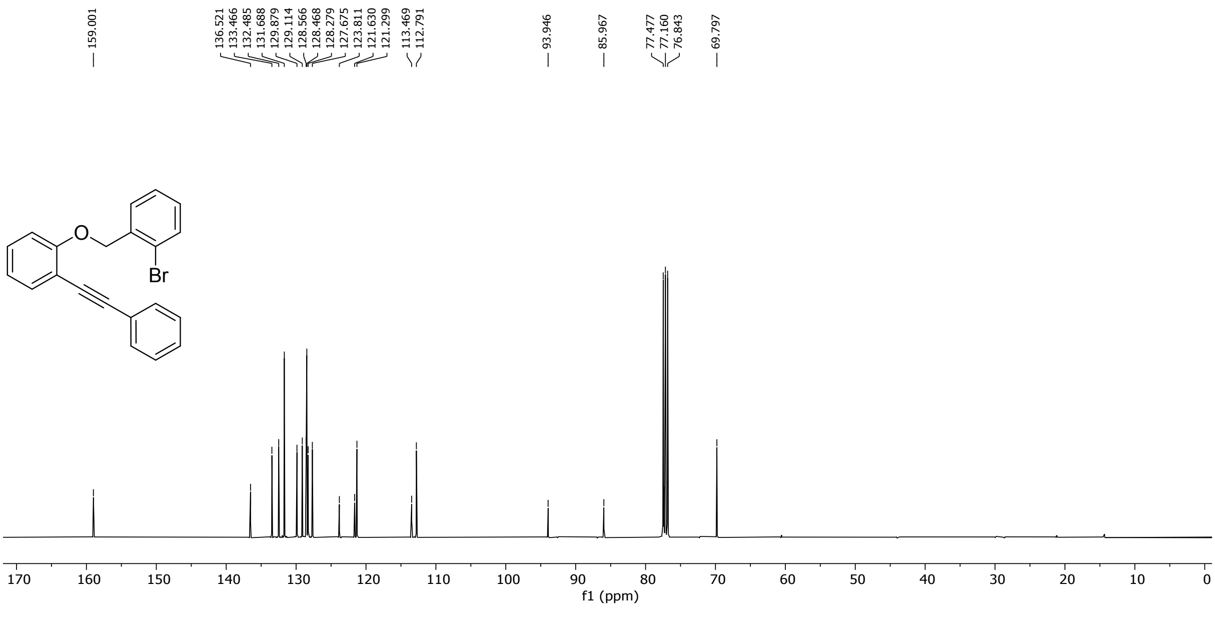

**Figure S2.** $^{1}H$ **NMR (400 MHz) and** $^{13}C$ **NMR (100 MHz) spectra of compound 1-bromo-2-((2-((4-methoxyphenyl)ethynyl)phenoxy)methyl)benzene (2b) in** $CDCl_3$**.**

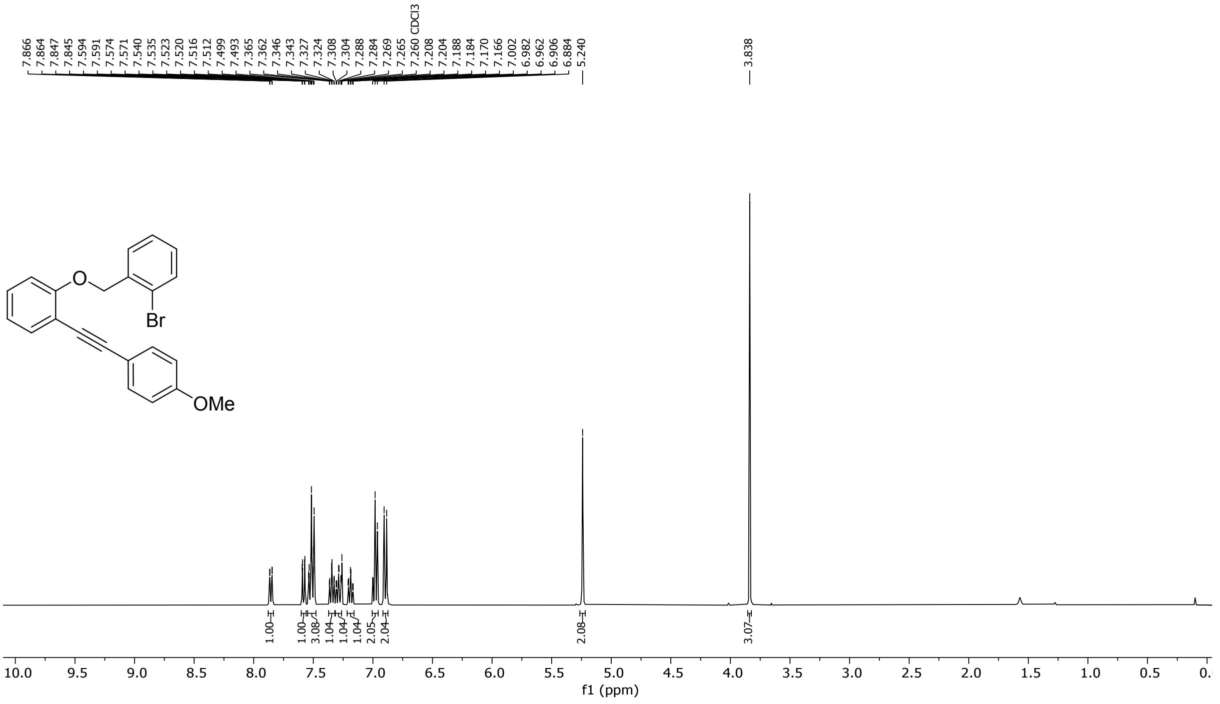


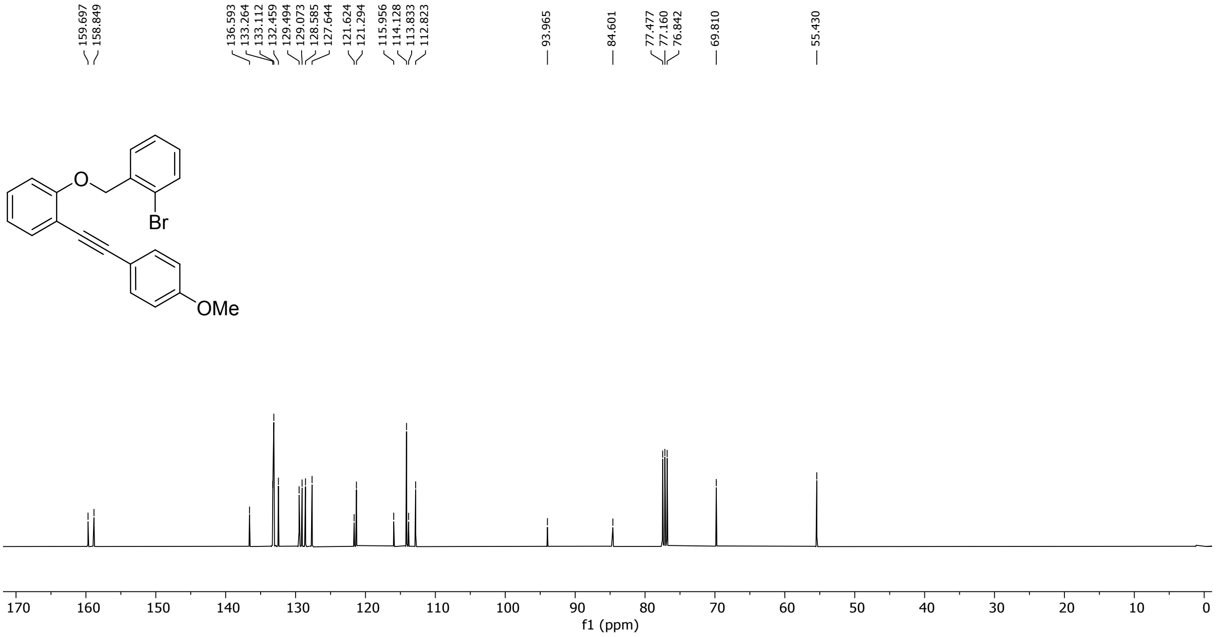

**Figure S3.** $^{1}H$ **NMR (400 MHz) and** $^{13}C$ **NMR (100 MHz) spectra of compound 1-bromo-2-((2-(p-tolylethynyl)phenoxy)methyl)benzene (2c) in** $CDCl_3$.

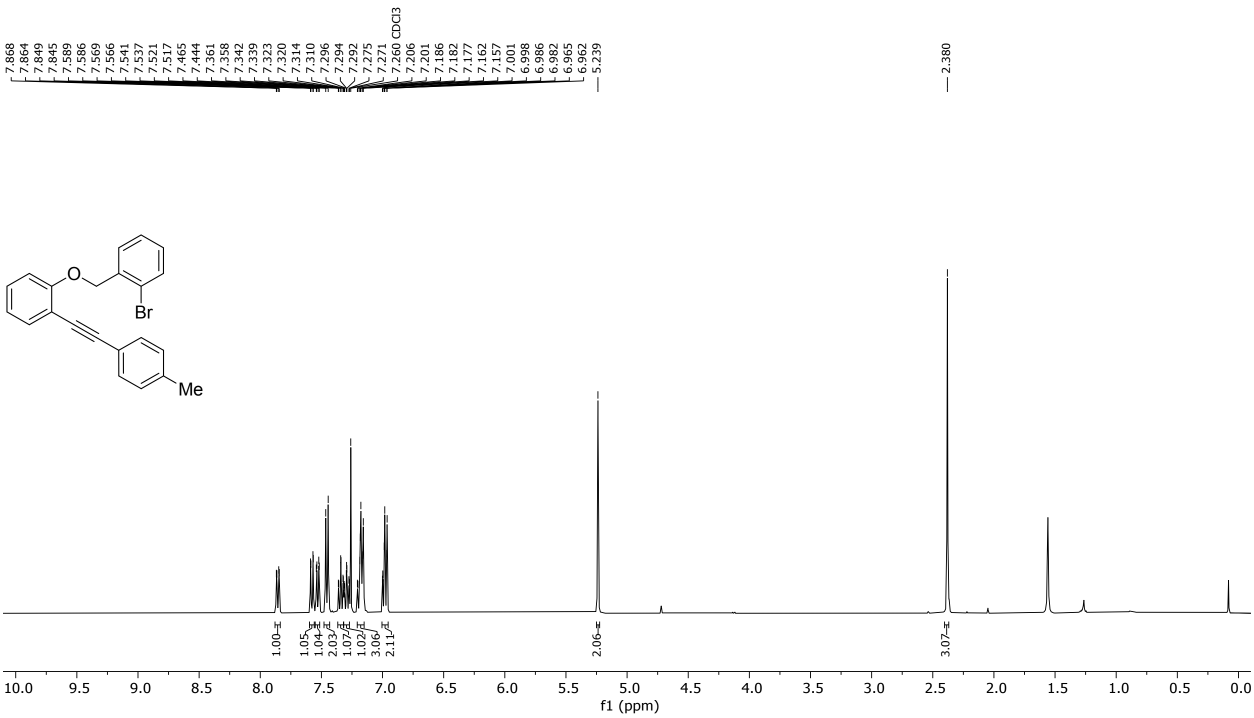


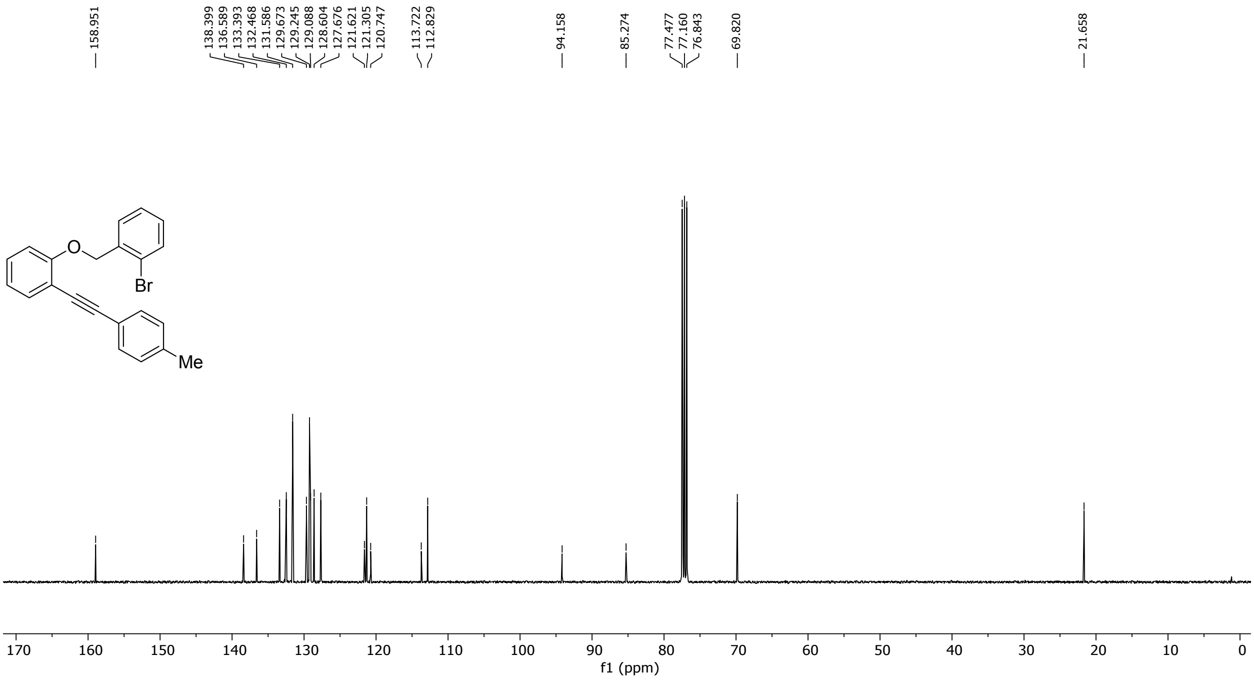

**Figure S4. $^1$H NMR (400 MHz) and $^{13}$C NMR (100 MHz) spectra of compound 1-bromo-2-((2-(hept-1-yn-1-yl)phenoxy)methyl)benzene (2d) in $CDCl_3$.**

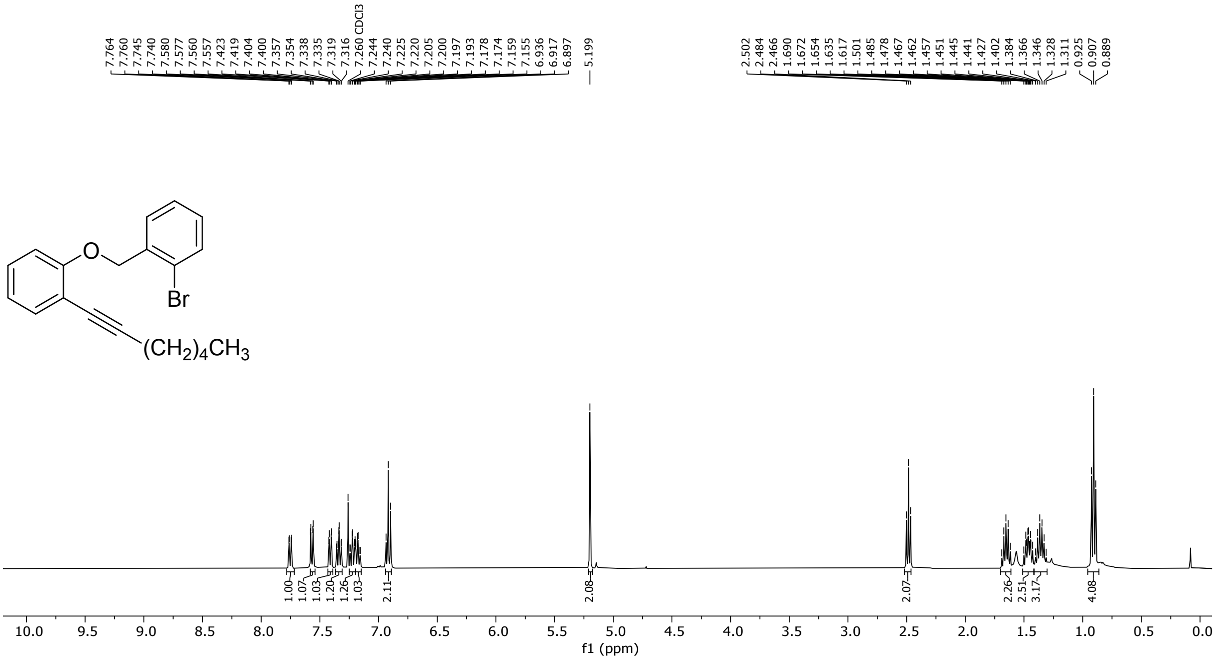

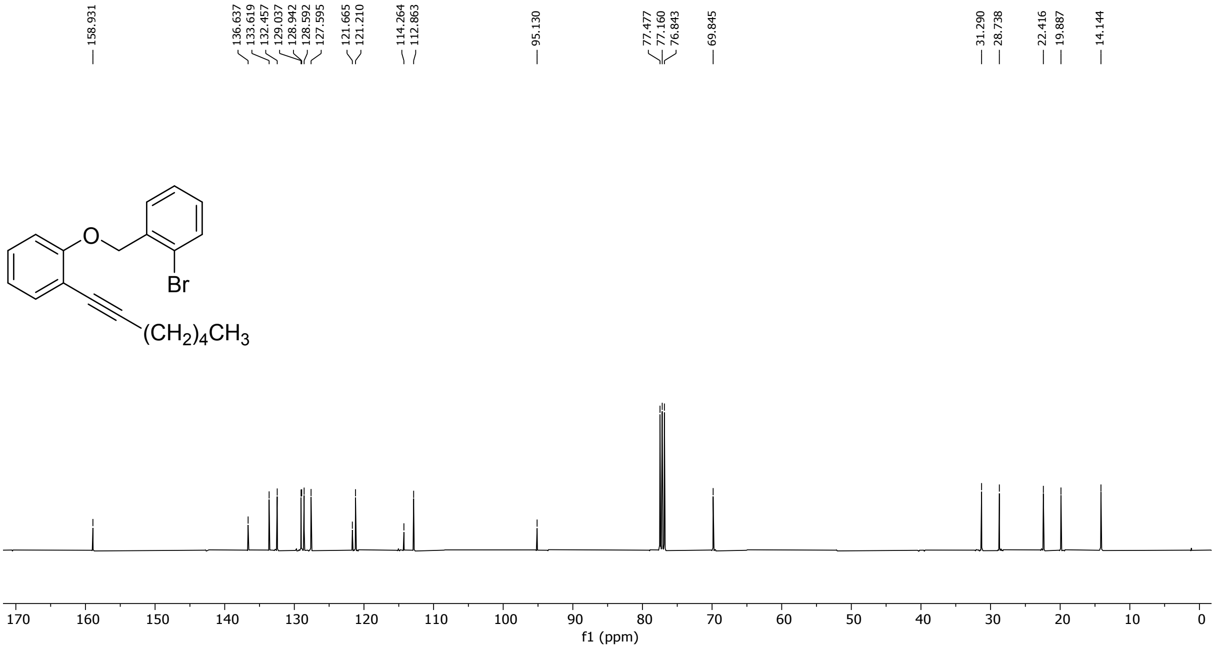

**Figure S5. $^{1}$H NMR (300 MHz) and $^{13}$C NMR (75 MHz) spectra of compound 1-bromo-2-((2-(hex-1-yn-1-yl)phenoxy)methyl)benzene (2e) in $CDCl_3$.**

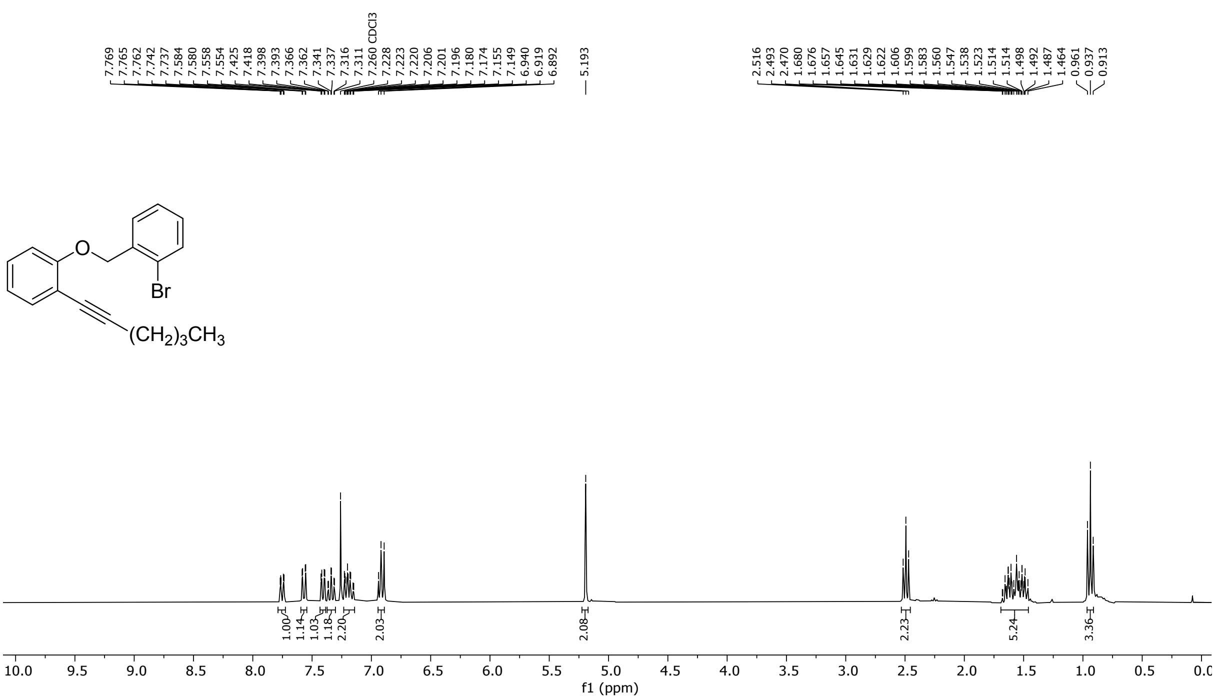


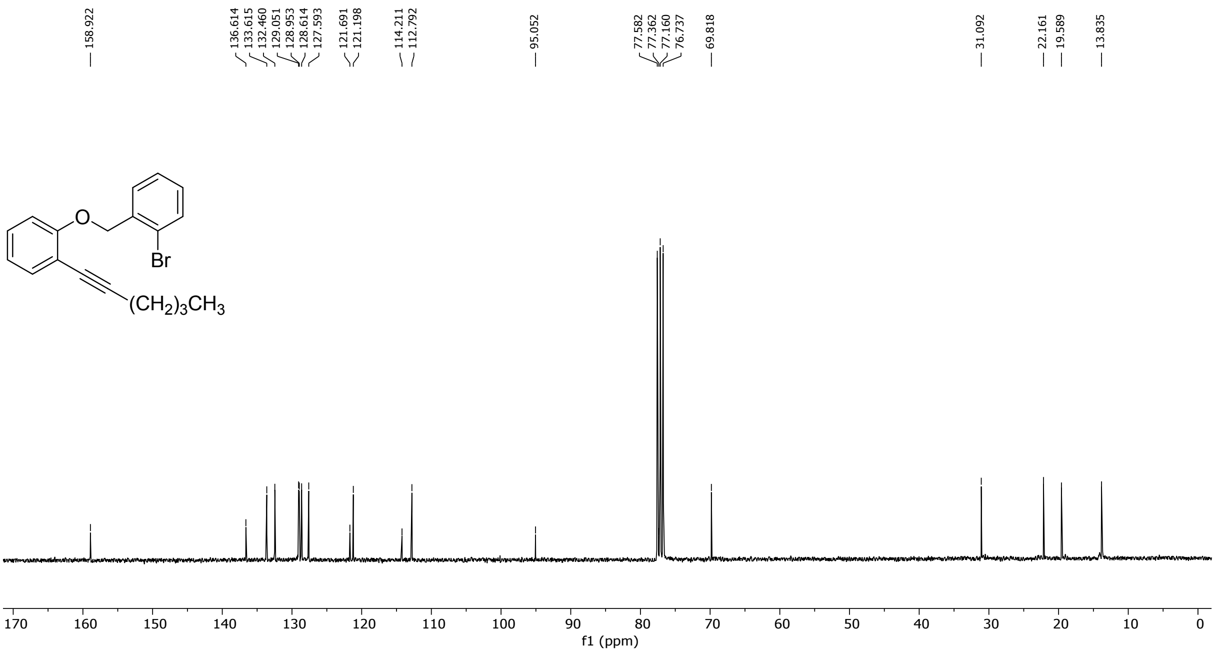

**Figure S6. $^1$H NMR (300 MHz) and $^{13}$C NMR (75 MHz) spectra of compound 4-(2-((2-bromobenzyl)oxy)phenyl)but-3-yn-1-ol (2f) in $CDCl_3$.**

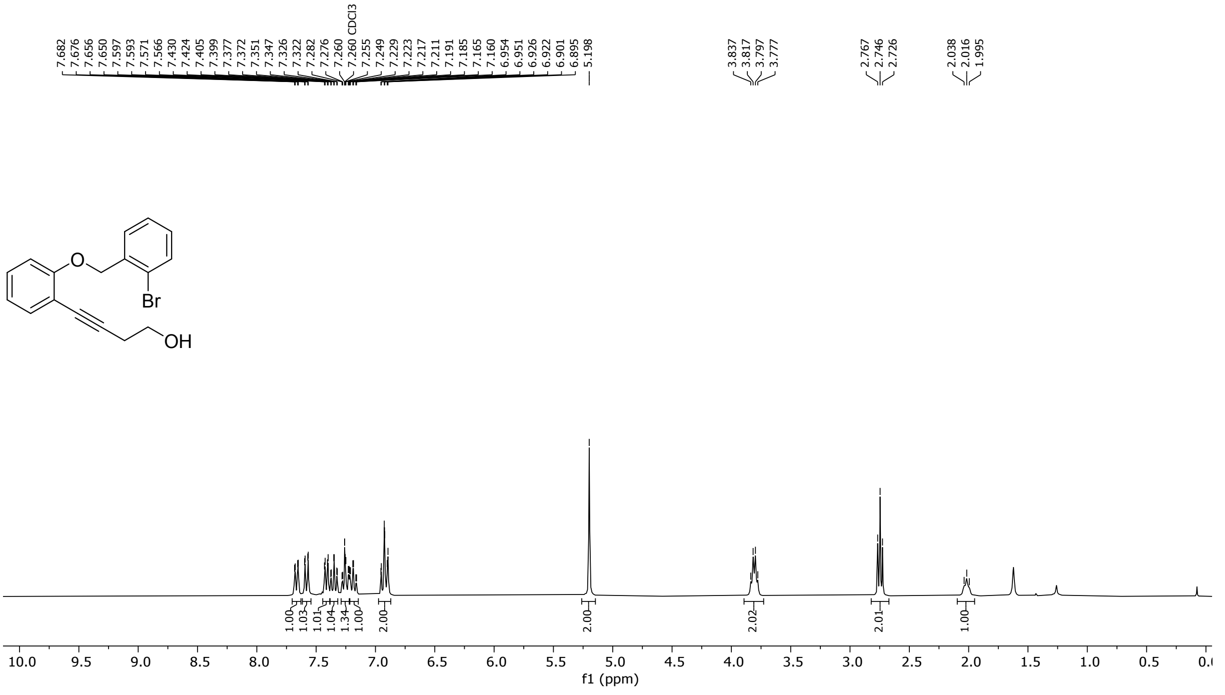


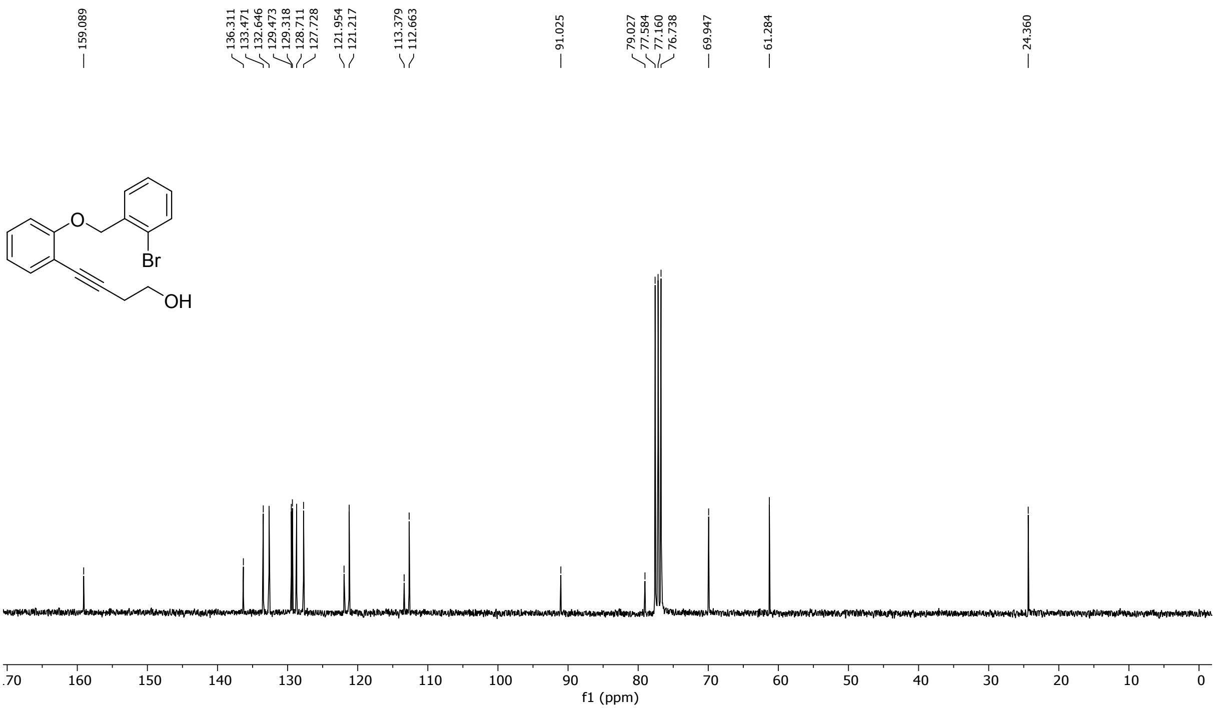

**Figure S7. $^{1}$H NMR (300 MHz) and $^{13}$C NMR (75 MHz) spectra of compound 1-bromo-4-methoxy-2-((2-(oct-1-yn-1-yl)phenoxy)methyl)benzene (2g) in $CDCl_3$.**

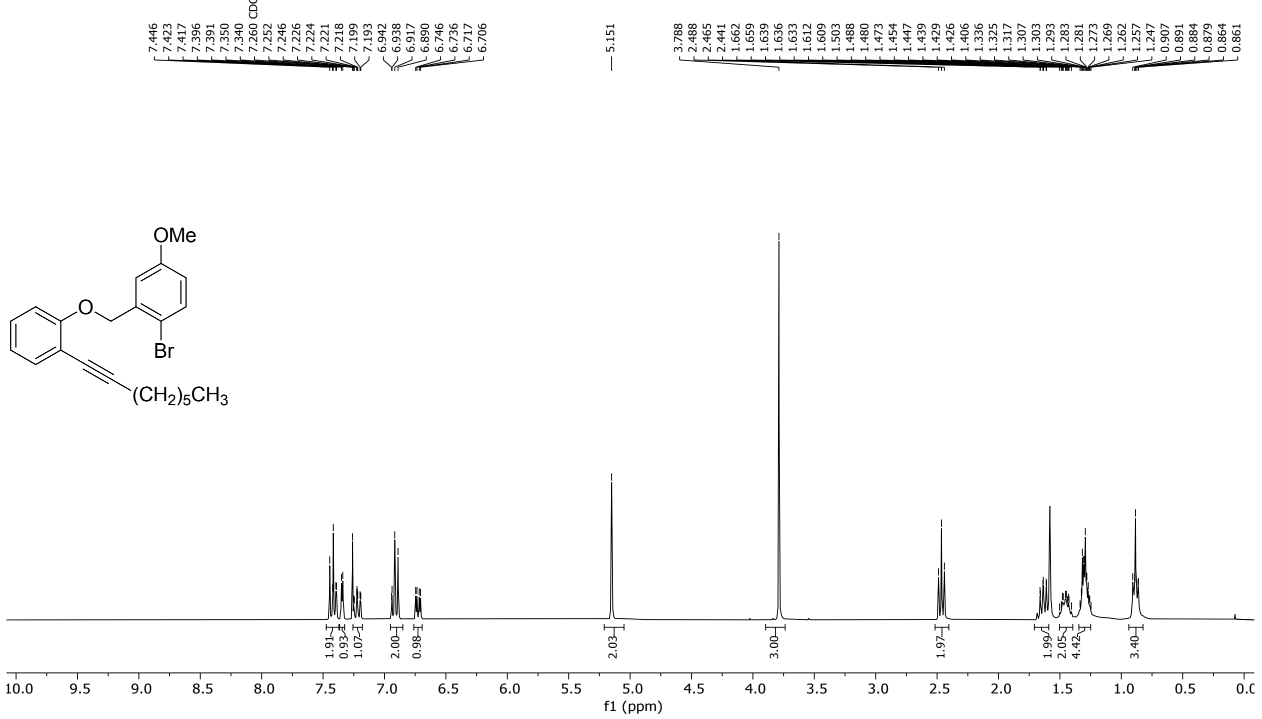

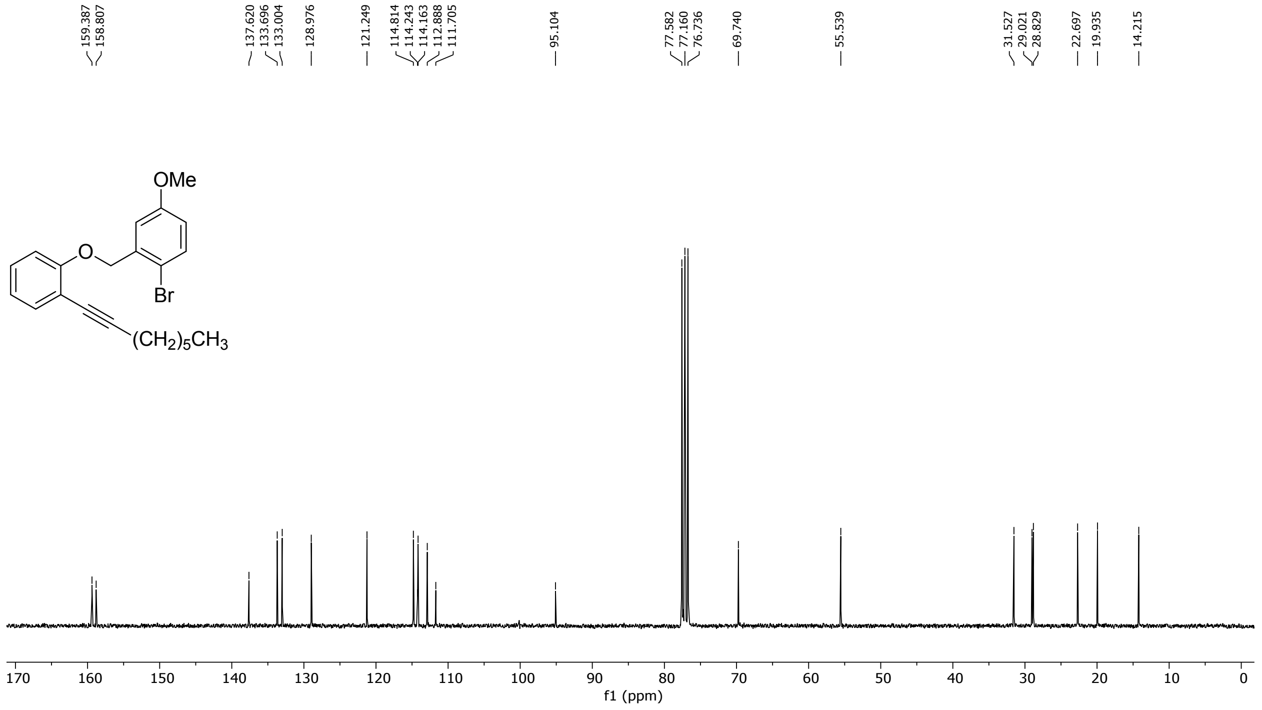

**Figure S8. $^{1}$H NMR (300 MHz) and $^{13}$C NMR (75 MHz) spectra of compound 4-(2-((2-bromo-5-methoxybenzyl)oxy)phenyl)-2-methylbut-3-yn-2-ol (2h) in $CDCl_3$.**

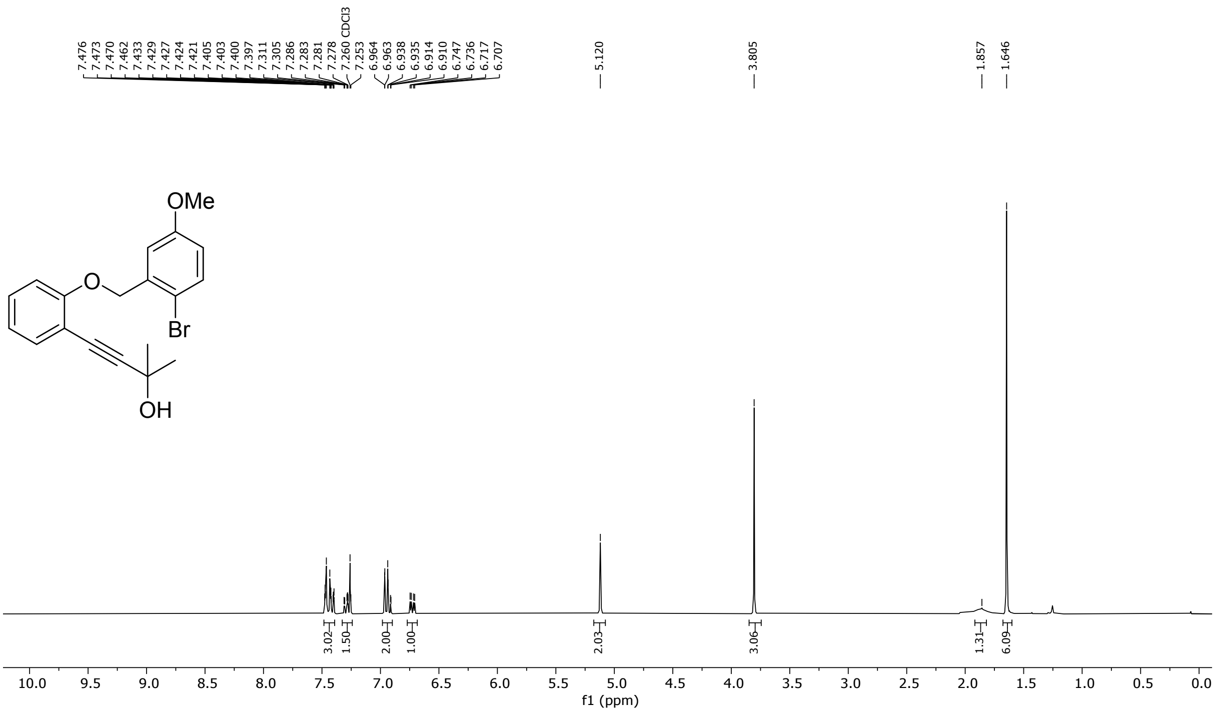

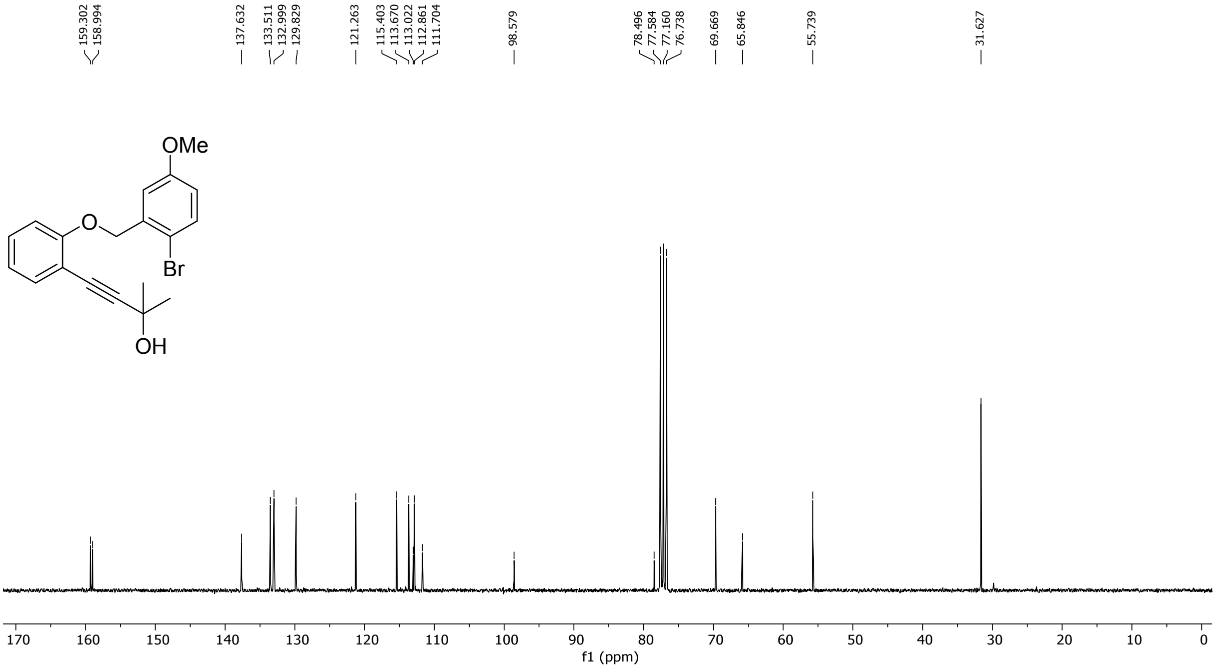

**Figure S9.** $^{1}H$ **NMR (400 MHz) and** $^{13}C$ **NMR (100 MHz) spectra of compound 1-bromo-4-methoxy-2-((2-(pent-1-yn-1-yl)phenoxy)methyl)benzene (2i) in** $CDCl_3$**.**

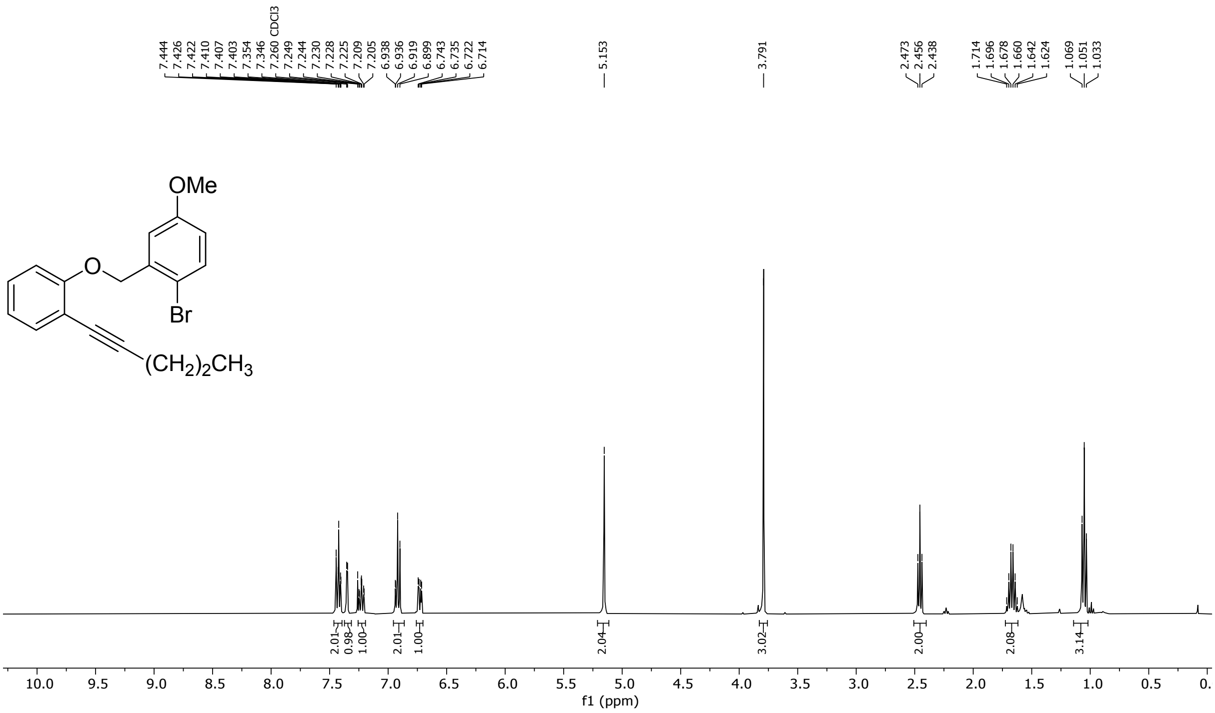


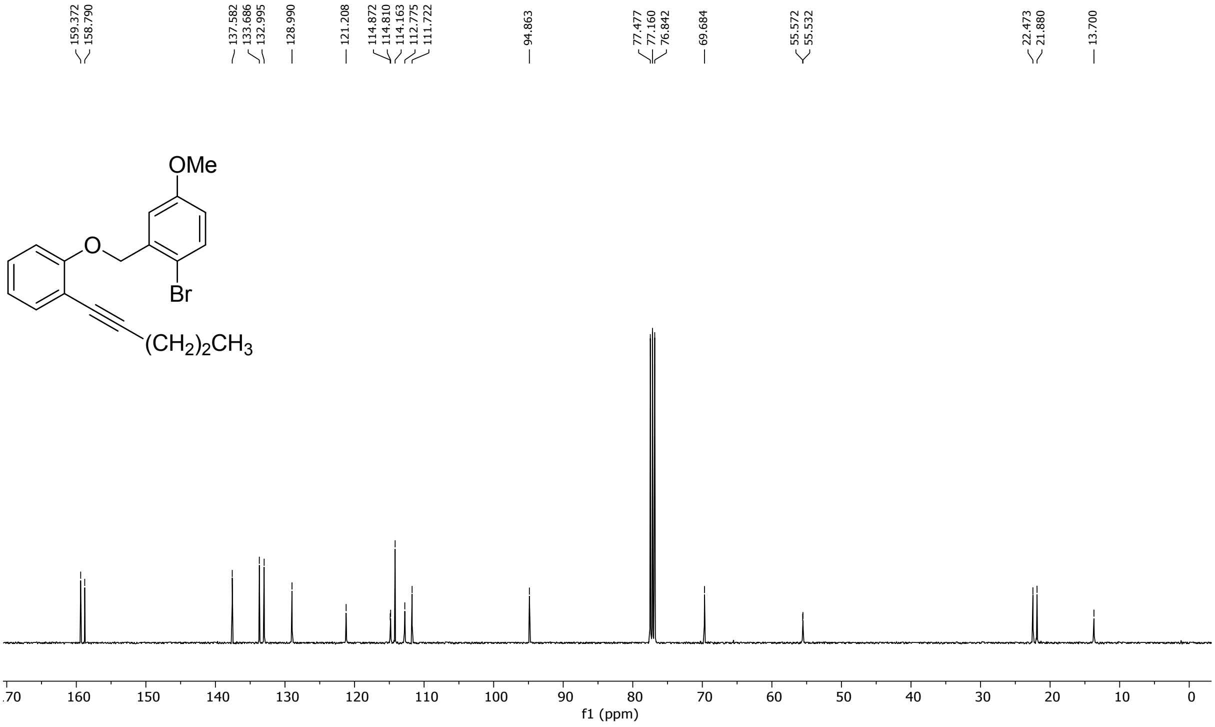

**Figure S10. $^{1}$H NMR (300 MHz) and $^{13}$C NMR (75 MHz) spectra of compound 4-(3-((2-bromobenzyl)oxy)pyridin-2-yl)-2-methylbut-3-yn-2-ol (2j) in $CDCl_3$.**

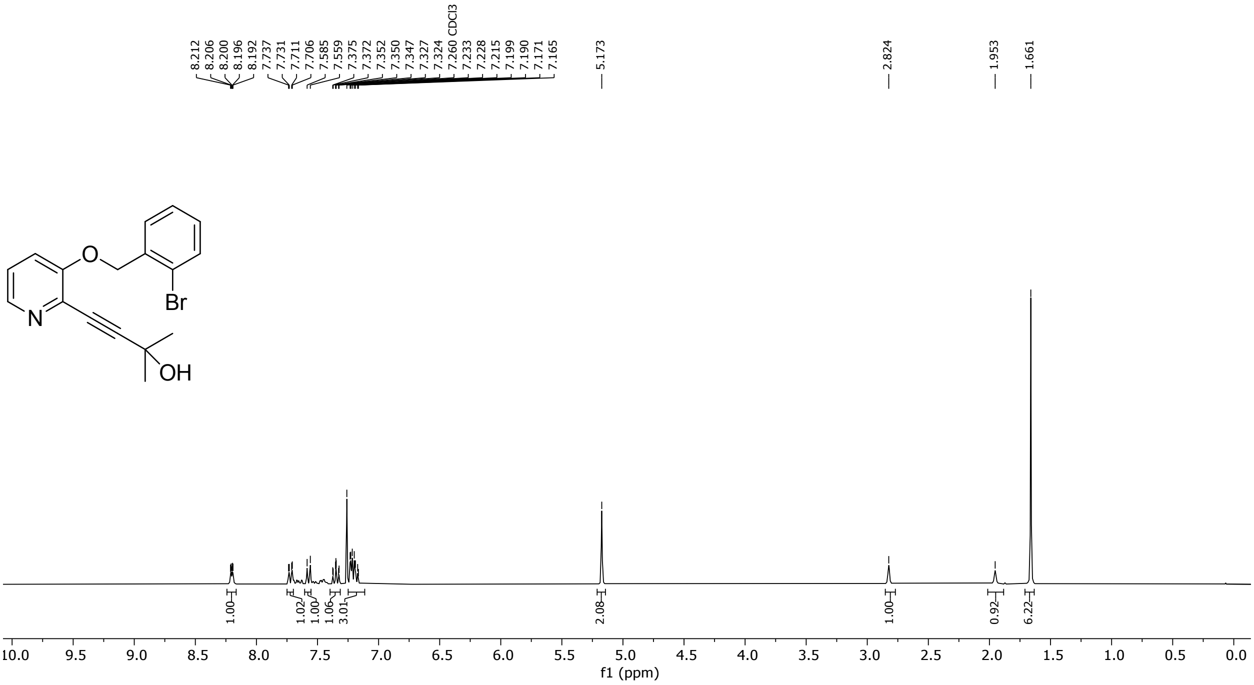


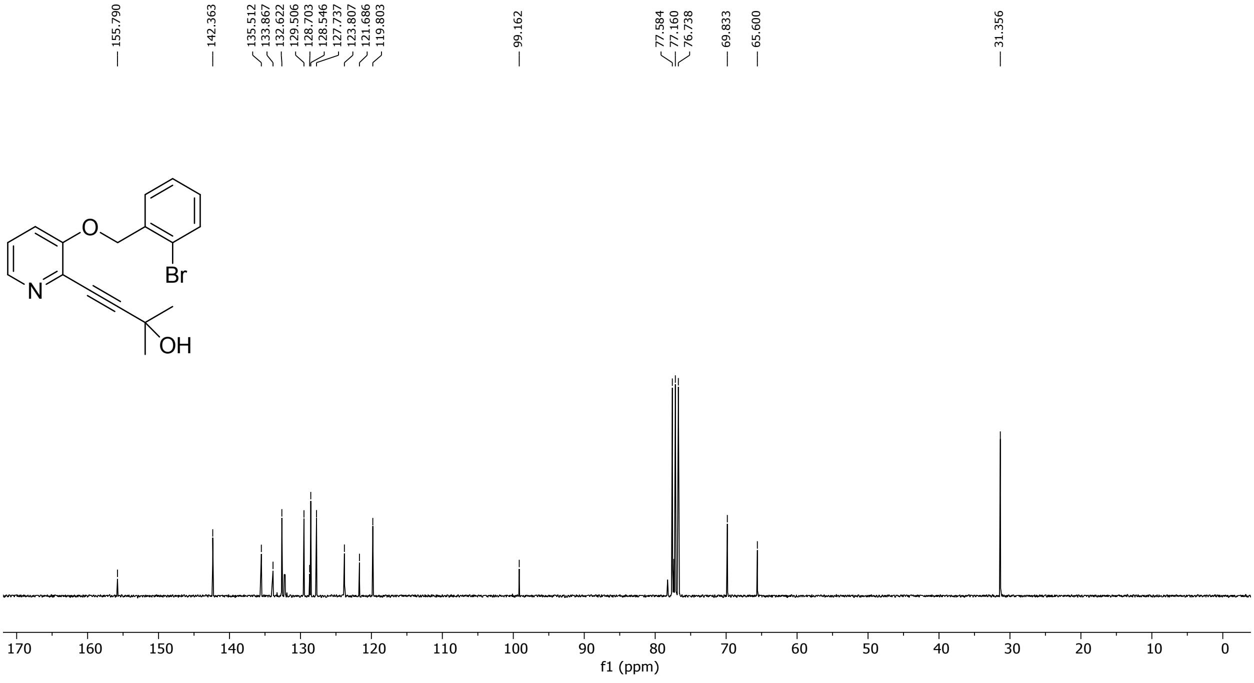

**Figure S11. ¹H NMR (300 MHz) and ¹³C NMR (75 MHz) spectra of compound 2-(phenylethynyl)phenyl 2-bromobenzenesulfonate (2k) in $CDCl_3$.**

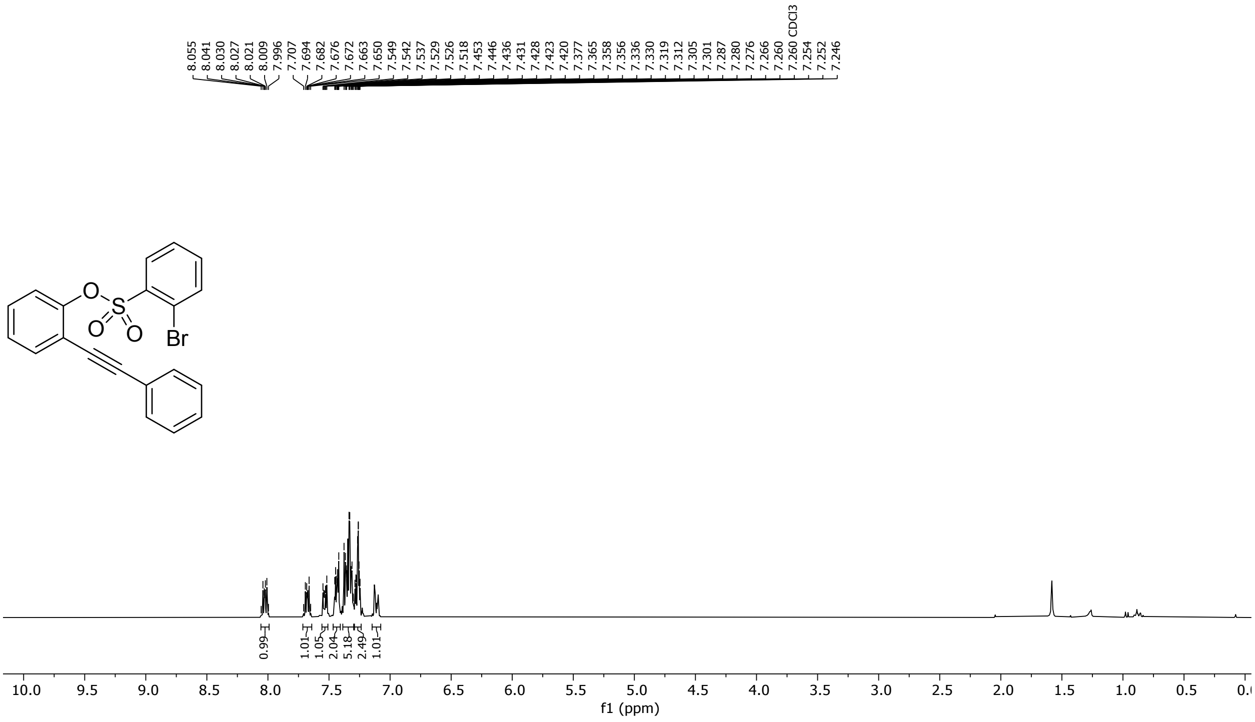


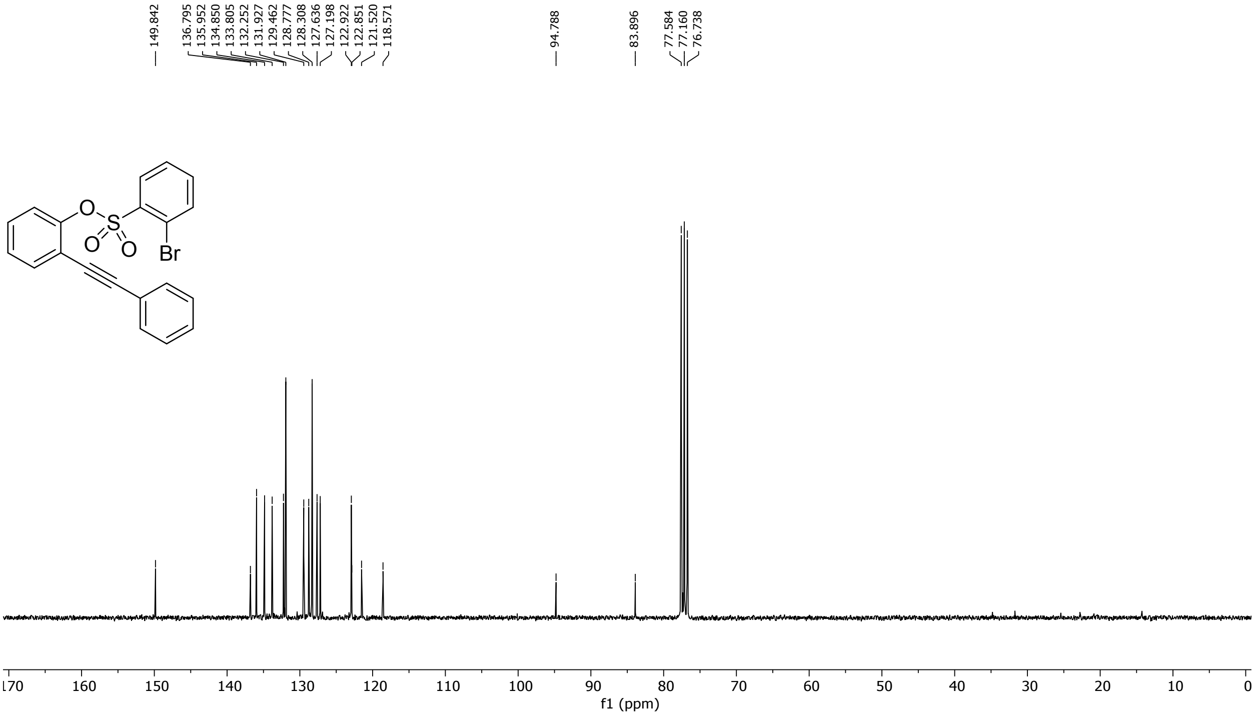

**Figure S12. $^{1}$H NMR (300 MHz) and $^{13}$C NMR (75 MHz) spectra of compound 2-(oct-1-yn-1-yl)phenyl 2-bromobenzenesulfonate (2l) in $CDCl_3$.**

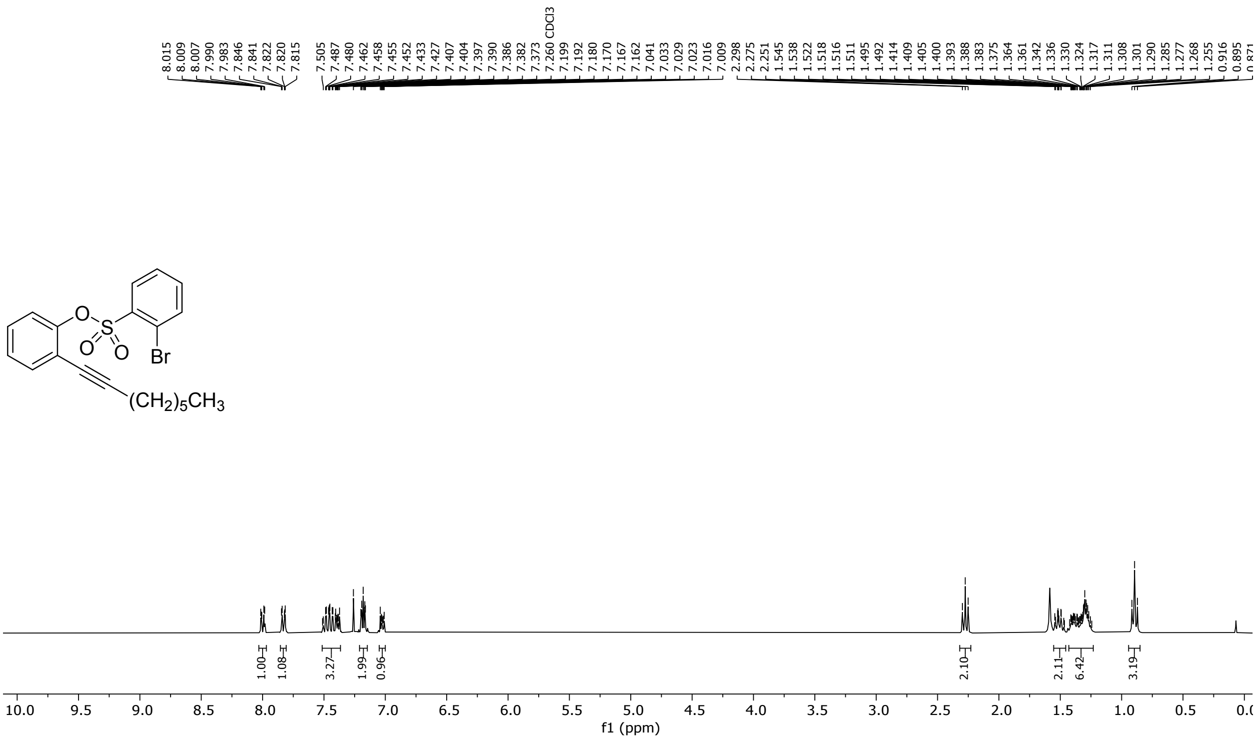


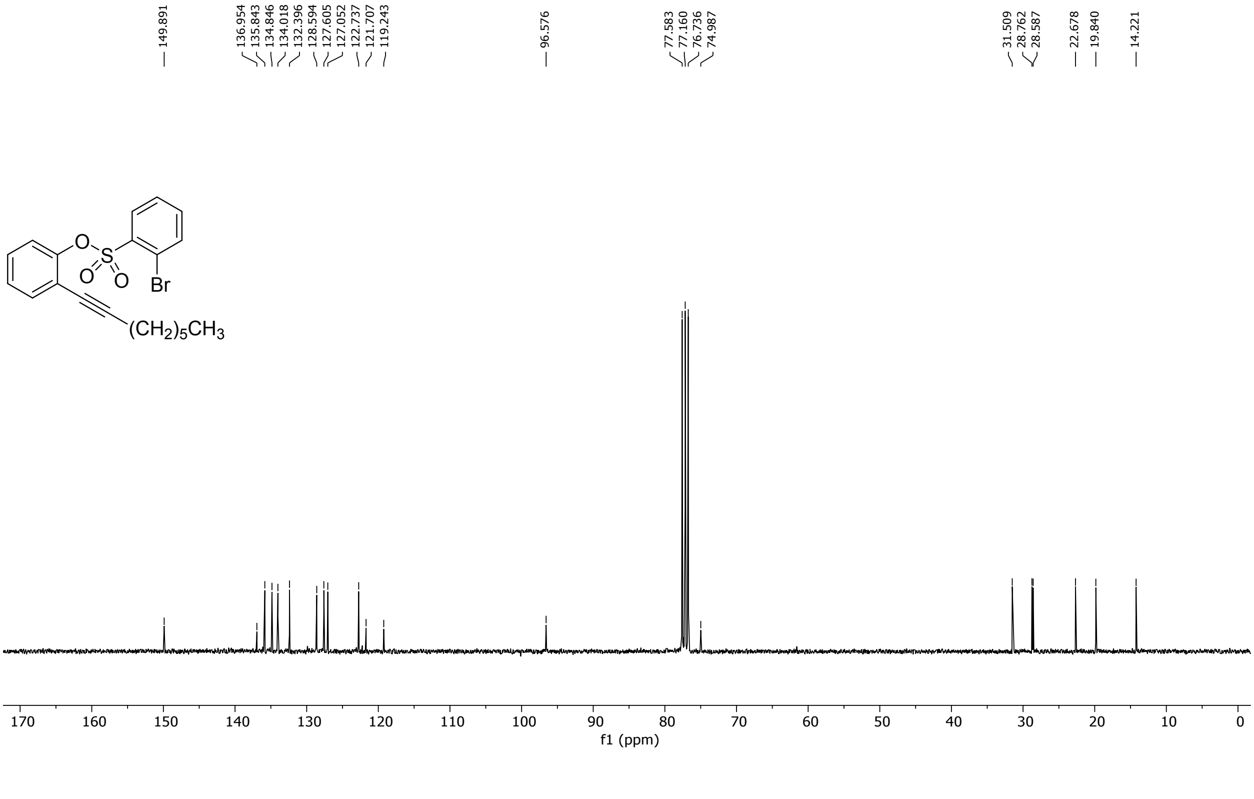

**Figure S13. $^1$H NMR (400 MHz) and $^{13}$C NMR (100 MHz) spectra of compound 2-(hept-1-yn-1-yl)phenyl 2-bromobenzenesulfonate (2m) in $CDCl_3$.**

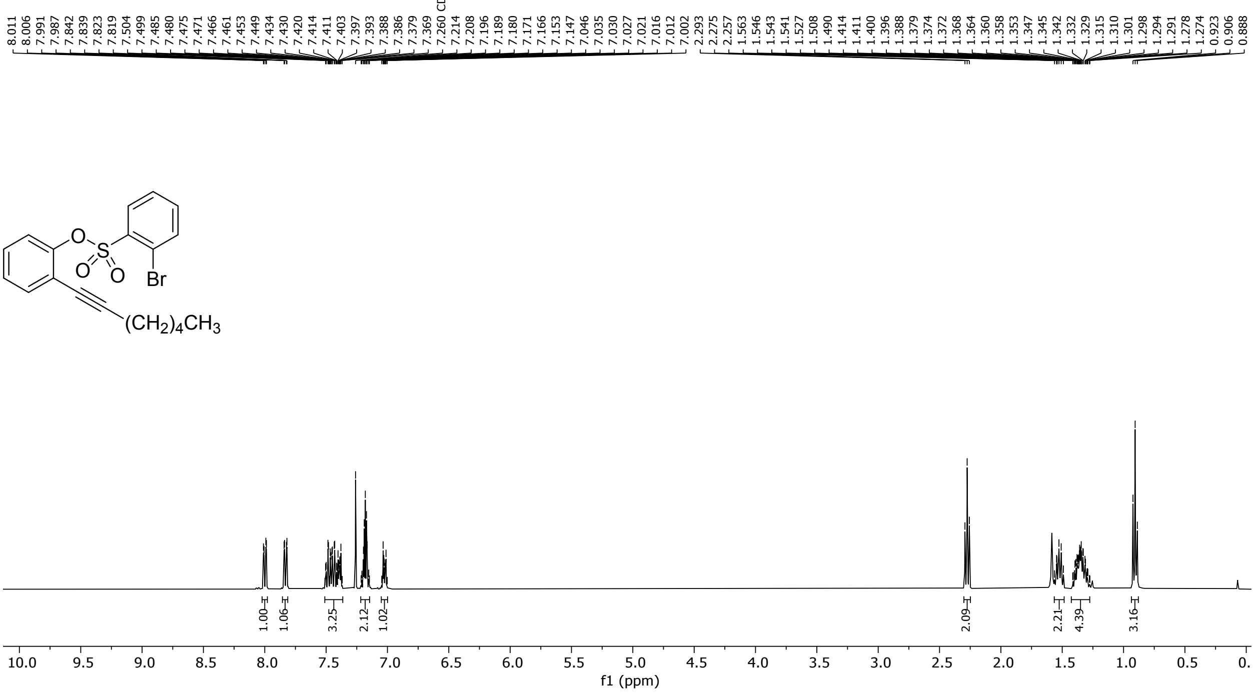


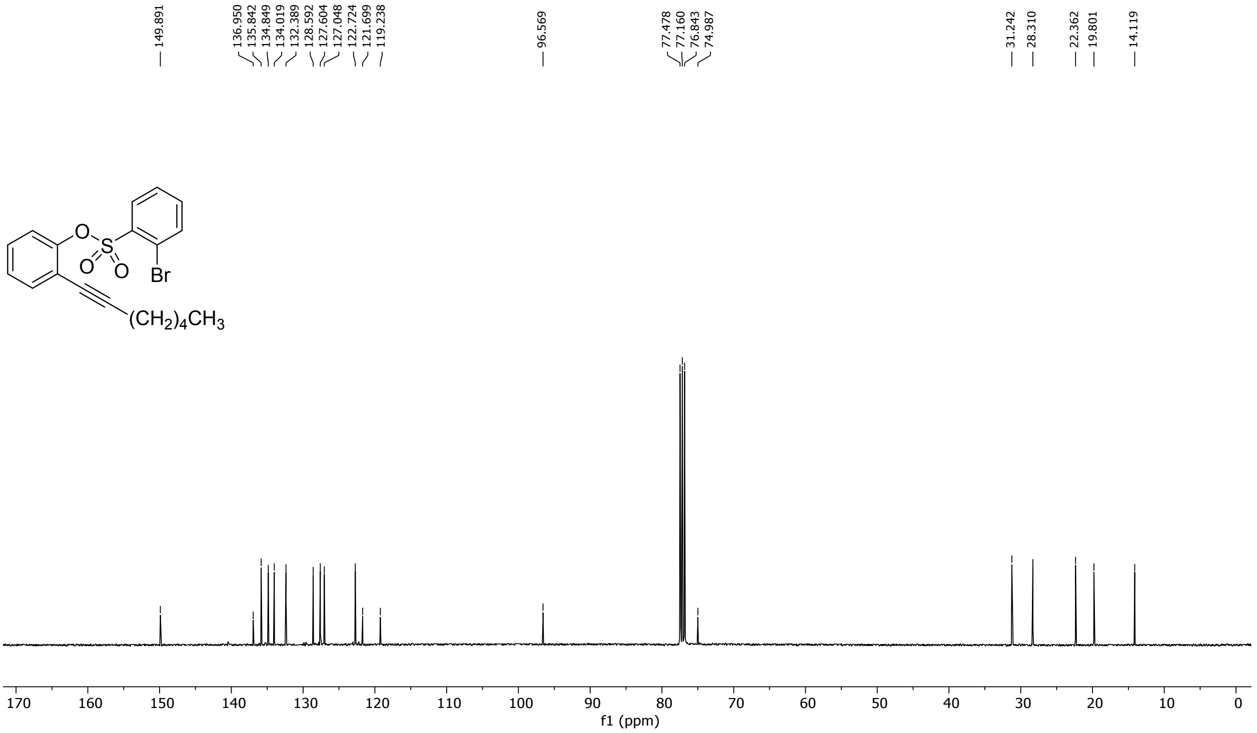

**Figure S14. $^1$H NMR (400 MHz) and $^{13}$C NMR (100 MHz) spectra of compound 2-(hex-1-yn-1-yl)phenyl 2-bromobenzenesulfonate (2n) in $CDCl_3$.**

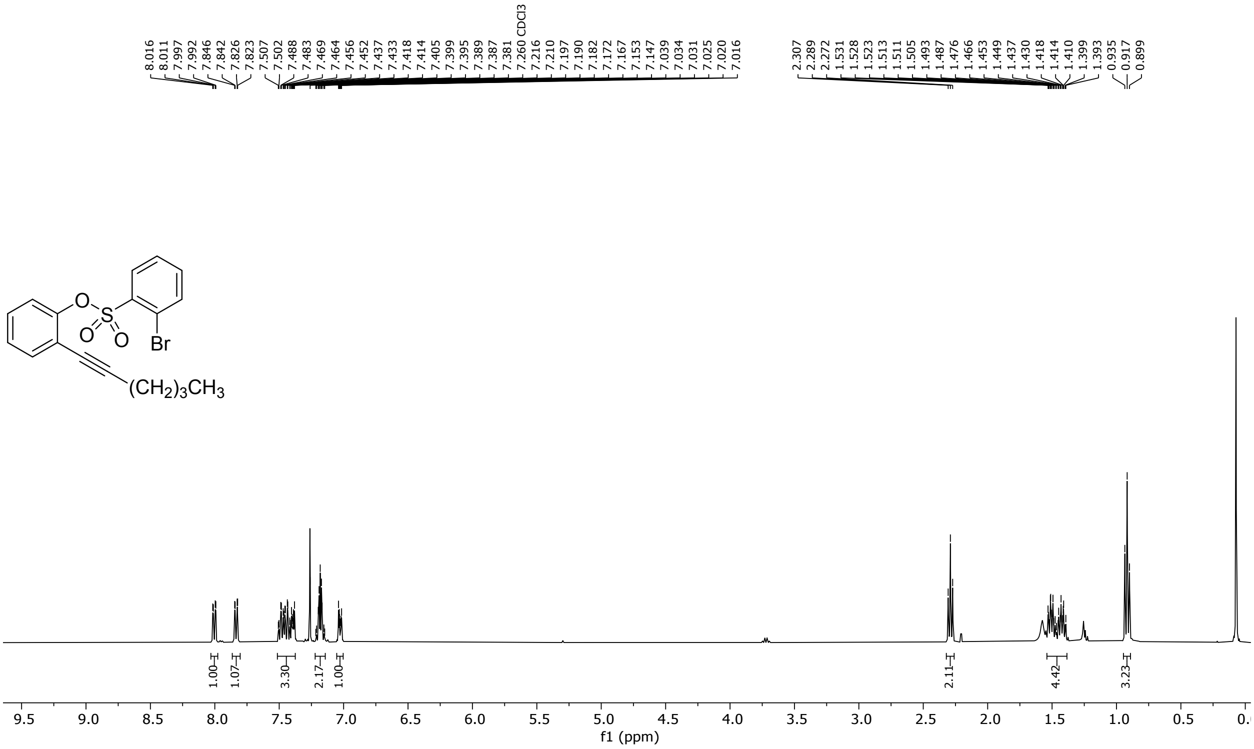

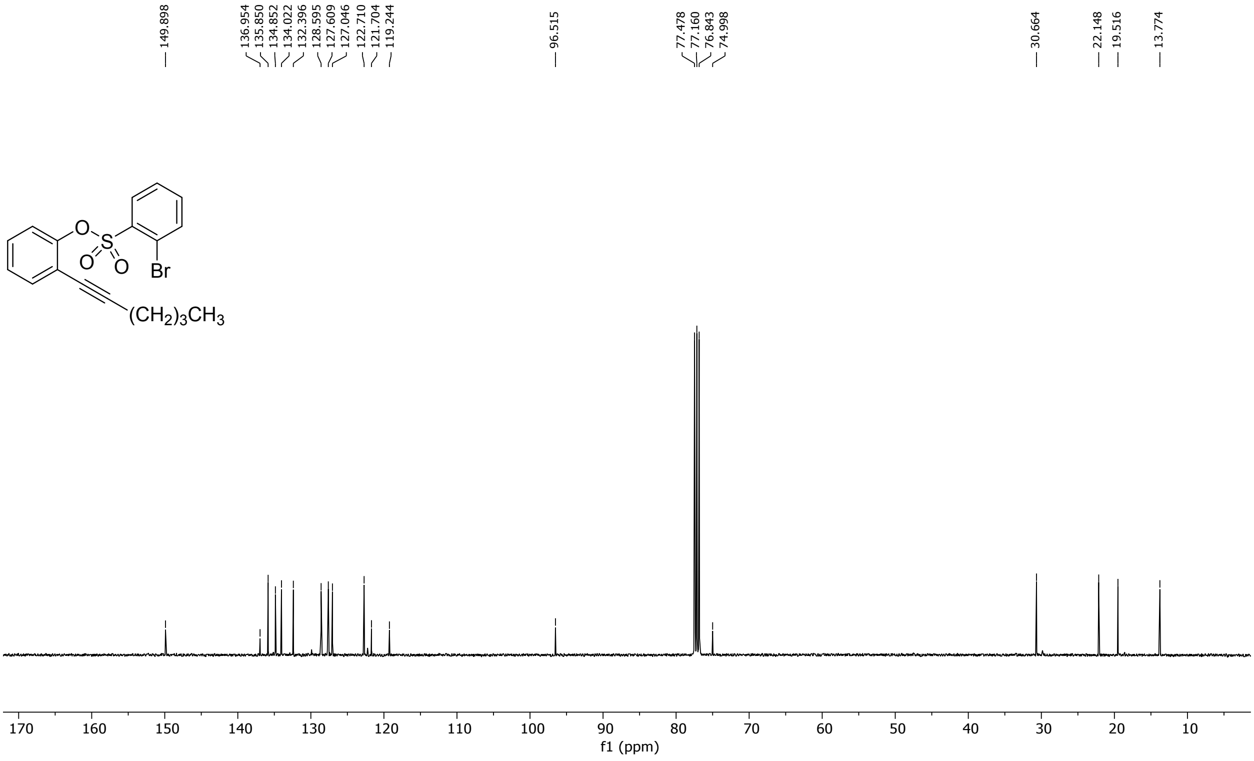

**Figure S15. $^{1}$H NMR (400 MHz) and $^{13}$C NMR (100 MHz) spectra of compound 2-(pent-1-yn-1-yl)phenyl 2-bromobenzenesulfonate (2o) in $CDCl_3$.**

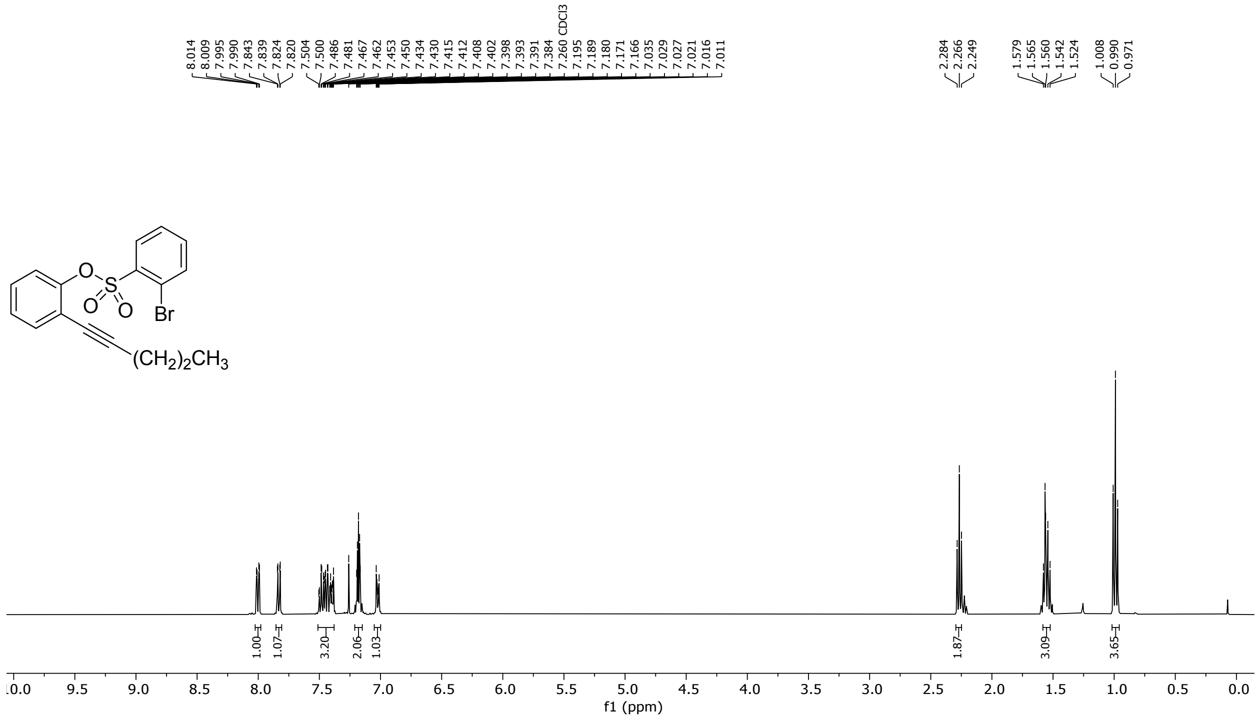

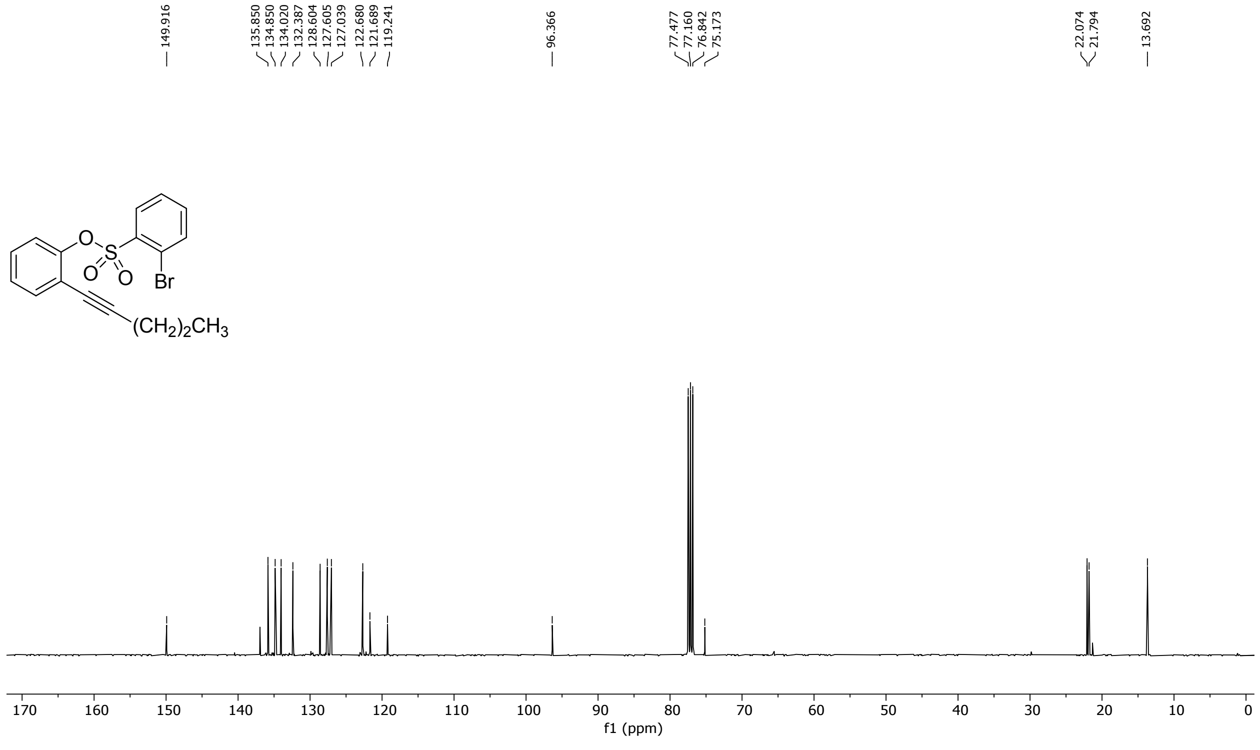

**Figure S16. $^{1}H$ NMR (300 MHz) and $^{13}C$ NMR (75 MHz) spectra of compound 2-(3-hydroxy-3-methylbut-1-yn-1-yl)phenyl 2-bromobenzenesulfonate (2p) in $CDCl_3$.**

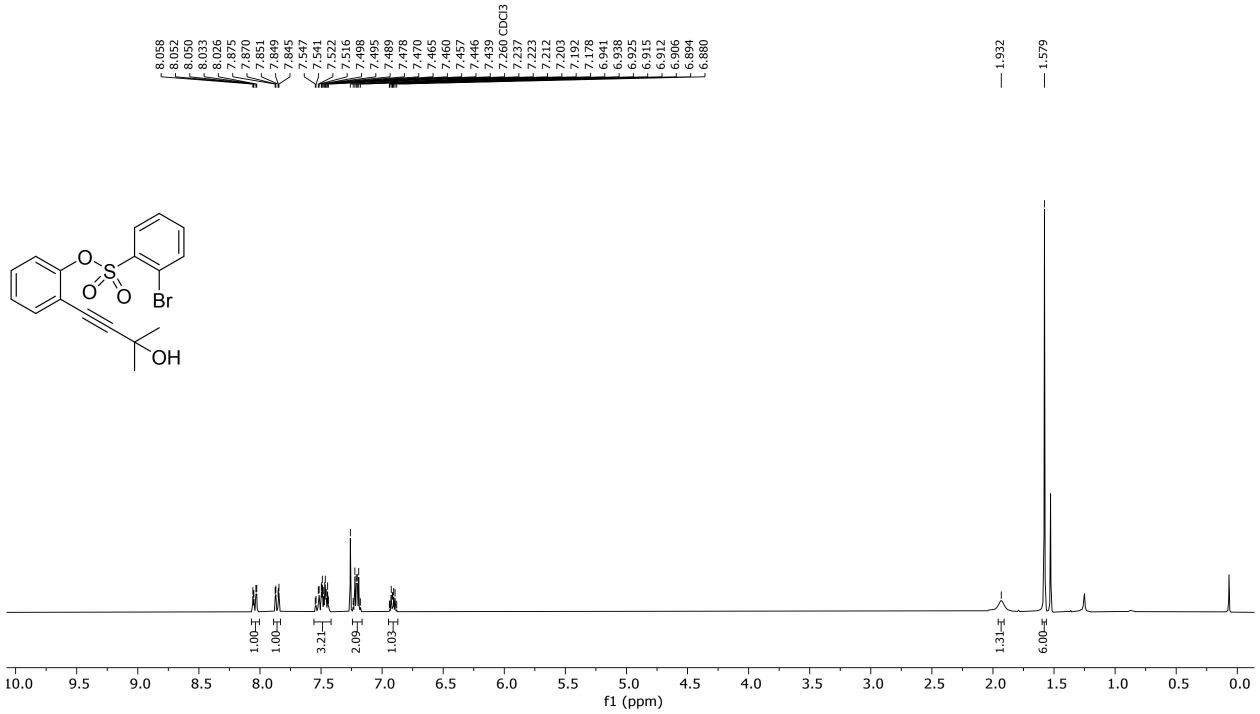


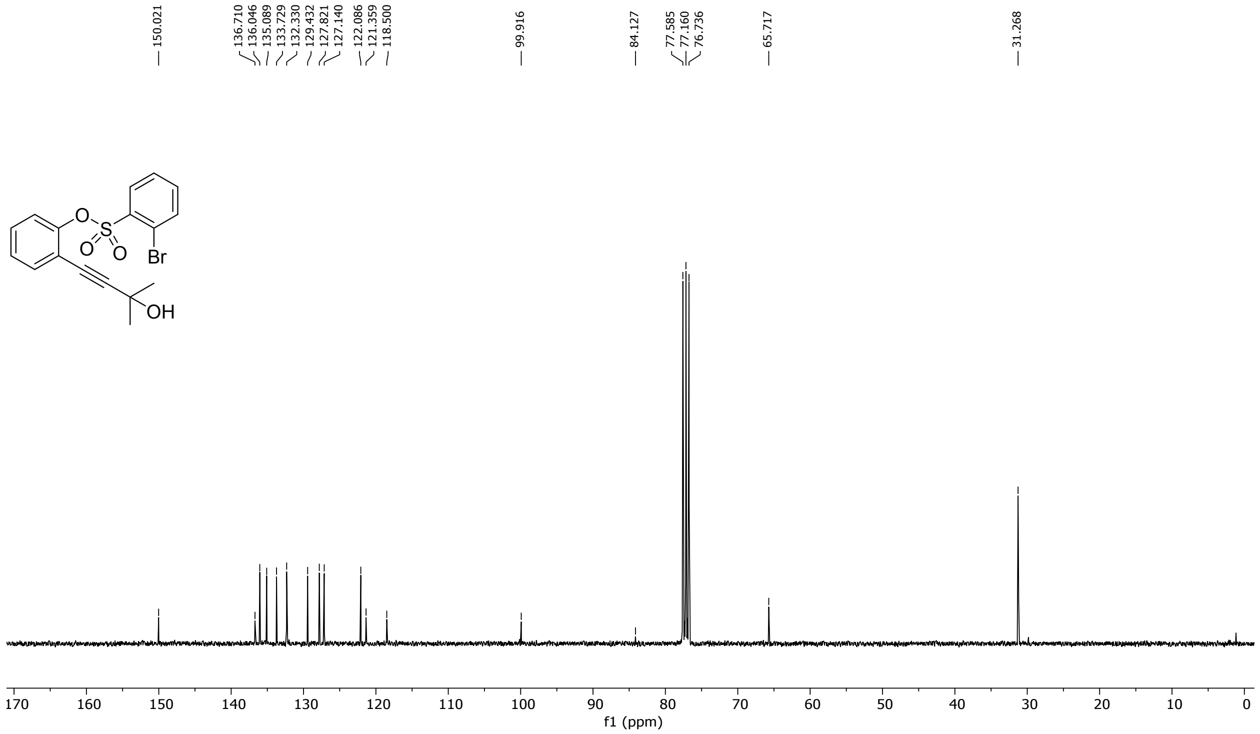

**Figure S17.** $^{1}$**H NMR (400 MHz) and** $^{13}$**C NMR (100 MHz) spectra of compound 2-(5-hydroxypent-1-yn-1-yl)phenyl 2-bromobenzenesulfonate (2q) in** $CDCl_3$.

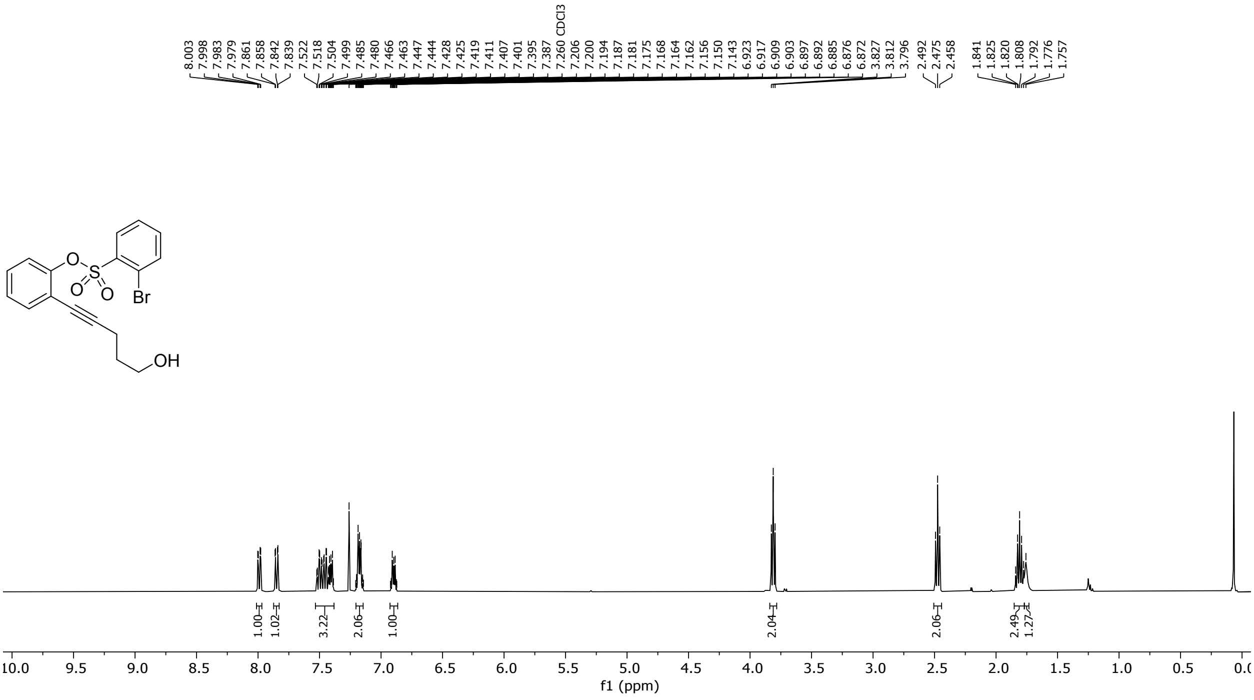


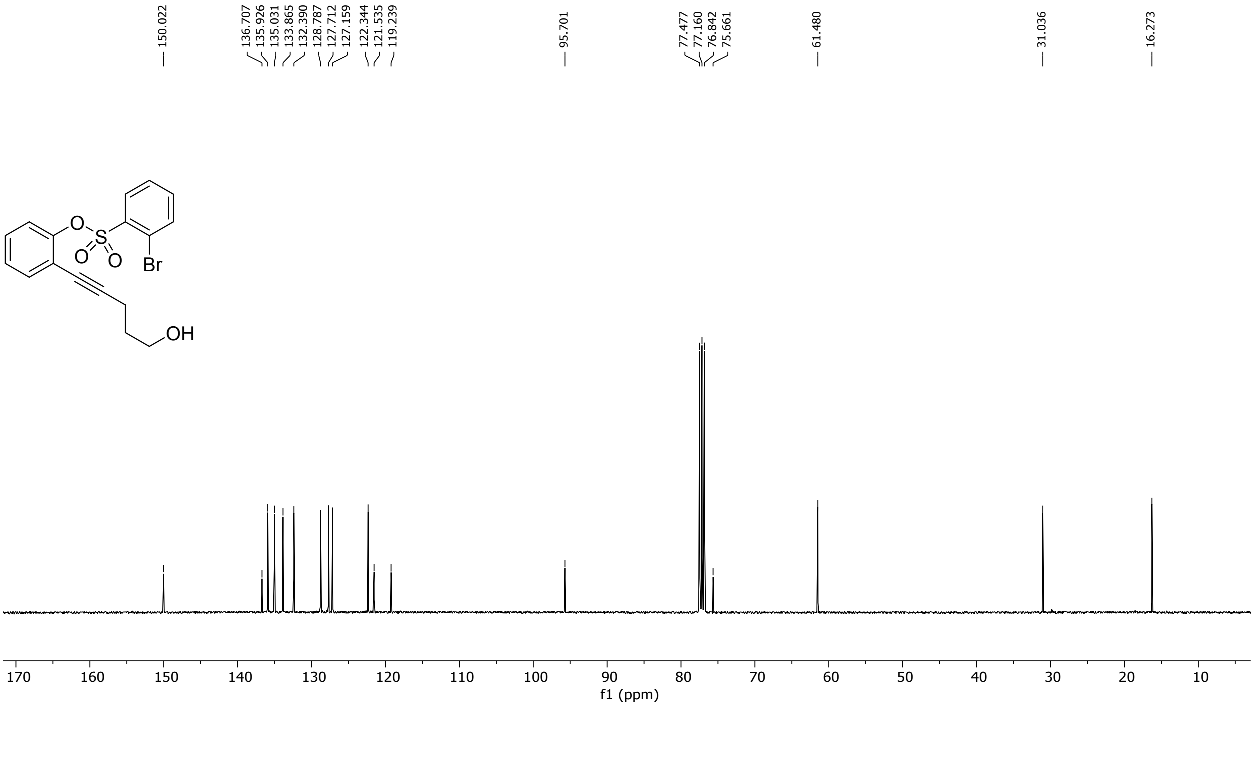

**Figure S18.** $^{1}$H NMR (300 MHz) and $^{13}$C NMR (75 MHz) spectra of compound 2-(4-hydroxybut-1-yn-1-yl)phenyl 2-bromobenzenesulfonate (2r) in $CDCl_3$.

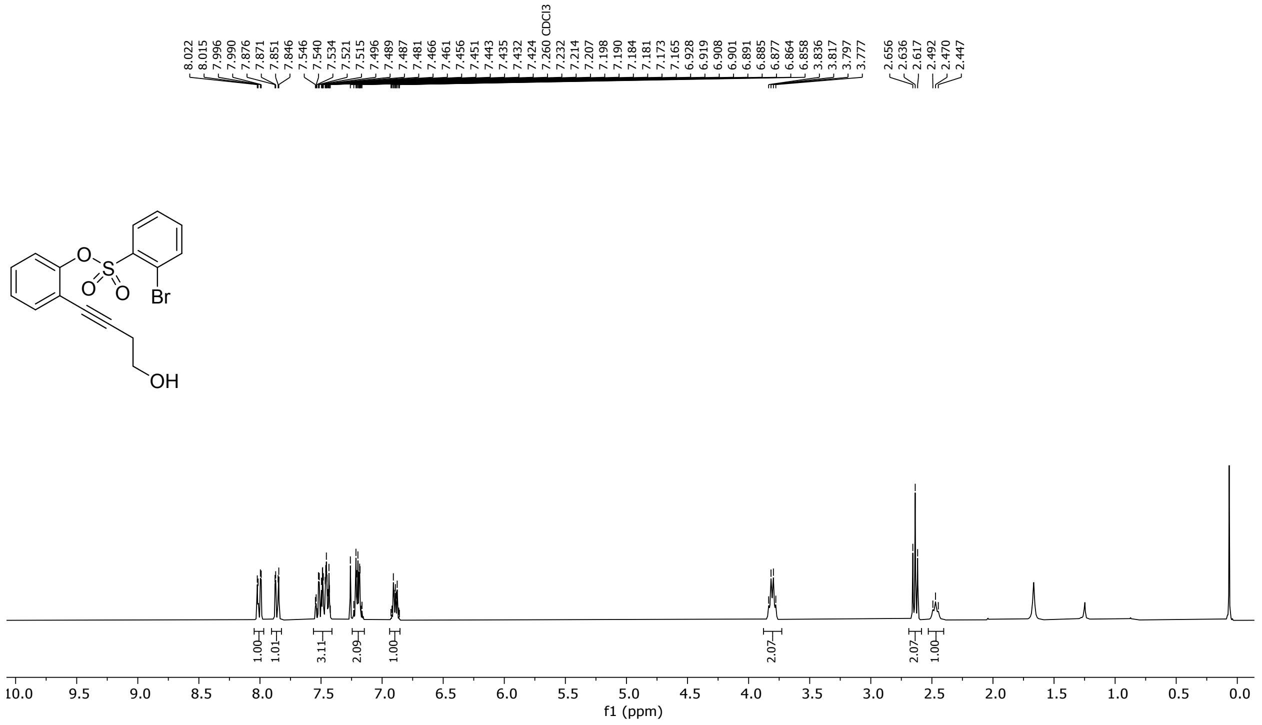

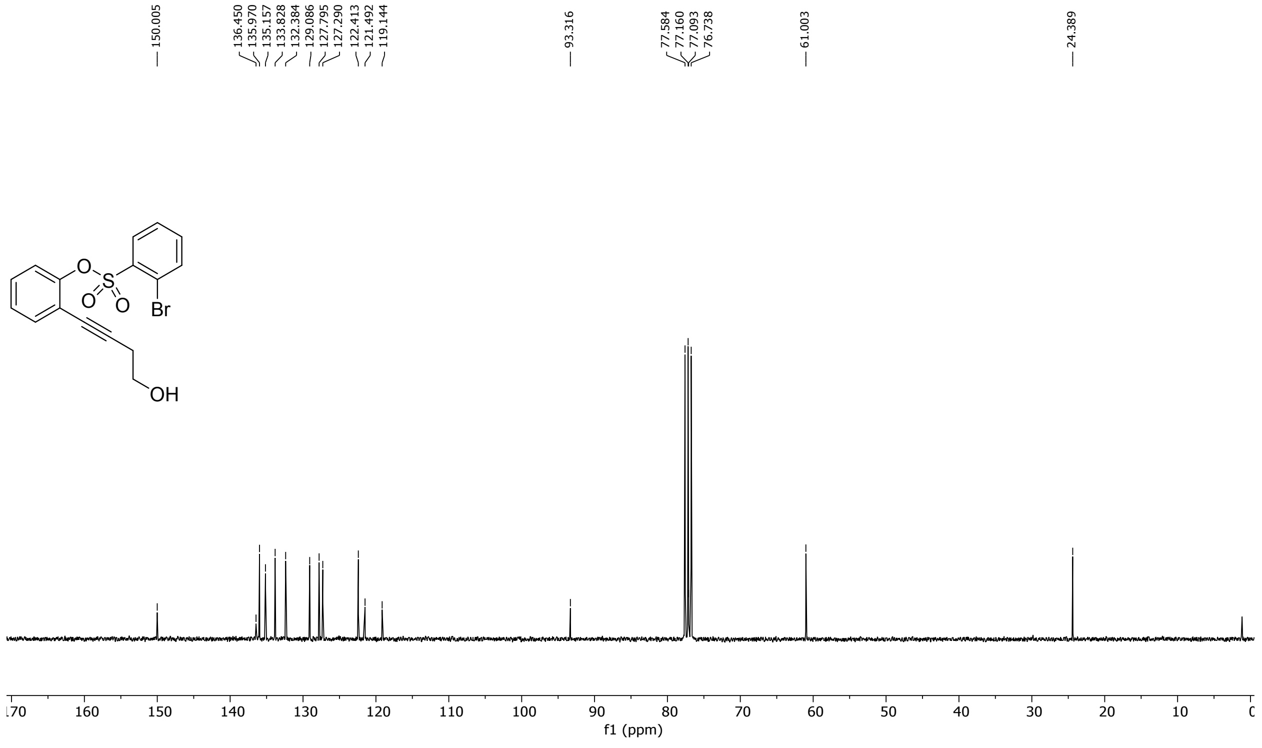

**Figure S19. $^{1}$H NMR (300 MHz) and $^{13}$C NMR (75 MHz) spectra of compound 2-(3-hydroxyprop-1-yn-1-yl)phenyl 2-bromobenzenesulfonate (2s) in $CDCl_3$.**

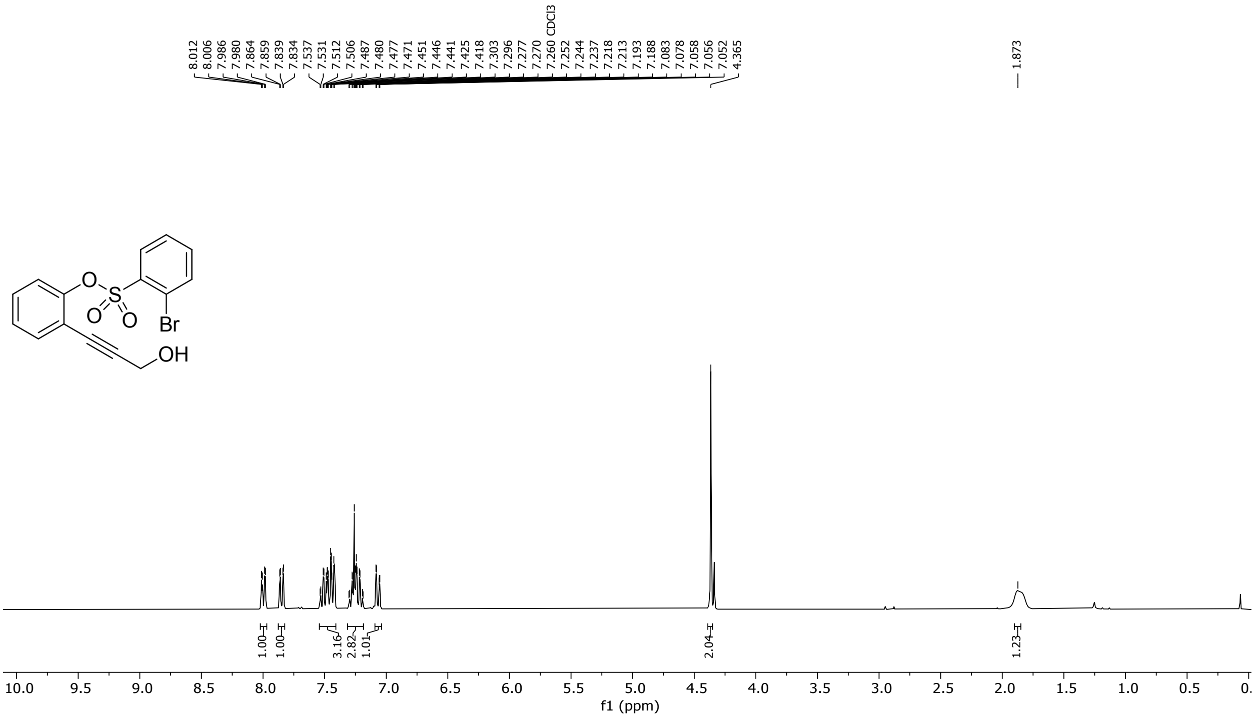


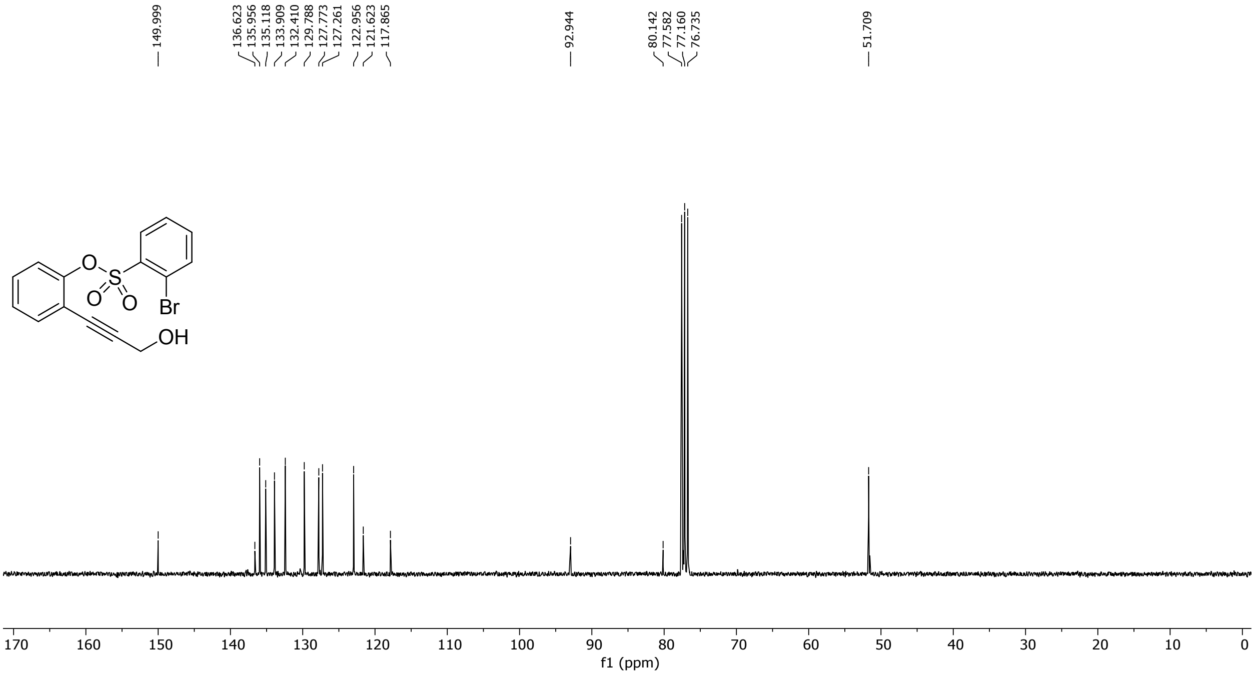

**Figure S20.** $^{1}$H NMR (400 MHz) and $^{13}$C NMR (100 MHz) spectra of compound 2-(phenylethynyl)pyridin-3-yl 2-bromobenzenesulfonate (2t) in $CDCl_3$.

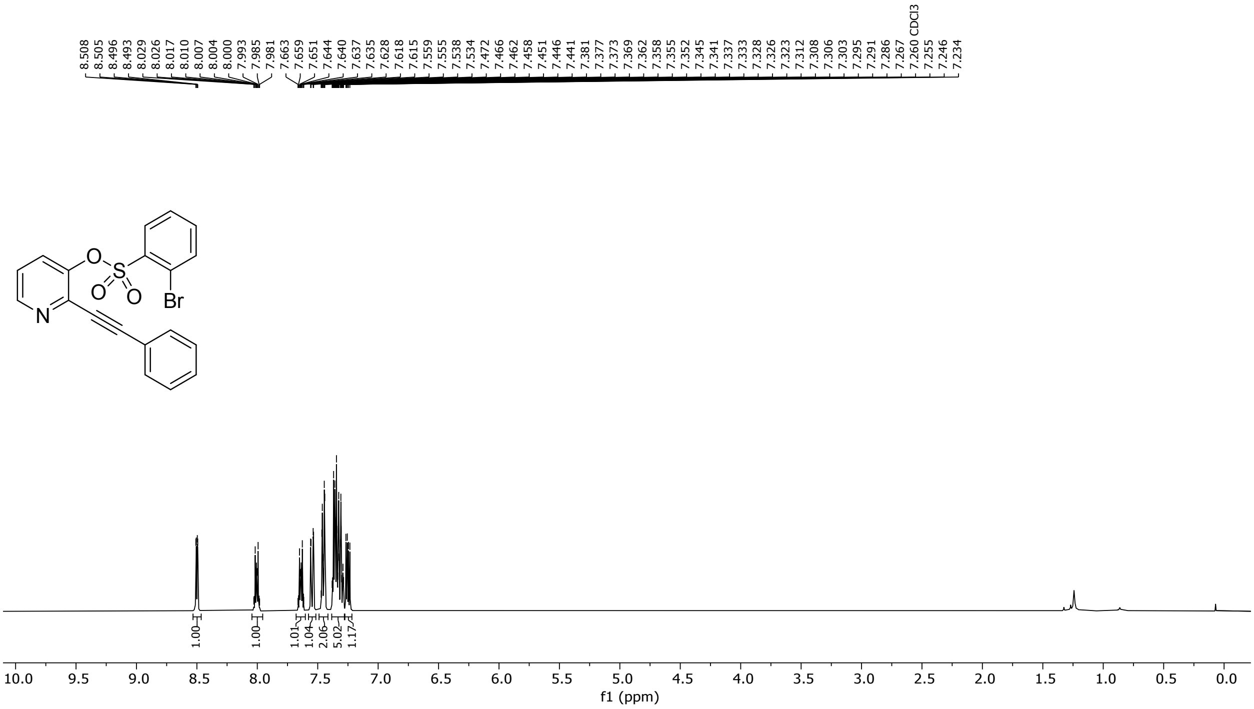


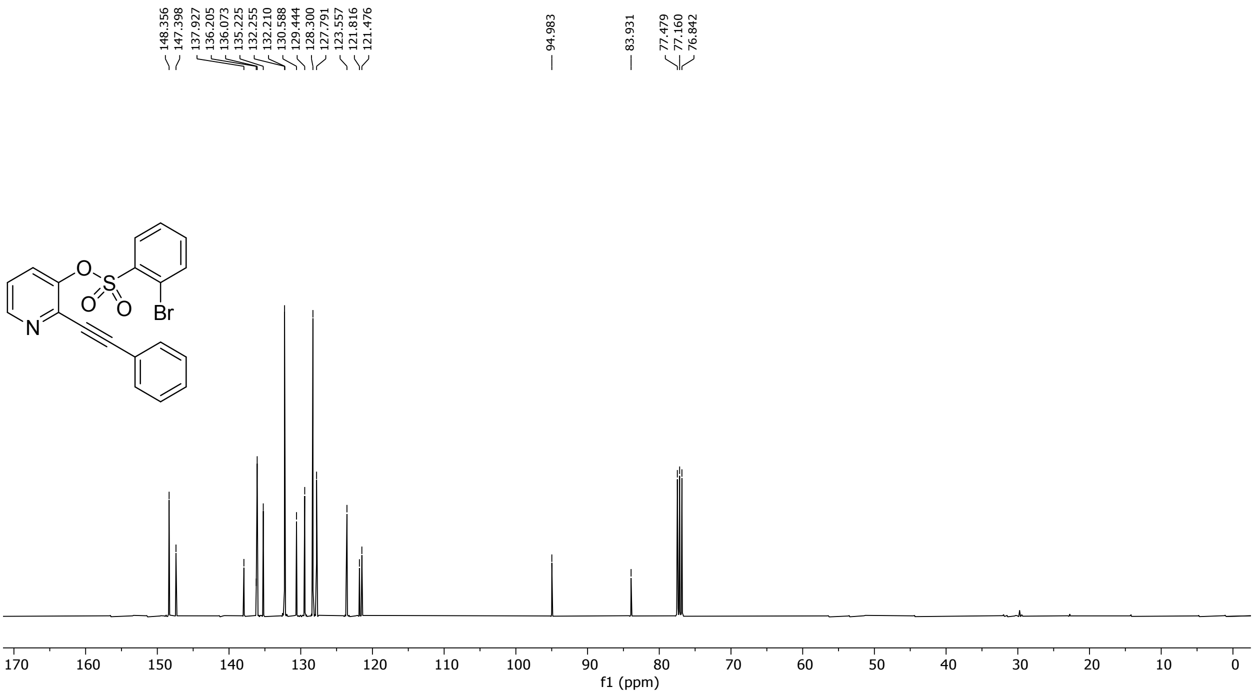

**Figure S21. $^1$H NMR (300 MHz) and $^{13}$C NMR (100 MHz) spectra of compound 4-fluoro-2-((4-methoxyphenyl)ethynyl)phenyl 2-bromobenzenesulfonate (2u) in $CDCl_3$.**

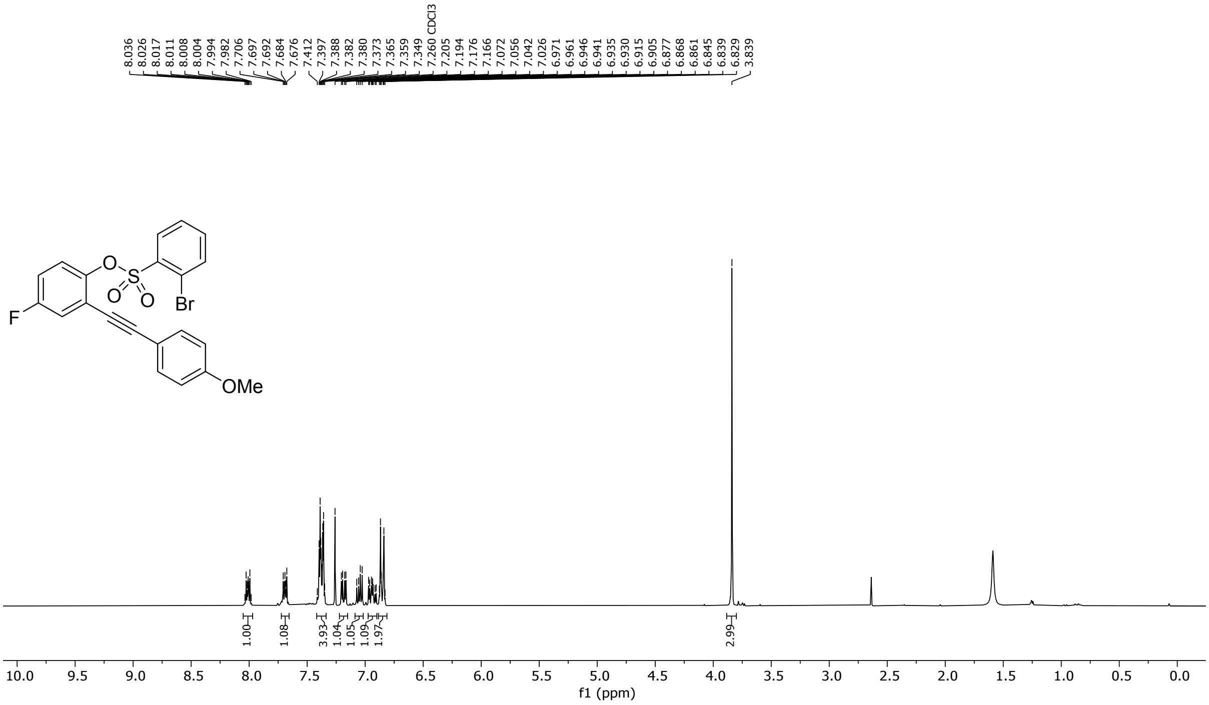

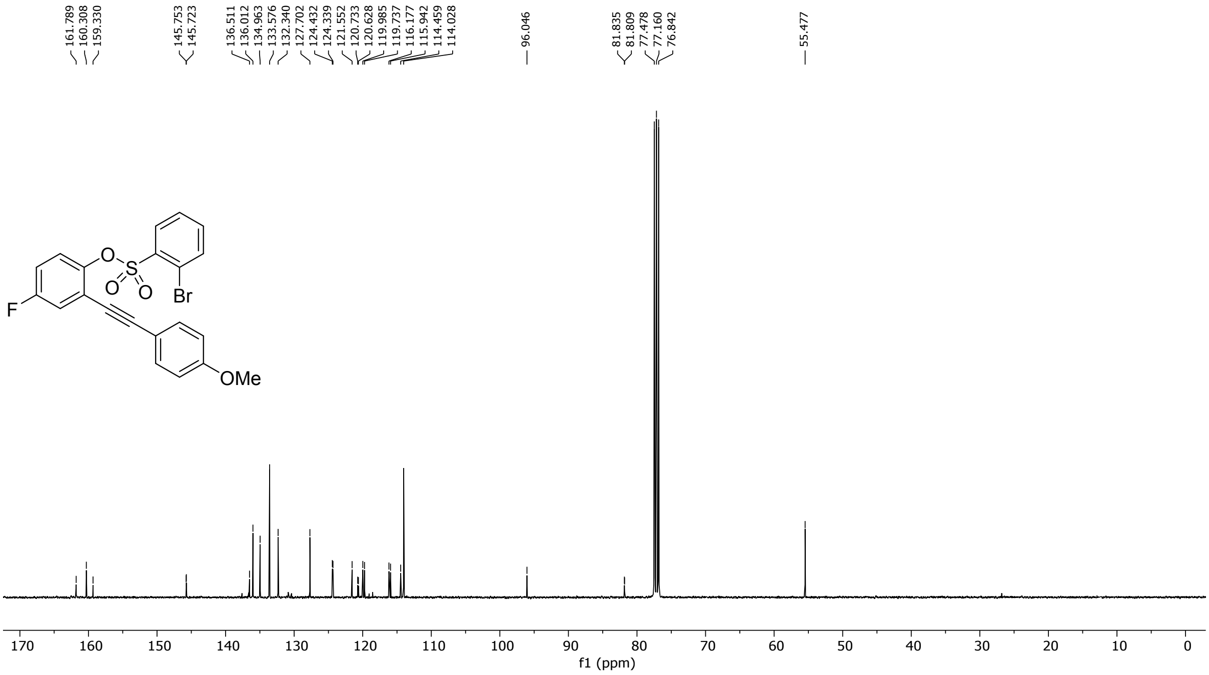

**Figure S22. $^{1}$H NMR (300 MHz) and $^{13}$C NMR (100 MHz) spectra of compound 2-ethynylphenyl 2-bromobenzenesulfonate (2v) in $CDCl_3$.**

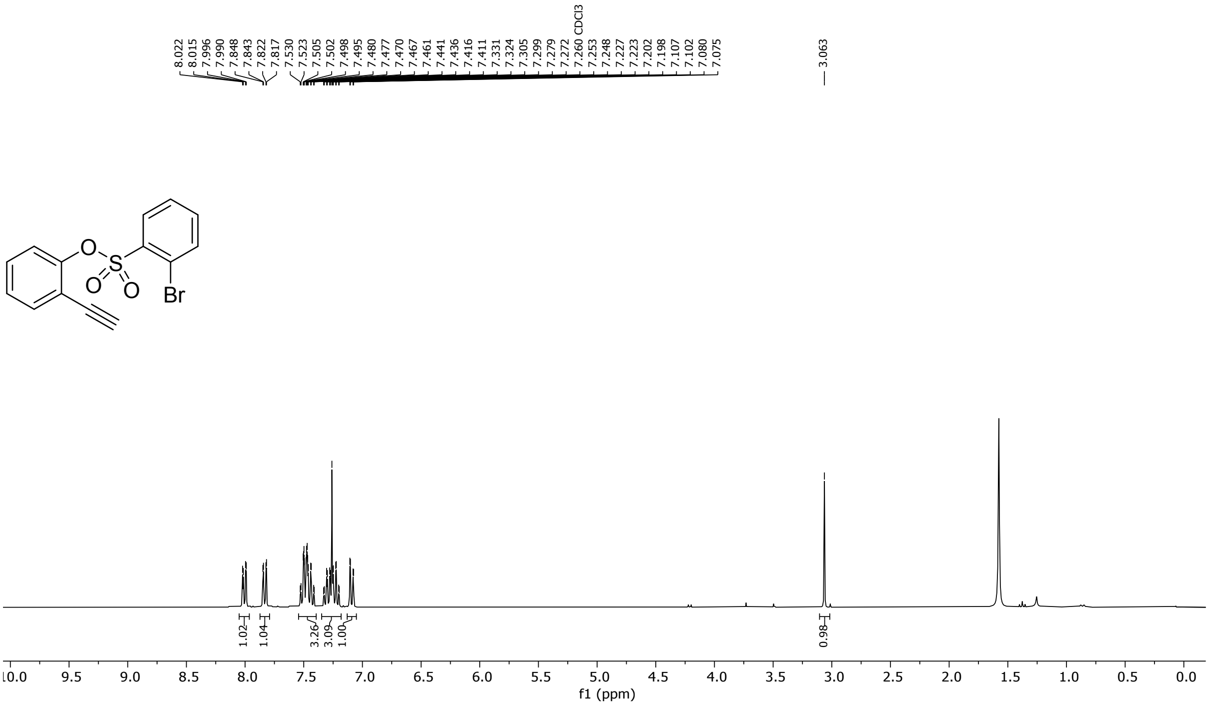


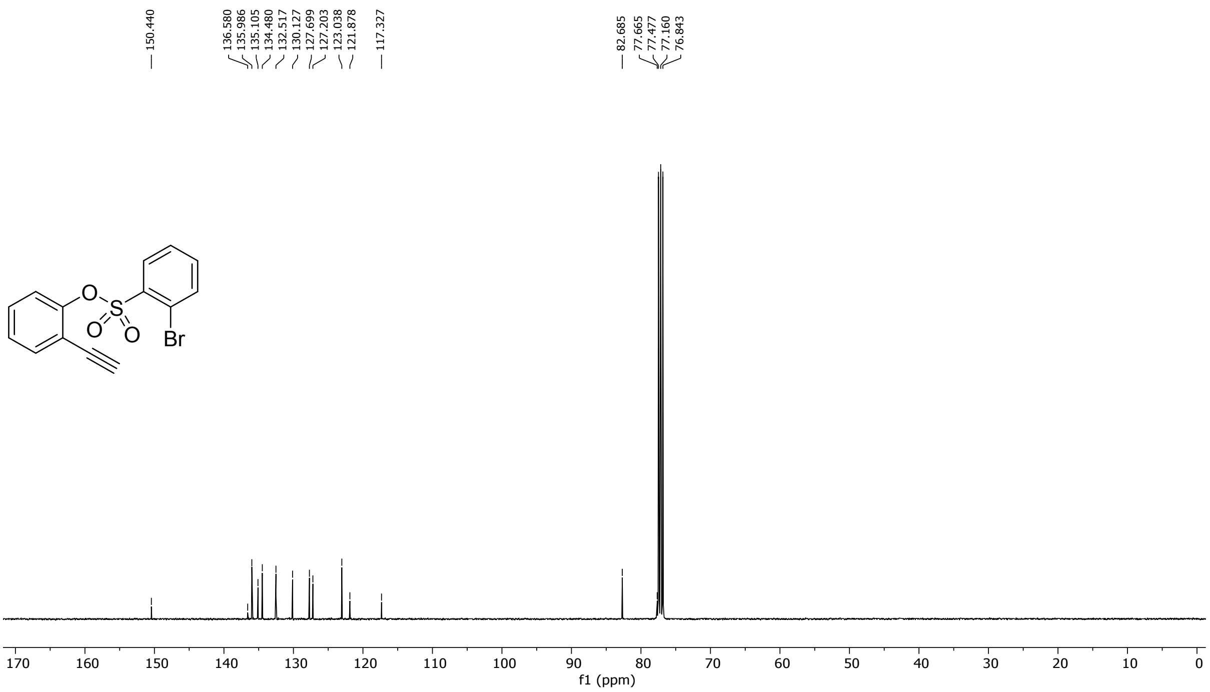

## 7) $^{1}H$ and $^{13}C$-NMR spectra of compounds 3a-v:

**Figure S23. $^{1}H$ NMR (400 MHz) and $^{13}C$ NMR (100 MHz) spectra of compound (*Z*)-11-benzylidene-6,11-dihydrodibenzo[*b*,*e*]oxepine (3a) in $CDCl_3$.**

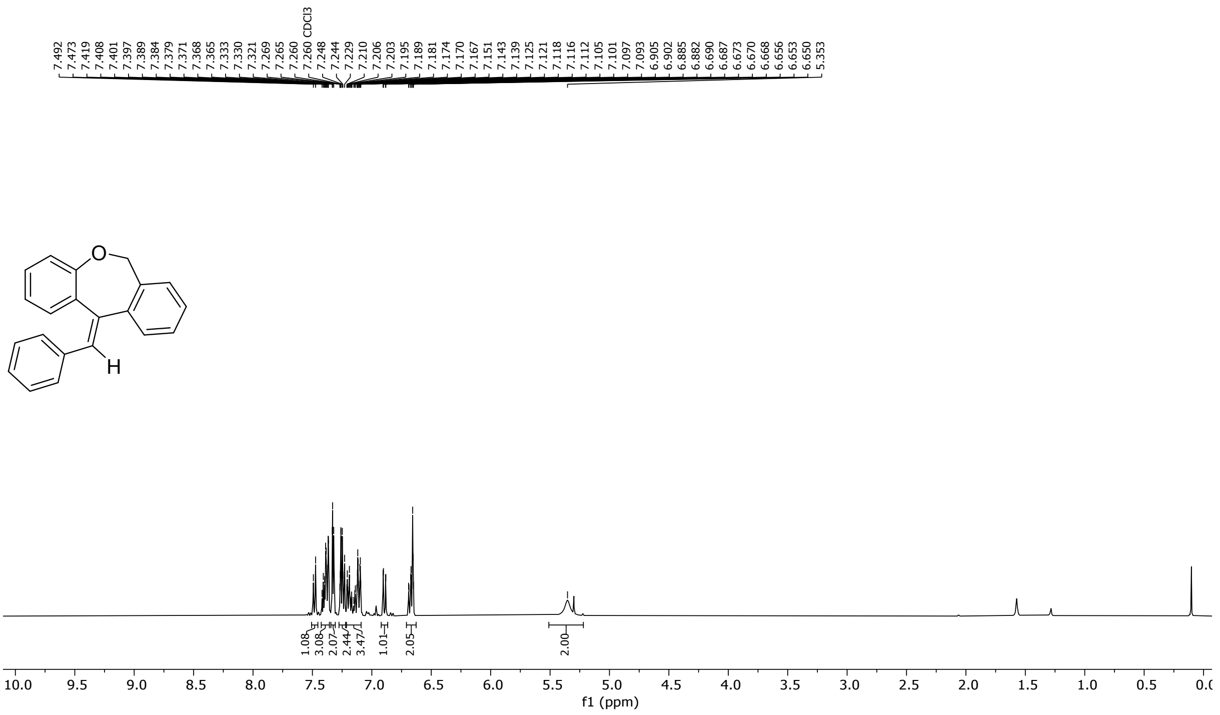


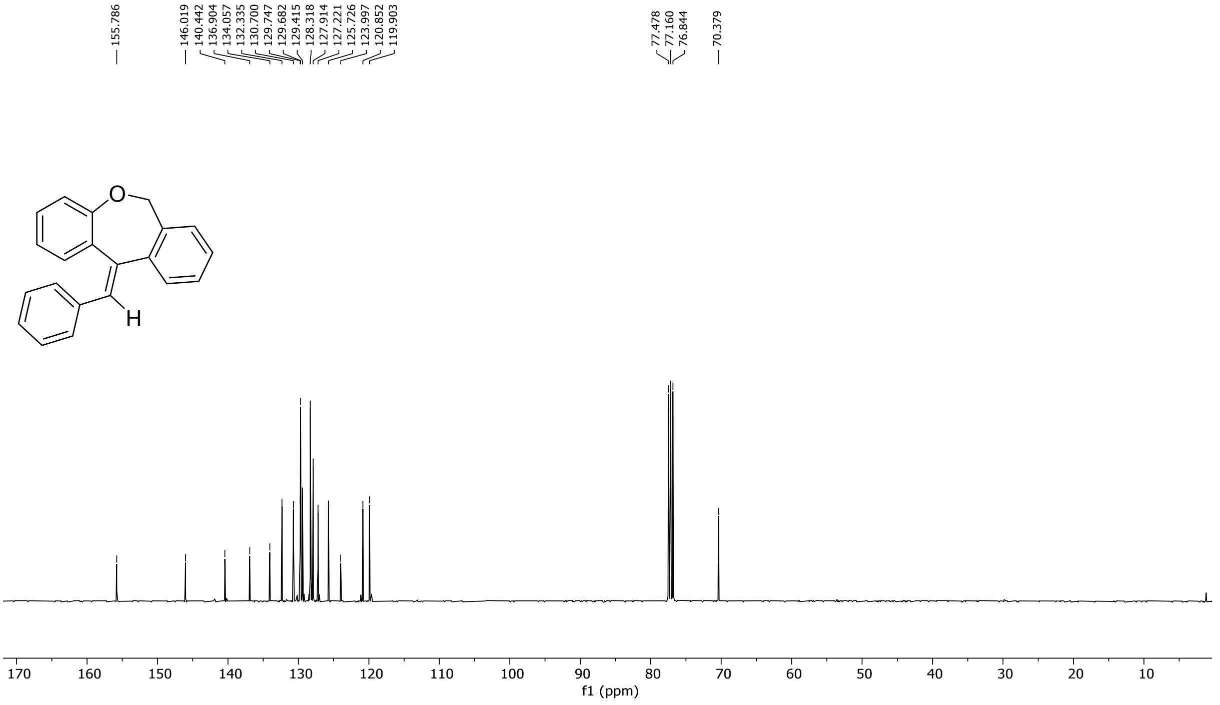

**Figure S24. $^{1}$H NMR (300 MHz) and $^{13}$C NMR (75 MHz) spectra of compound (*Z*)-11-(4-methoxybenzylidene)-6,11-dihydrodibenzo[*b*,*e*]oxepine (3b) in $CDCl_3$.**

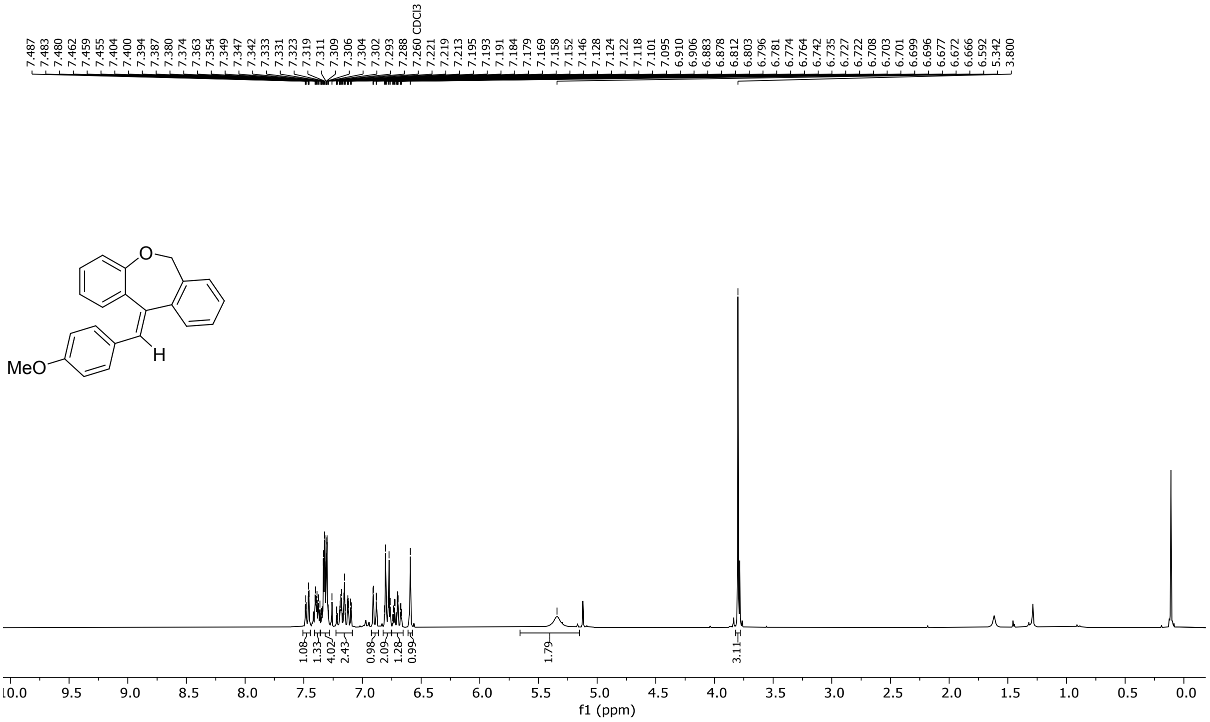


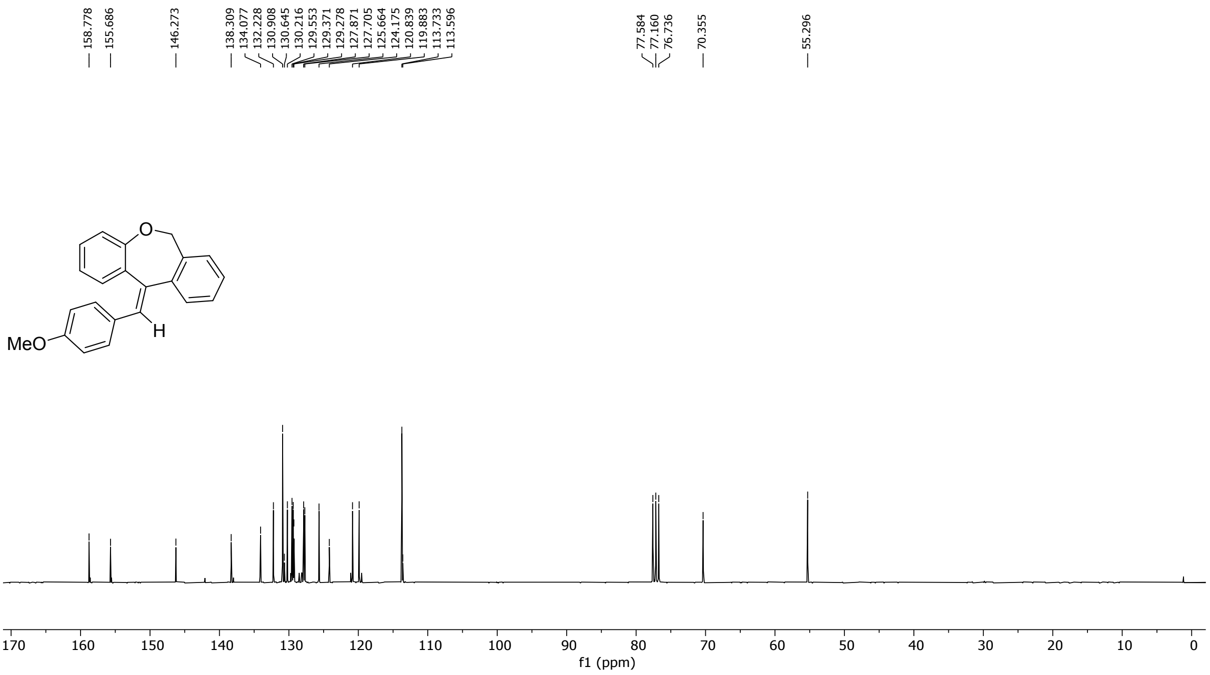

**Figure S25.** $^1$**H NMR (300 MHz) and** $^{13}$**C NMR (75 MHz) spectra of compound (*Z*)-11-(4-methylbenzylidene)-6,11-dihydrodibenzo[*b*,*e*]oxepine (3c) in CDCl$_3$.**

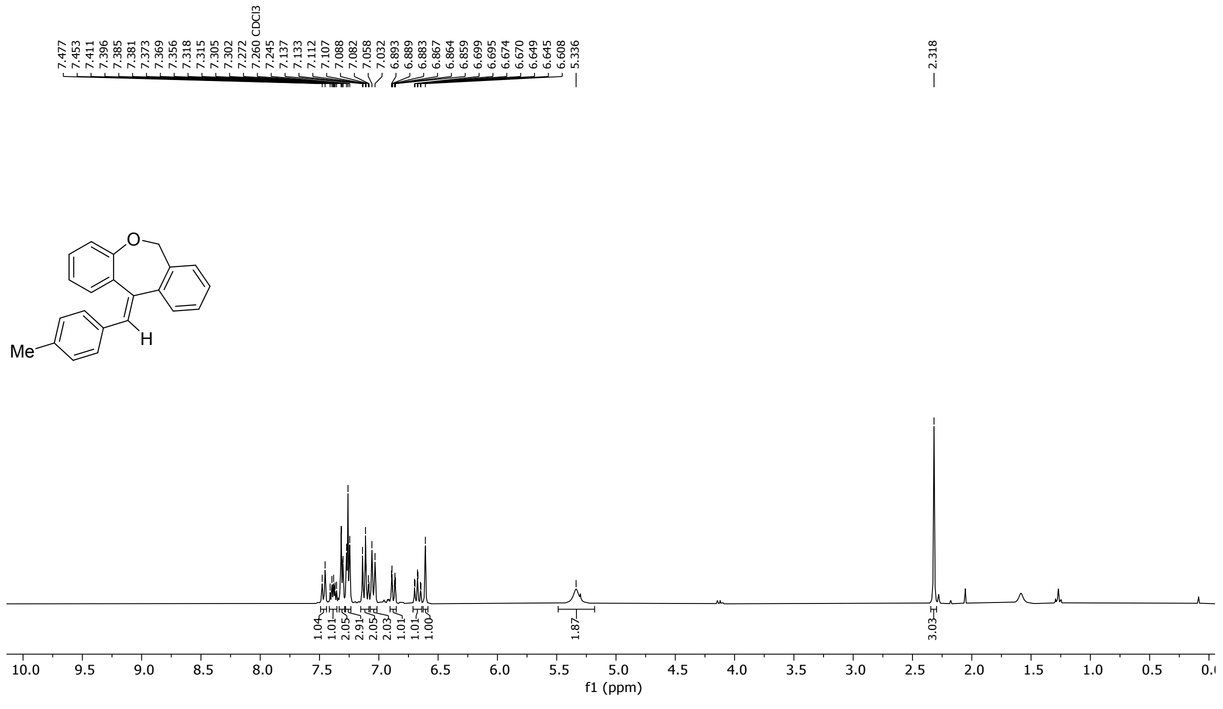

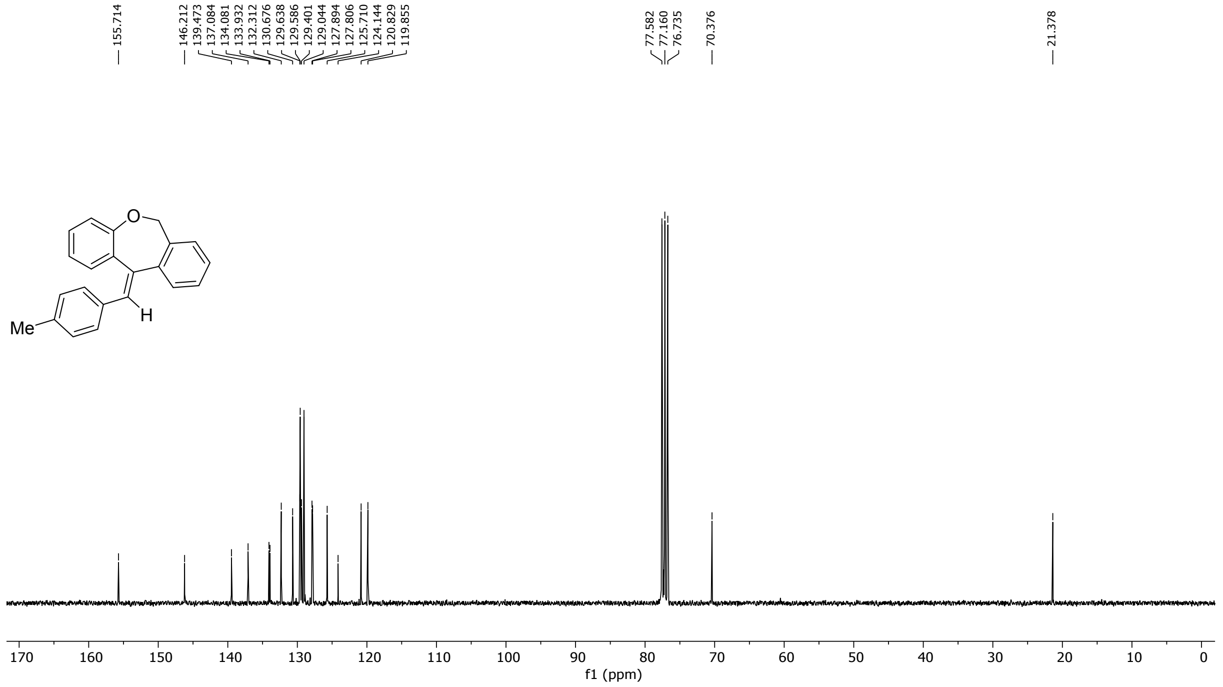

**Figure S26. $^{1}H$ NMR (300 MHz) and $^{13}C$ NMR (75 MHz) spectra of compound (*Z*)-11-hexylidene-6,11-dihydrodibenzo[*b*,*e*]oxepine (3d) in $CDCl_3$.**

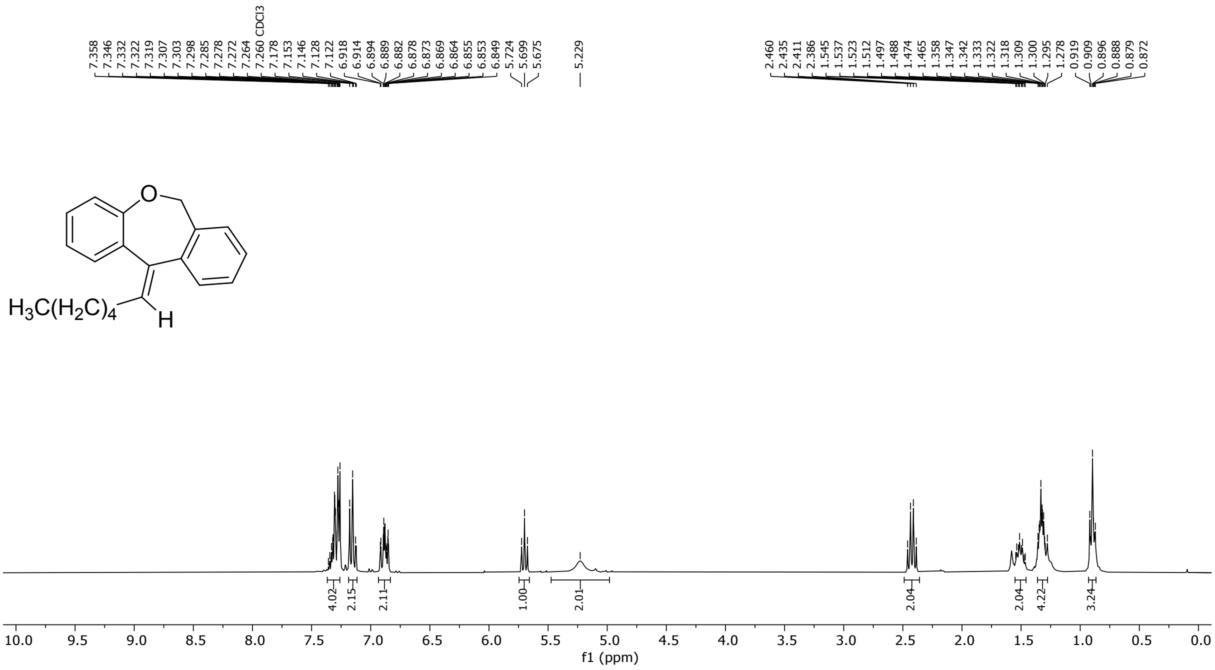


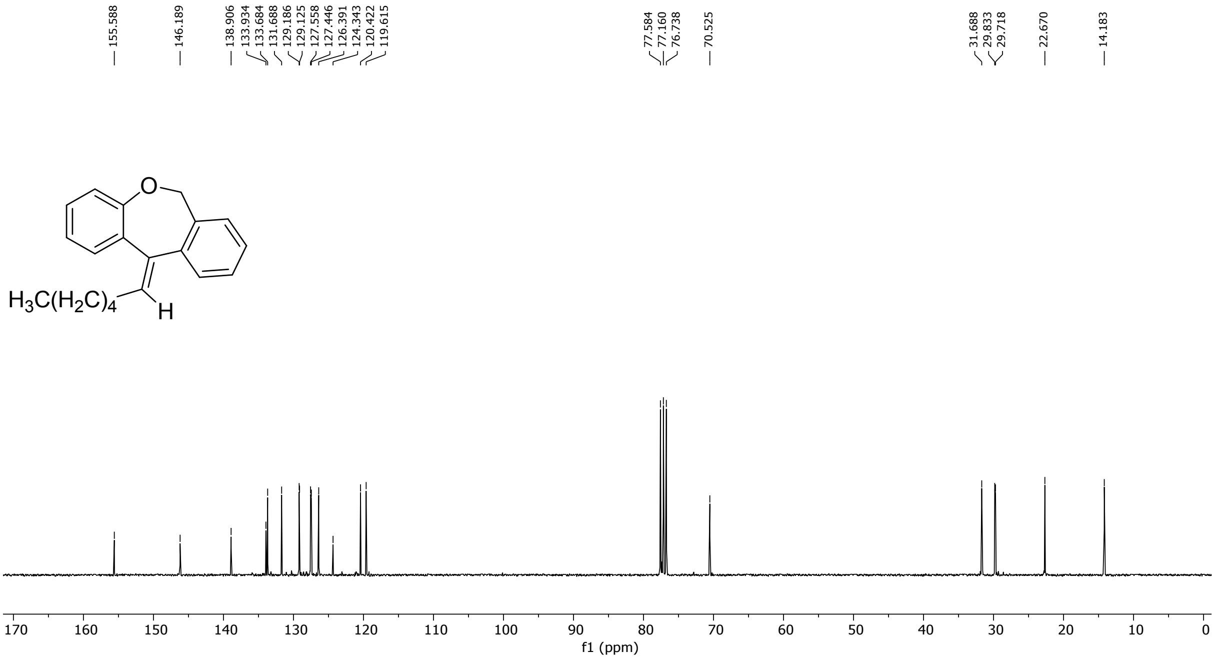

**Figure S27. $^{1}$H NMR (300 MHz) and $^{13}$C NMR (75 MHz) spectra of compound (*Z*)-11-pentylidene-6,11-dihydrodibenzo[*b*,*e*]oxepine (3e) in $CDCl_3$.**

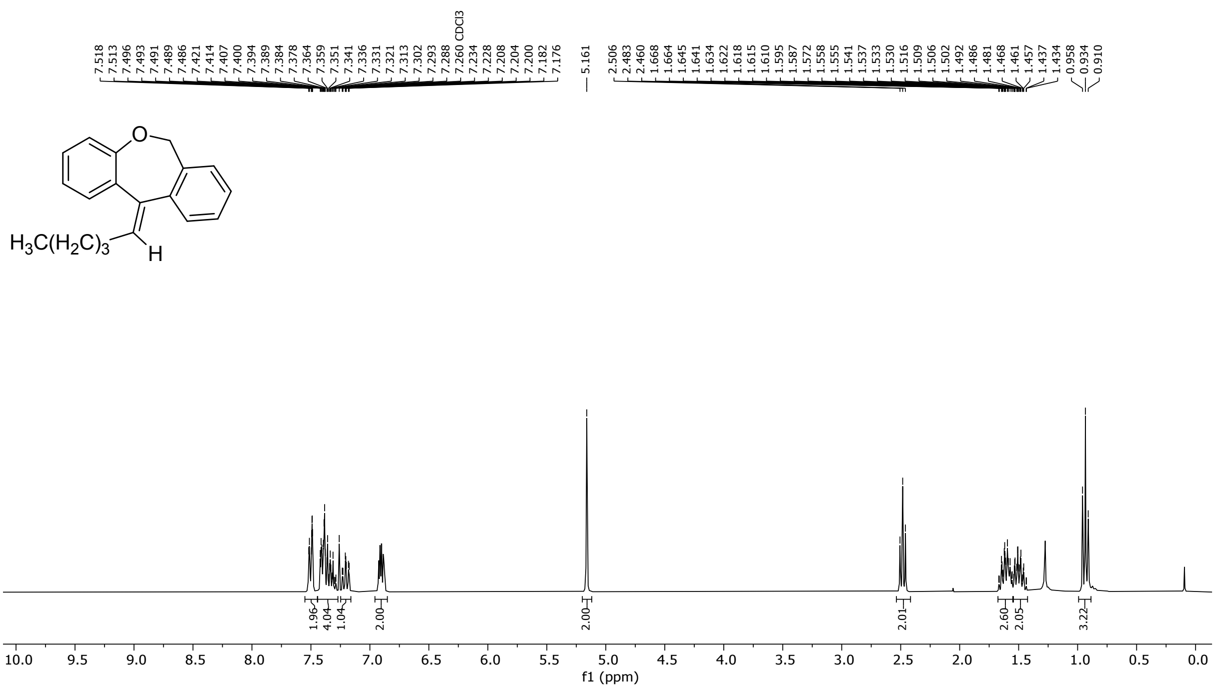


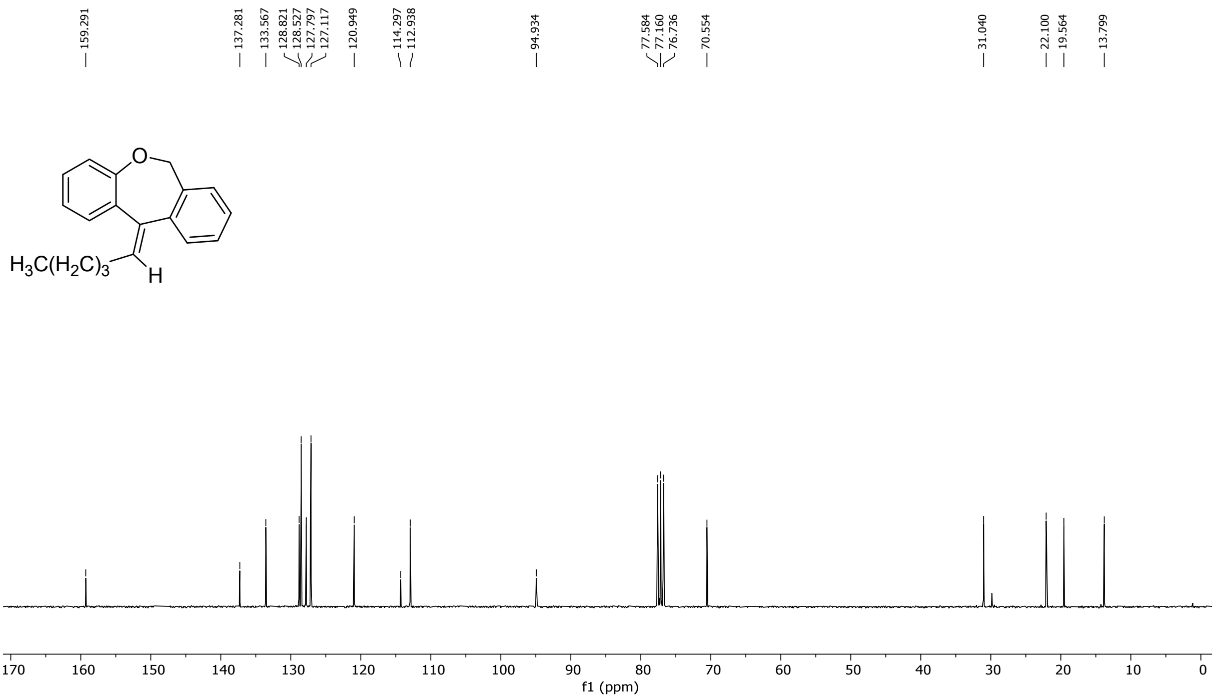

**Figure S28. ¹H NMR (400 MHz) and ¹³C NMR (100 MHz) spectra of compound (*Z*)-3-(dibenzo[*b*,*e*]oxepin-11(6*H*)-ylidene)propan-1-ol (3f) in $CDCl_3$.**

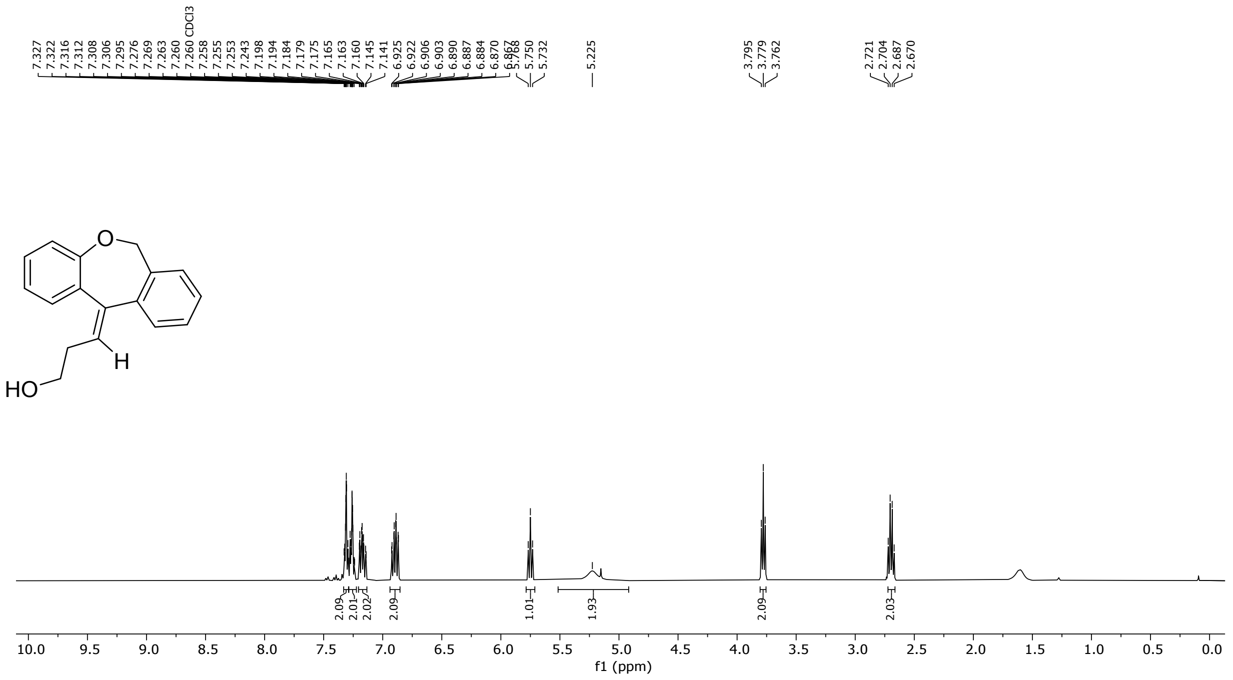


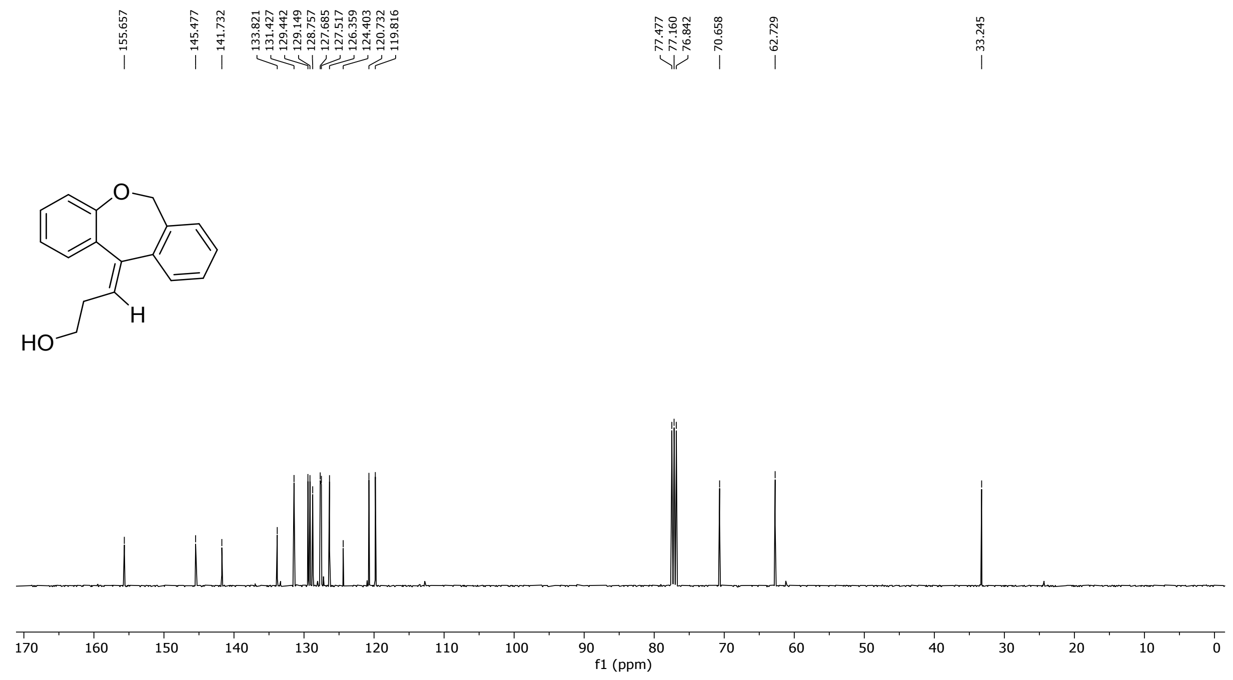

**Figure S29.** $^1H$ **NMR (300 MHz) and** $^{13}C$ **NMR (75 MHz) spectra of compound (*Z*)-11-heptylidene-8-methoxy-6,11-dihydrodibenzo[*b*,*e*]oxepine (3g) in** $CDCl_3$**.**

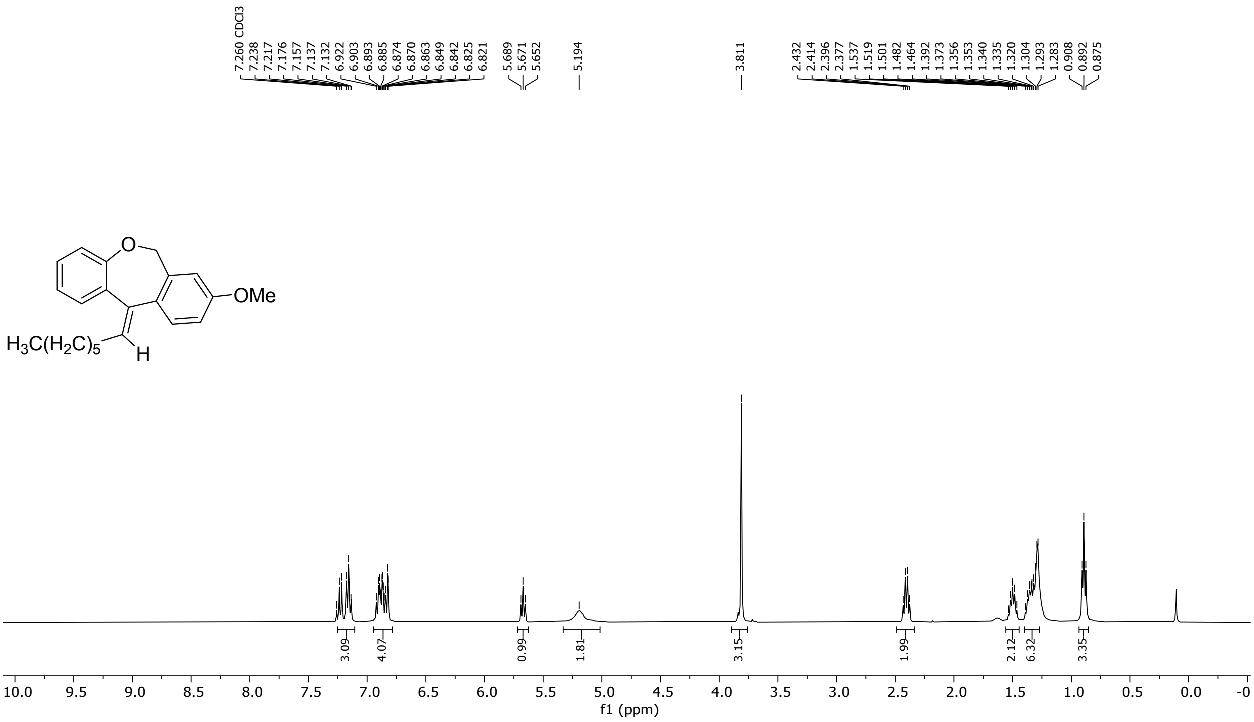

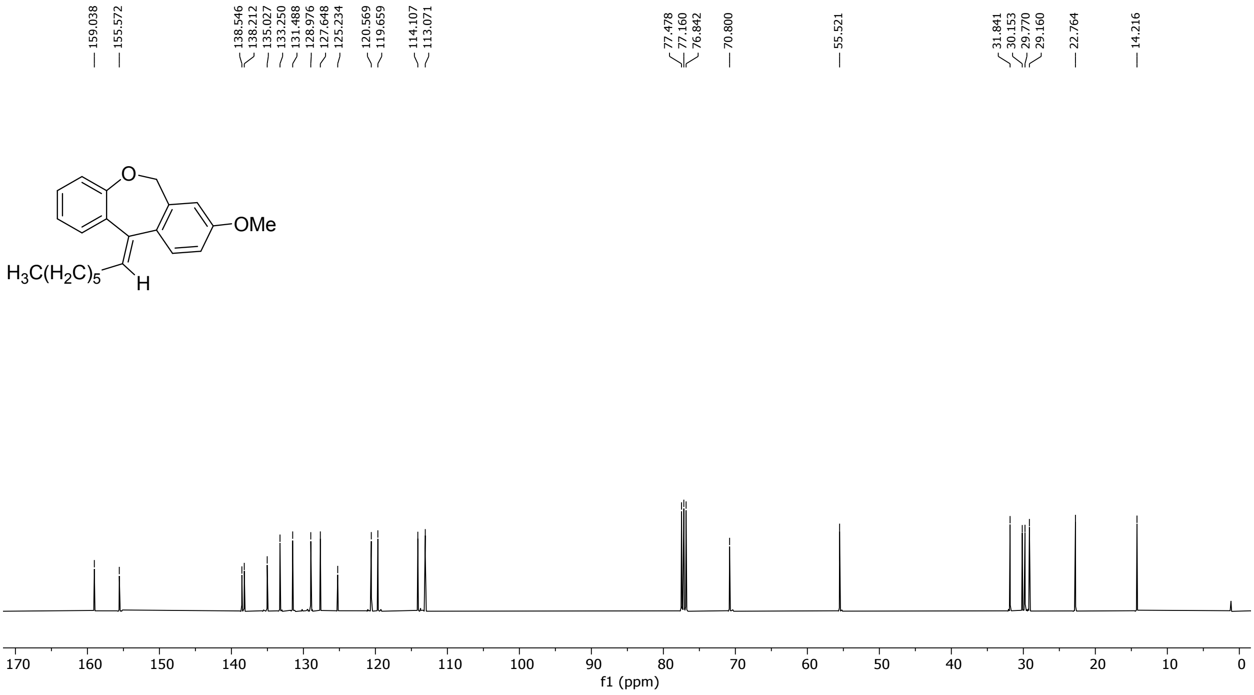

**Figure S30. $^{1}$H NMR (300 MHz) and $^{13}$C NMR (75 MHz) spectra of compound (*Z*)-1-(8-methoxydibenzo[*b*,*e*]oxepin-11(6*H*)-ylidene)-2-methylpropan-2-ol (3h) in $CDCl_3$.**

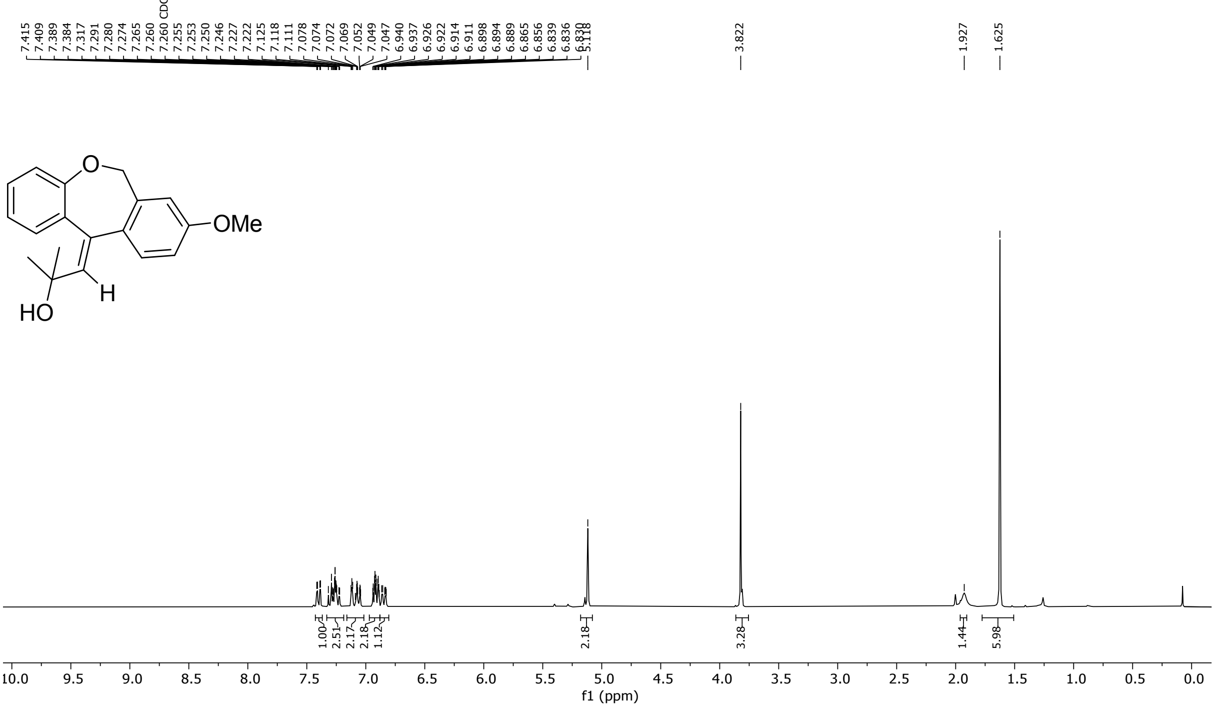


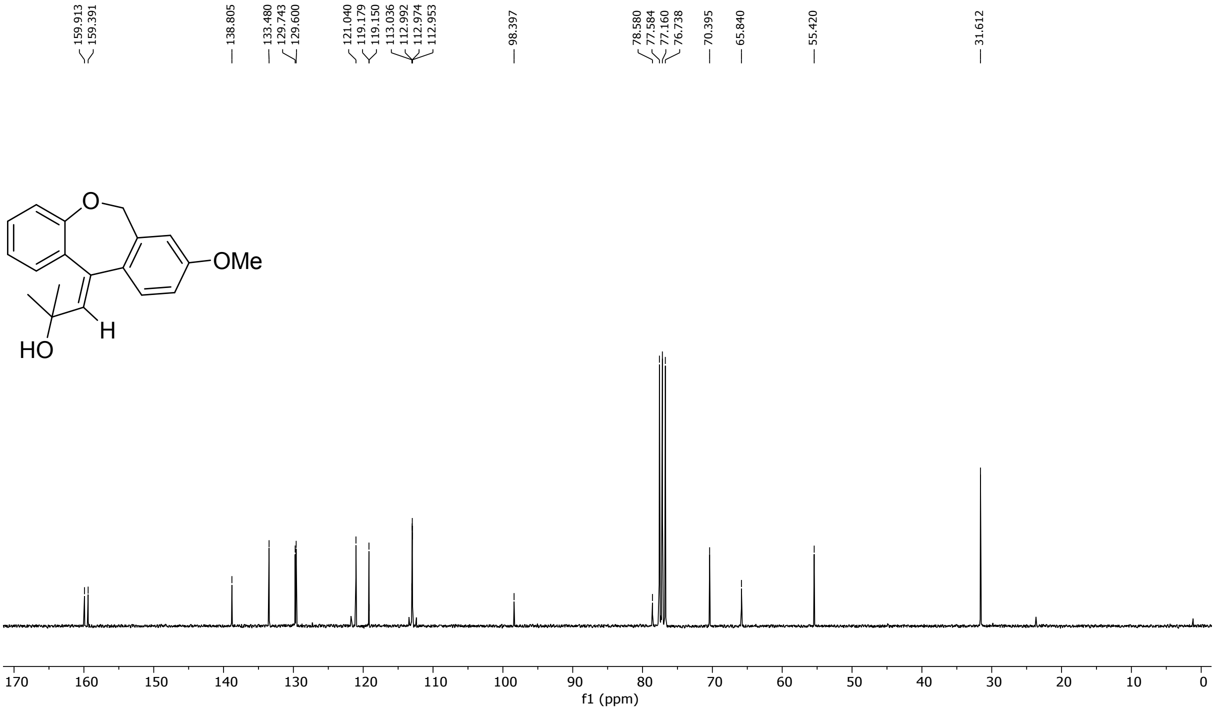

**Figure S31.** $^1$**H NMR (300 MHz) and** $^{13}$**C NMR (75 MHz) spectra of compound (*Z*)-11-butylidene-8-methoxy-6,11-dihydrodibenzo[*b*,*e*]oxepine (3i) in** $CDCl_3$.

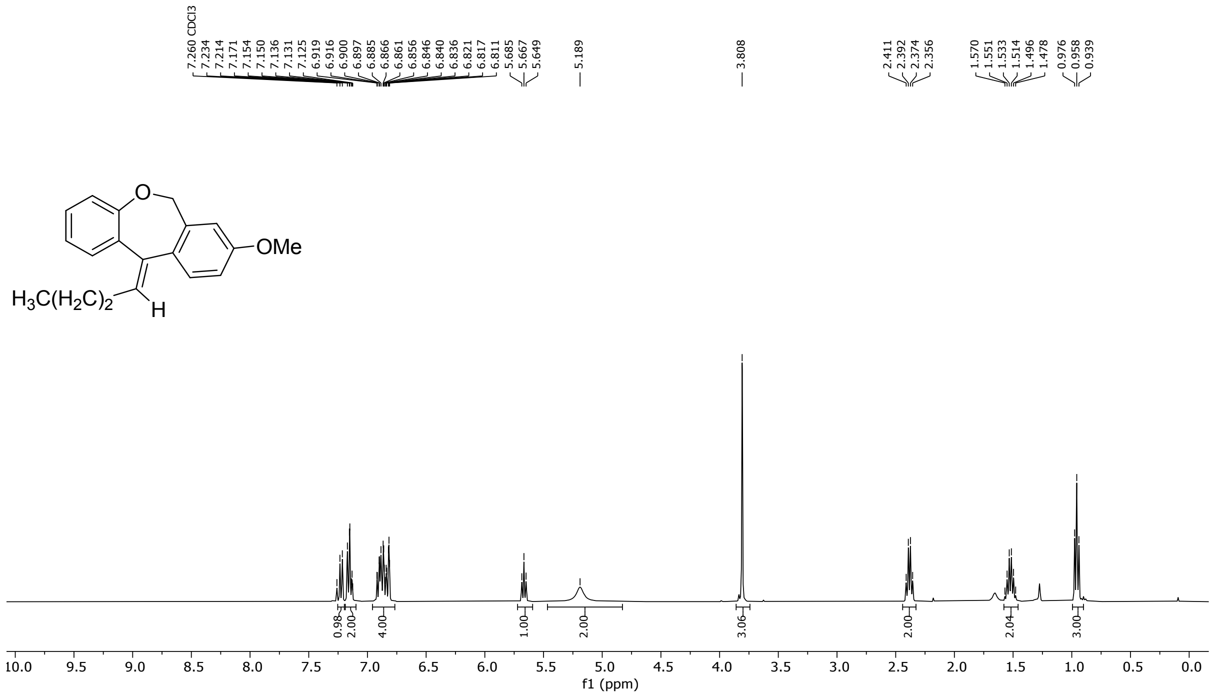

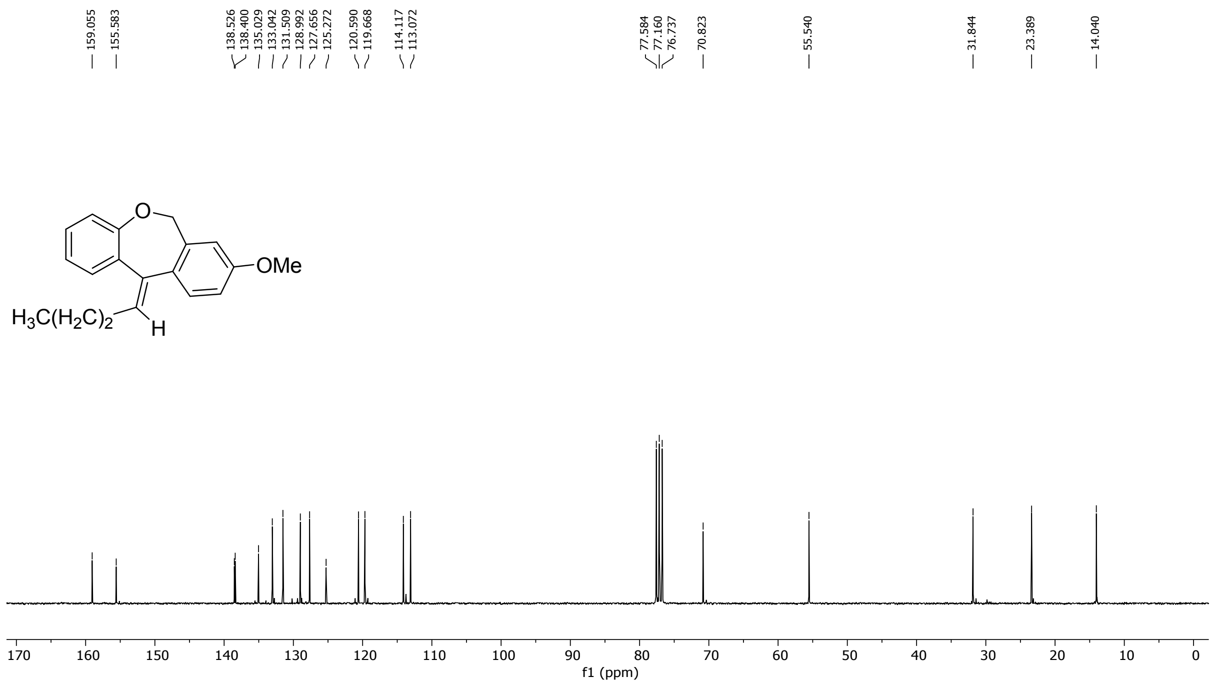

**Figure S32. $^{1}$H NMR (400 MHz) and $^{13}$C NMR (100 MHz) spectra of compound (E)-11-benzylidene-6,11-dihydrobenzo[5,6]oxepino[3,2-b]pyridine (3j) in $CDCl_3$.**

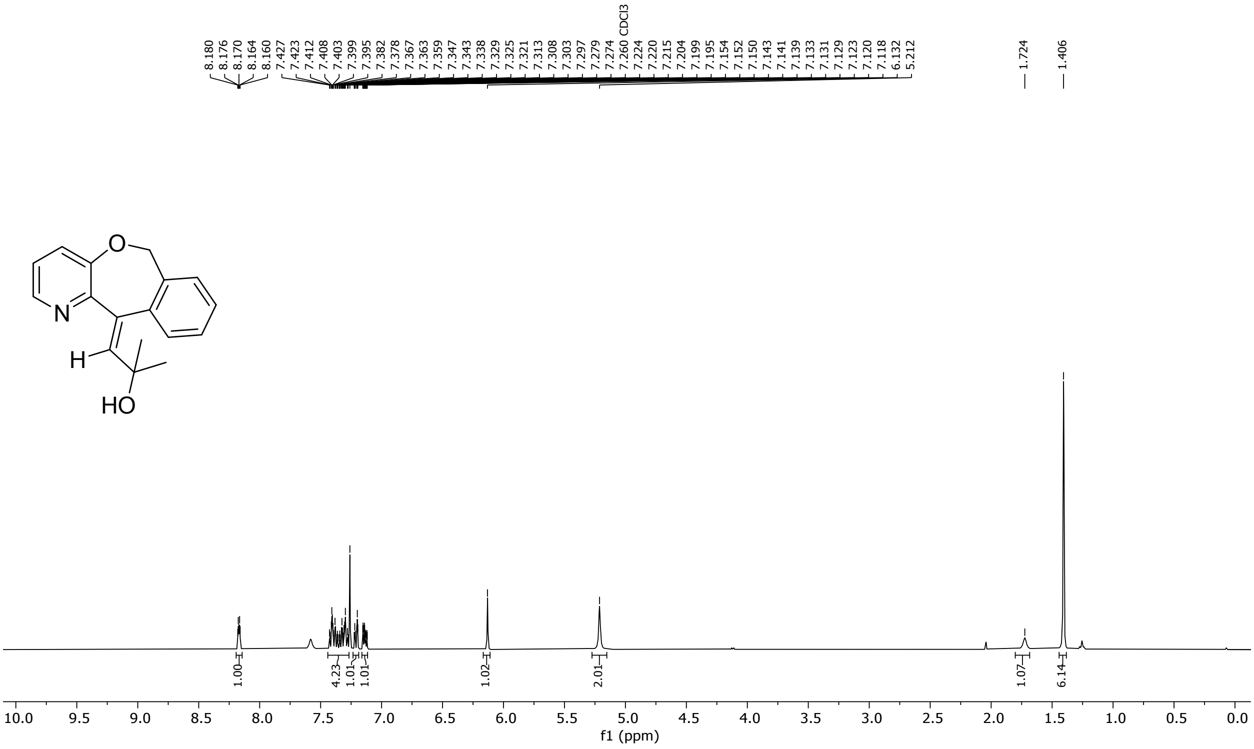


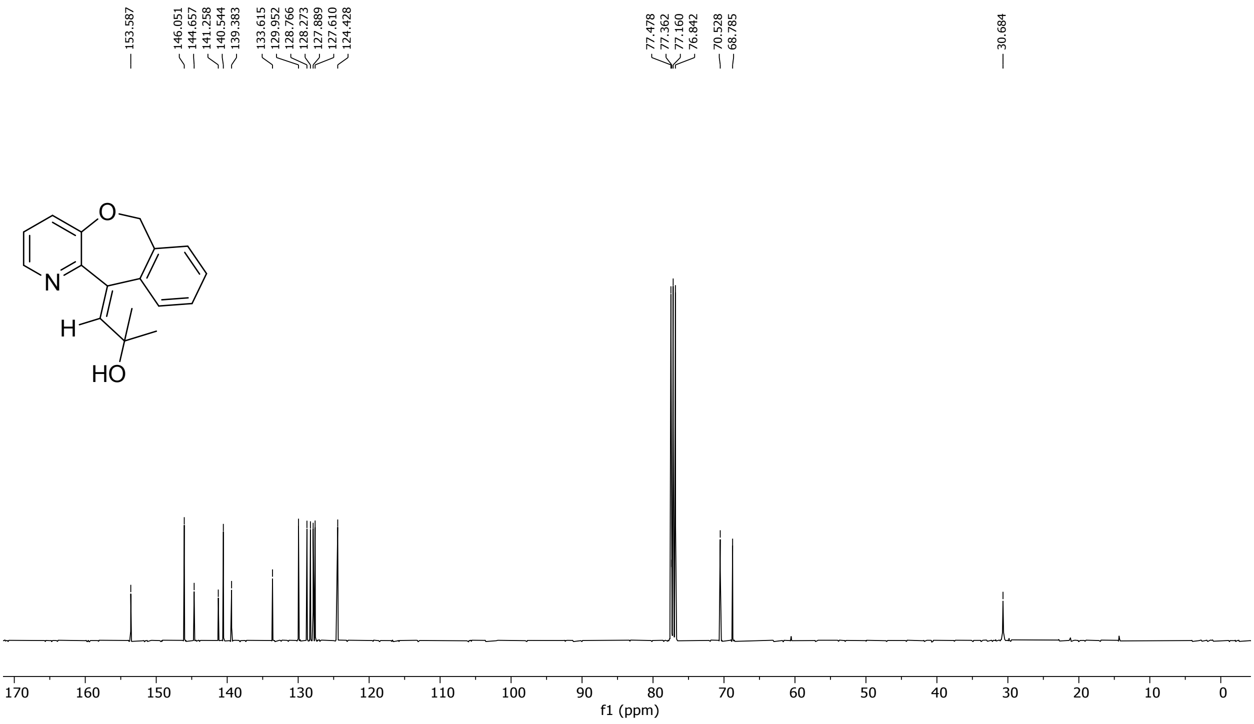

**Figure S33. $^{1}$H NMR (300 MHz) and $^{13}$C NMR (75 MHz) spectra of compound (*E*)-11-benzylidene-11*H*-dibenzo[*c*,*f*][1,2]oxathiepine 6,6-dioxide (3k) in $CDCl_3$.**

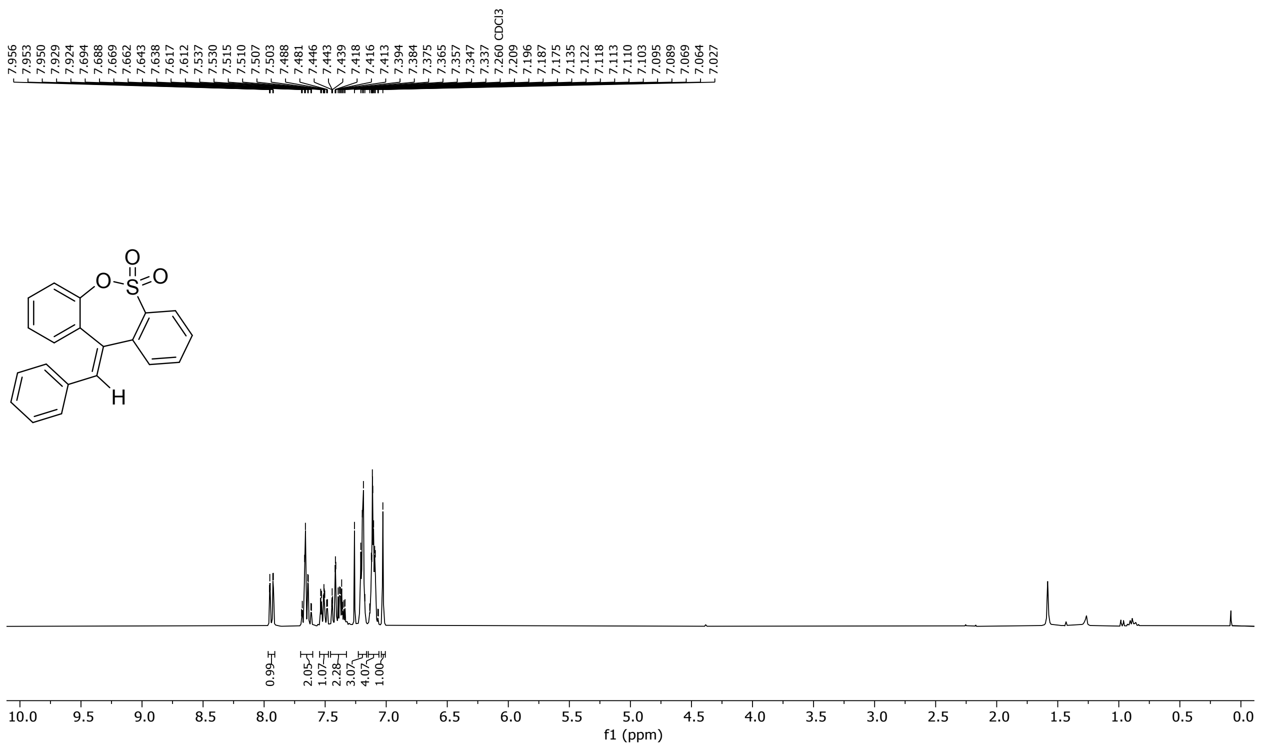


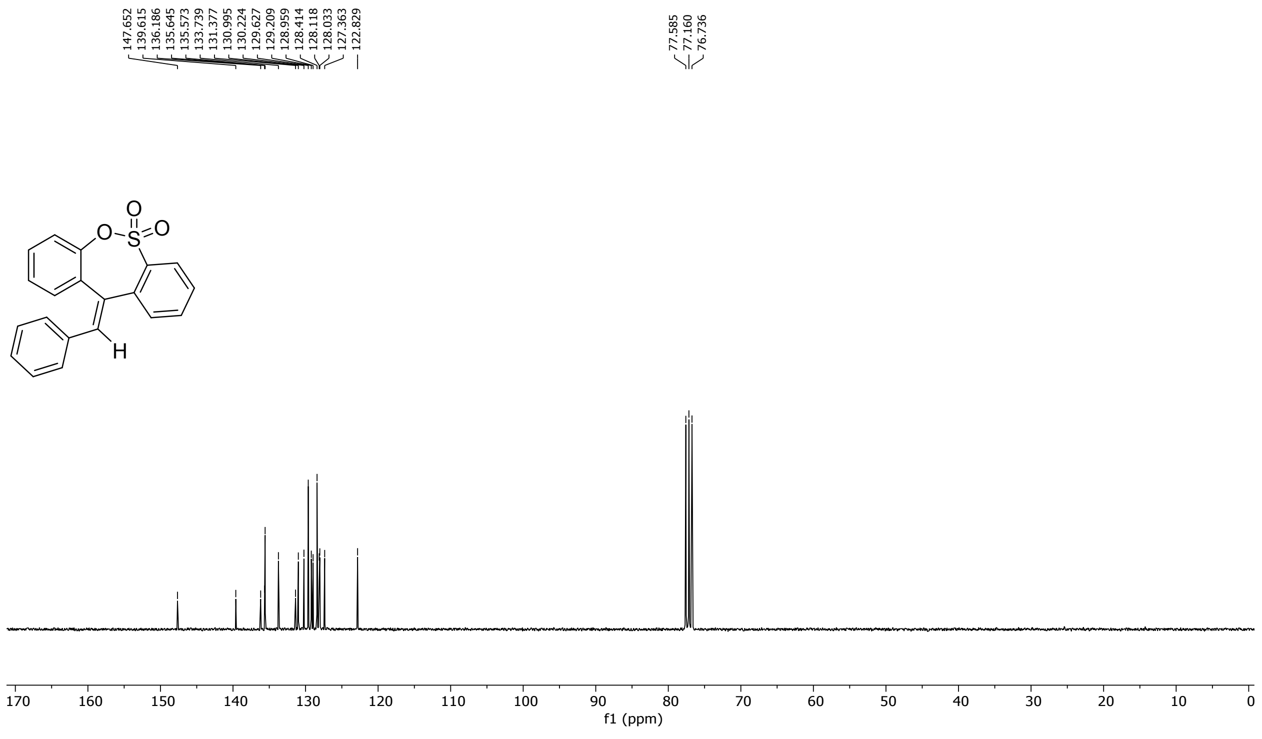

**Figure S34.** **$^{1}$H NMR (300 MHz) and $^{13}$C NMR (75 MHz) spectra of compound (*E*)-11-heptylidene-11*H*-dibenzo[*c*,*f*][1,2]oxathiepine 6,6-dioxide (3l) in $CDCl_3$.**

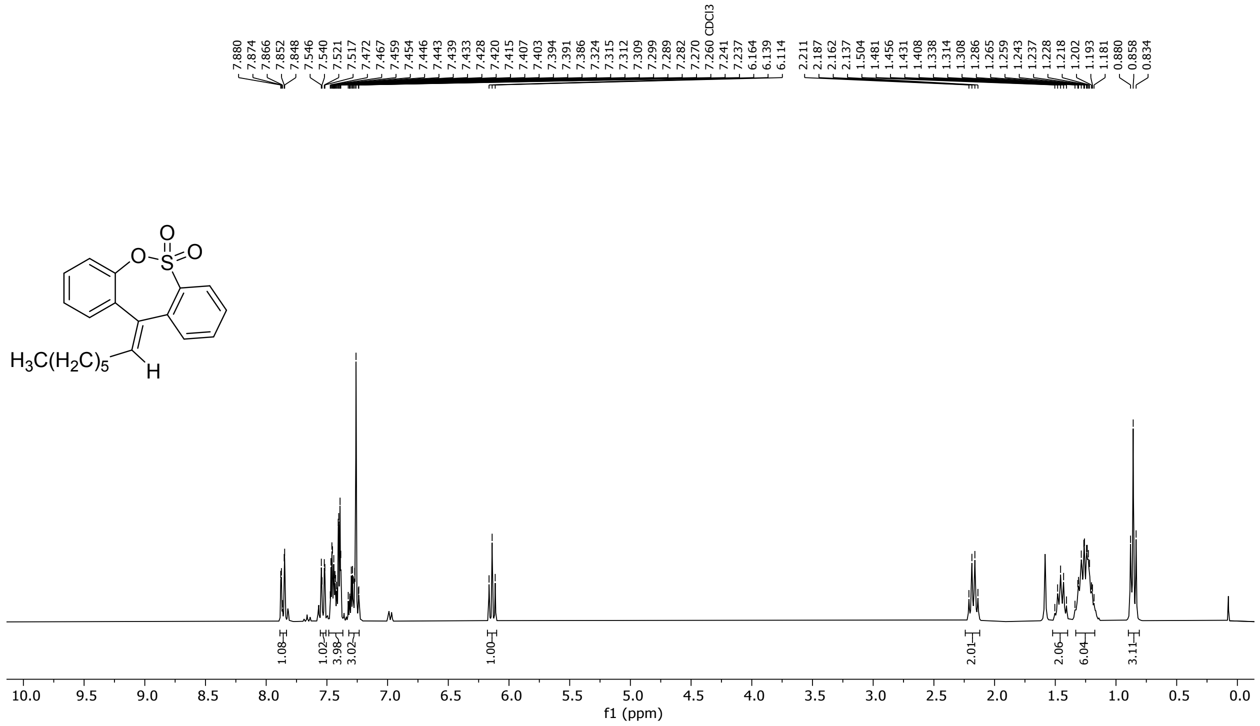


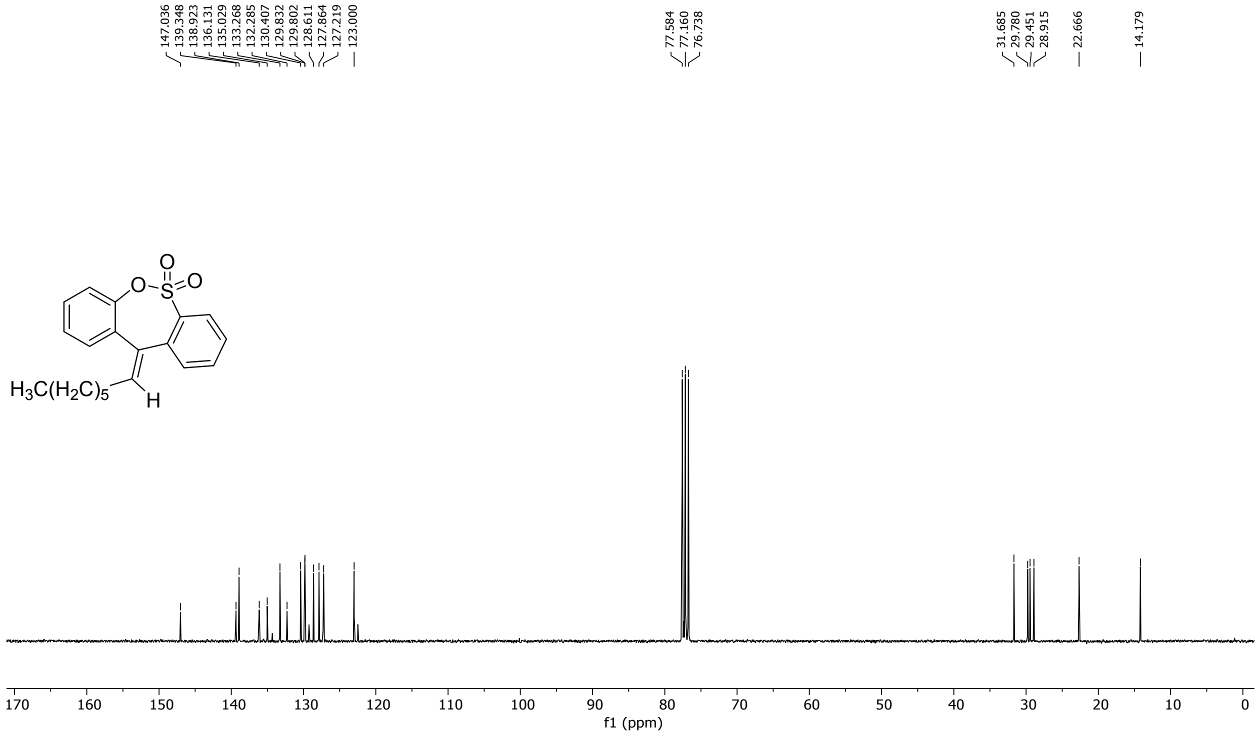

**Figure S35.** **$^1$H NMR (300 MHz) and $^{13}$C NMR (75 MHz) spectra of compound (*E*)-11-hexylidene-11*H*-dibenzo[*c*,*f*][1,2]oxathiepine 6,6-dioxide (3m) in $CDCl_3$.**

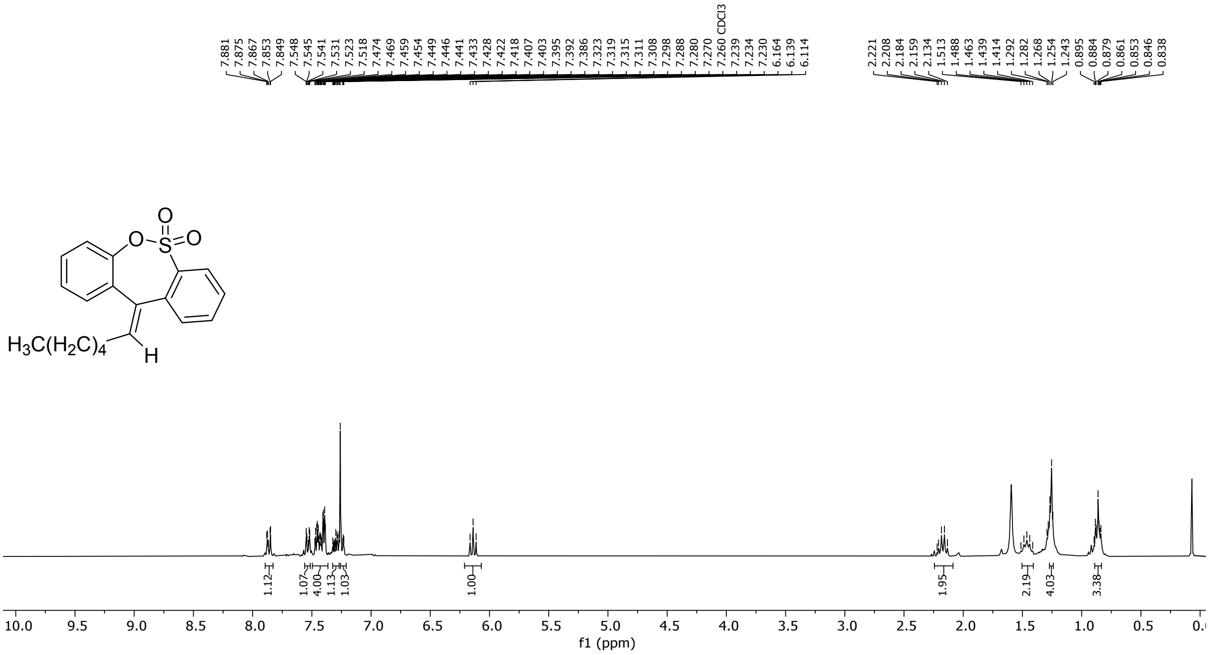


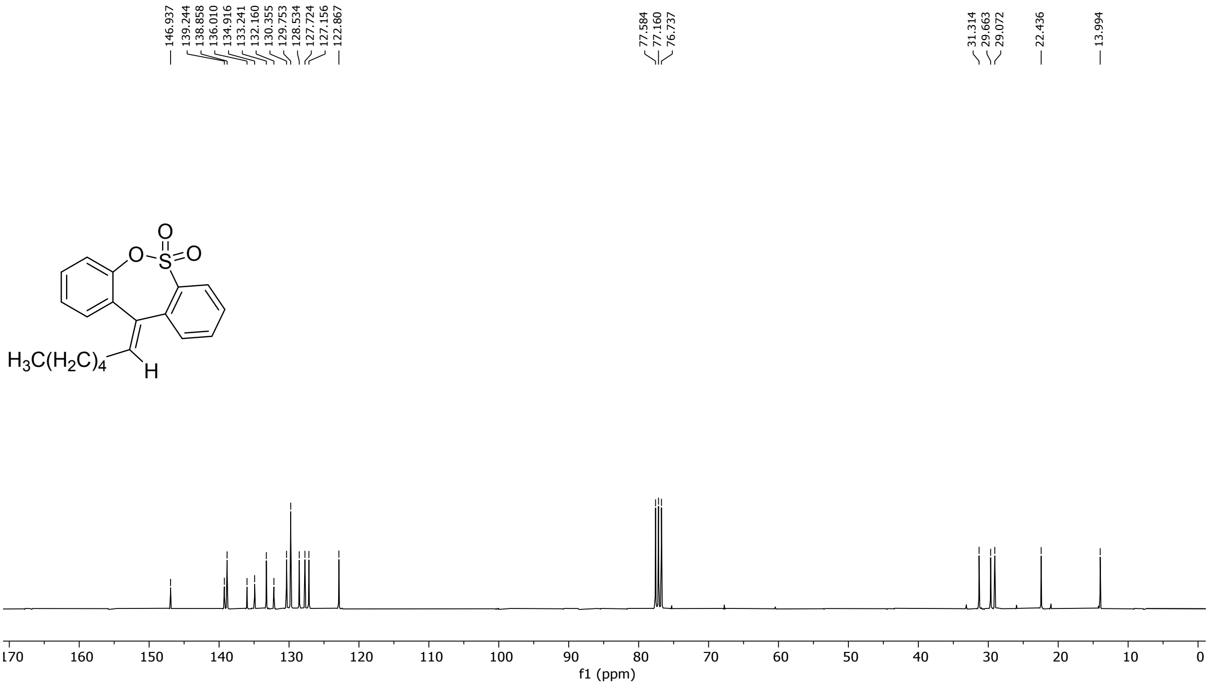

**Figure S36. $^{1}$H NMR (300 MHz) and $^{13}$C NMR (75 MHz) spectra of compound (*E*)-11-pentylidene-11*H*-dibenzo[*c*,*f*][1,2]oxathiepine 6,6-dioxide (3n) in $CDCl_3$.**

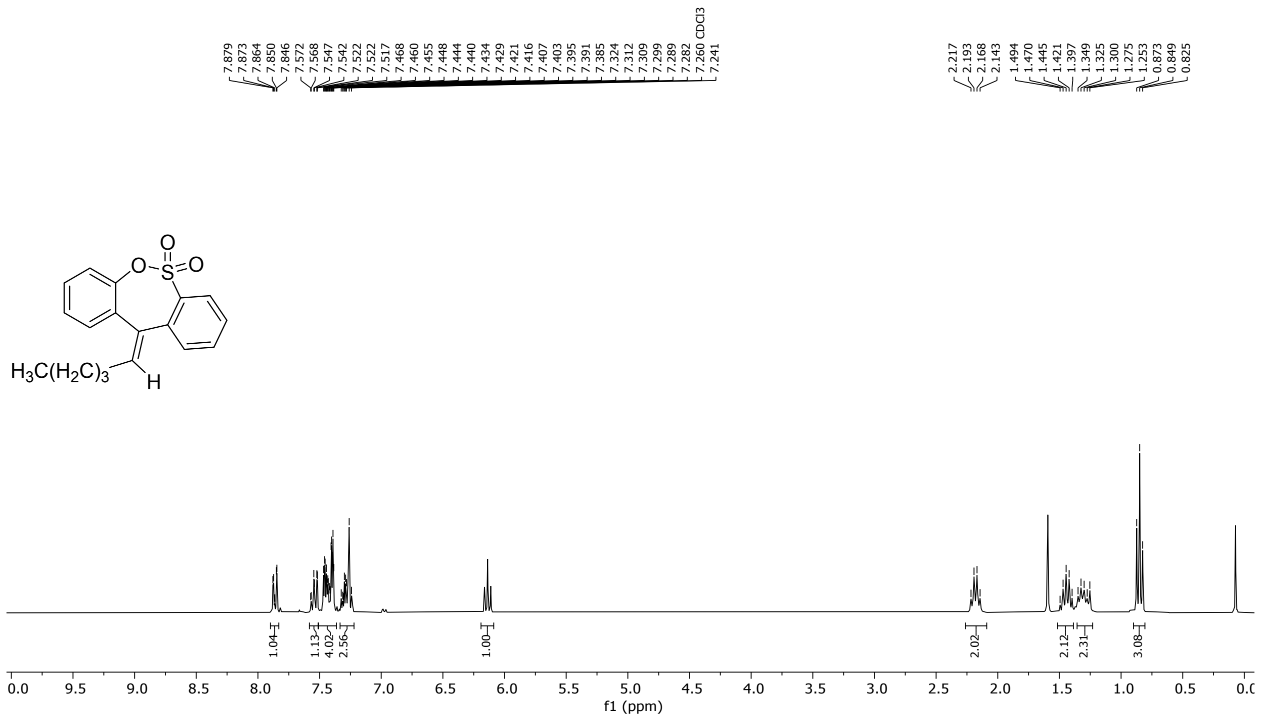

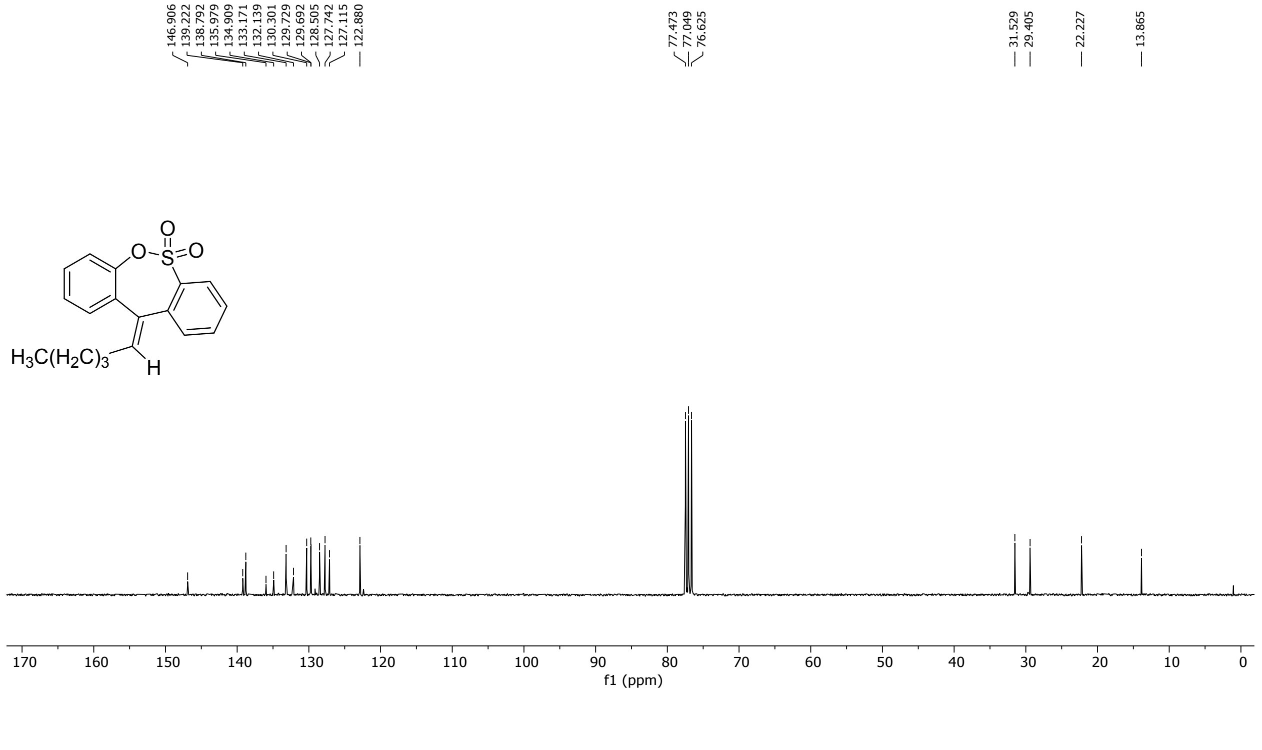

**Figure S37. $^{1}$H NMR (400 MHz) and $^{13}$C NMR (100 MHz) spectra of compound (*E*)-11-butylidene-11*H*-dibenzo[*c*,*f*][1,2]oxathiepine 6,6-dioxide (3o) in $CDCl_3$.**

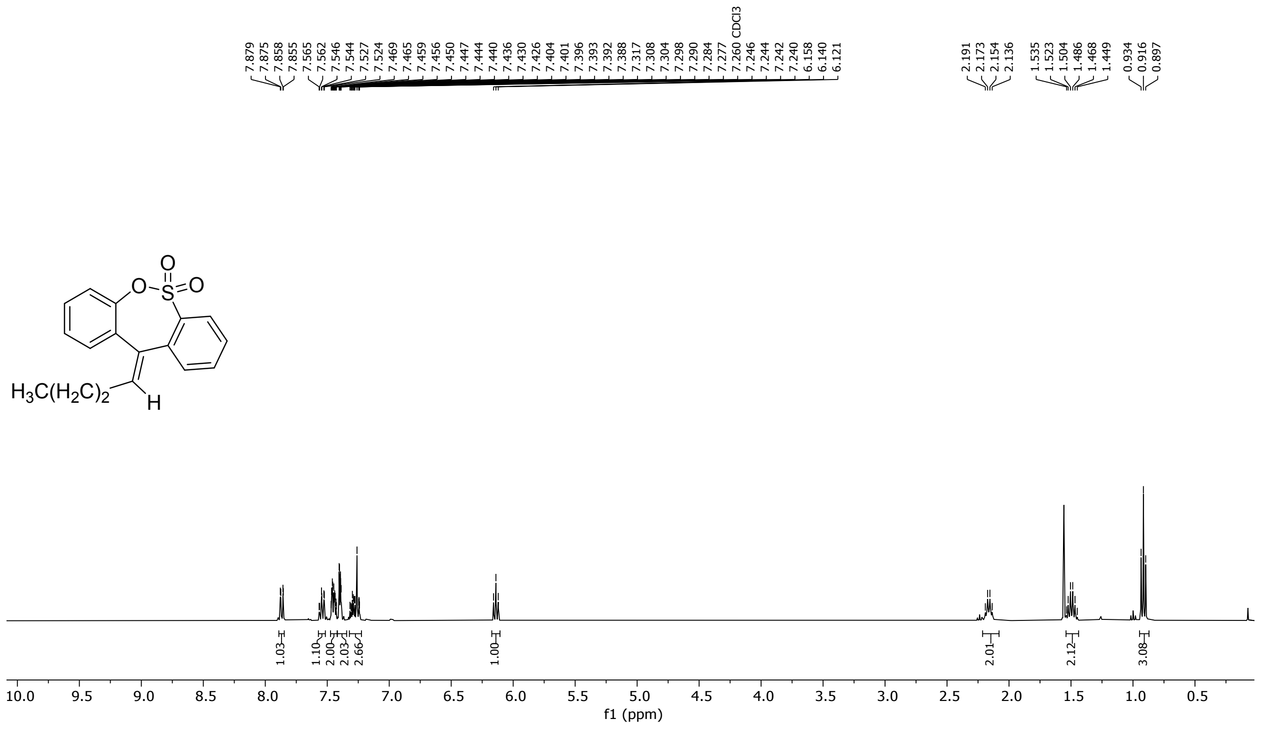

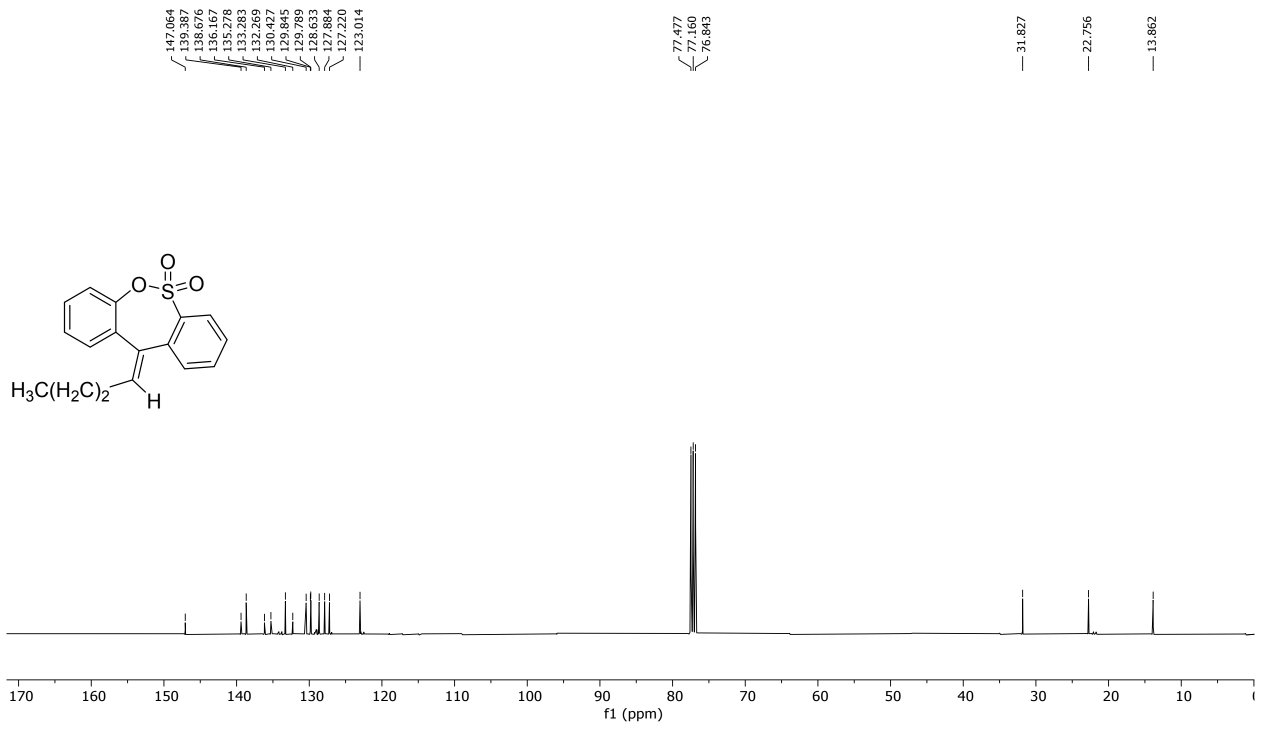

**Figure S38. ¹H NMR (300 MHz) and ¹³C NMR (75 MHz) spectra of compound (*E*)-11-(2-hydroxy-2-methylpropylidene)-11*H*-dibenzo[*c*,*f*][1,2]oxathiepine 6,6-dioxide (3p) in $CDCl_3$.**

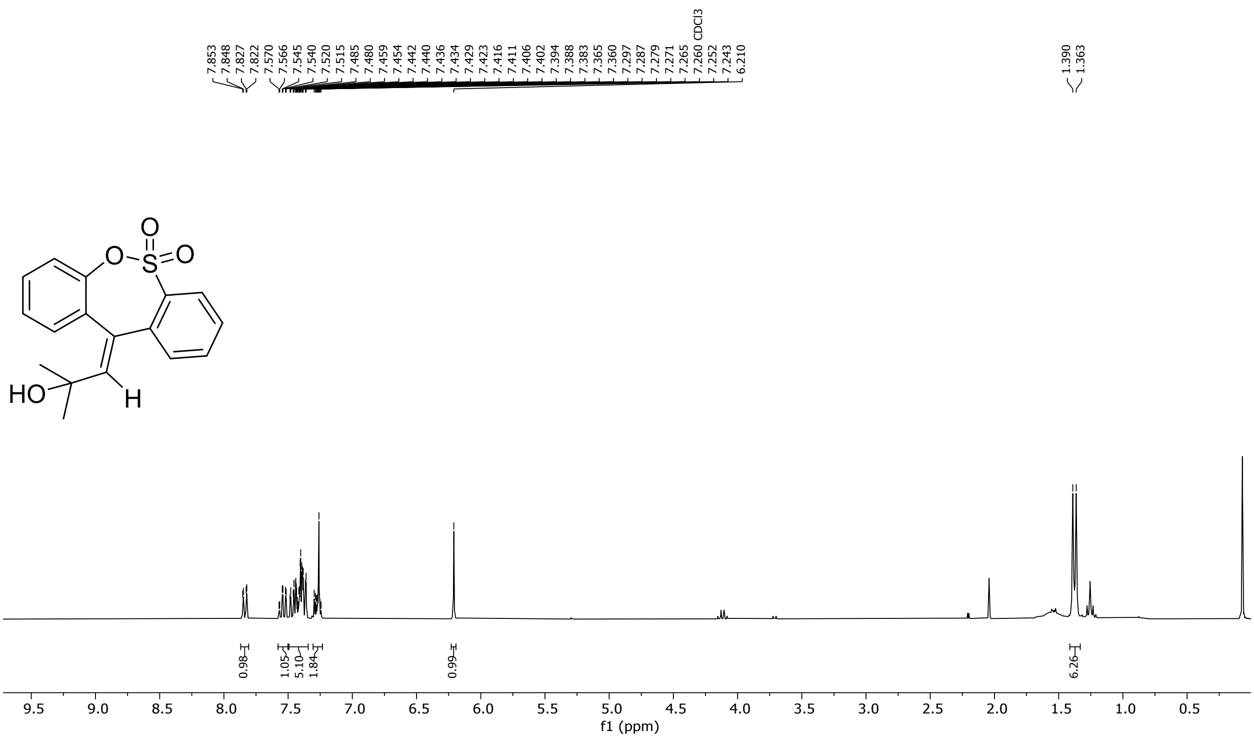


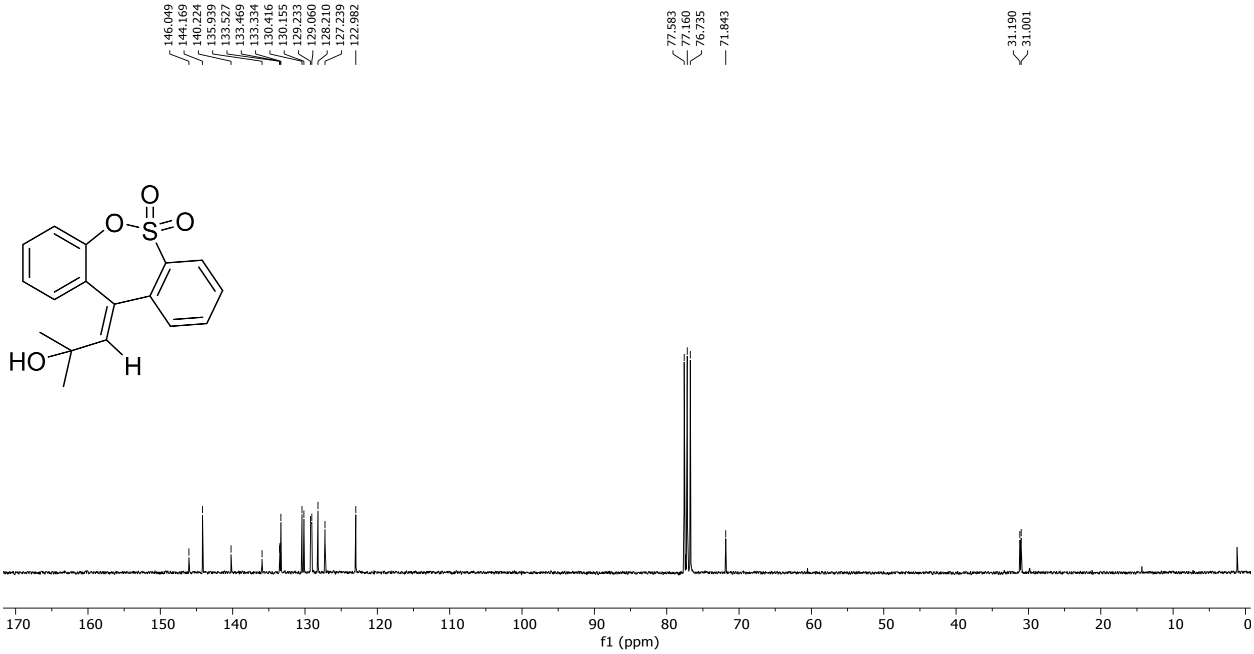

**Figure S39.** $^{1}H$ **NMR (300 MHz) and** $^{13}C$ **NMR (75 MHz) spectra of compound (*E*)-11-(4-hydroxybutylidene)-11*H*-dibenzo[*c,f*][1,2]oxathiepine 6,6-dioxide (3q) in** $CDCl_3$.

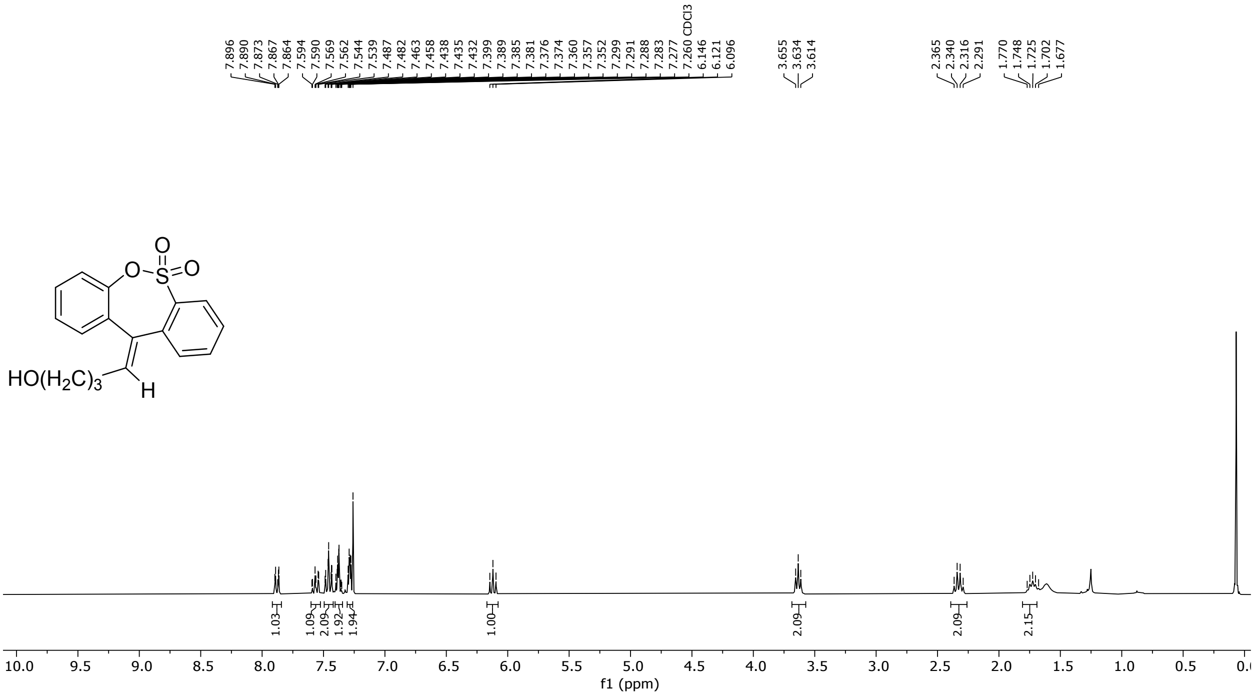


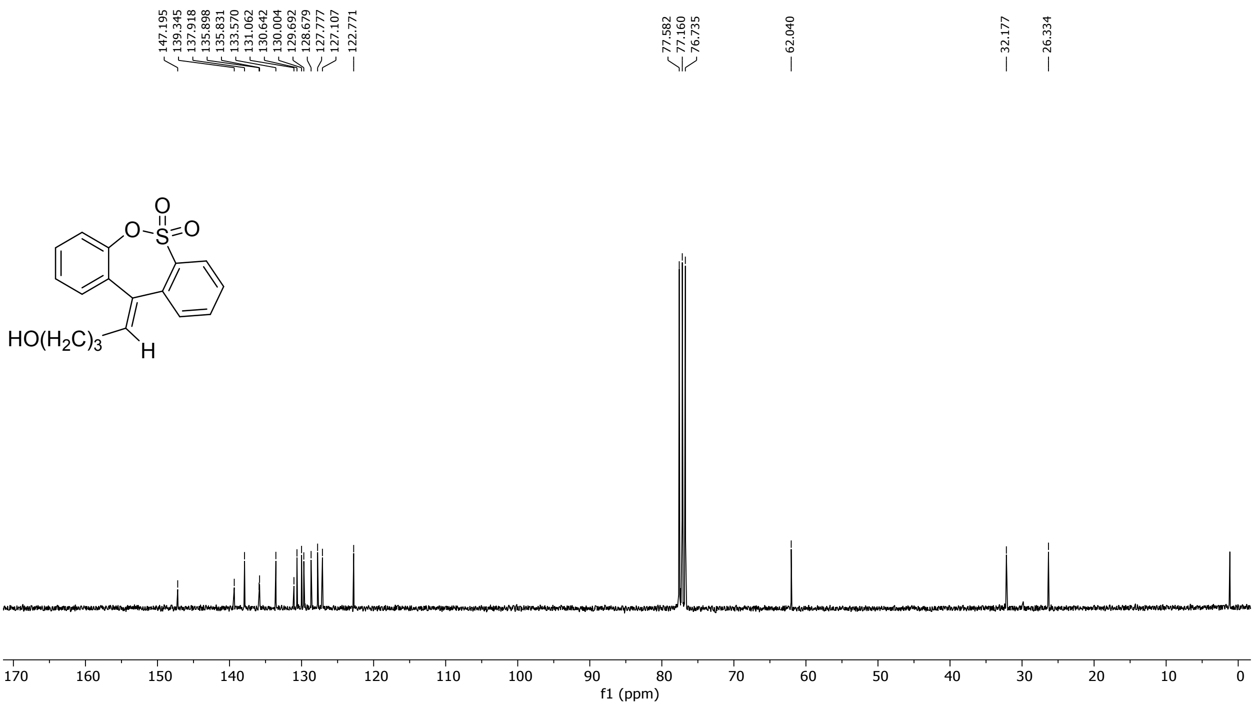

**Figure S40. $^{1}$H NMR (300 MHz) and $^{13}$C NMR (75 MHz) spectra of compound (*E*)-11-(3-hydroxypropylidene)-11*H*-dibenzo[*c*,*f*][1,2]oxathiepine 6,6-dioxide (3r) in $CDCl_3$.**

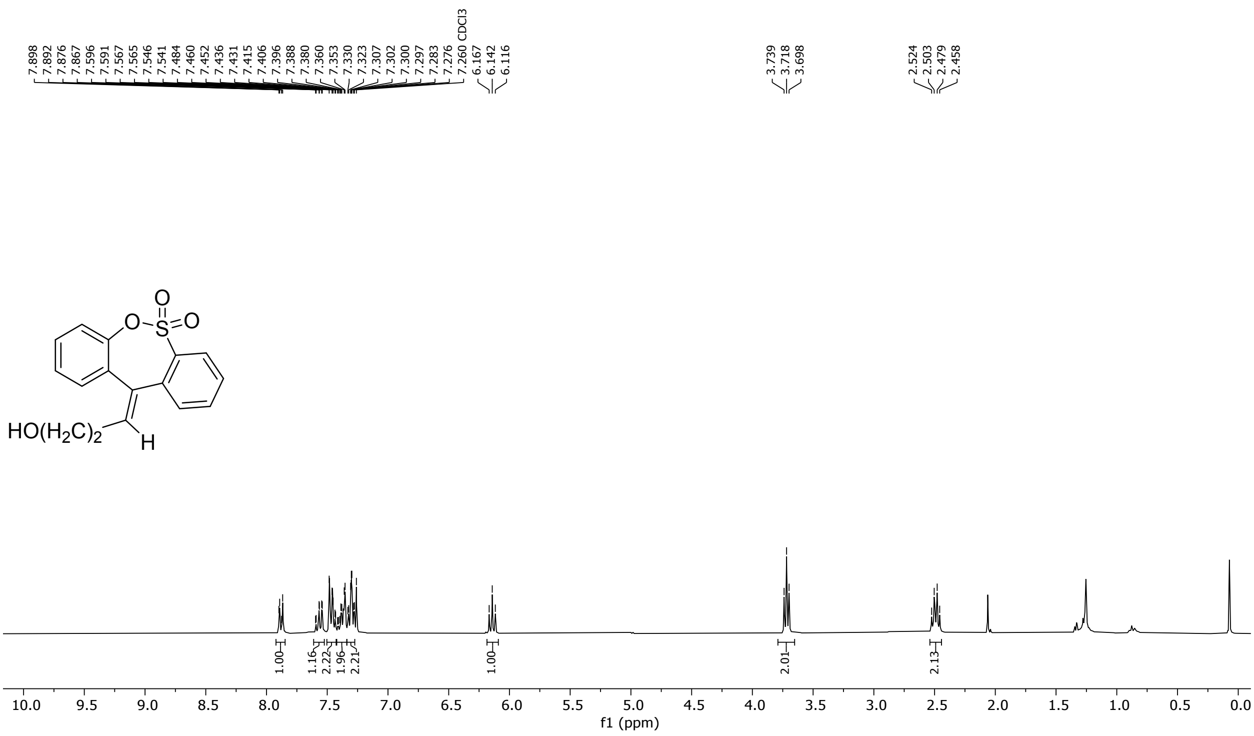


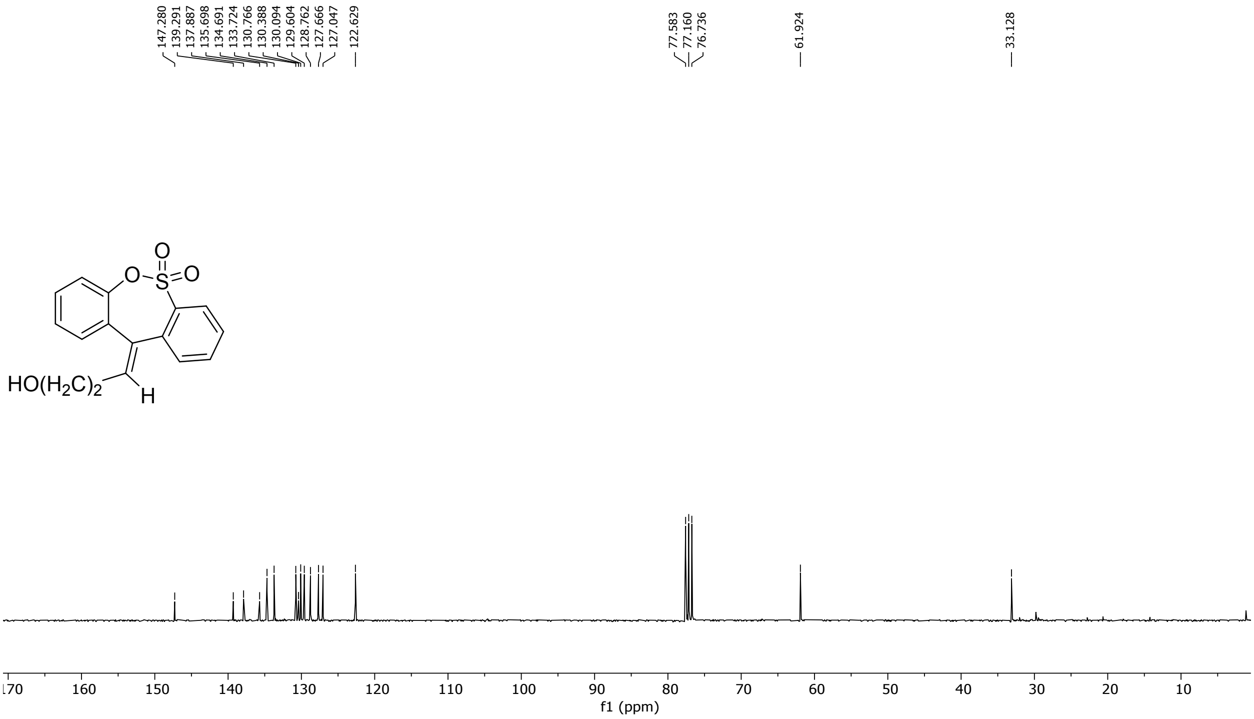

**Figure S41. $^{1}$H NMR (300 MHz) and $^{13}$C NMR (75 MHz) spectra of compound (*E*)-11-(2-hydroxyethylidene)-11*H*-dibenzo[*c*,*f*][1,2]oxathiepine 6,6-dioxide (3s) in $CDCl_3$.**

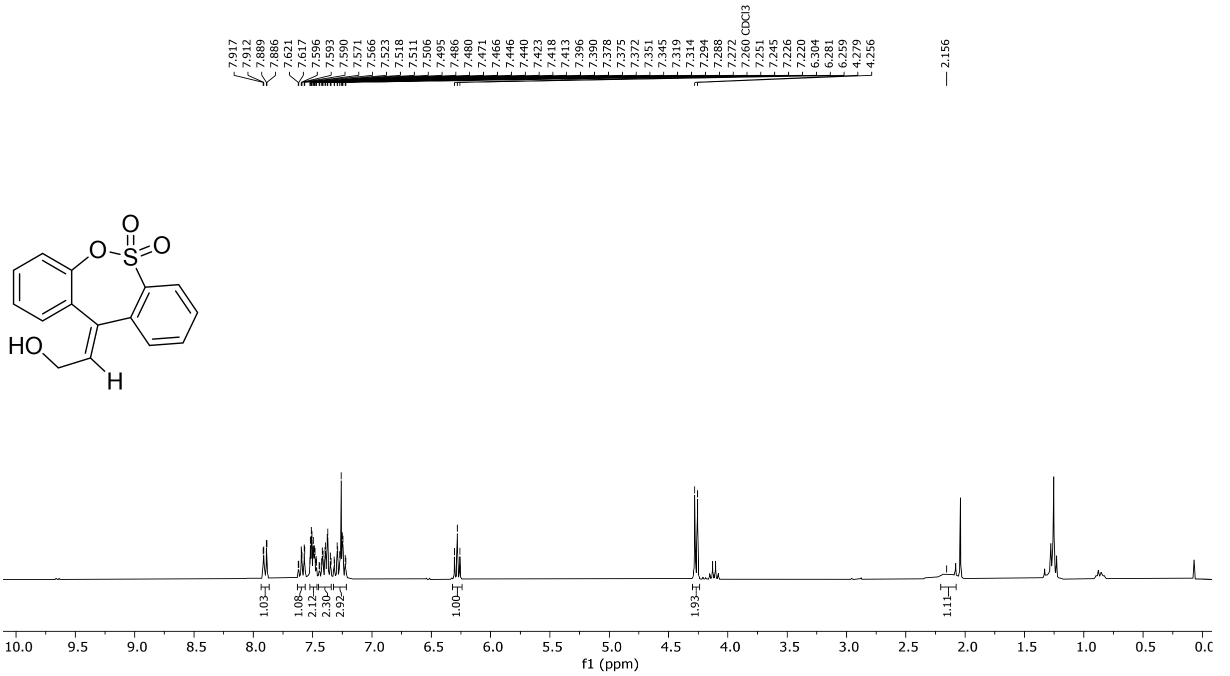


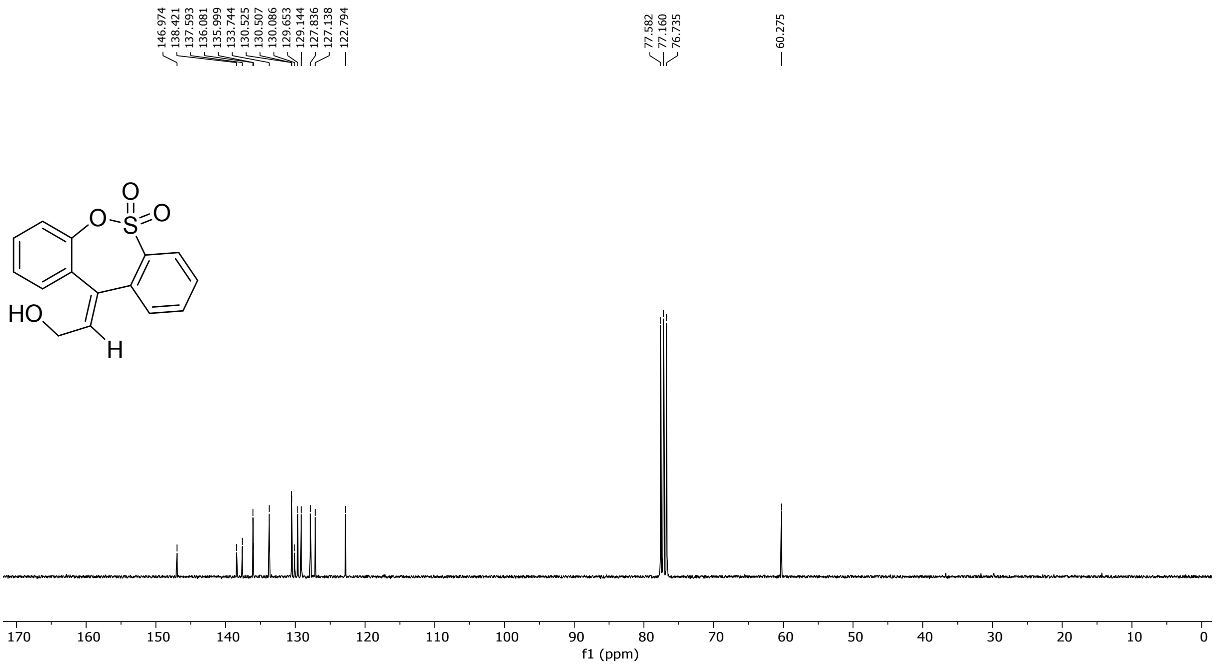

**Figure S42.** **$^1$H NMR (300 MHz) and $^{13}$C NMR (75 MHz) spectra of compound (Z)-11-benzylidene-11*H*-benzo[3,4][1,2]oxathiepino[6,7-*b*]pyridine 6,6-dioxide (3t) in $CDCl_3$.**

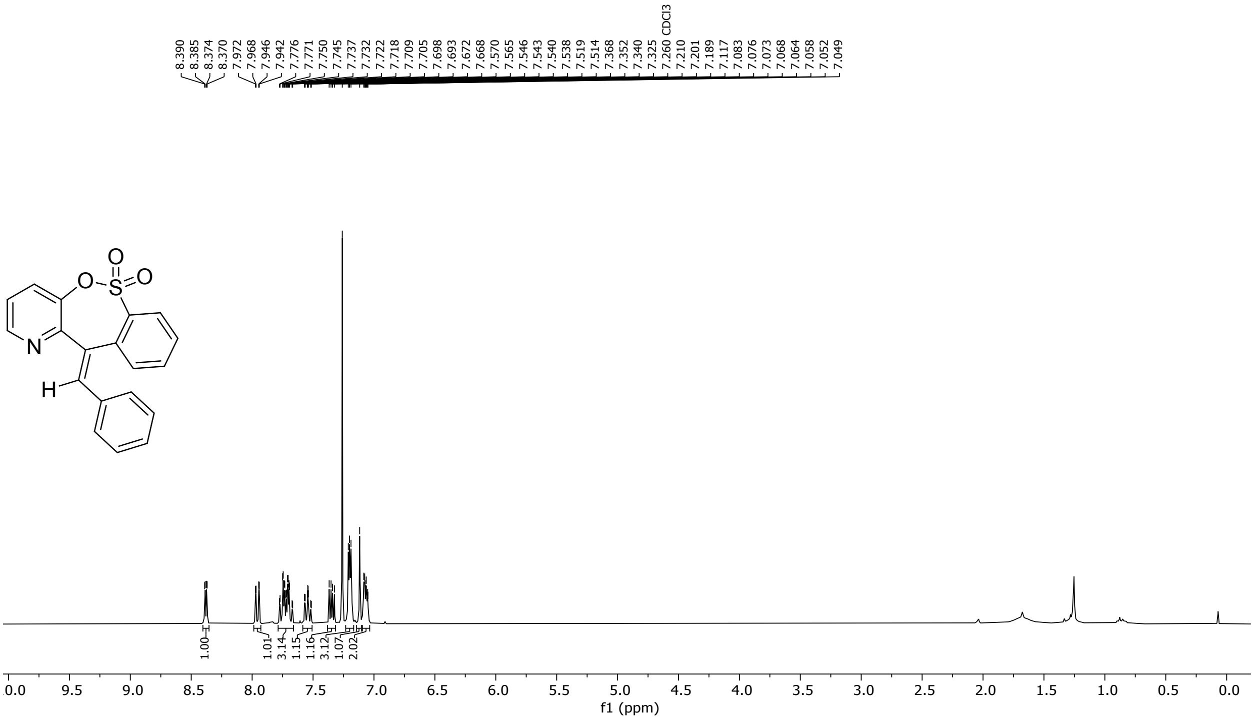


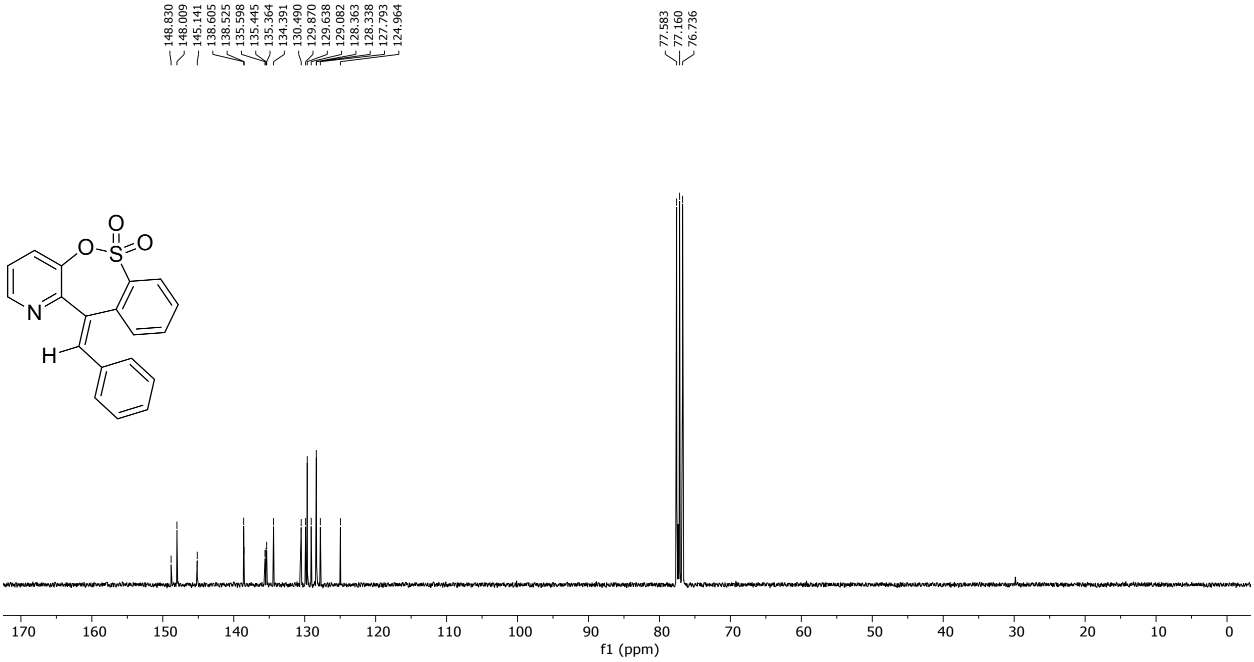

**Figure S43. $^{1}$H NMR (400 MHz) and $^{13}$C NMR (75 MHz) spectra of compound (*E*)-2-fluoro-11-(4-methoxybenzylidene)-11*H*-dibenzo[*c,f*][1,2]oxathiepine 6,6-dioxide (3u) in $CDCl_3$.**

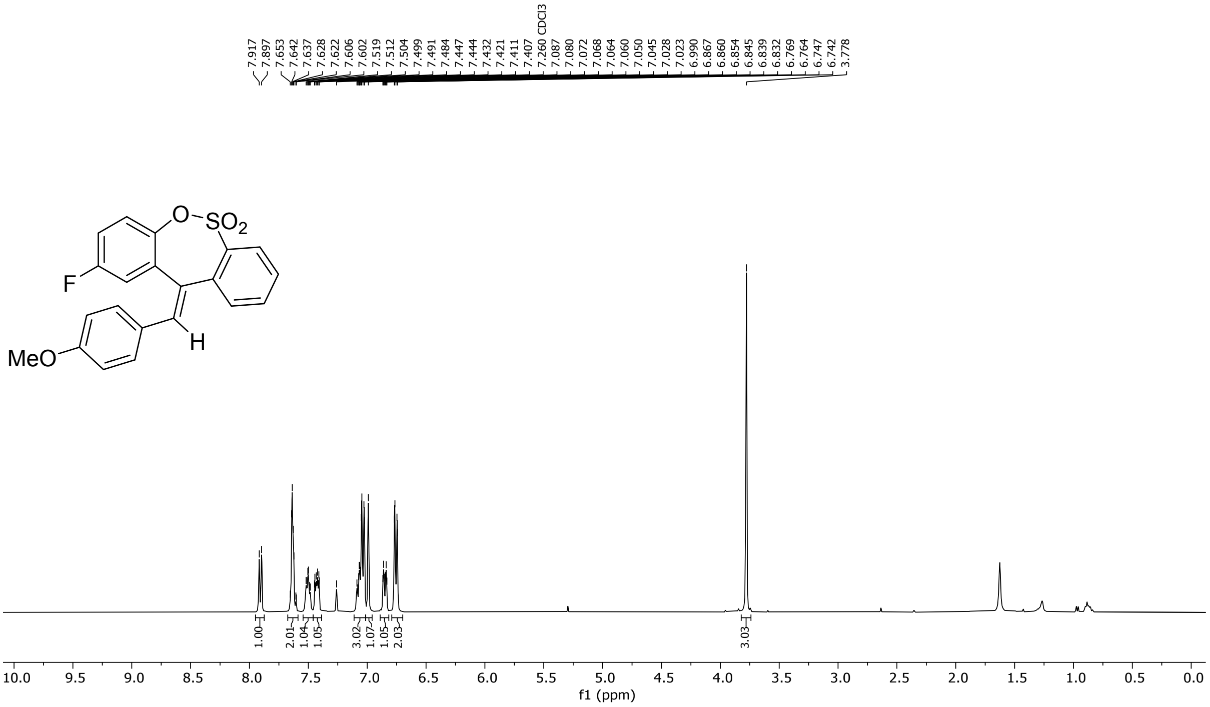


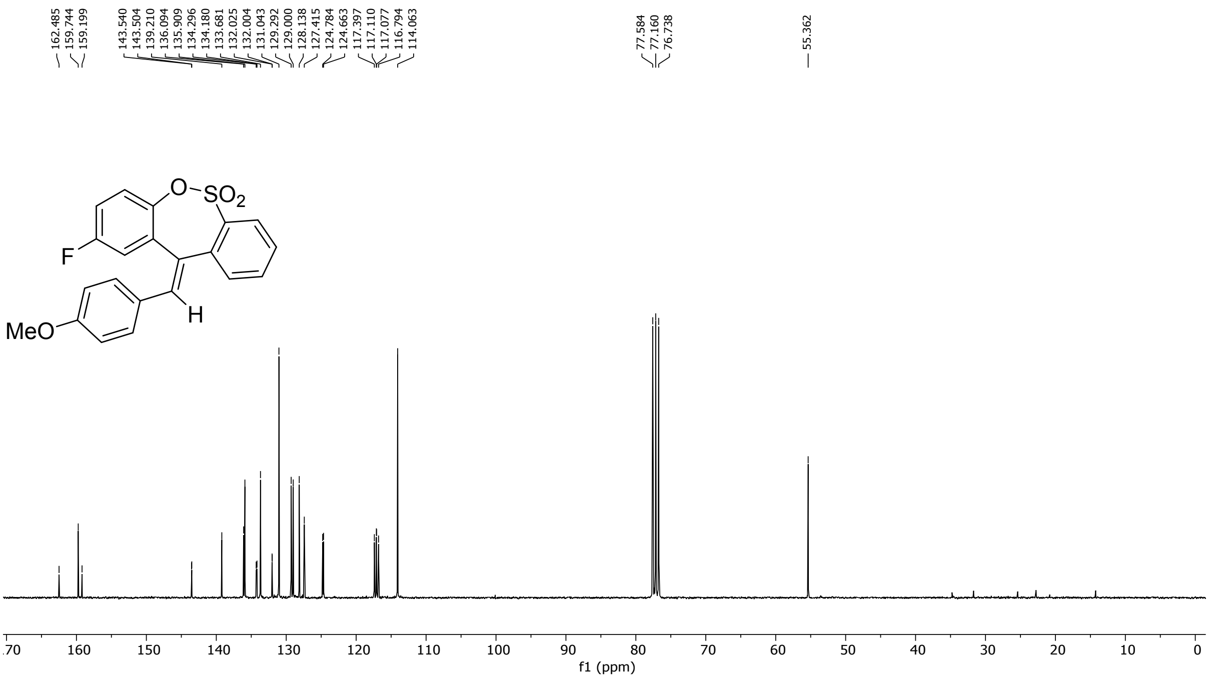

**Figure S44.** $^{1}H$ **NMR (400 MHz) and** $^{13}C$ **NMR (75 MHz) spectra of compound 11-methylene-11*H*-dibenzo[*c,f*][1,2]oxathiepine 6,6-dioxide (3v) in** $CDCl_3$.

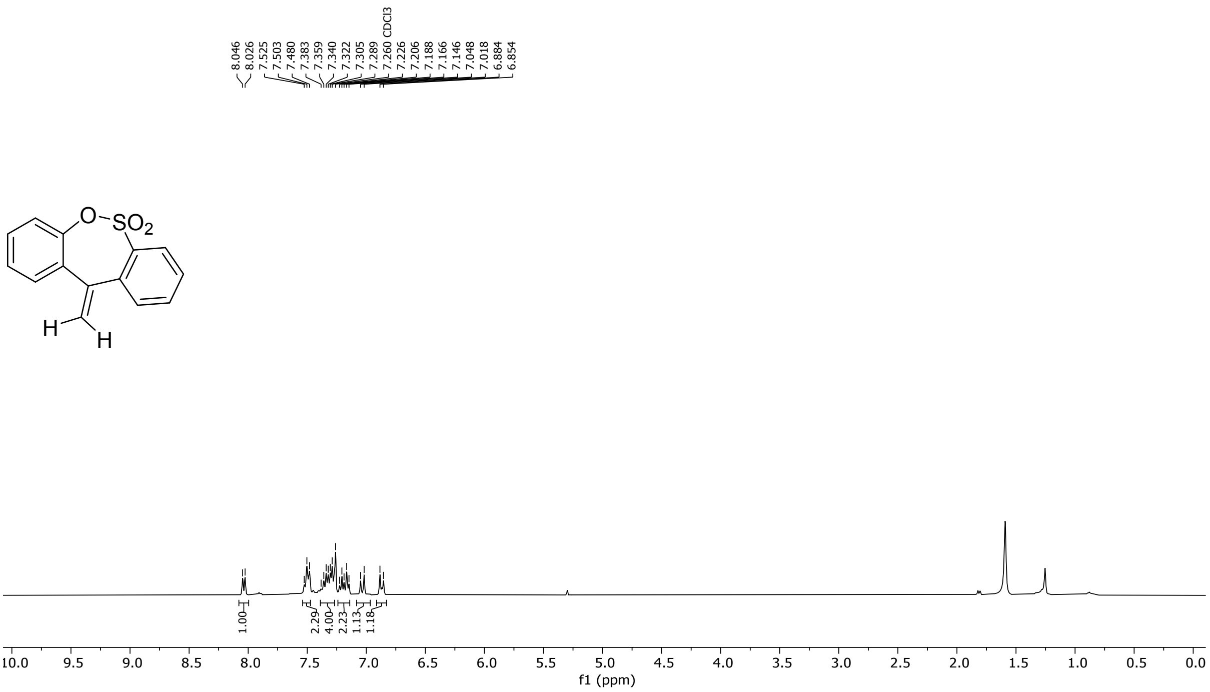


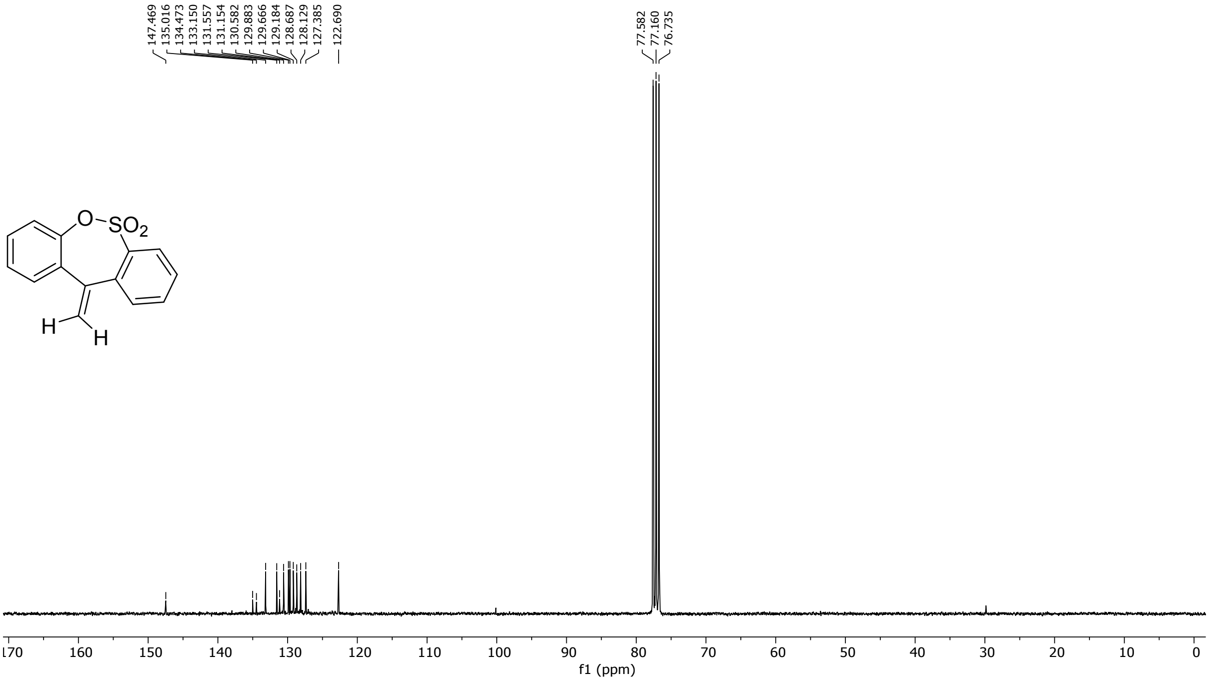

**8) $^{1}$H and $^{13}$C NMR spectra of compounds 4-6:**

**Figure S45. $^{1}$H NMR (300 MHz) and $^{13}$C NMR (75 MHz) spectra of compound (*Z*)-3-(dibenzo[*b*,*e*]oxepin-11(6*H*)-ylidene)propyl 4-methylbenzenesulfonate, 4**

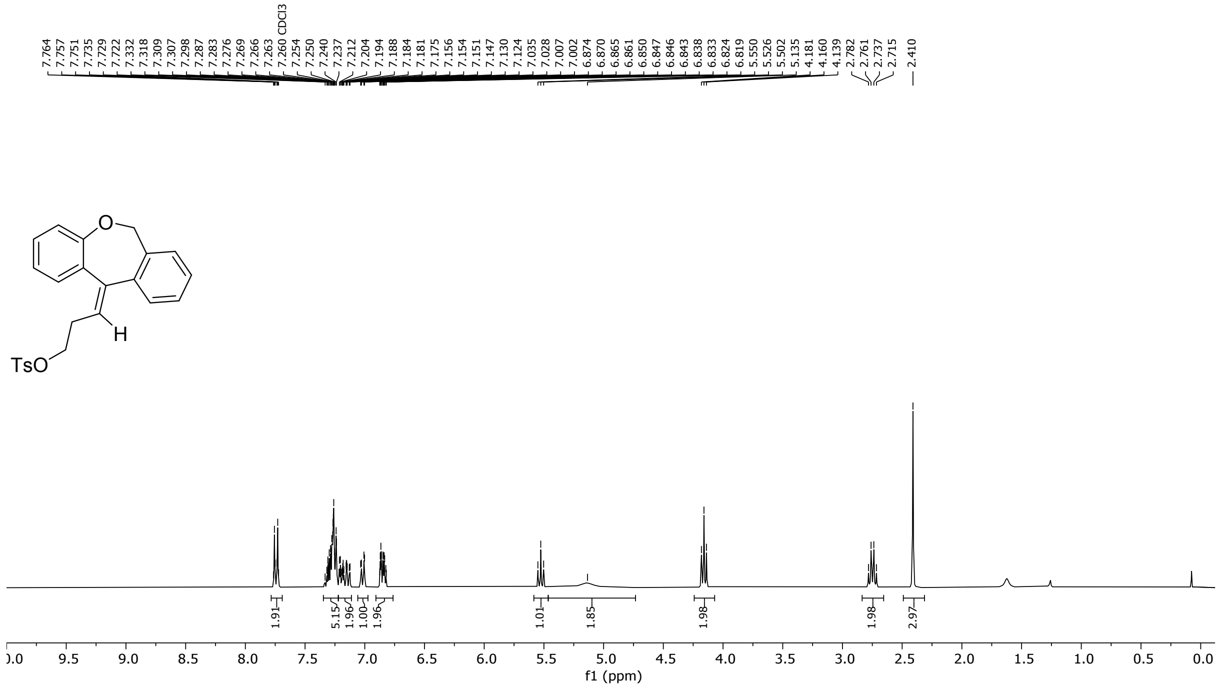

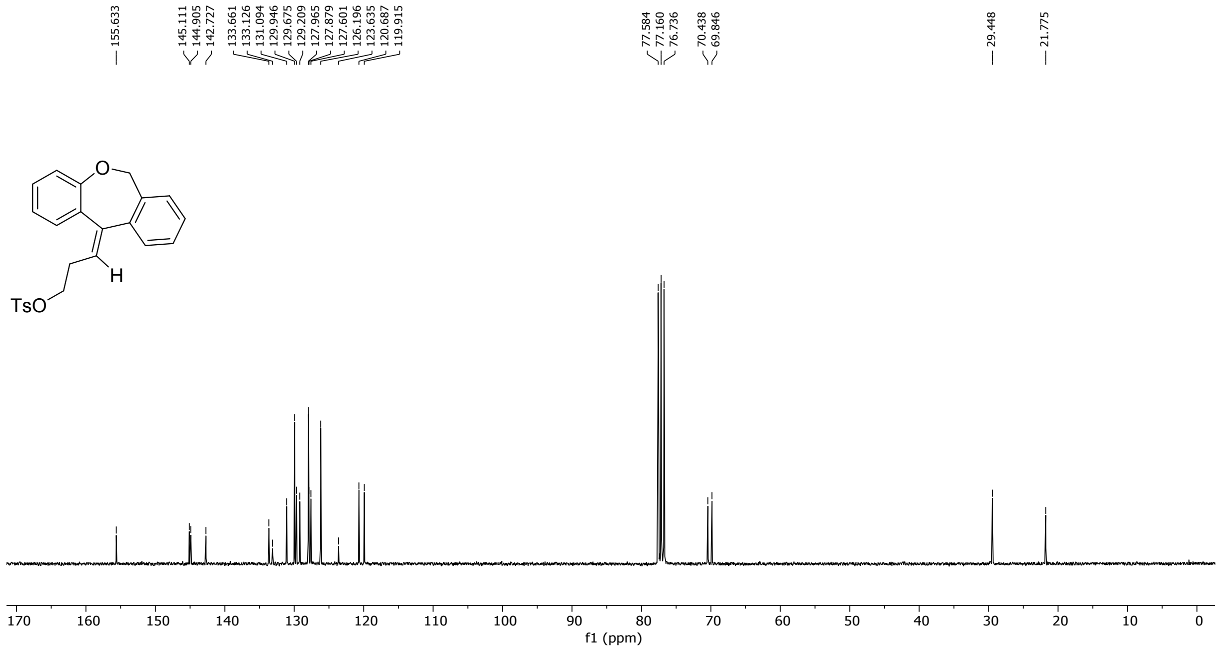

**Figure S46.** $^{1}$**H NMR (300 MHz) and** $^{13}$**C NMR (75 MHz) spectra of compound (*Z*)-3-(dibenzo[*b*,*e*]oxepin-11(6*H*)-ylidene)-*N*,*N*-dimethylpropan-1-amine, 5**

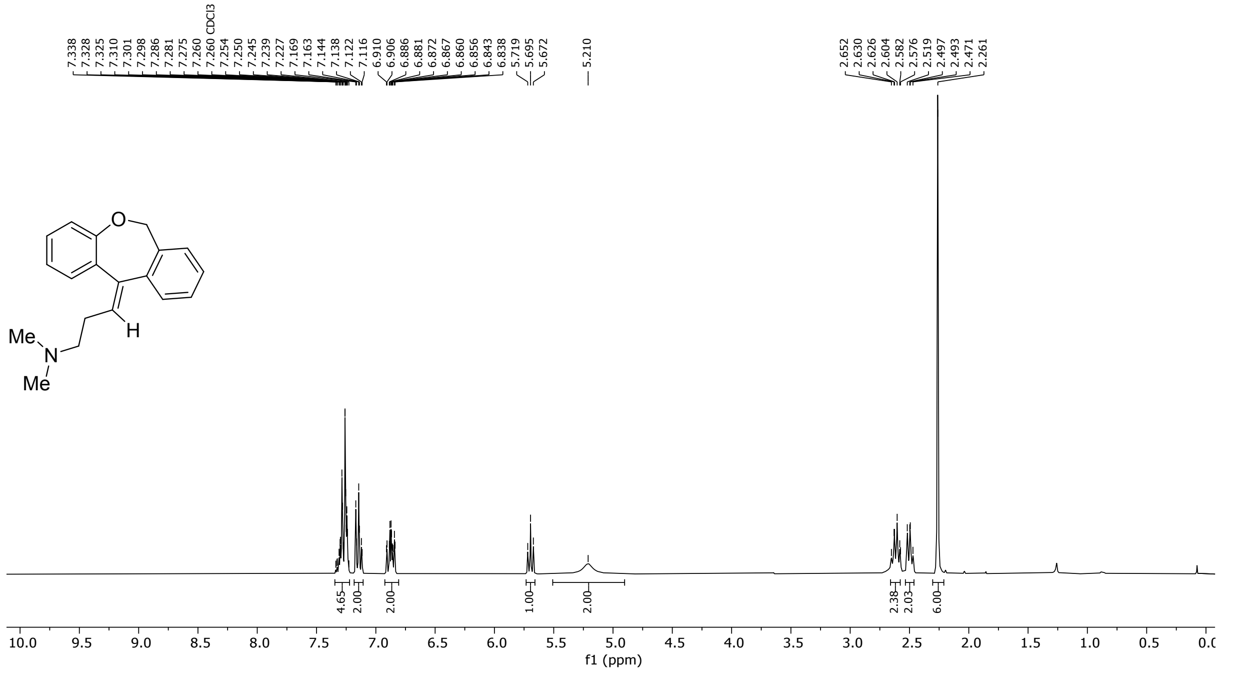

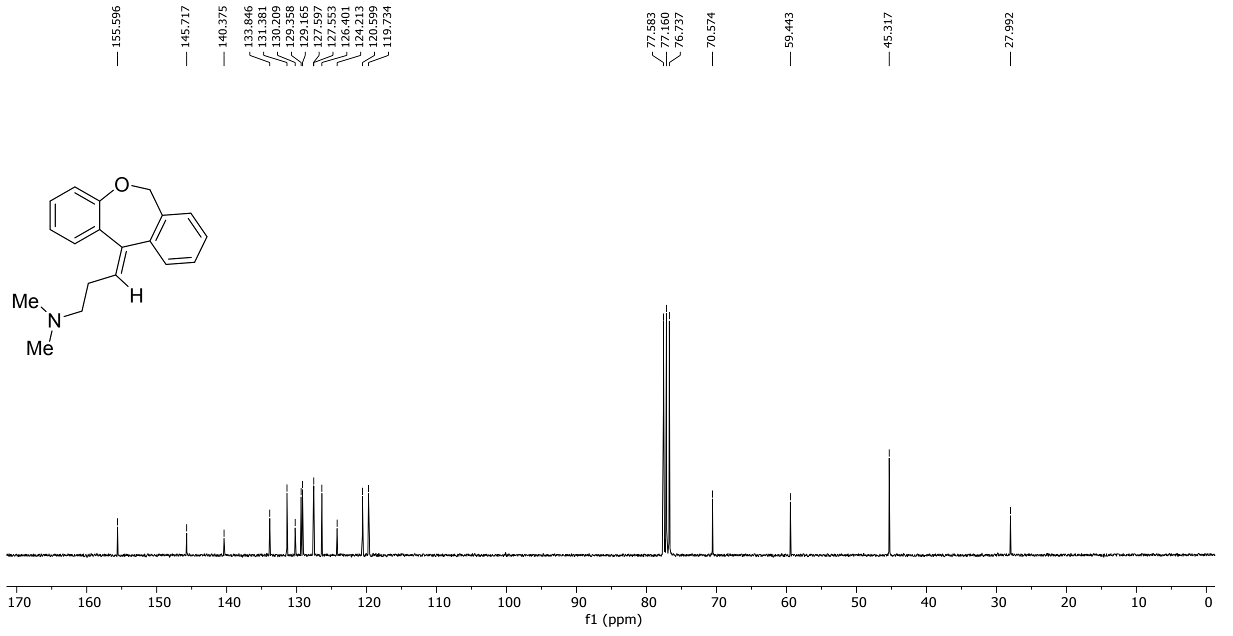

**Figure S47.** $^{1}$**H NMR (300 MHz) and** $^{13}$**C NMR (75 MHz) spectra of compound (*Z*)-3-(dibenzo[*b*,*e*]oxepin-11(6*H*)-ylidene)-*N*-methylpropan-1-amine, 6**

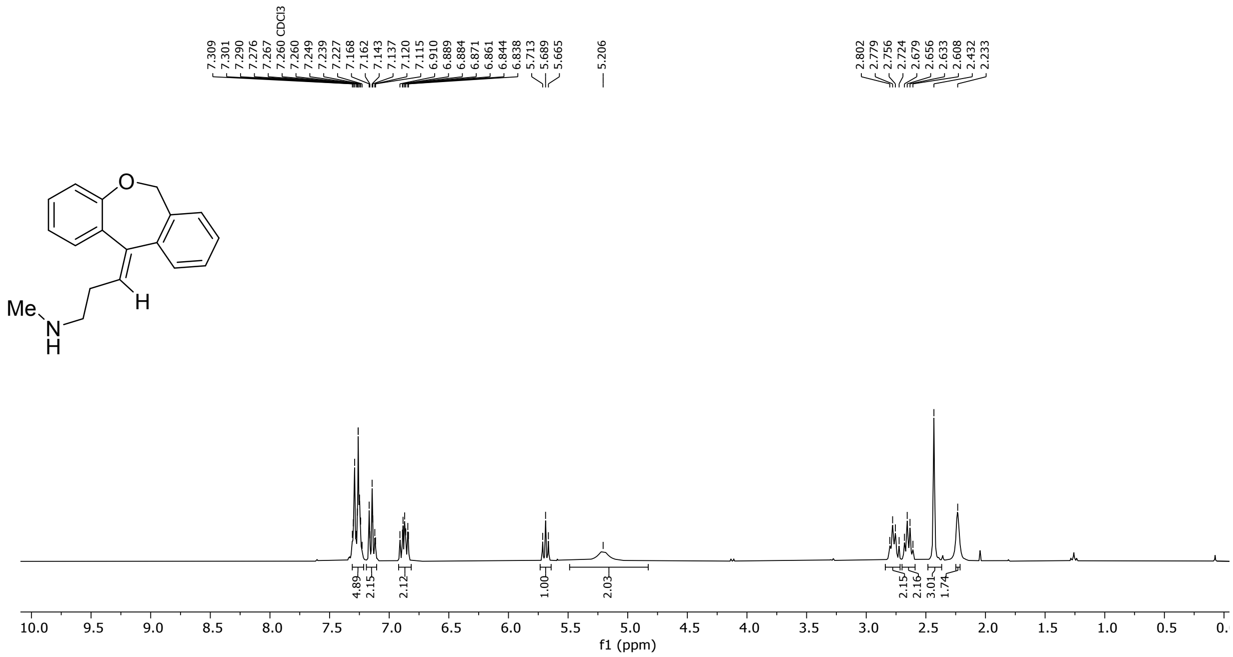


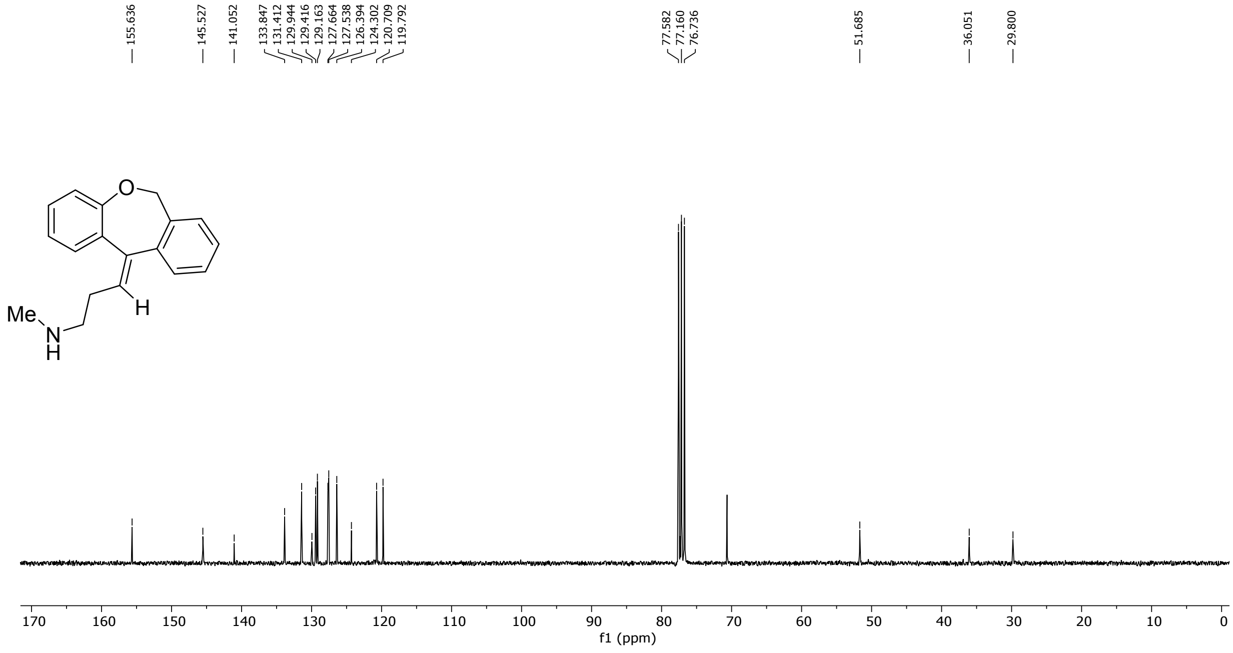

## 9) X-ray Crystal Structure of Compound 3c:

The X-ray single-crystal data for complex **3c** has been collected at room temperature in a Bruker made APEX III diffractometer. At first, single crystals of the compound **3c** have been isolated and then mounted on the glass fiber tip using commercial super glue. Mo−Kα radiation (λ = 0.71073 Å) from a sealed tube X-ray source has been used. The raw data have been integrated using the SAINT program and by utilizing SADABS, the absorption corrections were performed. The structures have been solved by SHELXL-2016/6, and full-matrix least-squares refinements on $F^2$ for all non-hydrogen atoms were performed by SHELXL-2016/6, with anisotropic displacement parameters. All the calculations and molecular graphics were done by SHELXL-2016/6, PLATON v1.15, WinGX system Ver-1.80, Mercury. All the crystallographic data and structural refinement parameters for the compound **3c** has been mentioned . CCDC 2366084 contain the supplementary crystallographic data for this paper.

**Crystallographic data of compound 3c (CCDC 2366084):**

```
Bond precision:   C-C = 0.0021 A          Wavelength=0.71073

Cell:             a=8.7200(6)     b=8.9444(6)       c=20.7592(14)
                  alpha=90        beta=95.565(2)    gamma=90
Temperature:      298 K

                 Calculated                 Reported
Volume           1611.49(19)                1611.49(19)
Space group      P 21/n                     P 21/n
Hall group       -P 2yn                     -P 2yn
Moiety formula   C22 H18 O                  ?
Sum formula      C22 H18 O                  C22 H18 O
Mr               298.36                     298.36
Dx,g cm-3        1.230                      1.230
Z                4                          4
Mu (mm-1)        0.074                      0.074
F000             632.0                      632.0
F000'            632.26
h,k,lmax         11,11,26                   11,11,26
Nref             3712                       3698
Tmin,Tmax        0.987,0.993                0.810,0.884
Tmin'            0.987

Correction method= # Reported T Limits: Tmin=0.810 Tmax=0.884
AbsCorr = MULTI-SCAN

Data completeness= 0.996          Theta(max)= 27.506

                                                    wR2(reflections)=
R(reflections)= 0.0453( 2981)                       0.1510( 3698)

S = 1.038                 Npar= 209
```

**Figure S48.** Thermal ellipsoidal (50% ellipsoid probability) structure of compound **3c**.

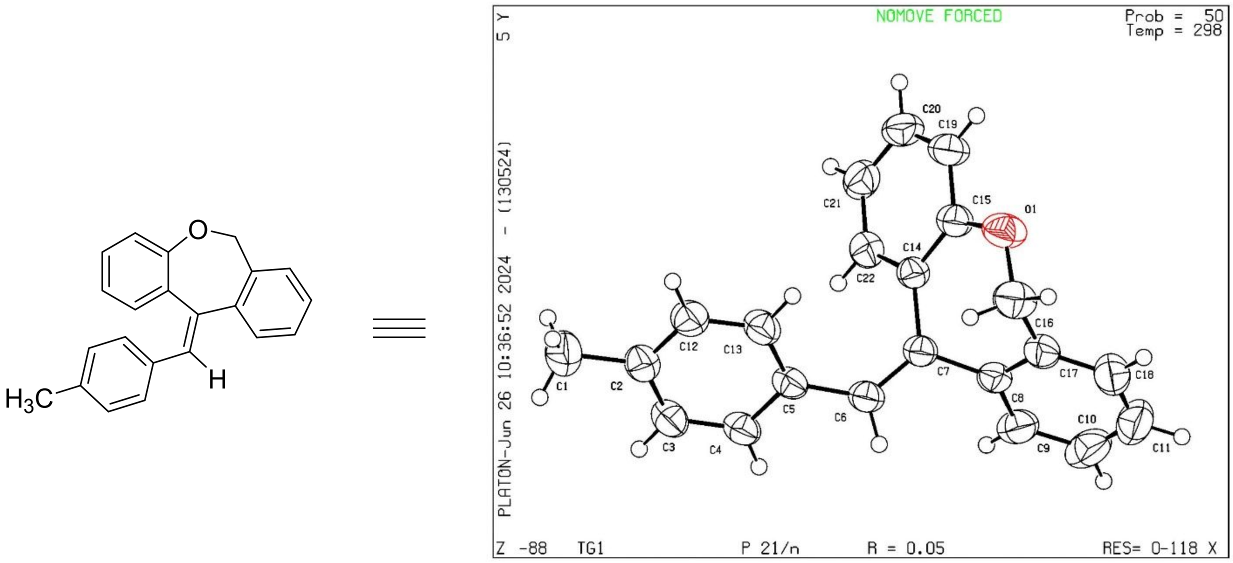

## 10) Computational analysis of reaction pathway and study of bioactivity

All the geometries examined in this study have been fully optimized using the dispersion-corrected PBE0-D3[2-3] functional with the def2-TZVPP basis set.[4] Solvent effects were taken into account using the COSMO solvation model[4] with n,n-dimethyl formamide as the solvent medium. Vibrational frequencies of each stationary point were computed at the same level of theory to categorize the stationary points as either real minima (with no imaginary frequencies) or as transition states with only one imaginary frequency. All the calculations were conducted using Gaussian 16.[5]

Molecular docking was conducted using the Schrodinger Suite molecular modeling package (version 2021-3) with the default parameters. The crystal structures of the Histamine H1 receptor (H1R) (PDB: 8X5Y)[7] with a resolution of 3.00 Å and human butyrylcholinesterase (BuChE) (PDB: 5LKR)[8] with a resolution of 2.52 Å were processed using the Protein Preparation Wizard. This involved assigning force field atom types and bond orders, adding missing atoms, determining tautomer/ionization states, and adjusting the tautomers of ionizable residues (Asn, Gln, and His residues) to optimize the hydrogen bond network. Following this, hydrogen-constrained energy minimization was performed. Glide SP docking was then used to allow full flexibility of ligands into the active site.[9-10] Afterward, a post-docking minimization was carried out, whereby only the ligands were made flexible, on the output complexes. Finally, the binding energies for each ligand were calculated.

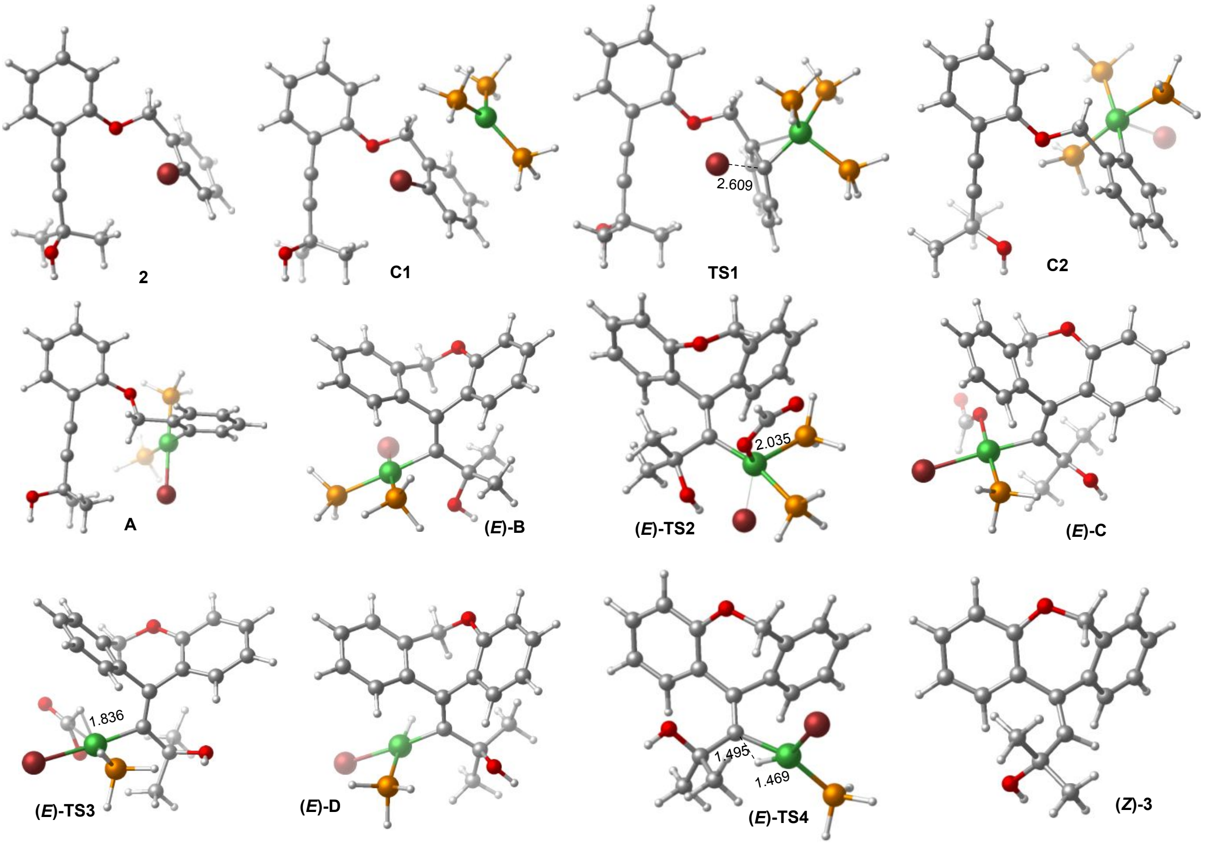


**Figure S49.** The optimized geometries of stationary points for forming the final product dibenzo[*b*,*e*]oxepine along with regeneration of the active catalyst. The distances are given in Å. [C: grey, O: red, P: orange, H: white, Br: reddish brown, Ni: green].

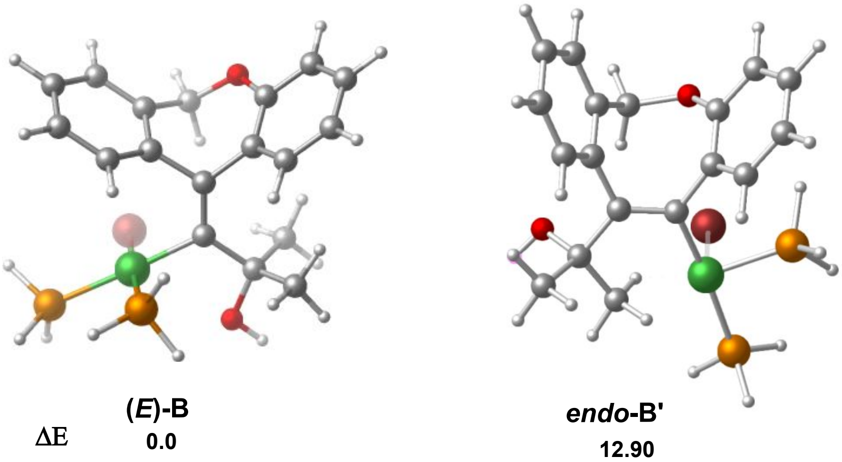


**Figure S50**. The optimized geometries of exo-cyclic and endo-cyclic nickel species for forming the final product dibenzo[*b*,*e*]oxepine. The relative free energies are given in kcal/mol. [C: grey, O: red, P: orange, H: white, Br: reddish brown, Ni: green].

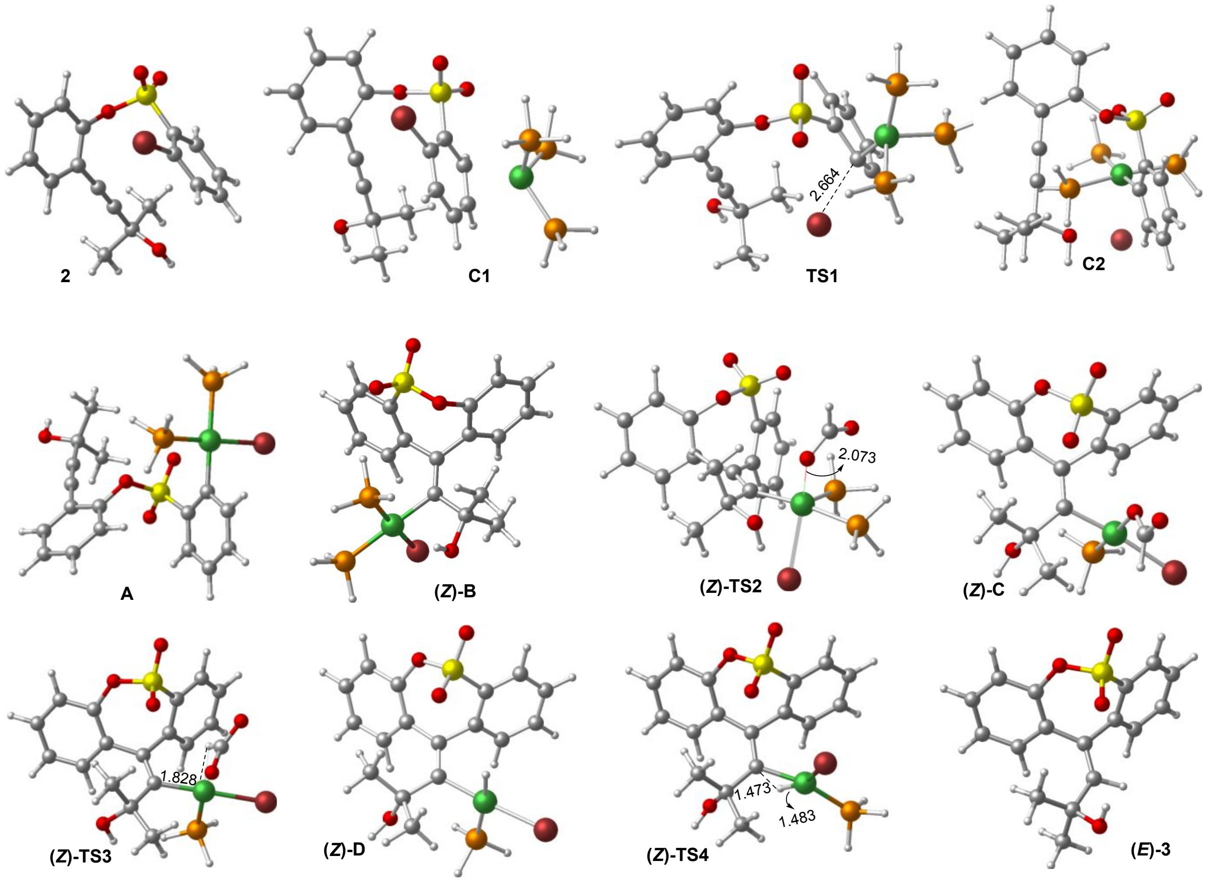


**Figure S51**. The optimized geometries of stationary points for forming the final product sultone along with regeneration of the active catalyst. The distances are given in Å. [C: grey, O: red, P: orange, H: white, Br: reddish brown, S: yellow, Ni: green].

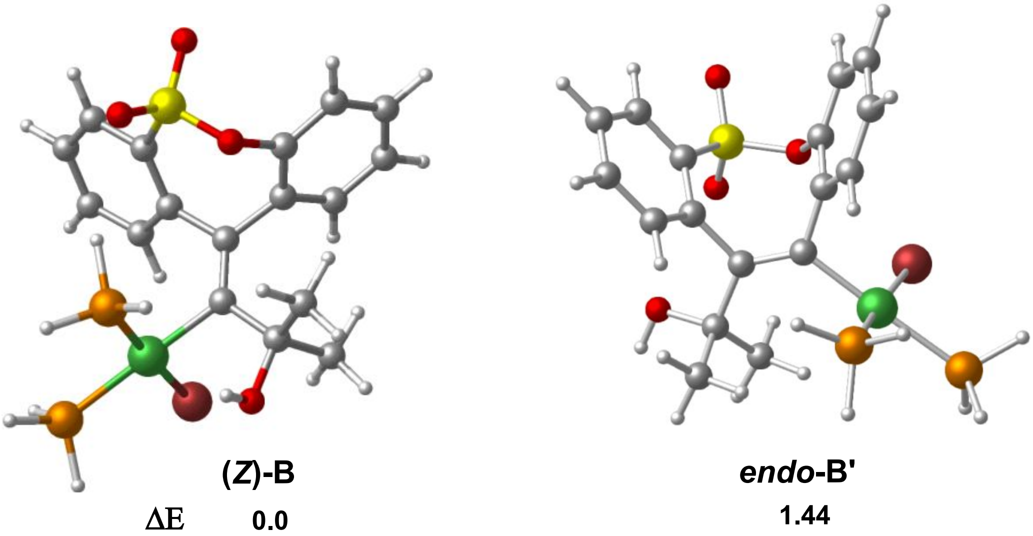


**Figure S52**. The optimized geometries of exo-cyclic and endo-cyclic nickel species for forming the final product sultone. The relative energies are given in kcal/mol. [C: grey, O: red, P: orange, H: white, Br: reddish brown, S: yellow, Ni: green].

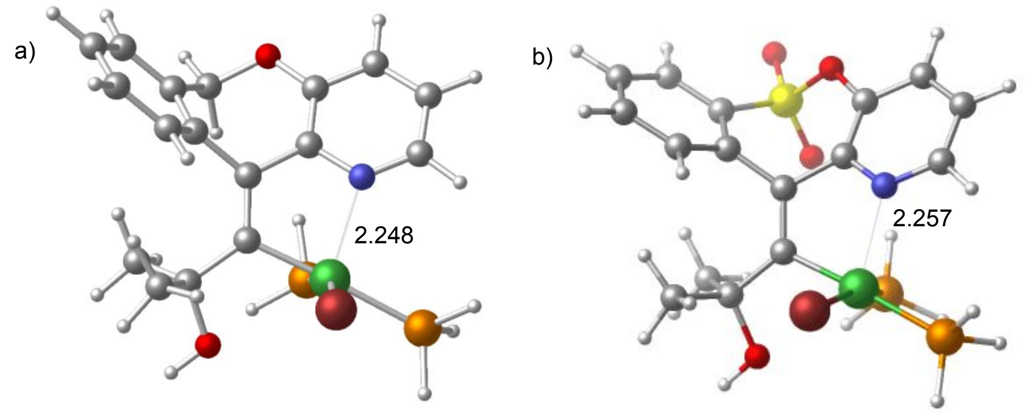


**Figure S53**. The optimized geometries of nickel species of a) pyridine-fused benzoxepine, and b) pyridine-fused sultones. The distances are given in Å. [C: grey, O: red, P: orange, H: white, Br: reddish brown, S: yellow, Ni: green].

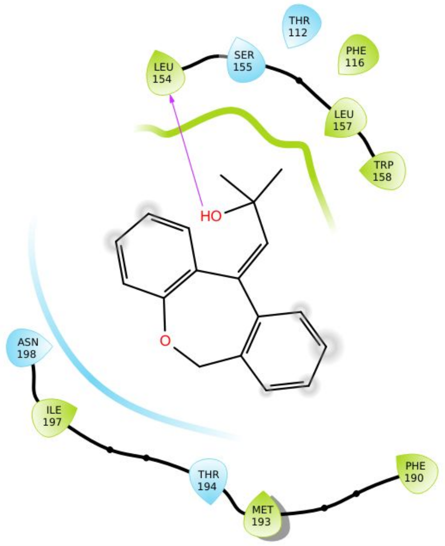


**Figure S54.** Protein-ligand schematic interaction diagram of the protein (PDB ID: 8X5Y) with dibenzo[*b*,*e*]oxepine.

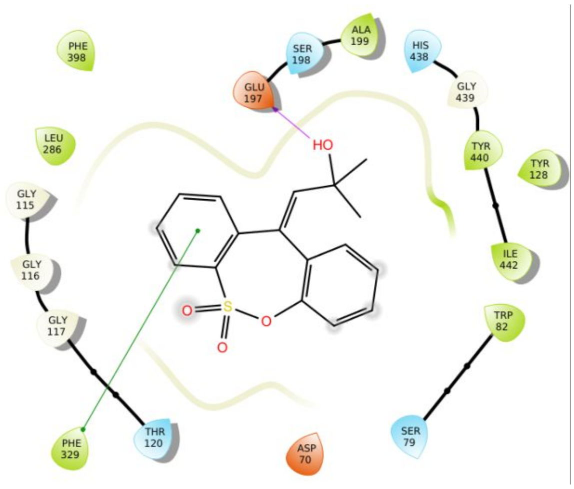


**Figure S55.** Protein-ligand schematic interaction diagram of the protein (PDB ID: 5LKR) with sultone.

## References:


1. Mondal, S.; Debnath, S.; Das, B. Synthesis of seven-membered fused sultones by reductive Heck cyclization: an investigation for stereochemistry through DFT study. *Tetrahedron* **2015**, *71*, 476-486.
2. Adamo, C.; Barone, V. Toward reliable density functional methods without adjustable parameters: The PBE0 model. *J. Chem. Phys*. **1999**, *110*, 6158-6170.
3. Grimme, S.; Antony, J.; Ehrlich, S.; Krieg, H. A consistent and accurate ab initio parametrization of density functional dispersion correction (DFT-D) for the 94 elements H-Pu. *J. Chem. Phys.* **2010**, *132*, 154104.
4. Weigend, F.; Ahlrichs, R. Balanced basis sets of split valence, triple zeta valence and quadruple zeta valence quality for H to Rn: Design and assessment of accuracy. *Phys. Chem. Chem. Phys*. **2005**, *7*, 3297-3305.
5. Klamt, A.; Schüürmann, G. COSMO: a new approach to dielectric screening in solvents with explicit expressions for the screening energy and its gradient. *J. Chem. Soc. Perkin Trans.2.* **1993**, *2*, 799-805.
6. Gaussian 16, Revision A.03, M. J. Frisch, et al. Gaussian, Inc., Wallingford CT, **2016**.
7. Wang, D.; Guo, Q.; Wu, Z.; Li, M.; He, B.; Du, Y.; Zhang, K.; Tao, Y. Molecular mechanism of antihistamines recognition and regulation of the histamine H1 receptor. *Nat. Commun*. **2024**, *15*, 84-84.
8. Košak, U.; Knez, D.; Coquelle, N.; Brus, B.; Pišlar, A.; Nachon, F.; Brazzolotto, X.; Kos, J.; Colletier, J.-P.; Gobec, S. *N*-Propargylpiperidines with napthalene-2-carboxamide or napthalene-2-sulfonamide moieties: Potential multifunctional anti-Alzheimer agent. *Bioor. Med. Chem.* **2017**, *25*, 633-645.
9. Halgren, T. A.; Murphy, R. B.; Friesner, R. A.; Beard, H. S.; Frye, L. L.; Pollard, W. T.; Banks, J. L. Glide: A New Approach for Rapid, Accurate Docking and Scoring. 2. Enrichment Factors in Database Screening. *J. Med. Chem.* **2004**, *47*, 1750-1759.
10. Friesner, R. A.; Banks, J. L.; Murphy, R. B.; Halgren, T. A.; Klicic, J. J.; Mainz, D. T.; Repasky, M. P.; Knoll, E. H.; Shelley, M.; Perry, J. K.; Shaw, D. E.; Francis, P.; Shenkin, P. S. Glide: A New Approach for Rapid, Accurate Docking and Scoring. 1. Method and Assessment of Docking Accuracy. *J. Med. Chem.* **2004**, *47*, 1739-1749.
